# Long-read genome sequencing reveals the sequence characteristics of pear self-incompatibility locus

**DOI:** 10.1186/s43897-024-00132-0

**Published:** 2025-03-01

**Authors:** Chao Gu, Ying Xu, Lei Wu, Xueping Wang, Kaijie Qi, Xin Qiao, Zewen Wang, Qionghou Li, Min He, Shaoling Zhang

**Affiliations:** Saya Institute of Nanjing Agricultural University, Nanjing Agricultural University, Nanjing, 211800 China

**Keywords:** *Pyrus*, Self-incompatibility, *S-*Locus, *F-Box* gene, Non-self-recognition, Cross-recognition

## Abstract

The S-RNase-based self-incompatibility locus (*S*-locus) in *Petunia* species contains 16–20 *F-box* genes, which collaboratively function in the recognition and subsequent degradation of non-self S-RNases, while distinguishing them from self S-RNase. However, the number of *S*-locus* F*-*box* genes (*SFBB*s) physically interacted with non-self S-RNases remains uncertain in *Pyrus* species. Utilizing Pacbio long-read sequencing, we successfully assembled the genome of pear cultivar ‘Yali’ (*Pyrus bretschneideri*), and identified 19 *SFBB*s from the *Pyrus S*_*17*_-locus spanning approximately 1.78 Mb. Additionally, we identified 17–21 *SFBB*s from other *Pyrus* and *Malus S*-loci spanning a range of 1.35 to 2.64 Mb. Based on the phylogenetic analysis, it was determined that *Pyrus* and *Malus SFBB*s could be classified into 22 groups, denoted as I to XXII. At amino acid level, *SFBB*s within a given group exhibited average identities ranged from 88.9% to 97.9%. Notably, all 19 *SFBB*s from the *S*_*17*_-locus co-segregated with *S*_*17*_*-RNase*, with 18 of them being specifically expressed in pollen. Consequently, these 18 pollen-specifically expressed *SFBB*s are considered potential candidates for the pollen-*S* determinant. Intriguingly, out of the 18 pollen-specifically expressed *SFBB*s, eight demonstrated interactions with at least one non-self S-RNase, while the remaining SFBBs failed to recognize any S-RNase. These findings provide compelling evidence supporting the existence of a collaborative non-self-recognition system governing self-incompatibility in pear species.

**Supplementary Information:**

The online version contains supplementary material available at 10.1186/s43897-024-00132-0.

## Core

A total of 17-21 *S-locus F-box brothers* (*SFBB*s) were clarified in *Pyrus* and *Malus S*-loci. Of the 19 *SFBB*s in *Pyrus S*_*17*_-locus, 18 are specifically expressed in pollen and eight of them interact with non-self S-RNase. SFBB.VIII is a universe factor in recognizing non-self S-RNases.

## Gene & accession numbers

PbrSFBB.Ia-S_17_ accession: ON918613, PbrSFBB.Ib-S_17_ accession: ON918621, PbrSFBB.II-S_17_ accession: ON918614, PbrSFBB.III-S_17_ accession: ON918615, PbrSFBB.IV-S_17_ accession: ON918616, PbrSFBB.V-S_17_ accession: ON918617, PbrSFBB.VI-S_17_ accession: ON918618, PbrSFBB.VII-S_17_ accession: ON918619, PbrSFBB.VIII-S_17_ accession: ON918620, PbrSFBB.X-S_17_ accession: ON918622, PbrSFBB.XI-S_17_ accession: ON918623, PbrSFBB.XII-S_17_ accession: ON918624, PbrSFBB.XIII-S_17_ accession: ON918625, PbrSFBB.XIV-S_17_ accession: ON918626, PbrSFBB.XV-S_17_ accession: ON918627, PbrSFBB.XVI-S_17_ accession: ON918628, PbrSFBB.XVII-S_17_ accession: ON918629, PbrSFBB.XVIII-S_17_ accession: ON918630.

## Introduction

Self-incompatibility (SI) is a prevalent genetic mechanism that facilitates the rejection of self-pollen by the pistil as a means to prevent inbreeding. This mechanism is observed in around 40% of flowering plant species across at least 100 families (de Nattancourt, 2001; Igic et al. 2008). SI can be categorized into two main types: sporophytic SI, primarily found in Brassicaceae family (Hiscock and Mclnnis 2003), and gametophytic SI (GSI), extensively studied in Rosaceae, Solanaceae, and Plantaginaceae families (Fuji et al., 2016). In these three families, GSI is controlled by an *S* locus that comprises genes encoding an S-RNase expressed in pistils and multiple S-locus F-box (SLF/SFB) proteins expressed in pollen (Ushijima et al. 2003; Kao and Tsukamoto, 2004; Franklin-Tong 2008; Kubo et al., 2010; Sun et al. 2018). Upon entering the pollen tube, the S-RNase has the potential to impede its growth if the haploid pollen's S-haplotype is identical to any of the S-haplotypes present in the style (Chen et al. 2018). The SLF/SFB proteins, as constituents of Skp1-Culin1-F-box protein degradation complex, facilitate the degradation of non-self S-RNase through E3 ligase-mediated ubiquitination (Hua and Kao 2006).

During the GSI reaction, the SLF/SFB proteins exhibit two distinct mechanisms for recognizing S-RNase (Fuji et al., 2016). Self-recognition is observed specifically in *Prunus* species, where the unique pollen SLF/SFB protein can selectively identify self S-RNase, thereby impeding ubiquitination and degradation mediated by other SLFs (Li et al. 2020). Non-self-recognition is reported in *Petunia* species, where self S-RNase failed to be recognized by any of multiple SLFs, while non-self S-RNases are recognized by at least one SLF (Kubo et al., 2010; Song et al. 2023; Sun et al. 2018). It is worth noting that certain SLFs may not interact with any of the tested S-RNases (Sun et al. 2018), suggesting their lack of involvement in non-self-recognition. Furthermore, the non-self recognition mechanism is also suggested in pear (*Pyrus pyrifolia)* species, wherein pollen bearing the *S*_*4*_-haplotype and lacking an *F-box* gene (*SFBB1-S*^*4*^) is rejected by the style containing *S*_*1*_-haplotype (Kakui et al. 2011). However, the specific number of F-box proteins capable of recognizing S_1_-RNase and the role of SFBB1-S^4^ in the degradation of S_1_-RNase remain unclear.

The *S*-locus in *Pyrus* and *Malus* species is believed to encompass multiple *F-box* genes for each *S*-haplotype (Kakui et al. 2011). Through the utilization of bacterial artificial chromosome (BAC) libraries, a total of 12 and 10 *F-box* genes were identified from the *Malus S*_*3*_- and *S*_*9*_-locus, respectively (Minamikawa et al. 2010). Similarly, 10 and six *F-box* genes were identified from the *Pyrus S*_*2*_- and *S*_*4*_-locus, respectively, and designated as *S*-locus *F-box brother* genes (*SFBB*s; Okada et al. 2011). In addition, a total of 27 *F-box* genes were identified from the two *Pyrus S*-locus (*S*_*21*_ and *S*_*34*_; Huang et al. 2023). In *Petunia* species, 17 *F-box* genes were identified from approximately 3.1 Mb within the *S*_*2*_-locus (Wu et al. 2020), while 16–20 *SLF* genes were identified from other *S*-haplotypes (Williams et al. 2014; Kubo et al. 2015). However, the repetitive nature of Solanaceae *S*-locus sequences poses challenges in accurately assembling the *S*-locus using short-read sequences (Wu et al. 2020). A recent study successfully assembled the *Antirrhinum* genome using PacBio long-read sequencing, resulting in the identification of 32 *SLF* genes from two *S*-haplotypes spanning approximately 1.2 Mb (Zhu et al., 2023). Thus, PacBio long-read sequencing is a reliable method for determining the size of the *S*-locus region and identifying the *S*-locus *F-box* genes. In a previous investigation, the *S*_*34*_*-RNase* detected in *Pyrus bretschneideri* ‘Yali’ was renamed as *S*_*17*_*-RNase* due to identical sequences (Wang et al. 2017). In this study, the reference genome of ‘Yali’ were assembled by using PacBio long-read sequences to elucidate the size of the *S*_*17*_-locus region and to identify the *F-box* genes within the region. Furthermore, protein–protein interaction was performed to investigate the cross-recognition between SFBBs and self/non-self S-RNases. The findings from this study contribute to a better understanding of the collaborative non-self recognition system in S-RNase-based GSI.

## Results

### Identification of S_17_-locus using the genome sequencing of ‘Yali’

**Fig. 1:**
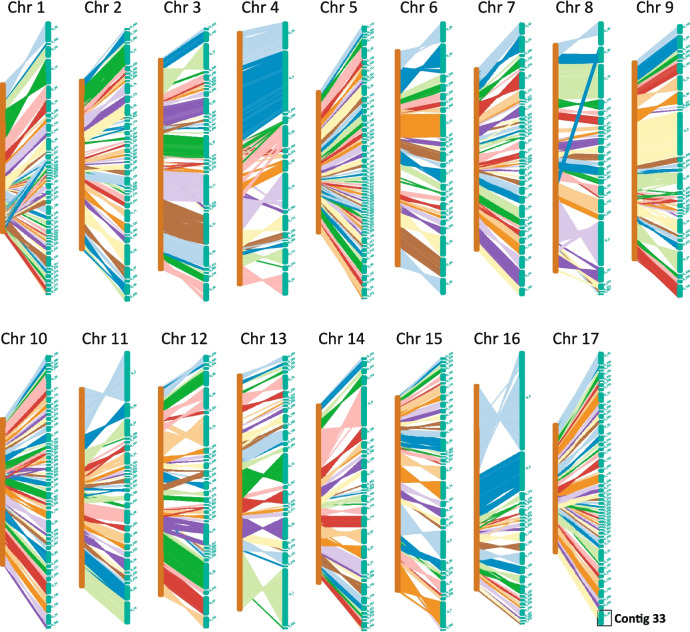
Mapping of ‘Yali’ contigs to the ‘Cuiguan’ genome. The columns with brown color are the chromosomes of ‘Cuiguan’ genome, while the columns with green color are the contigs of ‘Yali’ assembly. In each chromosome, the contigs are linked with the homologous regions of ‘Cuiguan’ genome by the lines with different colors. A total of 566 contigs could be mapped to the ‘Cuiguan’ genome

To develop a de novo assembly of ‘Yali’ genome, we initially generated 63.57 Gb of Illumina paired-end reads. Based on the 17-mer spectrum, the estimated genome size was 545.39 Mb with a heterozygosity rate of 1.35%. Subsequently, 34.44 Gb of long-reads (a mean length of 9,721 bp) were generated using Ilumina PacBio platform. The PacBio reads were self-corrected and assembled into genomic contigs using FALCON (Chin et al. 2016), and then polished with the Illumina paired-end reads using pilon software (Walker et al. 2014). The final assembly reached 534.45 Mb and consisted of 582 contigs, with an N50 of 1.81 Mb (Table S1). Of these contigs, 566 could be mapped to and almost cover all chromosomes in ‘Cuiguan’ genome (Fig. 1).

The completeness of the assembly was assessed using benchmarking universal single-copy orthologs (BUSCO; Simão et al. 2015), which revealed that 95.2% of the universal single-copy orthologs were present (Table S1), indicating a reliable assembly. A total of 262.46 Mb of repetitive elements accounted for 49.11% of assembly (Tables S2), and approximately 293.95 Kb of non-coding RNAs accounted for 0.06% of the assembly (Tables S3). Moreover, the total of 2438 non-coding RNAs were identified, including 745 microRNAs, 720 transfer RNAs, 155 ribosomal RNAs, and 818 small nuclear RNAs. Furthermore, a total of 45,092 protein-coding genes were predicted from the assembly (Table S1). Notably, the *S*_*17*_*-RNase* was annotated in the contig 33 (Fig. 1). These results suggest a reliable completeness of the assembly.

### Identification of the F-box genes in Pyrus S_17_-locus

**Fig. 2:**
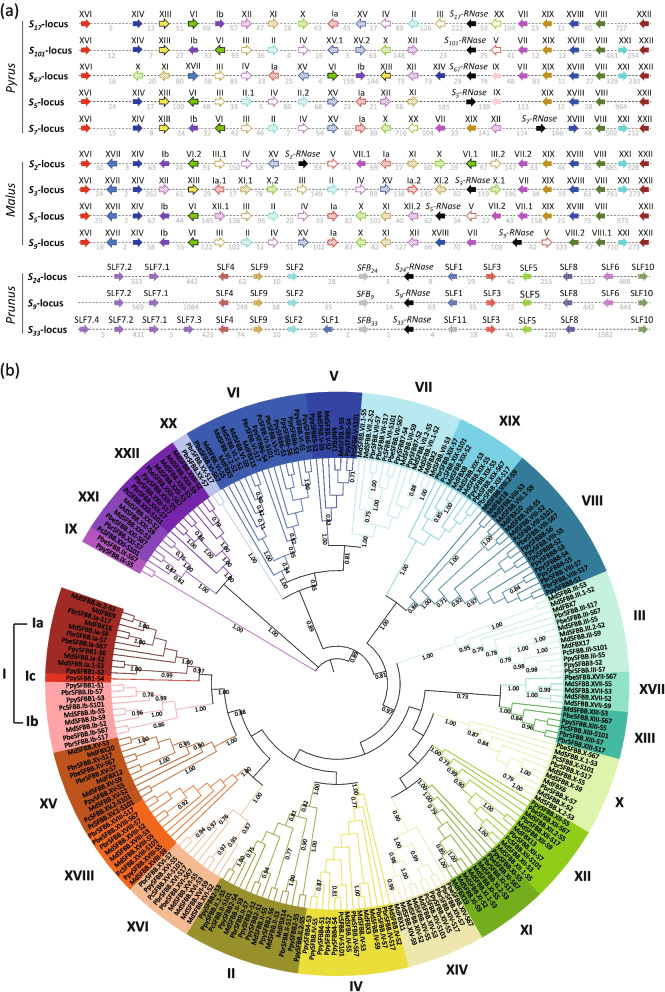
Sequence analysis of the *F-box* genes in *Pyrus* and *Malus S*-loci. **a** Arrangement of *F-box* genes in *Pyrus*, *Malus*, and *Prunus S*-loci. The direction of gene transcription is indicated by arrowheads, wherein black-colored arrowheads represent the *S-RNase* gene, and other colored arrowheads represent *S-locus F-box* genes. The characters above the arrowheads denote the classification of *S-locus F-box* genes in *Pyrus*, *Malus*, and *Prunus* species. The physical distance (in kilobases, Kb) between adjacent *F-box* genes is indicated below the dotted line, while the chromosome locations of these *F-box* genes are provided in Table S4. It should be noted that the abbreviation "SLF" specifically applies to *S-locus F-box* genes in Solanaceae, Plantaginaceae, and *Prunus*. **b** Phylogenetic classification of S-locus F-box brother (SFBB) proteins in *Pyrus* and *Malus*. The I → XXII labels signify the different groups of classified *S*-locus F-box proteins, with Group I including the sub-groups Ia, Ib, and Ic. All groups and sub-groups are highlighted in distinct colors

To identify the *F-box* genes within the *S*_*17*_-locus, a blasting search was performed in the contig 33 of the ‘Yali’ genomes using the conserved F-box motif (Figure S1) of *Pyrus* and *Malus SFBB*s as query sequences. This search yielded 19 *SFBB*s, each encoding more than 150 amino acids, within the contig 33 (Fig. 2a; Table S4), which is a larger number than the 15 *SFBB*s reported in a previous study that identified *SFBB*s within the same *S*-locus (Huang et al. 2023). Notably, these *SFBB*s were found within the region from 0.94 Mb upstream and 0.83 Mb downstream of *S*_*17*_*-RNase* (Tables S4 and S5). This finding indicates that *Pyrus SFBB*s are distributed within a genomic region of less than 4 Mb, probably.

Consequently, a subsequent blasting search was performed in the region from approximately 2 Mb upstream to 2 Mb downstream of the *S-RNase* in the *Pyrus* and *Malus* reference genomes that has been previously assembled using long-read sequences (Daccord et al. 2017; Linsmith et al., 2019; Zhang et al. 2019; Dong et al. 2020; Sun et al. 2020; Gao et al. 2021). The results showed that in *Pyrus* species, 19, 17, and 20 *SFBB*s were identified from *P. communis S*_*101*_-locus (*PcS*_*101*_-locus), *P. pyrifolia S*_*5*_-locus (*PpyS*_*5*_-locus), and *P. betuleafolia S*_*67*_-locus (*PbeS*_*67*_-locus), respectively (Fig. 2a; Table S4). In the case of *Malus domestica*, 21, 21, 20, and 20 *SFBB*s were identified from *S*_*2*_-, *S*_*3*_-,* S*_*5*_-, and *S*_*9*_-locus, respectively (Fig. 2a; Table S4). Furthermore, the short-read sequences from the *P. bretschneideri* cultivar ‘Dangshansuli’ (*S*_*7*_*S*_*17*_) genome (Shi et al., 2019; Wu et al., 2013) were mapped onto contig 33, and 20 *SFBB*s were identified from the remaining *S*_*7*_-locus (Fig. 2a; Table S4). These findings indicate that *Pyrus* and *Malus S*-loci contain 17–21 *SFBB*s. In contrast, *Prunus salicina S*_*33*_-locus, *Prunus avium S*_*9*_-locus, and *Prunus armeniaca S*_*24*_-locus displayed the identification of 14, 12, and 12 *SLF*s*/SFB*s, respectively (Fig. 2a; Table S4).

### Sequence analysis of protein-coding SFBBs

Phylogenetic classification of the 177 SFBBs derived from *Pyrus* and *Malus S*-haplotypes was performed based on their amino acid sequences. The SFBBs were classified into 22 distinct groups, denoted as groups I to XXII (Fig. 2b). Notably, groups I to VIII were found to correspond to the previously reported groups 1 to 8 (Kakui et al. 2011). Among these groups, group I exhibited further subdivision into sub-groups Ia, Ib, and Ic (Fig. 2b). As shown in Fig. 2a, *SFBB.XVIII* is positioned closest to the *SFBB*s in group XXI and/or XXII at one end of the *S*-locus in any *S*-haplotype, while *SFBB.XVII* is found closest to *SFBB.XVI* at another end but is absent in *Pyrus S*-locus. *SFBBXXI* is located between *SFBBVIII* and *SFBBXXII*, though it has been occasionally deleted. The remaining *SFBB*s appear to be randomly arranged within *Pyrus* and *Malus S*-loci (Fig. 2a). In contrast, the *F-box* genes within *Prunus S*-locus were grouped into 11 clusters, designated as SLF1 to SLF10 and SFB (Figure S2), with relatively stable positions (Fig. 2a).

The nucleotide acid sequence of all *F-box* genes present in the ‘Yali’ genome was utilized for phylogenetic analysis in conjunction with the 27 *SFBB*s previously identified in ‘Yali’ pollen (Huang et al. 2023). The results showed that all 27 *SFBB*s were grouped together with the 19 *SFBB*s identified in the *Pyrus S*_*17*_-locus (Figure S3). Furthermore, the *SFBB*s previously identified from the *Pyrus S*_*34*_-locus (which had been renamed as *S*_*17*_*-*locus; Wang et al. 2017) were closest clustered with those identified in the ‘Yali’ genome (Figure S3). Remarkably, the bootstrap values reached 100 for *PbrSFBB.XIX-S*_*17*_ and *PbrSLF16-S*_*21*_, *PbrSFBB.VI-S*_*17*_ and *PbrSLF6-S*_*21*_, as well as *PbrSFBB.VIII-S*_*17*_ and *PbrSFBB8-S*_*21*_ (Figure S3). This high bootstrap value indicates that the three *SFBB* genes, namely *PbrSLF6-S*_*21*_, *PbrSFBB8-S*_*21*_, and *PbrSLF16-S*_*21*_, are potentially located within the *S*_*17*_-locus.

The level of sequence polymorphism observed within each group of *SFBB*s was relatively low. At nucleotide acid level, the average identities ranged from 92.1% to 98.4%, while at amino acid level, the average identities ranged from 88.9% to 97.9% (Tables 1 and S6). The sequence identities among *SFBB*s within each group were similar to those observed among *Prunus SLF*s, but greater than those observed among *Prunus SFB*s and *Pyrus S-RNase*s (Tables S7 to S9). Notably, the inter-group identity of *SFBB*s was significantly lower than the intra-group identity of *SFBB*s (*p*-value = 0.0022 < 0.05). Across groups I to XXII, the average identities among *SFBB*s ranged from 12.3% to 84.0% at nucleotide acid level and from 0.06% to 71.8% at amino acid level (Tables 1 and S6). These values were closely resemble the sequence identities observed among *S-RNase*s (Table 1).

**Table 1:** Sequence similarity of *F-box* genes in *Pyrus*, *Malus*, and *Prunus S*-haplotypes

Group			Amino acid (%)			Nucleotide acid (%)		
Type	A	B	Min	Max	Average	Min	Max	Average
Intra-group	Ia	Ia	88.7	100.0	92.5	93.0	100.0	95.5
	Ib	Ib	93.0	100.0	95.5	96.4	99.8	97.5
	II	II	84.1	95.9	90.1	88.2	97.1	93.7
	III	III	89.5	100.0	93.5	94.3	100.0	96.4
	IV	IV	89.5	95.1	92.8	94.7	97.6	96.1
	V	V	95.1	96.6	95.7	96.6	97.2	96.8
	VI	VI	91.5	98.9	94.6	94.4	99.2	96.8
	VII	VII	78.9	100.0	90.5	86.4	100.0	93.7
	VIII	VIII	77.8	99.7	91.0	87.1	99.4	95.5
	X	X	87.7	100.0	93.3	86.4	96.9	93.3
	XI	XI	79.3	100.0	88.9	85.8	100.0	92.1
	XII	XII	86.5	100.0	91.0	90.1	100.0	93.5
	XIII	XIII	88.2	97.9	94.3	94.1	99.1	96.7
	XIV	XIV	83.0	100.0	93.3	89.7	100.0	95.5
	XV	XV	88.1	99.7	92.3	93.9	99.8	95.3
	XVI	XVI	96.4	100.0	97.6	97.5	100.0	98.4
	XVII	XVII	96.2	100.0	97.9	97.0	100.0	98.3
	XVIII	XVIII	94.6	99.2	97.5	95.7	99.3	97.8
	XIX	XIX	92.4	97.5	95.3	96.3	99.6	97.6
	XXI	XXI	95.0	100.0	96.5	94.6	99.8	97.2
	XXII	XXII	91.7	100.0	95.0	94.3	100.0	97.0
Inter-group	I → VIII	I → VIII	55.9	92.6	69.5	55.7	93.2	77.8
	I → VIII	IX	36.0	98.1	57.8	77.4	99.1	84.0
	I → VIII	X → XX	50.3	82.4	68.3	49.6	89.5	80.2
	X → XX	IX	21.1	89.9	56.0	40.9	88.6	74.4
	X → XX	X → XX	50.1	87.3	71.8	50.0	89.7	81.6
	XXI → XXII	I → VIII	4.1	13.4	8.5	18.7	33.1	21.8
	XXI → XXII	IX	5.7	37.8	20.3	9.9	33.5	21.5
	XXI → XXII	X → XX	3.7	37.8	0.06	12.5	37.5	22.3
	XXI	XXII	3.4	5.4	3.9	9.8	22.8	12.3
S-RNase			62.0	78.0	67.3	77.5	87.6	81.0

Long-read sequence analysis was utilized to examine gene duplication events in the *Pyrus* and *Malus* genomes. The findings revealed the presence of dispersed, proximal, tandem, and transposed duplications within various *S*-loci, such as *Pyrus S*_*17*_-locus and other *S*-locus, as well as *Malus S*_*3*_-locus *and Pyrus S*_*5*_-, *S*_*67*_-, and* S*_*101*_-locus (Figure S4; Table S10). For instance, in the *Pyrus S*_*17*_-locus, the tandem duplicate *SFBB.X* and *SFBB.XI* were found adjacent to each other without any intervening genes. The proximal duplicate *SFBB.X* and *SFBB.Ib* were separated by a few genes, and the dispersed duplicate *SFBB.XI* and *SFBB.XIII* were not adjacent and did not share a homologous segment. In the *Pyrus S*_*67*_-locus, it was observed that the transposed duplicate SFBB.XI and SFBB.XIV may have been relocated by DNA transposons. Additionally, through synteny analysis, it was determined that deletions, inversions, and/or translocations of these *SFBB*s were frequently observed between any two *S*-locus (Figures S5 to S11).

### Comparison analysis of the non-coding flanking sequences of SFBBs

**Fig. 3:**
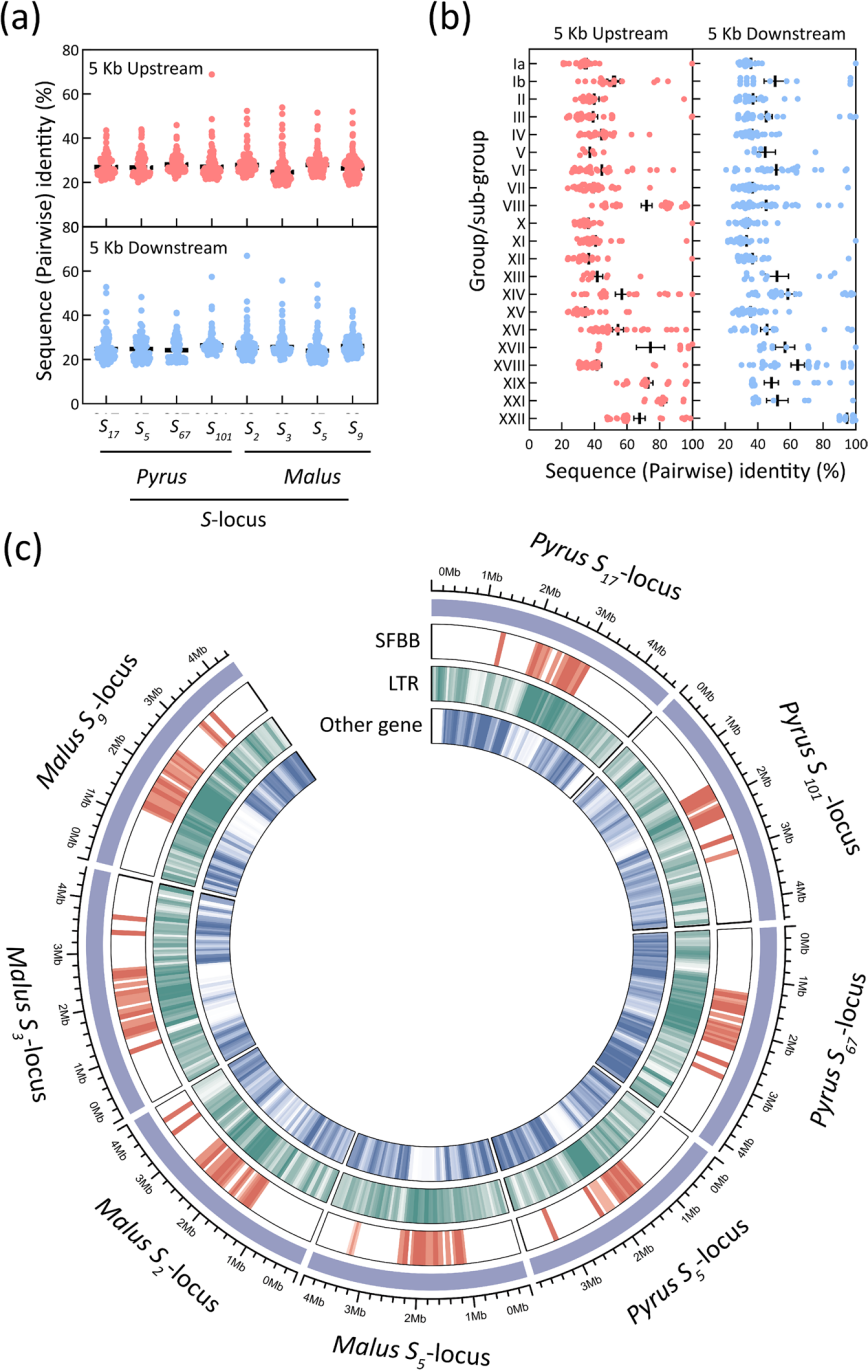
Comparison analysis of the non-coding sequences in *Pyrus* and *Malus S*-loci. **a** Pairwise identity of the 5 kb upstream and downstream sequences of *SFBB*s within each group and sub-group. The specific data can be found in Figures S11 to S31. **b** Pairwise identity of the 5 kb upstream and downstream sequences of *SFBB*s in *Pyrus* and *Malus S*-loci. The corresponding data can be found in Figures S32 to S39. **c** Location of *SFBB*s, LTRs, and other genes within *Pyrus* and *Malus S*-loci. The outer region is the length of DNA sequences that contain *S*-locus. *SFBB*s, LTRs, and other genes are depicted in red, green, and blue colors, respectively. The phylogenetic tree was constructed using MEGA5 with the neighbor-joining method. Bootstrap values were calculated from 1000 replicates

In order to investigate the sequence polymorphism of the non-coding flanking sequences of *SFBB*s, an analysis was conducted on the 5 kb upstream and 5 kb downstream sequences of *SFBB*s within each *S*-locus and group (Figures S12 to S40). The results showed that the identities between either the upstream or downstream sequences of all *SFBB*s in any *S*-locus were lower (Figs. 3a and S12 to 19), compared to those between all *SFBB*s within any group (Figs. 3b and S20 to S40; Table S11). Particularly, in group XXII, the downstream sequences displayed extremely high identities (> 90%) between any two *SFBB*s (Figs. 3b and S40d), as well as the upstream sequences between any two *Malus SFBB*s (Figure S40b), which closely resembled those found in the coding sequences of *SFBB*s (Table 1). However, although a few pairwise *SFBB*s in groups III (Figure S23b,d), VIII (Figure S28b,d), XI (Figure S30b,d), XII (Figure S31b,d), XIV (Figure S33b,d), XVI (Figure S35b,d), XVII (Figure S36b,d), XVIII (Figure S37b,d), and XIX (Figure S38b,d), as well as sub-group Ia (Figure S20b,d), exhibited extremely high identities in either the upstream or downstream sequences, the average identity in the upstream or downstream sequences between *SFBB*s in any group (excluding XXII) was lower than in the coding sequences of *SFBB*s (Figs. 3b; Tables 1 and S11). These results demonstrate that genetic variation frequently occurs in the non-coding flanking sequences of *Pyrus* and *Malus SFBB*s.

The significant role of long terminal repeat (LTR) retrotransposon in genetic recombination is widely acknowledged (Kumar and Bennetzen 1999). In this study, a survey of LTR retrotransposons within the *S*-loci of *Pyrus* and *Malus* species revealed that the total length of LTR retrotransposons accounted for approximately 13.49% of the tested regions (Table S12). A total of 568, 459, 521, and 515 LTR retrotransposons were detected in *Pyrus S*_*17*_-, *S*_*5*_-, *S*_*67*_-, and *S*_*101*_-locus, respectively, and 459, 633, 665, and 650 LTR retrotransposons were detected in *Malus S*_*2*_-, *S*_*3*_-, *S*_*5*_-, and *S*_*9*_-locus, respectively (Tables S12 and S13). On average, the number of LTR retrotransposons was 1.64 times (*p-*value < 0.001) that of the genes in the tested region (Table S12). Notably, the number of LTR retrotransposons was 4.26 times (*p-*value < 0.001) that of the genes in the region between *SFBB.XVI* and *SFBB.VIII*, but only 1.09 times (*p-*value = 0.55) that of the genes in the other region (Table S12). This result indicated that the density of LTR retrotransposons in the region between *SFBB.XVI* and *SFBB.VIII* was higher (3.89-fold;* p-*value < 0.005) compared to the other region (Fig. 3c). The elevated density of LTR retrotransposons between *SFBB.XVI* and *SFBB.VIII* may be associated with the genetic variations, such as gene duplication, deletion, inversion, and translocation of *SFBB*s in *Pyrus* and *Malus* species.

### Inheritance and tissue specific expression of SFBBs in Pyrus S_17_-locus

**Fig. 4:**
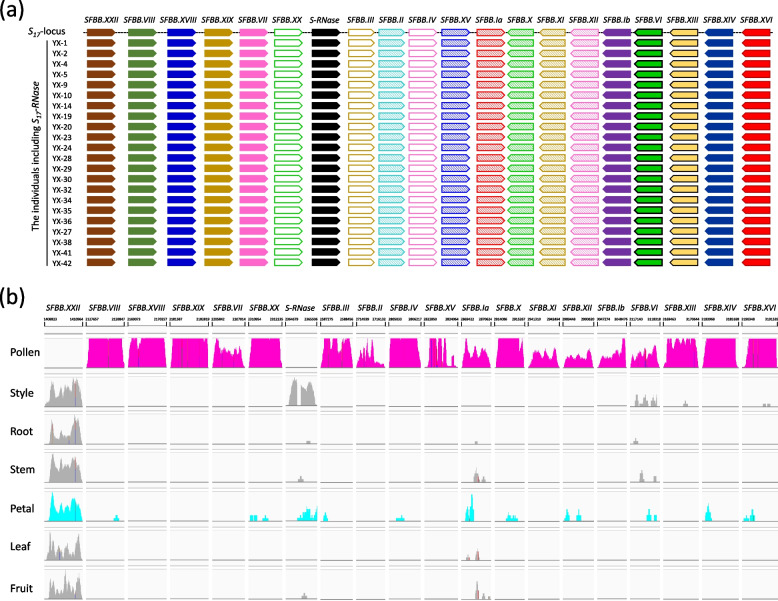
The co-segregation of pollen-specifically expressed *SFBB*s from the *Pyrus S*_*17*_-locus with *S*_*17*_*-RNase*. **a** A diagram shows the alignments of 19 *SFBB*s in the ‘Yali’ genome and in the hybrids containing* S*_*17*_*-RNase*. **b** Integrative Genomics Viewer (IGV) snapshots show the transcript levels of *S-RNase* and *SFBB* genes from the *Pyrus S*_*17*_-locus in various pear tissues. The pollen-specifically expressed *SFBB*s are highlighted in pink color. Detailed sequence alignments can be found in Figures S42 to S60

To investigate the inheritance pattern of *SFBB*s within the *Pyrus S*_*17*_-locus, a total of 42 individuals were selected from the progeny resulting from a cross between ‘Yali’ (*S*_*17*_*S*_*21*_) and ‘Xueqing’ (*S*_*3*_*S*_*16*_). By analyzing the segregation of *S-RNase* alleles, four different *S*-genotypes in this progeny, which aligned with the expected 1:1:1:1 ratio (χ^2^ = 0.286 < χ^2^_0.05, 3_ = 7.815; Figure S41). Furthermore, 19 pairs of primer were employed to amplify the full-length sequences of the 19 *SFBB*s within the *Pyrus S*_*17*_-locus. The resultant amplification products were subsequently cloned and sequenced. Alignment of the sequences revealed the presence of all *SFBB*s exclusively in the hybrids harboring the *S*_*17*_*-RNase* allele (Fig. 4a and S42 to S60). This finding strongly indicates that the *SFBB*s within the *Pyrus S*_*17*_-locus exhibit co-segregation with *S*_*17*_*-RNase*.

To reveal the expression pattern of the 19 *SFBB*s within the *Pyrus S*_*17*_-locus across various tissues, the transcriptome sequence reads from ‘Dangshansuli’ were utilized (Shi et al. 2017; Qiao et al. 2018; Li et al., 2019; Wu et al. 2023). These reads were previously generated and mapped to the genome of ‘Yali’. The analysis showed that 18 *SFBB*s exhibited specific expression in pollen, whereas *S*_*17*_*-RNase* displayed specific expression in the style (Fig. 4b and S61). Notably, the remaining SFBB, *PbSFBB.XXII-S*_*17*_, exhibited expression in the style, root, stem, petal, leaf, and fruit, but not in pollen (Figs. 4b and S61). These findings suggest that the 18 *SFBB*s specifically expressed in pollen are the candidates of pollen-*S* determinant and may directly interact with non-self S-RNase.

### Physical interaction between S-RNases and SFBBs

**Fig. 5:**
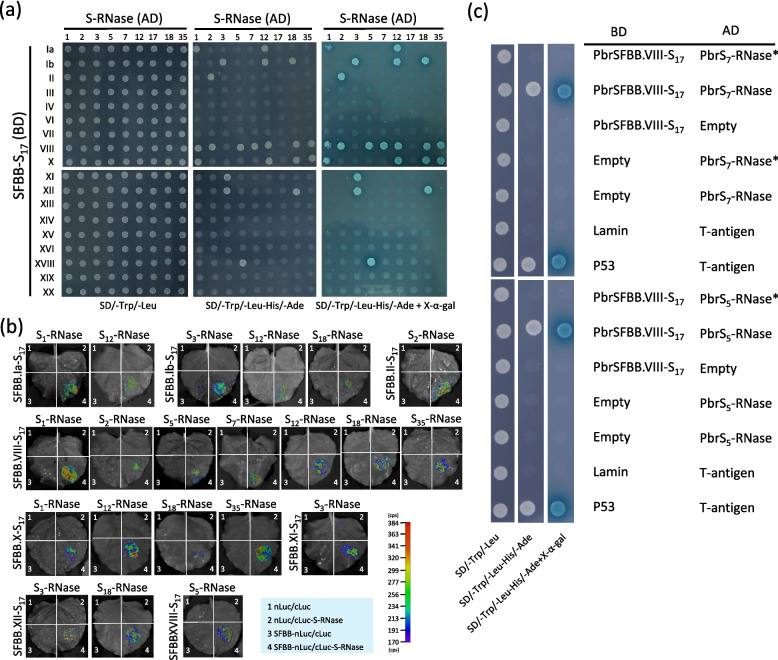
Interaction of self and non-self S-RNases with the pollen-specifically expressed SFBBs from the *Pyrus S*_*17*_-locus. **a** Yeast-two-hybrid assay showing the interaction of 18 SFBBs with 9 S-RNases. **b** Firefly luciferase complementation assay were performed between the potentially interacting proteins identified by the yeast-two-hybrid assay. The intensity of the interaction is depicted using different colors, which can be evaluated using the color column ruler. **c** Yeast-two-hybrid assay showed that S-RNases lacking the HV region (S_5_-RNase* and S_7_-RNase*) are unable to interact with PbSFBB.VIII-S_17_. AD and BD represent the pGADT7 and pGBKT7 vectors, respectively. Arabic numeral indicates the serial number assigned to each S-RNase. Ia, Ib, and II → XX are the serial numbers assigned to SFBBs in the *Pyrus S*_*17*_-locus (SFBB-S_17_). SD/-Trp/-Leu denotes the SD medium lacking Trp and Leu; SD/-Trp/-Leu/-His/-Ade denotes the SD medium lacking Trp, Leu, His, and Ade; SD/-Trp/-Leu-His/-Ade + X-α-gal indicates the addition of x-a-gal to the SD medium lacking Trp, Leu, His, and Ade. P53/T-antigen represents the positive controls, while Lamin/T-antigen represents the negative control

To examine the potential interaction between SFBBs and self/non-self S-RNases, yeast-two-hybrid (Y2H) and firefly luciferase complementation imaging (FLCI) assays were conducted. The Y2H assay demonstrated the absence of self-activation for any S-RNase or SFBB within yeast cells (Figure S62). Notably, none of the SFBBs expressed in the *S*_*17*_-locus exhibited interaction with S_17_-RNase (Fig. 5a; Table 2). In contrast, eight SFBBs, PbrSFBB.Ia-S_17_, PbrSFBB.Ib-S_17_, PbrSFBB.II-S_17_, PbrSFBB.VIII-S_17_, PbrSFBB.X-S_17_, PbrSFBB.XI-S_17_, PbrSFBB.XII-S_17_, and PbrSFBB.XVIII-S_17_, were found to interact with at least one S-RNase (Fig. 5a, b). This finding further supports the involvement of a collaborative non-self-recognition system in the regulation of S-RNase-based GSI in pear. However, it is worth noting that 10 SFBBs did not exhibit any interaction with the tested S-RNases (Fig. 5a). This outcome may be attributed to the limited number of non-self S-RNases utilized in this particular study.

**Table 2:** Summary of the interaction of the SFBBs expressed in *Pyrus S*_*17*_-locus with self- and non-self S-RNases

Protein	S_1_-RNase	S_2_-RNase	S_3_-RNase	S_5_-RNase	S_7_-RNase	S_12_-RNase	S_17_-RNase	S_18_-RNase	S_35_-RNase
SFBB.Ia-S_17_	+	-	-	-	-	+	-	-	-
SFBB.Ib-S_17_	-	-	+	-	-	+	+	-	-
SFBB.II-S_17_	-	-	-	-	-	-	-	-	-
SFBB.III-S_17_	-	-	-	-	-	-	-	-	-
SFBB.IV-S_17_	-	-	-	-	-	-	-	-	-
SFBB.VI-S_17_	-	-	-	-	-	-	-	-	-
SFBB.VII-S_17_	-	-	-	-	-	-	-	-	-
SFBB.VIII-S_17_	+	+	-	+	+	+	-	+	+
SFBB.X-S_17_	+	-	-	-	-	+	-	+	+
SFBB.XI-S_17_	-	-	+	-	-	-	-	-	-
SFBB.XII-S_17_	-	-	+	-	-	-	-	+	-
SFBB.XIII-S_17_	-	-	-	-	-	-	-	-	-
SFBBXIV-S_17_	-	-	-	-	-	-	-	-	-
SFBB.XV-S_17_	-	-	-	-	-	-	-	-	-
SFBB.XVI-S_17_	-	-	-	-	-	-	-	-	-
SFBB.XVIII-S_17_	-	-	-	+	-	-	-	-	-
SFBB.XIX-S_17_	-	-	-	-	-	-	-	-	-
SFBB.XX-S_17_	-	-	-	-	-	-	-	-	-

Previous studies have showed the significance of the hypervariable (HV) region of S-RNase in mediating the recognition between S-RNase and pollen-*S* determinant (Matton et al. 1997, 1999; Wu et al. 2013). To test whether the HV region of *Pyrus* S-RNase is involved in the interaction between SFBBs and non-self S-RNases, an Y2H assay was conducted after removing the HV region from S_5_- and S_7_-RNases. The results showed that PbrSFBB.VIII-S_17_ was unable to interact with the HV region-deleted S_5_- and S_7_-RNases within yeast cells (Fig. 5c). This outcome suggests that the HV region of *Pyrus* S-RNase plays a crucial role in facilitating the cross-recognition of non-self S-RNases by the interacting SFBBs.

**Fig. 6:**
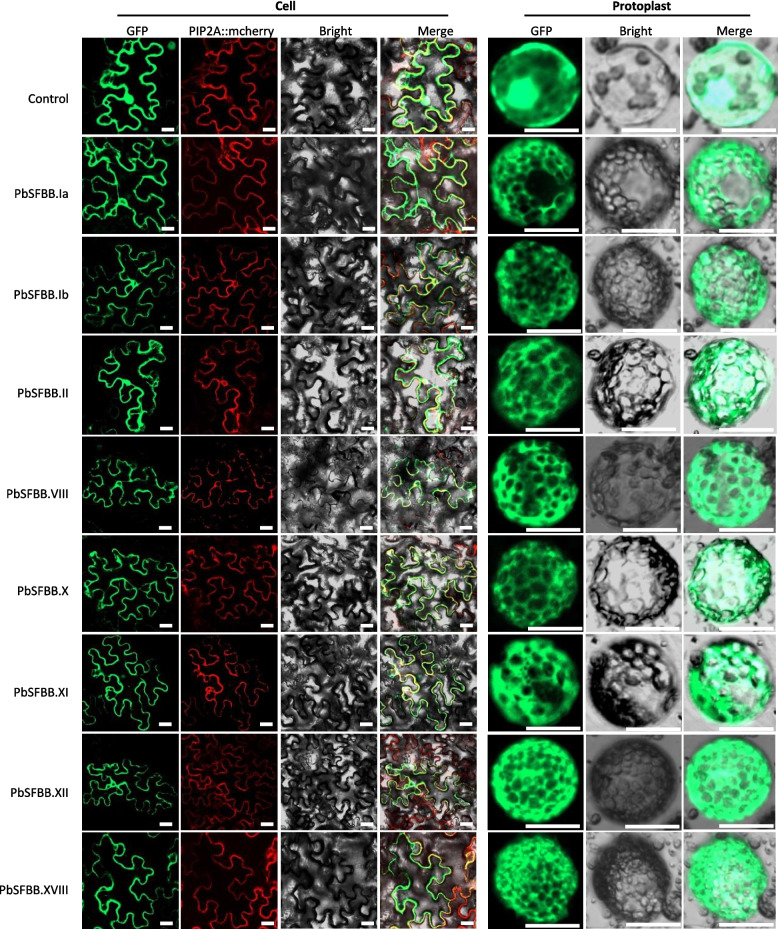
Subcellular localization of eight SFBBs physically interacting with non-self S-RNases in tobacco mesophyll cell and protoplast. PbSFBB.Ia-S17, PbSFBB.Ib-S17, PbSFBB.II-S17, PbSFBB.VIII-S17, PbSFBB.X-S17, PbSFBB.XI-S17, PbSFBB.XII-S17, and PbSFBB.XVIII-S17 are the SFBBs physically interacting with non-self S-RNases. Control is the empty vector. GFP represents the GFP-fused proteins. PIP2A::mcherry is a control for plasma membrane localization. Bright and Merge show the co-localization of GFP-fused protein and PIP2A::mcherry. Bars = 20 μm

To clarify the sub-cellular localization of the eight SFBBs physically interacting with non-self S-RNases, the SFBBs were fused with the green fluorescence protein GFP (SFBB::GFP). Arabidopsis aquaporin PIP2A fused with red fluorescence marker mCherry (PIP2A::mCherry) was used to show plasma membrane. Image analysis showed that in the control tobacco mesophyll cells co-transformed with PIP2A::mCherry and GFP empty vector, the GFP was detected in all visible tissues, while the PIP2A::mCherry was only detected in cell and nuclear membranes (Fig. 6). This result contributed to the presence of yellow fluorescence in cell and nuclear membranes in the merged images (Fig. 6). In the tobacco cells co-transformed with SFBB::GFP and PIP2A::mCherry, the GFP was detected in the inner side of yellow fluorescence in the merged images (Fig. 6), suggesting that all eight SFBBs may be localized in cytoplasm but not cell wall or cell membrane. To confirm it, protoplasts were extracted from the tobacco mesophyll cells. In the protoplast transformed with GFP empty vector, the GFP was detected in all visible tissues including cell membrane, cytoplasm, and nucleus (Fig. 6). In the protoplast transformed with SFBB::GFP, the GFP was only detected in cytoplasm (Fig. 6), suggesting that all eight SFBBs are localized in cytoplasm.

### The other genes in Pyrus S_17_-locus

**Fig. 7:**
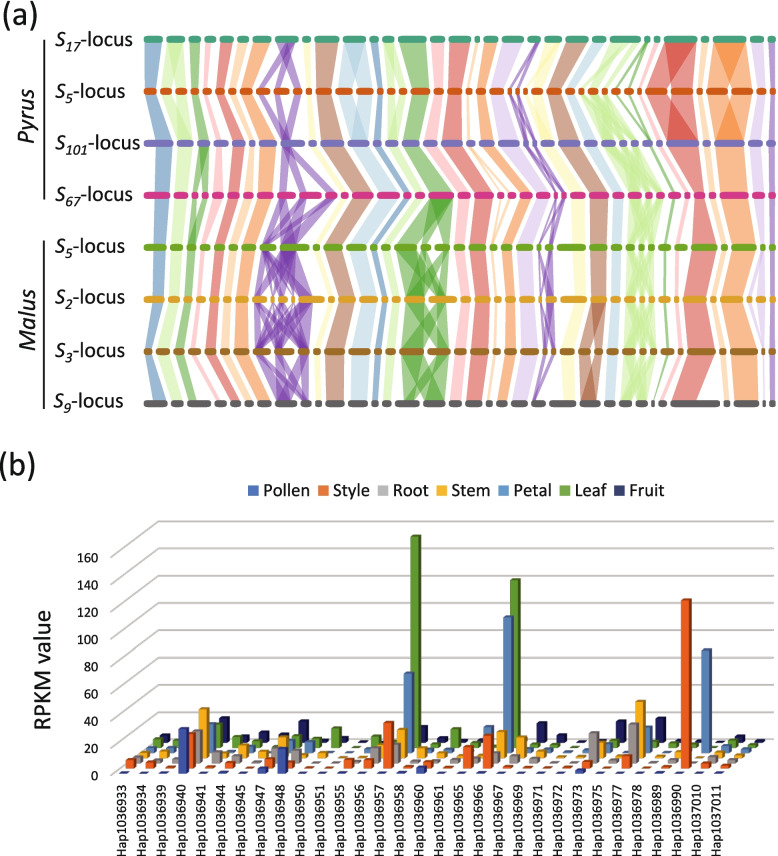
Identification and expression analysis of 29 genes shared in all tested *S*-loci. **a** Synteny of 29 genes across the tested *S*-loci. **b** Transcriptome analysis showing the gene expression level in various tissues, including pollen, style, root, stem, petal, leaf, and fruit. The Y-axis represents the PRKM value, while the X-axis represents the genes shared in all tested *S*-loci. The order of the 29 genes in the *S*_*17*_-locus is consistent between (**a**) and (**b**)

In the genome of ‘Yali’, besides *S-RNase* and *SFBB*s, a total of 114 genes were found between *PbrSFBB.XVI-S*_*17*_ and *PbrSFBB.XXII-S*_*17*_ (Table S5). Similarly, between *SFBB.XVI* and *PbSFBB.XXII* in *Pyrus S*_*101*_-, *Pyrus S*_*67*_-, *Pyrus S*_*5*_-, *Malus S*_*2*_-, *Malus S*_*3*_-, *Malus S*_*5*_-, and *Malus S*_*9*_-loci, there were 79, 153, 127, 156, 136, 138, and 115 genes, respectively (Table S5). Notably, 29 of these genes were shared between the *Pyrus S*_*17*_-locus and other *Pyrus* and *Malus S*-loci (Fig. 7a). Additionally, these 29 genes were consistently located between *SFBB.VIII* and *SFBB.XXII* in all *S*-locus. Transcriptome data analysis of various tissues revealed that 11 genes, *Serine/threonine-protein kinase CTR1* (evm.TU.G_33.131), *50S ribosomal protein* L1 (evm.TU.G_33.130), *zuotin-like* (evm.TU.G_33.107), *Ras-related protein Rab11D* (evm.TU.G_33.106), *ATP-dependent zinc metalloprotease FTSH2* (evm.TU.G_33.103), *Receptor protein kinase TMK1* (evm.TU.G_33.90), *U-box domain-containing protein 21* (evm.TU.G_33.89), *DELLA protein GAI* (evm.TU.G_33.76), *Protease Do-like 7* (evm.TU.G_33.37.2), *Phosphatidylinositol 4-kinase beta 1* (evm.TU.G_33.36), and *an uncharacterized protein* (evm.TU.G_33.130) were expressed (RPKM > 2) in style, but not in pollen (Fig. 7b; Table S14). These genes may be associated with the style growth and development. Moreover, five genes, *Multiprotein-bridging factor 1a* (evm.TU.G_33.125), *Shaggy-related protein kinase zeta* (evm.TU.G_33.119), *Aspartate aminotransferase* (evm.TU.G_33.115), *Nuclear poly(A) polymerase 1* (evm.TU.G_33.95), and *an uncharacterized protein* (evm.TU.G_33.81), were expressed in both pollen and style (Fig. 7b; Table S14), indicating their potential involvement in pollen tube growth and/or self-incompatibility reaction.

## Discussion

### Evaluation of the length of S-locus region in Maloideae species

The S-RNase-based GSI system has been extensively investigated in plants belonging to the Solanaceae, Plantaginaceae, and Rosaceae families (Hua et al. 2008). Recent studies utilizing PacBio long-read sequencing have revealed that two *Antirrhinum S*-haplotypes encompass approximately 1.2 Mb and contain 32 *SLF* genes (Zhu et al., 2023). Similarly, using a combination of BAC library and PCR-based approaches, it has been determined that the BAC contigs containing 17 *SLF*s cover roughly 3.1 Mb of the *Petunia S*_*2*_-locus (Wu et al. 2020). By comparing the *Petunia SLF*s to the *Solanum* genomes, it has been estimated that the *S*-locus region spans approximately 17.9 Mb in tomato (*Solanum* lycopersicum) and 14.6 Mb in potato (*S. tuberosum*; Kubo et al. 2015; Wu et al. 2020). In *Pyrus* species, 10 *SFBB*s were identified from the BAC contigs covering 378 kb of the* S*_*2*_-locus, while six *SFBB*s were identified from the BAC contigs covering 649 kb of the *S*_*4*_-locus (Okada et al. 2011). The number of *SFBB*s in *Pyrus S*-locus was found to be lower compared to *Petunia* and *Antirrhinum S*-loci, indicating that several *SFBB*s in *Pyrus S*-loci remained unidentified. In this study, we used long-read sequencing to assemble the genomes of pear cultivar ‘Yali’, and we successfully identified 19 *SFBB*s from *Pyrus S*_*17*_-locus located at the end of Chromosome 17 (Fig. 1). Furthermore, leveraging previously assembled genomes of *Pyrus* and *Malus S*-loci using long-read sequences (Daccord et al. 2017; Linsmith et al., 2019; Zhang et al. 2019; Dong et al. 2020; Sun et al. 2020; Gao et al. 2021), 17–21 *SFBB*s were identified from these reported *S*-loci (Fig. 2a; Table S4), which were distributed within a range of 1.35–2.64 Mb (Fig. 3c; Table S4). As a comparison, we also identified the *SLF*s/*SFB*s from *Prunus S*-locus (Figure S2), which were distributed within a range of 2.82–3.07 Mb (Table S4). These findings suggest that, on average, the *Pyrus* and *Malus S*-loci may have a smaller size compared to the *Prunus S*-locus.

### The pollen-specifically expressed SFBBs are good candidates for pollen-S determinant in pear

In the GSI mechanism, the pollen-S determinant in *Prunus* species of Rosaceae family is encoded by an *SLF*/*SFB* (Ushijima et al. 2003, 2004; Sonneveld et al. 2005). These *Prunus SLFs/SFB*s exhibit considerable sequence polymorphism among different S-haplotypes, are specifically expressed in pollen, and are tightly linked with the corresponding *S-RNase* (Ushijima et al. 2003; Wu et al. 2013). Conversely, the pollen-*S* determinant in Solanaceae and some Rosaceae species consists of multiple *SLF*s (Kubo et al., 2010; Kakui et al. 2011). These *SLF*s are also specifically expressed in pollen and are tightly linked with the corresponding *S-RNase*; however, the sequence polymorphism is relatively lower compared to the *Prunus SLFs/SFB*s (Kakui et al. 2011; Kubo et al. 2015). In this study, a total of 19 *SFBB*s were identified from the *Pyrus S*_*17*_-locus (Fig. 2a) and showed co-segregation with the *S*_*17*_*-RNase* in cross-pollinated progeny (Fig. 4a). Among these *SFBB*s, *PbrSFBB.Ia-S*_*17*_, *PbrSFBB.Ib-S*_*17*_, *PbrSFBB.II-S*_*17*_, *PbrSFBB.III-S*_*17*_, *PbrSFBB.IV-S*_*17*_, *PbrSFBB.VI-S*_*17*_, *PbrSFBB.VII-S*_*17*_, *PbrSFBB.VIII-S*_*17*_, *PbrSFBB.X-S*_*17*_, *PbrSFBB.XI-S*_*17*_, *PbrSFBB.XII-S*_*17*_, *PbrSFBB.XIII-S*_*17*_, *PbrSFBB.XIV-S*_*17*_, *PbrSFBB.XV-S*_*17*_, *PbrSFBB.XVI-S*_*17*_, *PbrSFBB.XVIII-S*_*17*_, *PbrSFBB.XIX-S*_*17*_, and *PbrSFBB.XX-S*_*17*_ were specifically expressed in pollen (Fig. 4b). The sequence polymorphism of each of these 18 *SFBB*s was also lower than that observed among *Prunus SLFs/SFB*s (Tables 1 and S6 to S8). Additionally, PbrSFBB.Ia-S_17_, PbrSFBB.Ib-S_17_, PbrSFBB.II-S_17_, PbrSFBB.VIII-S_17_, PbrSFBB.X-S_17_, PbrSFBB.XI-S_17_, PbrSFBB.XII-S_17_, and PbrSFBB.XVIII-S_17_ showed direct interaction with at least one non-self S-RNase, while the absence of interaction between the remaining 10 SFBBs and non-self S-RNases may be attributed to the limited number of non-self S-RNases used in this study (Fig. 5). Collectively, these findings indicate that these 18 *SFBB*s that are specifically expressed in pollen represent promising candidates for the pollen-S determinant in pear.

### The cross-recognition of SFBBs with non-self S-RNases

In the non-self recognition system, it has been observed that S-RNases cannot be recognized by any SLF present in the same *S*-haplotype, but they can be recognized by at least one of multiple SLFs present in different *S*-haplotypes (Sun et al. 2018). A similar mechanism is also proposed in *Pyrus* species (Kakui et al. 2011); however, direct evidence supporting this viewpoint is lacking. In this study, both Y2H and FLCI assays demonstrated that eight out of the 19 SFBBs in the *Pyrus S*_*17*_-locus are involved in recognizing non-self S-RNases (Fig. 5). This finding provides support for the viewpoint of the non-self recognition system in *Pyrus* species. It is important to note that PbrSFBB.VIII-S_17_ is the only SFBB recognizing *S*_*5*_- and *S*_*7*_-RNases but does not recognize *S*_*3*_-RNase (Fig. 5), despite the fact that there is a high amino sequence identity of 94.2% between *S*_*3*_- and *S*_*5*_-RNases (Table S15). If there are minor changes in the amino acid residues of *S*_*5*_-RNase, the mutant *S*_*5*_-RNase may not be recognized by PbrSFBB.VIII-S_17_, potentially leading to cross-incompatibility. This assumption is consistent with the non-self-recognition system, as the SLFs within an *S*-haplotype may not recognize approximately 5% of S-RNases in a population (Kubo et al. 2015).

Among the SFBBs that interact with at least one non-self S-RNase, PbrSFBB. VIII-S_17_ has the ability to recognize a majority of non-self S-RNases. On the other hand, PbrSFBB.Ia-S_17_, PbrSFBB.Ib-S_17_, PbrSFBB.II-S_17_, PbrSFBB.X-S_17_, PbrSFBB.XI-S_17_, PbrSFBB.XII-S_17_, and PbrSFBB.XVIII-S_17_ can only recognize a limited number of non-self S-RNases (Fig. 5). It should be noted that *SFBB.VIII* and *SFBB.XVIII* are present in both *Pyrus* and *Malus S*-loci, whereas deletion events occasionally occurs in the other *SFBB*s (Fig. 2a). It has been determined that these gene deletions may result from ectopic recombination between two LTR retrotransposons belonging to the same family and having the same orientation on the same chromosome (Kumar and Bennetzen 1999). Consequently, the presence of enriched LTR retrotransposons in the two flanking regions of these six *SFBB*s may be responsible for these deletions (Table S13; Shang et al. 2017). The absence of these *SFBB*s may lead to cross-incompatibility.

Furthermore, we observed deletions in most genes located between *SFBB.XVI* and *SFBB.VIII* (Table S5), with the exception of SFBB.III, SFBB.IV, SFBB.XIV and *SFBB.XVIII*. Additionally, frequent inversions and/or translocations were detected in this genomic region (Figs. 2a and S5 to S11). It has been determined that inversion may arise from unequal recombination events between two LTR retrotransposons with the opposite orientation on the same chromosome, while translocations may result from ectopic recombination between two LTR retrotransposons on different chromosomes (Kumar and Bennetzen 1999). Therefore, the presence of enriched LTR retrotransposons in *Pyrus* and *Malus S*-loci may be linked to the re-arrangement of *SFBB*s. This finding aligns with previous research indicating that transposable elements contribute to the reshaping of the *Petunia S*-locus (Wu et al. 2020).

In conclusion, based on the genomes assembled by long-read, we revealed the 17–21 *SFBB*s in *Pyrus* and *Malus S*-loci spanning a range of 1.35 to 2.64 Mb. These *SFBB*s could be classified into 22 groups, with 18 of them being specifically expressed in pollen. Notably, eight SFBBs demonstrated interactions with at least one non-self S-RNase, while the remaining SFBBs failed to recognize any S-RNase. These findings provide compelling evidence supporting the existence of a collaborative non-self-recognition system governing self-incompatibility in pear species.

## Materials and methods

### Plant materials

In accordance with a previous study on *S*-genotypes (Wang et al. 2017), a total of 10 pear cultivars, ‘Yali’ (*S*_*17*_*S*_*21*_), ‘Huanghua’ (*S*_*1*_*S*_*2*_), ‘Cuiguan’ (*S*_*3*_*S*_*5*_), ‘Dangshansuli’ (*S*_*7*_*S*_*17*_), ‘Xinxing’ (*S*_*4*_*S*_*7*_), ‘Mantianhong’ (*S*_*4*_*S*_*12*_), ‘Jinhua’ (*S*_*3*_*S*_*18*_), ‘Hongtaiyang’ (*S*_*8*_*S*_*35*_), and ‘Nanyue’ (*S*_*2*_*S*_*9*_), were selected for this investigation. These cultivars were maintained in an orchard located at Hushu, Nanjing, China. During the spring season, styles and anthers from these cultivars, as well as leaves from these cultivars were collected. The collected leaves and styles were promptly frozen in liquid nitrogen and then stored −80℃ until further use. The anthers, on the other hand, were enclosed in a dry container to facilitate the release of pollen grains. These pollen grains were then enclosed in sulfuric acid paper bags and stored in silica gel at −20℃ until needed.

Following the cross-pollination of ‘Yali’ and ‘Xueqing’ (*S*_*3*_*S*_*16*_), a total of 270 seeds were obtained and subjected to vernalization by placing them in moist sand at 4℃. The resulting seedlings were subsequently planted in an orchard located at Jiangpu, Nanjing, China. After eliminating pseudo-hybrid plants, 113 trees reached the flowering stage. Young leaves from these trees were collected during the spring season and stored −80℃ until analysis.

### DNA sequencing and genome assembly

The genomic DNA of ‘Yali’ was extracted from young leaves using the DNAsecure Plant Kit (Tiangen, Beijing, China) and subsequently fragmented. These fragments underwent size selection using AMPure PB Magnetic Beads (Pacific Biosciences), were repaired for any damages, and ligated to hairpin adapters. The resulting fragments were then used to construct a long-read sequencing library with an average insert size of 20 kb. This library was sequenced using the single-molecule real-time (SMRT) DNA sequencer on the PacBio sequel platform. Moreover, a short-read sequencing library was constructed using the Illumina TruSeq library construction kit (Illumina, San Diego, CA), and a Hi-C sequencing library was constructed using the standard protocol (Belton et al. 2012). Both libraries were sequenced on the Illumina Novaseq platform (Illumina).

To improve the quality of the PacBio reads, a self-correction step was perfomed by aligning all sequenced reads pairwise. Subsequently, these corrected reads were assembled into contigs using an overlap-layout-consensus algorithm and FALCON (https://github.com/PacificBiosciences/FALCON; Chin et al. 2016). The specific parameters used for the assembly were as follows: length_cutoff_pr = 10,000, max_diff = 95, and max_cov = 95. The resulting contigs were further refined through polishing steps using Quiver (Chi et al. 2013), which involved aligning the SMRT reads, as well as using pilon software (Walker et al. 2014) and aligning Illumina paired-end reads.

In order to evaluate the quality of the assembly, several assessments were conducted. Initially, sequence consistency was examined by aligning paired-end reads to the assembly using BWA software (http://bio-bwa.sourceforge.net/). The assembly of the ‘Yali’ genome had an average sequencing depth of 101.1-fold and a coverage of 99.83%. Additionally, over 97.5% of paired-end reads could be mapped to the assemblies. Furthermore, the completeness of the assembly was assessed using the BUSCO (http://busco.ezlab.org/).

### Genome annotation

Protein-coding genes were predicted with three different strategies. The first strategy involved d*e novo* prediction, which was conducted according to the previous reported method (Shang et al. 2020) without any modifications. For the second strategy, homolog-based prediction was employed using TBLASTN (Altschul et al. 1997), aligning the protein sequences of white pear (*Pyrus x bretschneideri*), apple (*Malus domestica*), sweet cherry (*Prunus avium*), Japanese apricot (*Prunus mume*), peach (*Prunus persica*), and oriental cherry (*Prunus yedoensis*) against the assembly. In addition, transcriptome sequencing was carried out on pollen grains, styles, leaves, and/or developing fruits. The transcriptome-based prediction and function annotation of protein-coding genes were performed following the previous reported method (Shang et al. 2020) without any modification. The coding sequencing of these protein-coding genes can be found in Files S1 and S2, and their annotations are provided in Files S3 and S4. Furthermore, non-coding RNAs and repeats were predicted using both de novo and homolog-based approaches, with the details mirroring those of the previous report (Shang et al. 2020). The annotations of non-coding RNAs and repeats are respectively listed in Tables S3 and S2, respectively.

### Identification and sequence analysis of Pyrus S-locus F-box genes

The nucleotide acid sequences of *S*_*17*_*-RNase* were utilized as a reference to determine the location of *Pyrus S*_*17*_-locus in the ‘Yali’ genome. It was observed that *S*_*17*_*-RNase* resided within the contig 33 in the ‘Yali’ genome. Next, the F-box motif's amino acid sequences (Figure S1) were subjected to BLAST against Chromosome 17 in the contig 33 in the ‘Yali’ genome. This search was performed using BioEdit software version 7.0.9.0 (http://www.mbio. ncsu.edu/bioedit/bioedit. html) with default parameters. Subsequently, the full-length sequences of *F-box* genes were manually predicted using Geneious Prime version 2021.1.1 (www.geneious.com). A similar approach was followed to predict the *F-box* genes in other *S*-loci of *Pyrus*, *Malus*, and *Prunus* species. The genomes used in this study were provided in Table S1.

To clone the *SFBB*s in the *Pyrus S*_*17*_-locus, first-strand cDNA from ‘Yali’ pollen were employed, while the rest were obtained from the National Center for Biotechnology Information database (https://www.ncbi.nlm.nih.gov/nucleotide/), as listed in Table S16. Sequence alignment was conducted using ClustalW, and sequence similarity was calculated using BioEdit software. A phylogenetic tree was constructed using MEGA software version 5.04 (www.megasoftware.net) with the neighbor-joining method. Bootstrap values were calculated from 1000 replicates and recorded for values exceeding 0.6. The resulting phylogenetic tree was color-coded using the iTOL tool (https://itol.embl.de/). To identify conserved residues of *SFBB*s, WebLogo tool (http://weblogo.berkeley.edu/logo.cgi) was utilized. Furthermore, gene duplication events were predicted using the MCScanX package (Wang et al. 2012).

### Yeast-two hybrid assay

Total RNAs were extracted from the styles of seven pear cultivars. The first-strand cDNA was synthesized and used as template for PCR amplification of *S-RNase* genes. A subset of nine S-RNases were randomly chosen for the Y2H assay. From ‘Huanghua’, *Pyrus S*_*1*_*-RNase* and *S*_*2*_*-RNase* were amplified. From ‘Cuiguan’, *S*_*3*_*-RNase* and *S*_*5*_*-RNase* were amplified. From ‘Dangshansuli’, ‘Nanyue’, ‘Mantianhong’, ‘Yali’, ‘Jinhua’, and ‘Hongtaiyang’, *S*_*7*_*-RNase*, *S*_*9*_*-RNase*, *S*_*12*_*-RNase*, *S*_*17*_*-RNase*, *S*_*18*_*-RNase*, and *S*_*35*_*-RNase* were amplified, respectively. The sequence coding signal peptide were removed from these *S-RNase*s, and the remaining coding sequences of these *S-RNase*s, including the HV region, were amplified from the styles of pear cultivars. The amplified product was ligated into the pGADT7 vector that was digested by EcoR I and BamH I enzymes (New England Biolabs, Illinois, USA), using the 2X MultiF Seamless Assembly Mix (ABclonal, Wuhan, China) that contains a recombinase. The full-length coding sequences of *SFBB*s in the *Pyrus S*_*17*_-locus were amplified from ‘Yali’ pollen. The amplified product was inserted into the pGBKT7 vector that was digested by EcoR I and Sal I enzymes (New England Biolabs), using the 2X MultiF Seamless Assembly Mix (ABclonal). The Y2H was performed in the yeast strain Y2HGold using the Matchmaker Gold Yeast Two-Hybrid System (Clontech, Palo Alto, CA). Moreover, to generate an S-RNase protein without the HV region, the coding sequences between the signal peptide and HV, and between HV and stop codon of the *PbS*_*5*_*-RNase* were successively inserted into the same one pGADT7 vector. The Y2H assay was conducted to assess the interaction between PbrSFBB.VIII-S_17_ and PbrS_5_-RNase without signal peptide and HV region. The primer sequences used for these experiments are provided in Table S17.

### Firefly Luciferase complementation imaging (FLCI) assay

The interaction between eight S-RNases and eight SFBBs were assessed using the FLCI assay. The full-length coding sequences of *Pyrus S*_*1*_*-*, *S*_*2*_*-*, *S*_*3*_*-*, *S*_*5*_*-*, *S*_*7*_*-*, *S*_*12*_*-*, *S*_*18*_*-*, and *S*_*35*_*-RNase* genes were individually inserted into the pCAMBIA1300-nLuc vector. Similarly, the full-length coding sequences of *PbrSFBB.Ia-S*_*17*_, *PbrSFBB.Ib-S*_*17*_, *PbrSFBB.II-S*_*17*_, *PbrSFBB.VIII-S*_*17*_, *PbrSFBB.X-S*_*17*_, *PbrSFBB.XI-S*_*17*_, *PbrSFBB.XII-S*_*17*_*,* and *PbrSFBB.XVIII-S*_*17*_ genes were individually inserted into the pCAMBIA1300-cLuc vector. These constructs were organized into 21 groups, each representing one of the 21 interaction pairs that were examined in the Y2H assay. The constructs in each group were transiently co-expressed in tobacco leaves after being introduced into *A. tumefaciens* strain GV3101. Luciferase activity was checked at 3 days after infiltration using a Luminescence & Fluorescence Imaging System (PIXIS 1024B/BUV, Teledyne Princeton Instruments, USA). The primer sequences used for this experiment are provided in Table S17.

### Subcellular localization

The full-length CDS of *PbSFBB.Ia-S*_*17*_, *PbSFBB.Ib-S*_*17*_, *PbSFBB.II-S*_*17*_, *PbSFBB.VIII-S*_*17*_, *PbSFBB.X-S*_*17*_, *PbSFBB.XI-S*_*17*_, *PbSFBB.XII-S*_*17*_*,* and *PbSFBB.XVIII-S*_*17*_ genes were individually inserted into a *GFP* vector to construct the CAMV-35S-drived fusion protein (Wu et al. 2023), PbSFBB.Ia-*S*_*17*_-GFP, PbSFBB.Ib-*S*_*17*_-GFP, PbSFBB.II-*S*_*17*_-GFP, PbSFBB.VIII-*S*_*17*_-GFP, PbSFBB.X-*S*_*17*_-GFP, PbSFBB.XI-*S*_*17*_-GFP, PbSFBB.XII-*S*_*17*_-GFP, and PbSFBB.XVIII-*S*_*17*_-GFP. Moreover, the full-length CDS of aquaporin PIP2A (At3g53420) were amplified from Arabidopsis and was inserted into a mCherry vector to construct the PIP2A::mCherry fusion protein as a plasma membrane control (Santiago et al*.*, 2013). The constructs were transferred into *A. tumefaciens* strain GV3101 and were then transiently co-expressed in tobacco epidermal cells. The protoplasts of tobacco epidermal cells were isolated according to the method described in a previous study (Panda et al., 2024). The fluorescence was observed using a confocal microscope LSM780 (Zelss, Germany). All the primer sequences are listed in Table S17.

## Supplementary Information

Supplementary Material 1: Table S1 Comparison of ‘Yali’ genome with previously published assemblies of *Pyrus* and *Malus* species. Table S2 Annotation of the repeats in ‘Yali’ genome. Table S3 Annotation of the non-coding RNAs in ‘Dananguo’ and 'Yali' genomes. Table S4 Identification of the* F-box* genes in *Pyrus*,* Malus* and *Prunus* S-loci. Table S5 Function annotation of the predicted genes in *S*-loci. Table S6 Sequence similarity (%) among *Pyrus* and *Malus SFBB* genes. Table S7 Sequence similarity (%) among *Prunus SFB* and *SLF* genes. Table S8 Sequence similarity among *Prunus SFB* and *SLF* genes. Table S9 Sequence similarity (%) among *Pyrus* and *Malus S-RNase* genes. Table S10 Prediction of gene duplication events of *Pyrus* and *Malus SFBB *genes. Table S11 Sequence similarity of the non-coding flanking sequences of *SFBB*s in *Pyrus* and *Malus S*-loci. Table S12 Analysis of number and length of LTR retrotransposon in different *S*-loci. Table S13 Identification of the LTR retrotransposon in different *S*-loci. Table S14 RPKM values of the genes commonly existed in the tested *S*-loci. Table S15 Sequence similarity (%) among the reported *Pyrus S-RNase* genes. Table S16 The accession numbers of *S-RNase* and *S*-locus* F-box* genes in *Pyrus*, *Malus*, and *Prunus*.Table S17 Primers used in this study. Figure S1 Isolation of the conserved F-box motif in the reported *S*-locus F-box proteins in *Pyrus* and *Malus*. The accession numbers of these F-box proteins were listed in Table S13. Figure S2 Phylogenetic classifications of *S*-locus *F-box* genes in *Prunus*. The S-locus F-box (SLF/SFB) proteins in Prunus comprised by 12 groups, SLF1→SLF11 and SFB. Each group were highlighted with different colors. Figure S3 Phylogenetic analysis of the *F-box* genes identified from this and previous studies. Cycles with black color present the F-box genes identified from previous study (Huang et al., 2023). The rates (%) of different types of gene duplication events (dispersed, proximal, tandem and transposed) of the *S*-locus *F-box* genes were calculated for three *Pyrus* (PbrS17, PcS101, and PpyS5) and three *Malus* (MdS2, MdS3, MdS5, and MdS9) *S*-loci. The details of these duplication events are listed in Table S10. Figure S4 Gene duplication events of *Pyrus* and *Malus SFBB*s in whole genome. Figures S5 and S6 Synteny analysis of *SFBB* genes in *Pyrus* and *Malus S*-loci. Arrowhead present the transcriptional direction of a gene. The arrowheads with black color present *S-RNase* gene, while the arrowheads with other colors present *S*-locus*F-box* genes. The characters above arrowhead are the classification of *S*-locus*F-box* genes in *Pyrus*, *Malus*, and *Prunus* species. The Arabic numerals below the dotted line are the physical distance (Kb) between two adjacent *F-box* genes. The Chromosome location of these *F-box* genes were listed in Table S4. Figures S7 to S11 Synteny analysis of *SFBB* genes in* Malus S*-loci and *Pyrus* S_*17*_-, *S*_*101*_-, *S*_*67*_-,*S*_*7*_-, and *Pyrus S*_*5*_-loci. Arrowhead present the transcriptional direction of a gene. The arrowheads with black color present *S-RNase* gene, while the arrowheads with other colors present *S*-locus*F-box* genes. The characters above arrowhead are the classification of *S*-locus*F-box* genes in *Pyrus*, *Malus*, and *Prunus* species. The Arabic numerals below the dotted line are the physical distance (Kb) between two adjacent *F-box* genes. The Chromosome location of these *F-box* genes were listed in Table S4. Figures S12 to S14 Comparison analysis of the 5 kb non-coding flanking sequences of *SFBB*s in *Pyrus S*_*17*_-, *S*_*5*_-,* S*_*67*_-, and *S*_*101*_-loci. Figures S15 to S19 Comparison analysis of the 5 kb non-coding flanking sequences of *SFBB*s in *Malus S*_*2*_-, *S*_*3*_-, *S*_*5*_-, and *S*_*9*_-loci. Figures S20 to S40 Comparison analysis of the 5 kb non-coding flanking sequences of *SFBB*s in groups Ia, Ib, II to VIII, X to XIX, XXI, and XXII. (a) A snapshot showing the alignment of the 5kb upstream sequences of SFBBs. (b) Pairwise identity of the 5kb upstream sequences of SFBBs. (c) A snapshot showing the alignment of the 5kb downstream sequences of SFBBs. (d) Pairwise identity of the 5kb downstream sequences of SFBBs. Figure S41 Identification of *S*-genotypes of the individuals in the cross-pollinated progeny of ‘Yali’ × ‘Xueqing’. The lanes 1-42 represent the individuals. Q and Y represent the pear cultivars ‘Xueqing’ and ‘Yali’, respectively. M indicates the DNA ladder. Figures S42 to S60:The amino acid sequences of *PbrSFBB.Ia-S*_*17*_, *PbrSFBB.Ib-S*_*17*_, *PbrSFBB.II-S*_*17*_, *PbrSFBB.III-S*_*17*_, *PbrSFBB.IV-S*_*17*_,* PbrSFBB.VI-S*_*17*_, *PbrSFBB.VII-S*_*17*_, *PbrSFBB.VIII-S*_*17*_, *PbrSFBB.X-S*_*17*_, *PbrSFBB.XI-S*_*17*_, *PbrSFBB.XII-S*_*17*_, *PbrSFBB.XIII-S*_*17*_, *PbrSFBB.XIV-S*_*17*_, *PbrSFBB.XV-S*_*17*_, *PbrSFBB.XVI-S*_*17*_, *PbrSFBB.XVIII-S*_*17*_, *PbrSFBB.XIX-S*_*17*_, *PbrSFBB.XX-S*_*17*_, and *PbrSFBB.XXII-S*_*17*_ in any individual including *S*_*17*_-RNase were identical to that in ‘Yali’. YX-1, 2, 4, 5, 9, 10, 14, 19, 20, 23, 24, 28, 29, 30, 32, 34, 35, 36, 37, 38, 41, and 42 are the individuals of the cross-pollinated progeny of ‘Yali’ × ‘Xueqing’. Figure S61 Expression analysis of *S-RNase* and *SFBB* genes in *Pyrus S*_*17*_-locus in different tissues. Figure S62 Self-activation of S-RNase and SFBB proteins in *Pyrus S*_*17*_-locus in yeast cells. AD and BD present the pGADT7 and pGBKT7 vectors respectively. Positive and negative controls were assigned as P53/T-antigen and Lamin/T-antigen, respectively. SD/-Trp/-Leu indicates the SD medium lacking Trp and Leu; SD/-Trp/-Leu/-His/-Ade indicates the SD medium lacking Trp, Leu, His, and Ade; SD/-Trp/-Leu-His/-Ade + X-α-gal indicates that x-a-gal was mixed into the SD medium lacking Trp, Leu, His, and Ade. Files S1 The coding sequences of all predicted genes in ‘Yali’ genome. Files S2 Gene annotation of‘Yali’ genome.

## Data Availability

Data supporting the findings of this work are available within the paper and its Supplementary Information files. The datasets are available from the corresponding author upon request. Raw genome sequencing reads and the assembly of ‘Yali’ genome are respectively deposited into the NCBI sequence read archive (SRA) with the BioProject number PRJNA852504. Sequence data of 18 *S*_*17*_*-locus F-box brother* genes are deposited into the GenBank with the accession numbers ON918613-ON918630.

## Abbreviations

SFBB: S-locus F-box brother
BUSCO: Benchmarking universal single-copy orthologs
SLF: S-locus F-box
SFB: S-locus haplotype F-box
Y2H: Yeast two-hybrid
FLCI: Firefly luciferase complementation imaging
GFP: Green fluorescent protein
QRT-PCR: Quantitative real-time PCR
RPKM: Reads per kilobase per million
SI: Self-incompatibility
