# Supplementary material for: Long-read genome sequencing reveals the sequence characteristics of pear self-incompatibility locus
Source: Mol Hortic. 2025 Mar 1;5:13. doi: 10.1186/s43897-024-00132-0 (PMC11871771; doi:10.1186/s43897-024-00132-0)
Supplement: Supplementary file 1 — Supplementary Material 1: Table S1 Comparison of ‘Yali’ genome with previously published assemblies of Pyrus and Malus species. Table S2 Annotation of the repeats in ‘Yali’ genome. Table S3 Annotation of the non-coding RNAs in ‘Dananguo’ and 'Yali' genomes. Table S4 Identification of the F-box genes in Pyrus, Malus and Prunus S-loci. Table S5 Function annotation of the predicted genes in S-loci. Table S6 Sequence similarity (%) among Pyrus and Malus SFBB genes. Table S7 Sequence similarity (%) among Prunus SFB and SLF genes. Table S8 Sequence similarity among Prunus SFB and SLF genes. Table S9 Sequence similarity (%) among Pyrus and Malus S-RNase genes. Table S10 Prediction of gene duplication events of Pyrus and Malus SFBB genes. Table S11 Sequence similarity of the non-coding flanking sequences of SFBBs in Pyrus and Malus S-loci. Table S12 Analysis of number and length of LTR retrotransposon in different S-loci. Table S13 Identification of the LTR retrotransposon in different S-loci. Table S14 RPKM values of the genes commonly existed in the tested S-loci. Table S15 Sequence similarity (%) among the reported Pyrus S-RNase genes. Table S16 The accession numbers of S-RNase and S-locus F-box genes in Pyrus, Malus, and Prunus.Table S17 Primers used in this study. Figure S1 Isolation of the conserved F-box motif in the reported S-locus F-box proteins in Pyrus and Malus. The accession numbers of these F-box proteins were listed in Table S13. Figure S2 Phylogenetic classifications of S-locus F-box genes in Prunus. The S-locus F-box (SLF/SFB) proteins in Prunus comprised by 12 groups, SLF1→SLF11 and SFB. Each group were highlighted with different colors. Figure S3 Phylogenetic analysis of the F-box genes identified from this and previous studies. Cycles with black color present the F-box genes identified from previous study (Huang et al., 2023). The rates (%) of different types of gene duplication events (dispersed, proximal, tandem and transposed) of the S-locus F-box ge [file 43897_2024_132_MOESM1_ESM.zip › Supplementary Figures S1 to S11.docx]

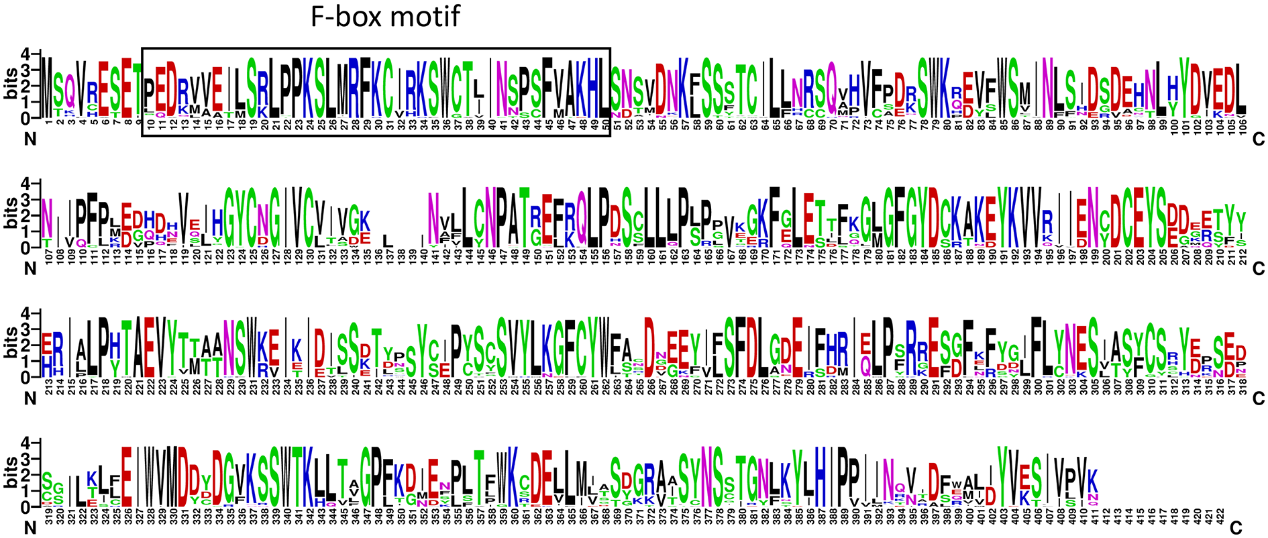


**Figure S1** Isolation of the conserved F-box motif in the reported *S*-locus F-box proteins in *Pyrus* and *Malus*. The accession numbers of these F-box proteins were listed in Table S13.


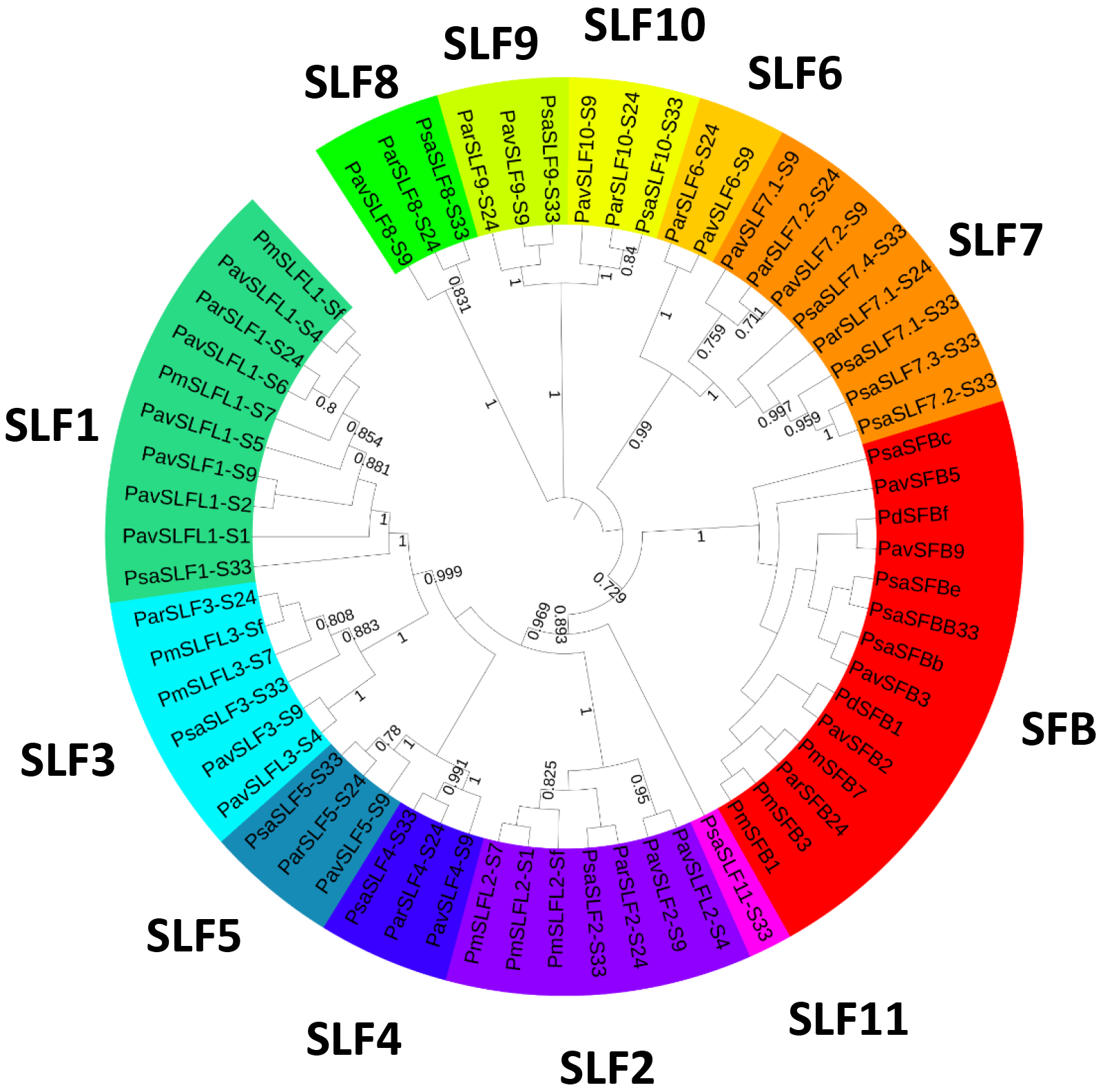


**Figure S2** Phylogenetic classifications of *S*-locus *F-box* genes in *Prunus*. The *S*-locus F-box (SLF/SFB) proteins in *Prunus* comprised by 12 groups, SLF1→SLF11 and SFB. Each group were highlighted with different colors.





**Figure S3** Phylogenetic analysis of the *F-box* genes isolated from this and previous studies. Cycles with black color present the *F-box* genes isolated from previous study (Huang et al., 2023).


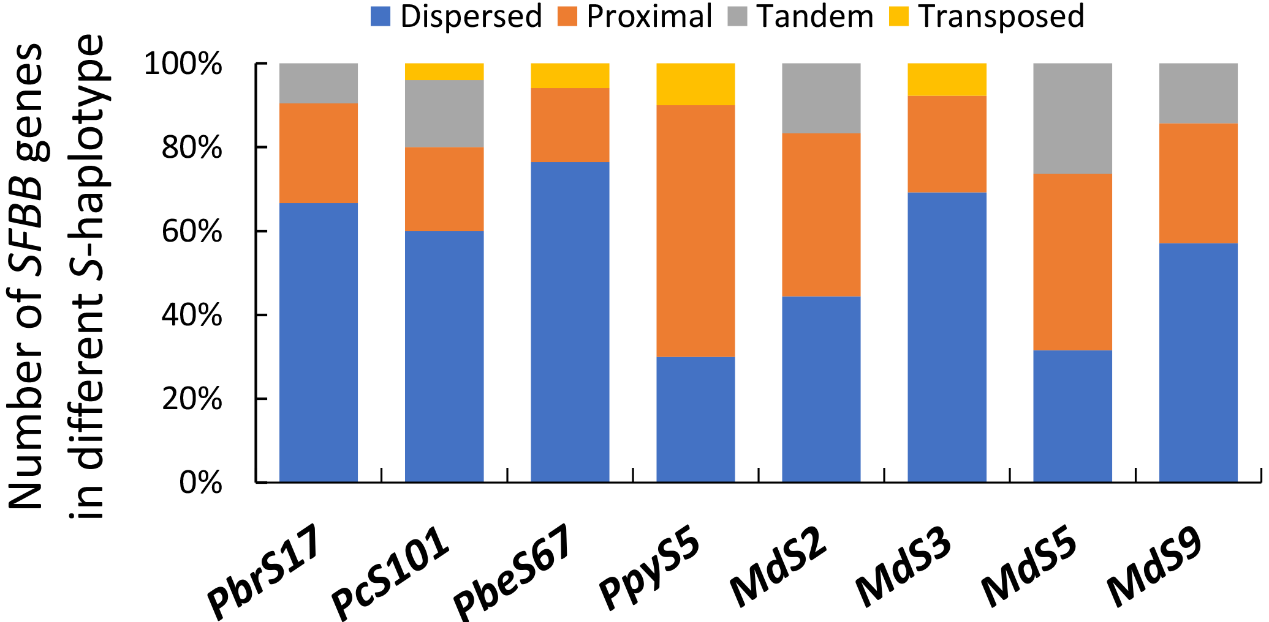


**Figure S4** Gene duplication events of *Pyrus* and *Malus SFBB*s in whole genome. The rates (%) of different types of gene duplication events (dispersed, proximal, tandem and transposed) of the *S*-locus *F-box* genes were calculated for three *Pyrus* (PbrS17, PcS101, and PpyS5) and three *Malus* (MdS2, MdS3, MdS5, and MdS9) *S*-loci. The details of these duplication events are listed in Table S10.


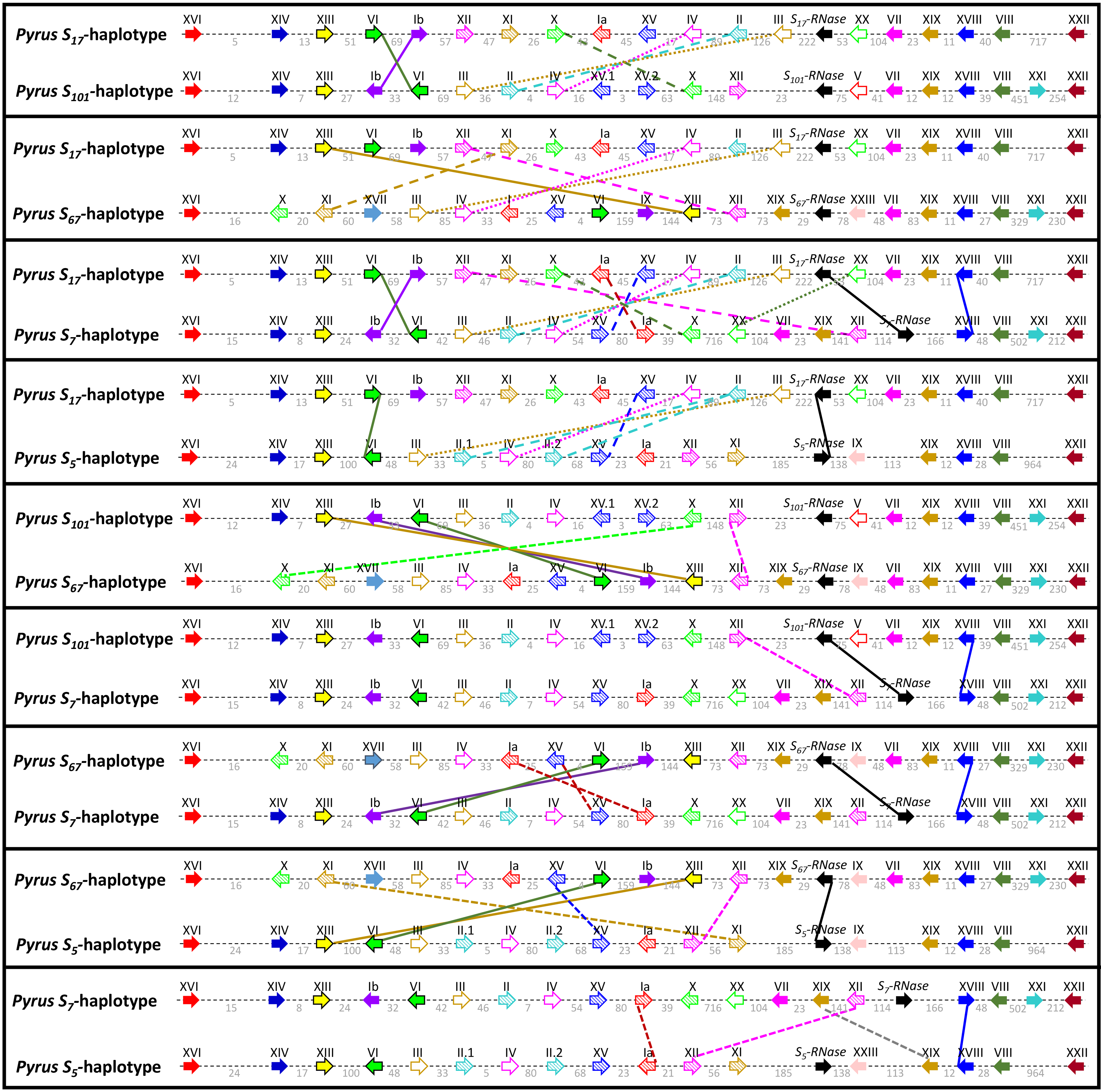


**Figure S5** Synteny analysis of *SFBB* genes in *Pyrus S*-loci. The genes clustered in a group were marked by different symbols, respectively. Arrowhead present the transcriptional direction of a gene. The arrowheads with black color present *S-RNase* gene, while the arrowheads with other colors present *S*-locus *F-box* genes. The characters above arrowhead are the classification of *S*-locus *F-box* genes in *Pyrus*, *Malus*, and *Prunus* species. The Arabic numerals below the dotted line are the physical distance (Kb) between two adjacent *F-box* genes. The Chromosome location of these *F-box* genes were listed in Table S4.


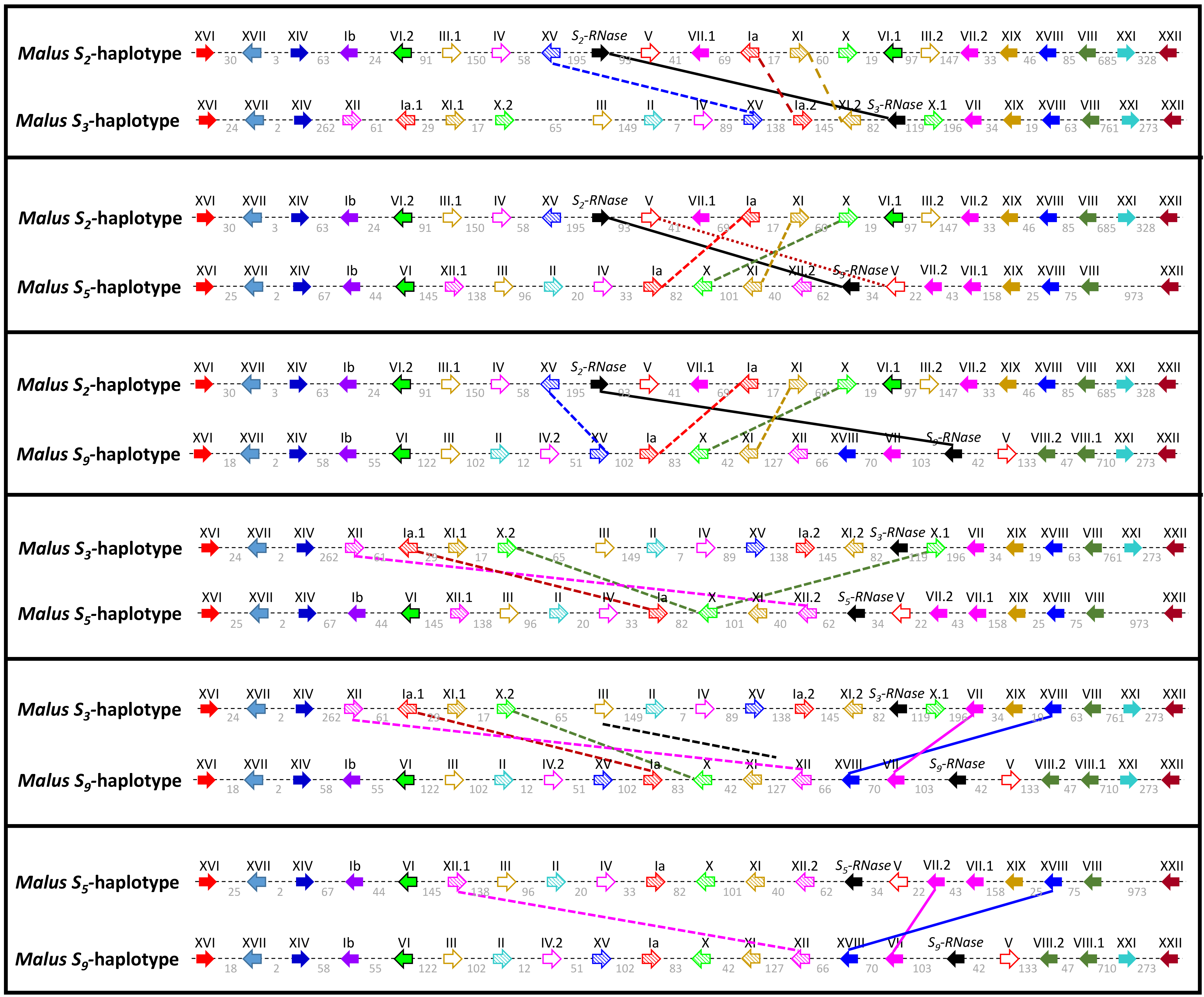


**Figure S6** Synteny analysis of *SFBB* genes in *Malus S*-loci. The genes clustered in a group were marked by different symbols, respectively. Arrowhead present the transcriptional direction of a gene. The arrowheads with black color present *S-RNase* gene, while the arrowheads with other colors present *S*-locus *F-box* genes. The characters above arrowhead are the classification of *S*-locus *F-box* genes in *Pyrus*, *Malus*, and *Prunus* species. The Arabic numerals below the dotted line are the physical distance (Kb) between two adjacent *F-box* genes. The Chromosome location of these *F-box* genes were listed in Table S4.


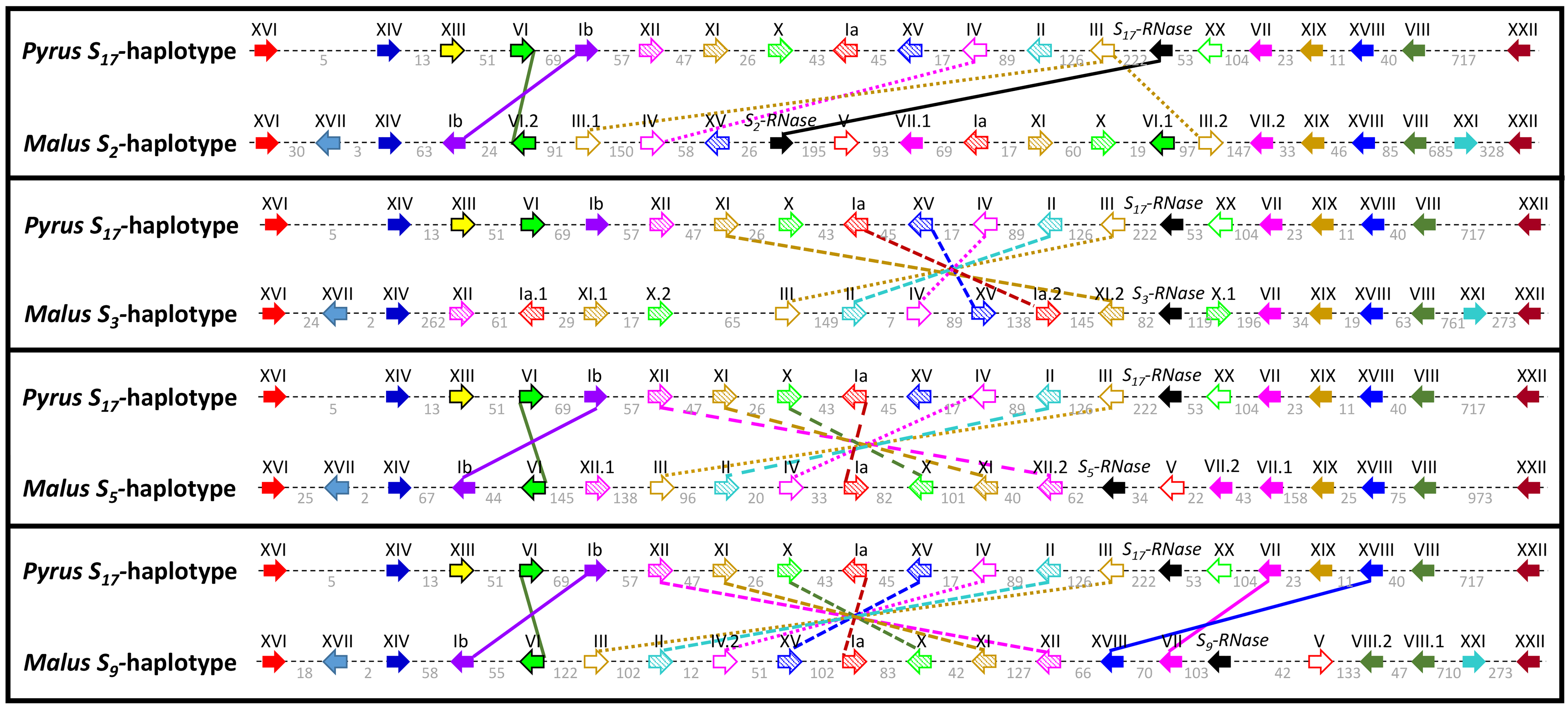


**Figure S7** Synteny analysis of *SFBB* genes in *Pyrus S_17_*-locus and *Malus S*-loci. The genes clustered in a group were marked by different symbols, respectively. Arrowhead present the transcriptional direction of a gene. The arrowheads with black color present *S-RNase* gene, while the arrowheads with other colors present *S*-locus *F-box* genes. The characters above arrowhead are the classification of *S*-locus *F-box* genes in *Pyrus*, *Malus*, and *Prunus* species. The Arabic numerals below the dotted line are the physical distance (Kb) between two adjacent *F-box* genes. The Chromosome location of these *F-box* genes were listed in Table S4.


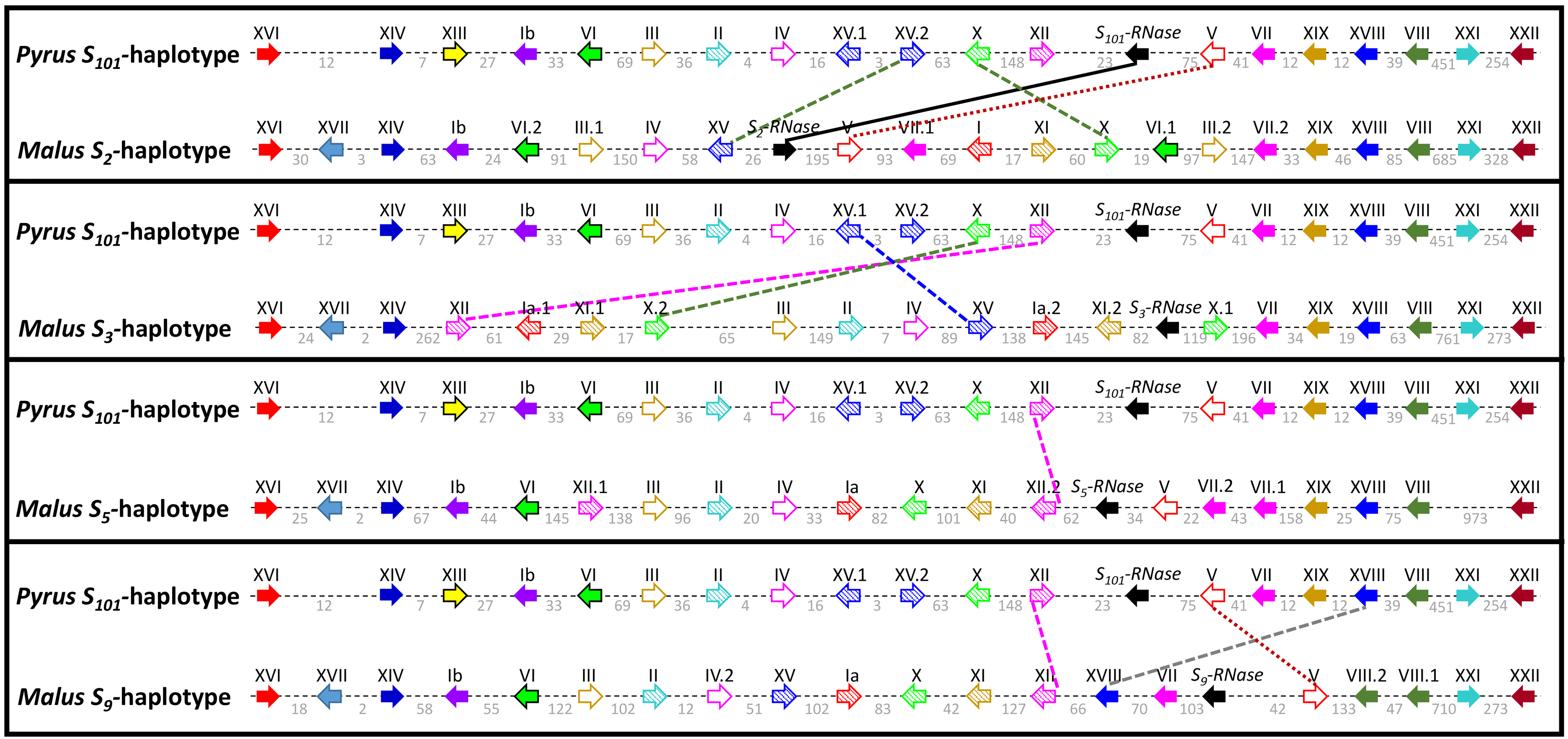


**Figure S8** Synteny analysis of *SFBB* genes in *Pyrus S_101_*-locus and *Malus S*-loci. The genes clustered in a group were marked by different symbols, respectively. Arrowhead present the transcriptional direction of a gene. The arrowheads with black color present *S-RNase* gene, while the arrowheads with other colors present *S*-locus *F-box* genes. The characters above arrowhead are the classification of *S*-locus *F-box* genes in *Pyrus*, *Malus*, and *Prunus* species. The Arabic numerals below the dotted line are the physical distance (Kb) between two adjacent *F-box* genes. The Chromosome location of these *F-box* genes were listed in Table S4.


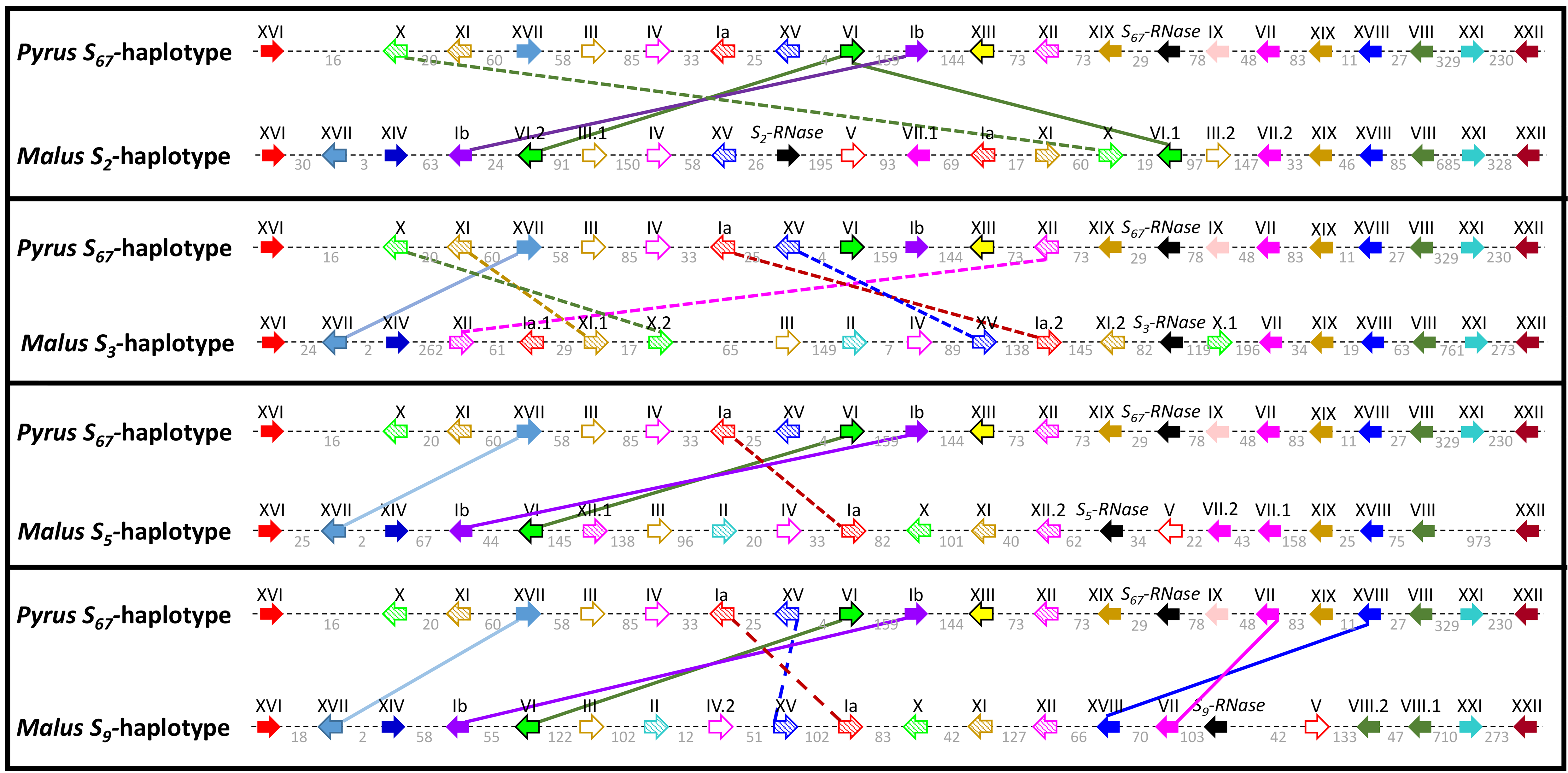


**Figure S9** Synteny analysis of *SFBB* genes in *Pyrus S_67_*-locus and *Malus S*-loci. The genes clustered in a group were marked by different symbols, respectively. Arrowhead present the transcriptional direction of a gene. The arrowheads with black color present *S-RNase* gene, while the arrowheads with other colors present *S*-locus *F-box* genes. The characters above arrowhead are the classification of *S*-locus *F-box* genes in *Pyrus*, *Malus*, and *Prunus* species. The Arabic numerals below the dotted line are the physical distance (Kb) between two adjacent *F-box* genes. The Chromosome location of these *F-box* genes were listed in Table S4.


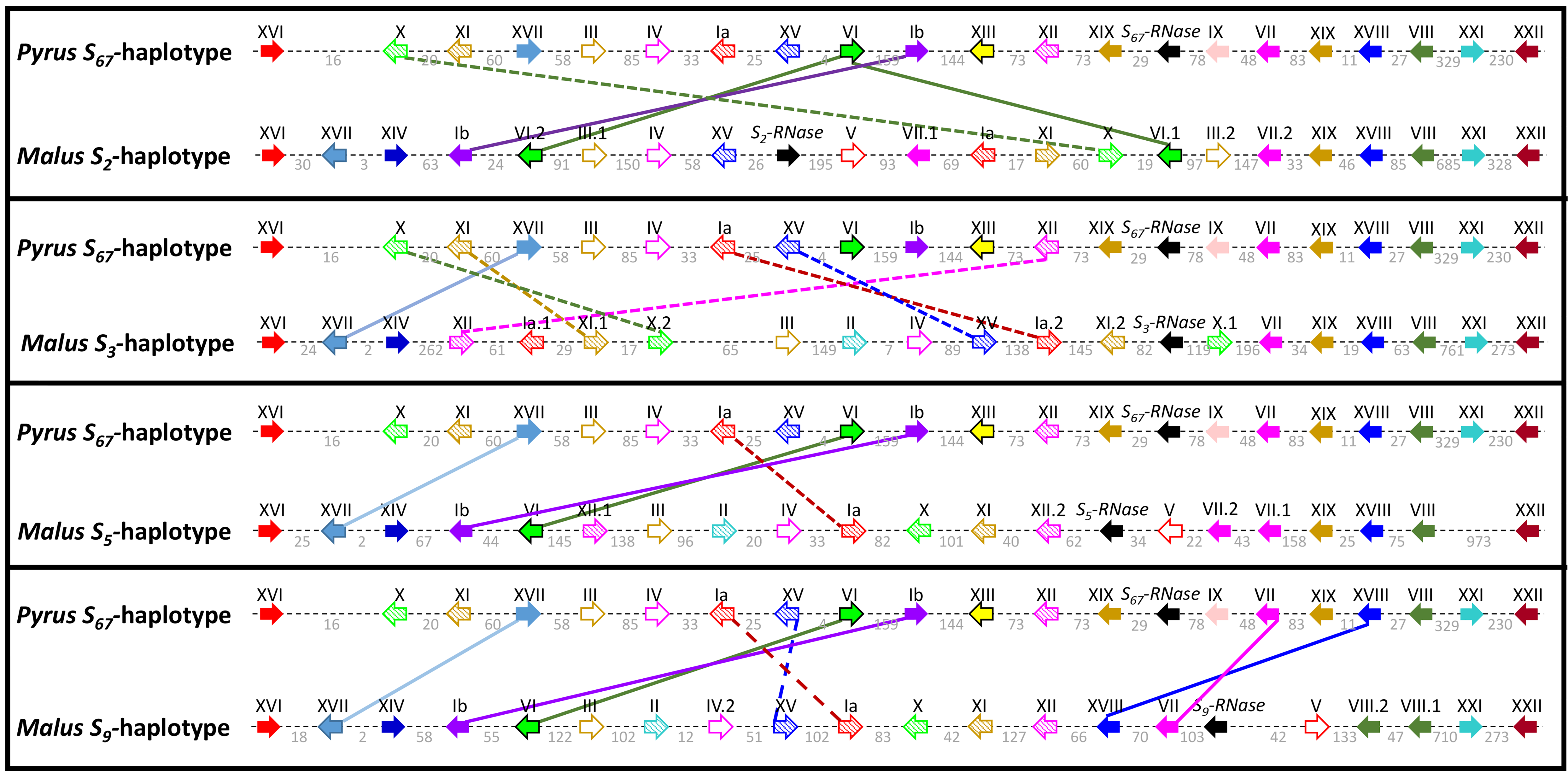


**Figure S10** Synteny analysis of *SFBB* genes in *Pyrus S_7_*-locus and *Malus S*-loci. The genes clustered in a group were marked by different symbols, respectively. Arrowhead present the transcriptional direction of a gene. The arrowheads with black color present *S-RNase* gene, while the arrowheads with other colors present *S*-locus *F-box* genes. The characters above arrowhead are the classification of *S*-locus *F-box* genes in *Pyrus*, *Malus*, and *Prunus* species. The Arabic numerals below the dotted line are the physical distance (Kb) between two adjacent *F-box* genes. The Chromosome location of these *F-box* genes were listed in Table S4.


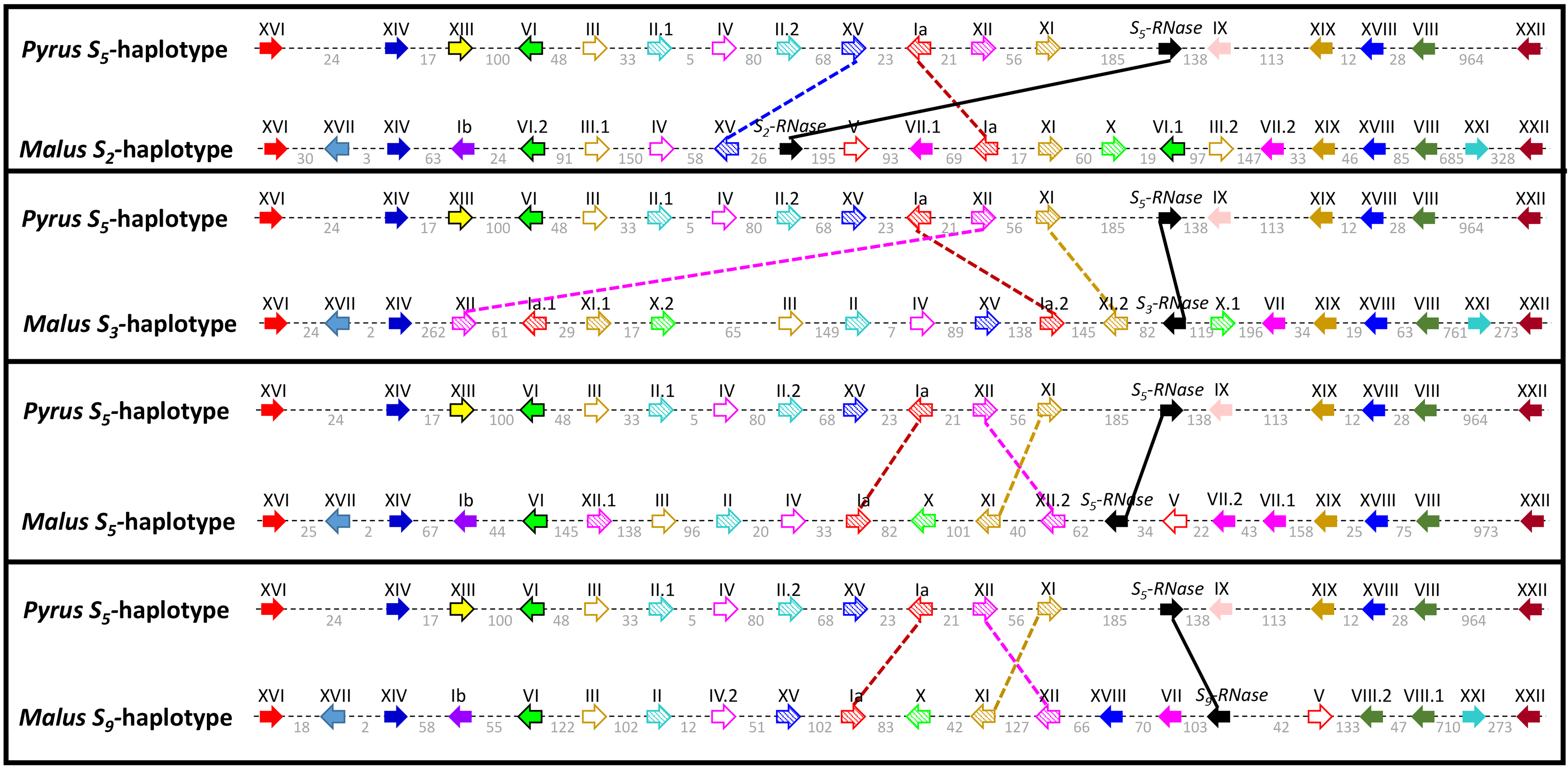


**Figure S11** Synteny analysis of *SFBB* genes in *Pyrus S_5_*-locus and *Malus S*-loci. The genes clustered in a group were marked by different symbols, respectively. Arrowhead present the transcriptional direction of a gene. The arrowheads with black color present *S-RNase* gene, while the arrowheads with other colors present *S*-locus *F-box* genes. The characters above arrowhead are the classification of *S*-locus *F-box* genes in *Pyrus*, *Malus*, and *Prunus* species. The Arabic numerals below the dotted line are the physical distance (Kb) between two adjacent *F-box* genes. The Chromosome location of these *F-box* genes were listed in Table S4.
