# Supplementary material for: Long-read genome sequencing reveals the sequence characteristics of pear self-incompatibility locus
Source: Mol Hortic. 2025 Mar 1;5:13. doi: 10.1186/s43897-024-00132-0 (PMC11871771; doi:10.1186/s43897-024-00132-0)
Supplement: Supplementary file 1 — Supplementary Material 1: Table S1 Comparison of ‘Yali’ genome with previously published assemblies of Pyrus and Malus species. Table S2 Annotation of the repeats in ‘Yali’ genome. Table S3 Annotation of the non-coding RNAs in ‘Dananguo’ and 'Yali' genomes. Table S4 Identification of the F-box genes in Pyrus, Malus and Prunus S-loci. Table S5 Function annotation of the predicted genes in S-loci. Table S6 Sequence similarity (%) among Pyrus and Malus SFBB genes. Table S7 Sequence similarity (%) among Prunus SFB and SLF genes. Table S8 Sequence similarity among Prunus SFB and SLF genes. Table S9 Sequence similarity (%) among Pyrus and Malus S-RNase genes. Table S10 Prediction of gene duplication events of Pyrus and Malus SFBB genes. Table S11 Sequence similarity of the non-coding flanking sequences of SFBBs in Pyrus and Malus S-loci. Table S12 Analysis of number and length of LTR retrotransposon in different S-loci. Table S13 Identification of the LTR retrotransposon in different S-loci. Table S14 RPKM values of the genes commonly existed in the tested S-loci. Table S15 Sequence similarity (%) among the reported Pyrus S-RNase genes. Table S16 The accession numbers of S-RNase and S-locus F-box genes in Pyrus, Malus, and Prunus.Table S17 Primers used in this study. Figure S1 Isolation of the conserved F-box motif in the reported S-locus F-box proteins in Pyrus and Malus. The accession numbers of these F-box proteins were listed in Table S13. Figure S2 Phylogenetic classifications of S-locus F-box genes in Prunus. The S-locus F-box (SLF/SFB) proteins in Prunus comprised by 12 groups, SLF1→SLF11 and SFB. Each group were highlighted with different colors. Figure S3 Phylogenetic analysis of the F-box genes identified from this and previous studies. Cycles with black color present the F-box genes identified from previous study (Huang et al., 2023). The rates (%) of different types of gene duplication events (dispersed, proximal, tandem and transposed) of the S-locus F-box ge [file 43897_2024_132_MOESM1_ESM.zip › Supplementary Figures S12 to S15.pdf]

(a)

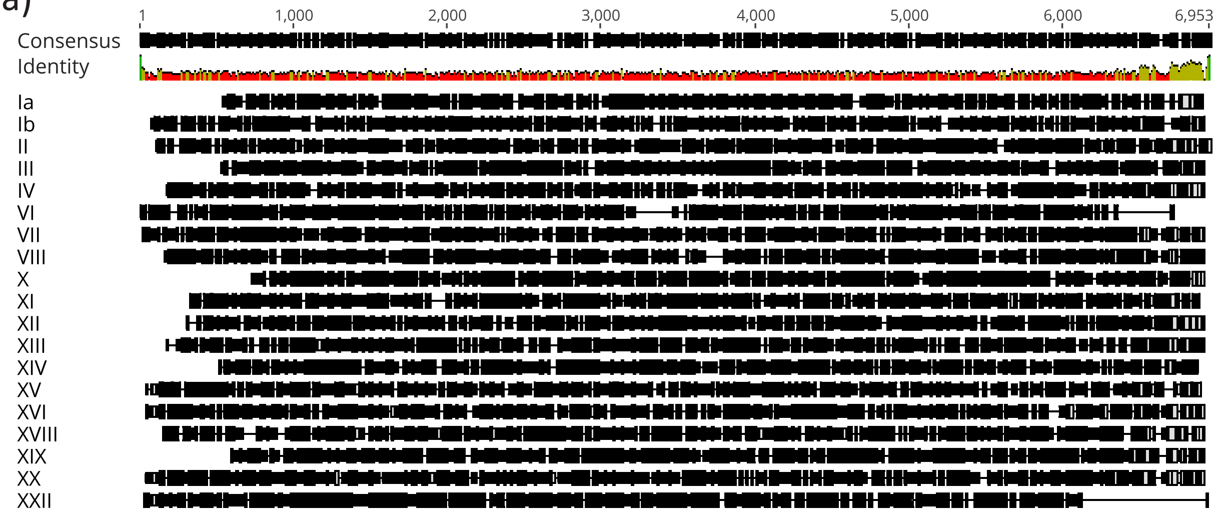

(b)

	Ia	Ib	II	III	IV	VI	VII	VIII	X	XI	XII	XIII	XIV	XV	XVI	XVIII	XIX	XX	XXII
Ia		32.78%	24.01%	25.70%	29.90%	23.01%	23.78%	23.03%	25.89%	24.77%	31.95%	23.27%	24.47%	31.81%	23.20%	22.43%	25.08%	22.78%	23.24%
Ib	32.78%		23.29%	24.07%	34.71%	22.33%	24.22%	22.60%	27.46%	24.63%	32.22%	23.56%	24.79%	41.42%	23.78%	23.19%	24.94%	23.03%	23.37%
II	24.01%	23.29%		29.06%	24.57%	22.78%	34.09%	33.15%	26.88%	29.99%	24.08%	30.56%	29.61%	23.98%	32.54%	31.13%	29.78%	33.16%	23.17%
III	25.70%	24.07%	29.06%		23.34%	23.63%	30.97%	28.92%	27.60%	28.82%	25.05%	28.05%	30.94%	24.31%	30.68%	28.96%	30.63%	29.28%	24.03%
IV	29.90%	34.71%	24.57%	23.34%		21.92%	23.93%	24.46%	25.54%	24.70%	32.98%	24.27%	24.58%	35.62%	23.71%	24.22%	25.11%	23.69%	22.18%
VI	23.01%	22.33%	22.78%	23.63%	21.92%		23.53%	22.62%	23.42%	22.23%	20.81%	22.74%	23.74%	21.31%	21.97%	23.27%	23.00%	23.50%	25.80%
VII	23.78%	24.22%	34.09%	30.97%	23.93%	23.53%		35.17%	26.04%	29.78%	24.50%	30.23%	29.69%	23.89%	40.44%	31.99%	31.42%	43.59%	24.13%
VIII	23.03%	22.60%	33.15%	28.92%	24.46%	22.62%	35.17%		26.02%	30.38%	25.03%	29.82%	29.50%	22.97%	35.15%	32.35%	29.40%	33.95%	22.10%
X	25.89%	27.46%	26.88%	27.60%	25.54%	23.42%	26.04%	26.02%		27.03%	25.80%	27.50%	26.09%	25.32%	27.25%	26.12%	27.85%	26.29%	24.51%
XI	24.77%	24.63%	29.99%	28.82%	24.70%	22.23%	29.78%	30.38%	27.03%		25.70%	31.40%	30.12%	25.14%	29.49%	29.82%	29.67%	29.82%	23.26%
XII	31.95%	32.22%	24.08%	25.05%	32.98%	20.81%	24.50%	25.03%	25.80%	25.70%		24.90%	26.25%	32.69%	25.36%	23.65%	25.65%	23.39%	23.25%
XIII	23.27%	23.56%	30.56%	28.05%	24.27%	22.74%	30.23%	29.82%	27.50%	31.40%	24.90%		29.58%	23.65%	29.33%	29.56%	28.66%	29.94%	23.29%
XIV	24.47%	24.79%	29.61%	30.94%	24.58%	23.74%	29.69%	29.50%	26.09%	30.12%	26.25%	29.58%		25.21%	30.37%	28.97%	29.64%	28.85%	25.59%
XV	31.81%	41.42%	23.98%	24.31%	35.62%	21.31%	23.89%	22.97%	25.32%	25.14%	32.69%	23.65%	25.21%		23.80%	22.61%	24.79%	23.28%	23.25%
XVI	23.20%	23.78%	32.54%	30.68%	23.71%	21.97%	40.44%	35.15%	27.25%	29.49%	25.36%	29.33%	30.37%	23.80%		31.48%	29.31%	38.03%	23.17%
XVIII	22.43%	23.19%	31.13%	28.96%	24.22%	23.27%	31.99%	32.35%	26.12%	29.82%	23.65%	29.56%	28.97%	22.61%	31.48%		29.08%	30.91%	23.03%
XIX	25.08%	24.94%	29.78%	30.63%	25.11%	23.00%	31.42%	29.40%	27.85%	29.67%	25.65%	28.66%	29.64%	24.79%	29.31%	29.08%		29.51%	24.30%
XX	22.78%	23.03%	33.16%	29.28%	23.69%	23.50%	43.59%	33.95%	26.29%	29.82%	23.39%	29.94%	28.85%	23.28%	38.03%	30.91%	29.51%		22.70%
XXII	23.24%	23.37%	23.17%	24.03%	22.18%	25.80%	24.13%	22.10%	24.51%	23.26%	23.25%	23.29%	25.59%	23.25%	23.17%	23.03%	24.30%	22.70%	

(c)

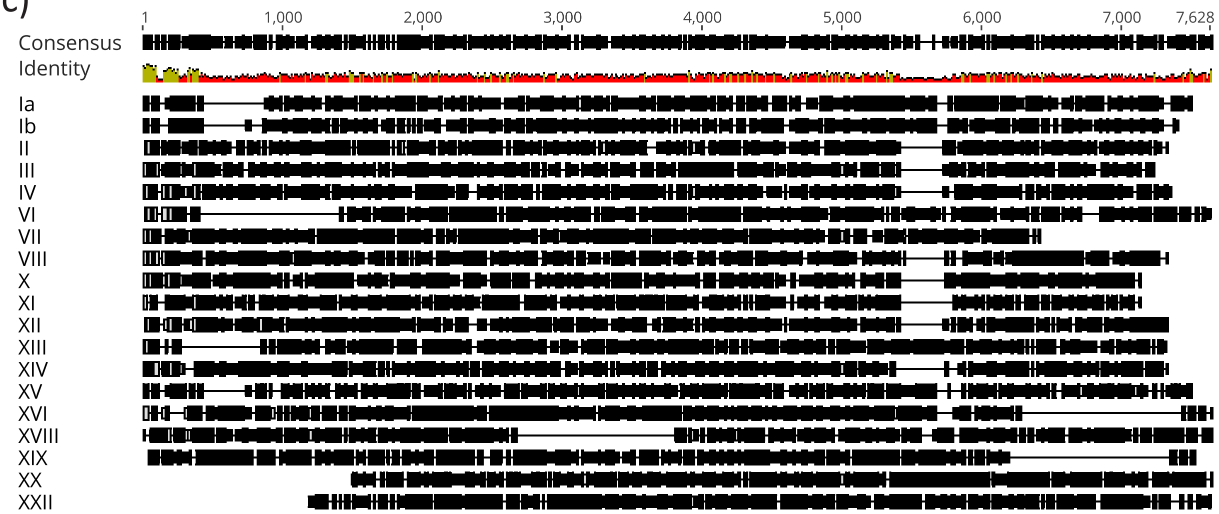

(d)

	Ia	Ib	II	III	IV	VI	VII	VIII	X	XI	XII	XIII	XIV	XV	XVI	XVIII	XIX	XX	XXII
Ia		37.00%	21.12%	21.97%	21.07%	21.56%	21.96%	21.04%	19.03%	20.87%	20.62%	30.38%	20.88%	41.51%	18.54%	17.47%	18.76%	23.20%	23.20%
Ib	37.00%		21.42%	20.84%	20.58%	21.07%	22.30%	20.51%	19.74%	21.27%	20.06%	30.11%	19.49%	36.96%	18.30%	18.03%	18.08%	21.91%	22.06%
II	21.12%	21.42%		31.08%	29.61%	23.73%	27.96%	29.53%	29.46%	29.68%	29.27%	20.14%	29.64%	20.73%	23.85%	22.88%	24.41%	24.00%	24.75%
III	21.97%	20.84%	31.08%		33.10%	23.81%	28.89%	34.05%	32.62%	30.61%	32.34%	21.35%	52.87%	21.58%	23.89%	24.52%	24.19%	25.95%	26.26%
IV	21.07%	20.58%	29.61%	33.10%		22.60%	28.22%	31.84%	32.49%	31.20%	41.87%	19.71%	32.75%	20.65%	23.82%	22.93%	24.08%	24.06%	24.99%
VI	21.56%	21.07%	23.73%	23.81%	22.60%		25.22%	23.24%	21.74%	23.02%	23.29%	21.55%	23.28%	21.24%	21.93%	19.33%	21.79%	28.58%	25.73%
VII	21.96%	22.30%	27.96%	28.89%	28.22%	25.22%		26.41%	26.18%	28.70%	27.42%	21.20%	27.35%	22.19%	27.86%	24.80%	28.62%	50.06%	27.39%
VIII	21.04%	20.51%	29.53%	34.05%	31.84%	23.24%	26.41%		30.44%	30.47%	31.97%	19.31%	32.62%	20.98%	23.15%	22.25%	23.09%	23.40%	24.89%
X	19.03%	19.74%	29.46%	32.62%	32.49%	21.74%	26.18%	30.44%		31.08%	32.64%	19.19%	31.20%	20.75%	23.06%	23.42%	23.77%	24.43%	24.49%
XI	20.87%	21.27%	29.68%	30.61%	31.20%	23.02%	28.70%	30.47%	31.08%		31.40%	20.30%	29.98%	20.15%	23.99%	22.54%	24.78%	24.40%	23.94%
XII	20.62%	20.06%	29.27%	32.34%	41.87%	23.29%	27.42%	31.97%	32.64%	31.40%		19.59%	32.13%	19.85%	22.67%	23.30%	22.96%	23.33%	24.19%
XIII	30.38%	30.11%	20.14%	21.35%	19.71%	21.55%	21.20%	19.31%	19.19%	20.30%	19.59%		20.45%	30.25%	18.90%	18.37%	18.76%	23.23%	23.14%
XIV	20.88%	19.49%	29.64%	52.87%	32.75%	23.28%	27.35%	32.62%	31.20%	29.98%	32.13%	20.45%		21.37%	22.73%	24.15%	22.78%	25.02%	25.83%
XV	41.51%	36.96%	20.73%	21.58%	20.65%	21.24%	22.19%	20.98%	20.75%	20.15%	19.85%	30.25%	21.37%		19.13%	18.14%	19.25%	22.67%	21.94%
XVI	18.54%	18.30%	23.85%	23.89%	23.82%	21.93%	27.86%	23.15%	23.06%	23.99%	22.67%	18.90%	22.73%	19.13%		17.91%	28.06%	23.31%	22.70%
XVIII	17.47%	18.03%	22.88%	24.52%	22.93%	19.33%	24.80%	22.25%	23.42%	22.54%	23.30%	18.37%	24.15%	18.14%	17.91%		19.92%	25.46%	21.45%
XIX	18.76%	18.08%	24.41%	24.19%	24.08%	21.79%	28.62%	23.09%	23.77%	24.78%	22.96%	18.76%	22.78%	19.25%	28.06%	19.92%		23.53%	21.52%
XX	23.20%	21.91%	24.00%	25.95%	24.06%	28.58%	50.06%	23.40%	24.43%	24.40%	23.33%	23.23%	25.02%	22.67%	23.31%	25.46%	23.53%		27.03%
XXII	23.20%	22.06%	24.75%	26.26%	24.99%	25.73%	27.39%	24.89%	24.49%	23.94%	24.19%	23.14%	25.83%	21.94%	22.70%	21.45%	21.52%	27.03%	

Figure S12 Comparison analysis of the 5 kb non-coding flanking sequences of *SFBB*s in *Pyrus* S_{17}-locus. (a) A snapshot showing the alignment of the 5kb upstream sequences of *SFBB*s. (b) Pairwise identity of the 5kb upstream sequences of *SFBB*s. (c) A snapshot showing the alignment of the 5kb downstream sequences of *SFBB*s. (d) Pairwise identity of the 5kb downstream sequences of *SFBB*s.

(a)

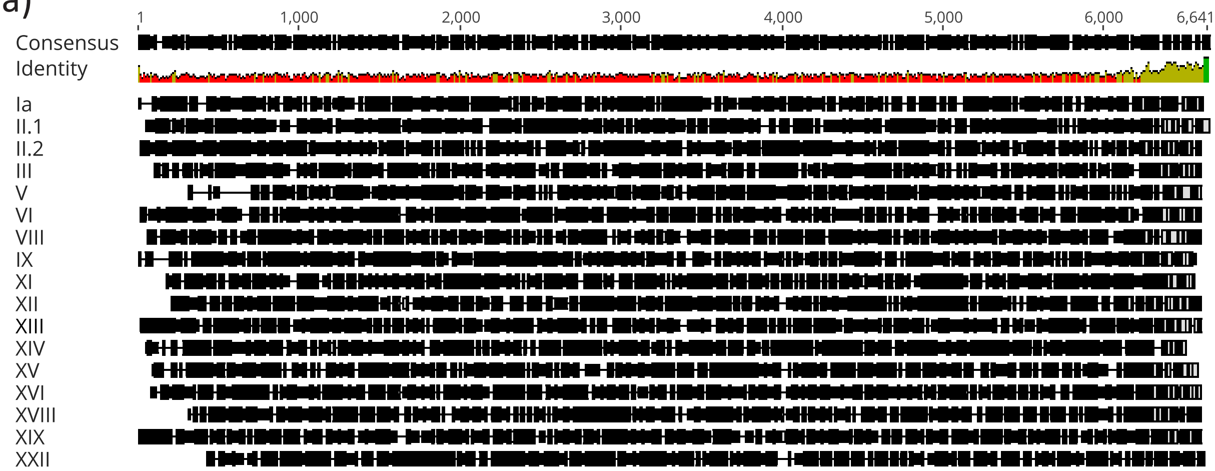

(b)

	Ia	II.1	II.2	III	V	VI	VIII	IX	XI	XII	XIII	XIV	XV	XVI	XVIII	XIX	XXII
Ia		22.47%	23.10%	24.17%	21.57%	26.31%	23.23%	34.92%	22.09%	26.28%	24.08%	22.45%	34.48%	26.27%	31.23%	33.18%	26.58%
II.1	22.47%		43.03%	30.26%	29.35%	23.04%	30.43%	22.03%	30.93%	22.56%	32.77%	29.80%	22.47%	23.03%	23.04%	21.60%	22.06%
II.2	23.10%	43.03%		31.28%	30.60%	23.11%	30.29%	24.11%	31.42%	24.36%	32.22%	29.10%	24.57%	22.92%	22.89%	22.96%	22.26%
III	24.17%	30.26%	31.28%		30.37%	23.21%	30.79%	23.78%	31.06%	23.61%	31.08%	29.82%	23.97%	23.44%	24.95%	23.28%	23.56%
V	21.57%	29.35%	30.60%	30.37%		22.90%	35.89%	21.91%	31.04%	21.72%	36.39%	28.39%	22.11%	22.92%	22.52%	20.11%	21.30%
VI	26.31%	23.04%	23.11%	23.21%	22.90%		24.14%	27.56%	23.48%	36.64%	23.46%	22.37%	26.46%	43.97%	28.57%	25.98%	28.28%
VIII	23.23%	30.43%	30.29%	30.79%	35.89%	24.14%		24.73%	31.42%	23.68%	41.61%	29.55%	24.15%	23.78%	23.78%	22.40%	21.65%
IX	34.92%	22.03%	24.11%	23.78%	21.91%	27.56%	24.73%		22.53%	28.19%	24.49%	24.27%	33.19%	27.48%	31.73%	40.45%	28.28%
XI	22.09%	30.93%	31.42%	31.06%	31.04%	23.48%	31.42%	22.53%		23.70%	31.11%	30.72%	23.07%	23.41%	24.08%	22.34%	22.93%
XII	26.28%	22.56%	24.36%	23.61%	21.72%	36.64%	23.68%	28.19%	23.70%		23.68%	23.35%	27.69%	36.40%	28.45%	27.75%	27.20%
XIII	24.08%	32.77%	32.22%	31.08%	36.39%	23.46%	41.61%	24.49%	31.11%	23.68%		31.32%	22.64%	23.39%	23.25%	22.45%	22.51%
XIV	22.45%	29.80%	29.10%	29.82%	28.39%	22.37%	29.55%	24.27%	30.72%	23.35%	31.32%		22.62%	23.46%	24.05%	22.07%	23.03%
XV	34.48%	22.47%	24.57%	23.97%	22.11%	26.46%	24.15%	33.19%	23.07%	27.69%	22.64%	22.62%		26.92%	32.52%	31.79%	27.48%
XVI	26.27%	23.03%	22.92%	23.44%	22.92%	43.97%	23.78%	27.48%	23.41%	36.40%	23.39%	23.46%	26.92%		28.97%	26.71%	27.67%
XVIII	31.23%	23.04%	22.89%	24.95%	22.52%	28.57%	23.78%	31.73%	24.08%	28.45%	23.25%	24.05%	32.52%	28.97%		30.64%	29.41%
XIX	33.18%	21.60%	22.96%	23.28%	20.11%	25.98%	22.40%	40.45%	22.34%	27.75%	22.45%	22.07%	31.79%	26.71%	30.64%		27.30%
XXII	26.58%	22.06%	22.26%	23.56%	21.30%	28.28%	21.65%	28.28%	22.93%	27.20%	22.51%	23.03%	27.48%	27.67%	29.41%	27.30%	

(c)

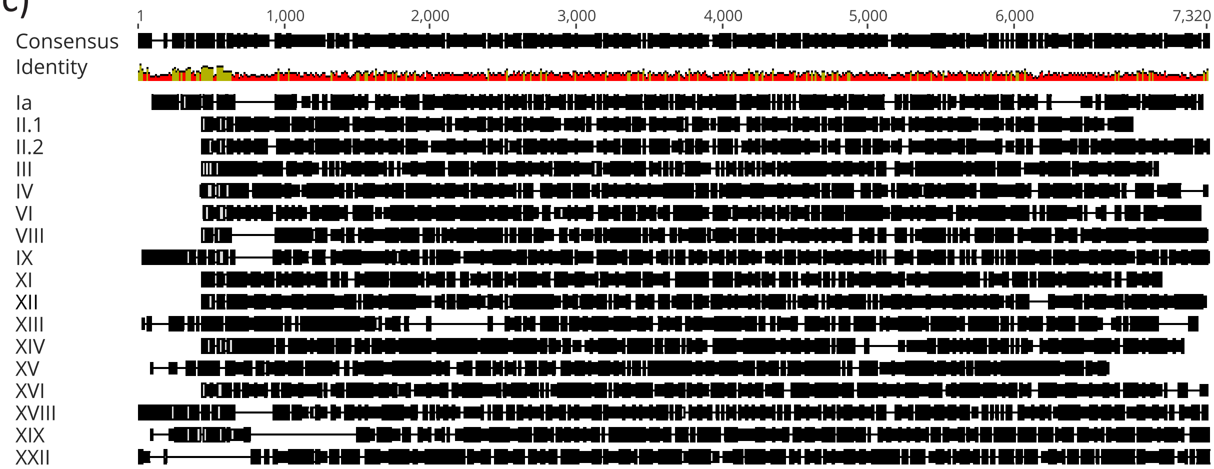

(d)

	Ia	II.1	II.2	III	IV	VI	VIII	IX	XI	XII	XIII	XIV	XV	XVI	XVIII	XIX	XXII
Ia		18.22%	19.83%	28.72%	19.23%	19.79%	32.71%	31.53%	21.05%	20.13%	17.86%	20.41%	19.86%	19.60%	31.44%	20.63%	25.85%
II.1	18.22%		48.27%	22.91%	29.98%	31.62%	21.40%	20.01%	27.98%	35.80%	23.57%	28.81%	27.10%	29.74%	18.60%	25.78%	19.83%
II.2	19.83%	48.27%		23.51%	30.28%	31.78%	22.80%	20.81%	28.99%	36.05%	23.16%	30.15%	26.73%	28.44%	19.87%	27.19%	20.97%
III	28.72%	22.91%	23.51%		23.14%	21.77%	32.04%	29.03%	23.16%	22.70%	21.78%	22.91%	23.08%	23.67%	27.70%	22.38%	28.14%
IV	19.23%	29.98%	30.28%	23.14%		30.34%	22.06%	19.97%	29.51%	29.93%	22.85%	29.17%	27.35%	29.09%	19.29%	26.23%	19.86%
VI	19.79%	31.62%	31.78%	21.77%	30.34%		21.50%	20.63%	29.81%	32.69%	23.06%	28.60%	27.18%	29.06%	19.09%	25.71%	19.82%
VIII	32.71%	21.40%	22.80%	32.04%	22.06%	21.50%		35.16%	22.85%	22.27%	19.47%	22.16%	21.89%	22.90%	34.82%	22.39%	28.68%
IX	31.53%	20.01%	20.81%	29.03%	19.97%	20.63%	35.16%		20.07%	20.79%	18.57%	20.81%	19.10%	20.58%	42.16%	21.45%	24.35%
XI	21.05%	27.98%	28.99%	23.16%	29.51%	29.81%	22.85%	20.07%		30.23%	25.54%	29.11%	28.78%	29.42%	19.74%	26.78%	20.22%
XII	20.13%	35.80%	36.05%	22.70%	29.93%	32.69%	22.27%	20.79%	30.23%		23.36%	30.04%	27.39%	28.21%	20.41%	25.83%	20.08%
XIII	17.86%	23.57%	23.16%	21.78%	22.85%	23.06%	19.47%	18.57%	25.54%	23.36%		22.12%	25.45%	24.03%	17.57%	22.48%	18.60%
XIV	20.41%	28.81%	30.15%	22.91%	29.17%	28.60%	22.16%	20.81%	29.11%	30.04%	22.12%		27.91%	28.28%	19.90%	25.80%	20.63%
XV	19.86%	27.10%	26.73%	23.08%	27.35%	27.18%	21.89%	19.10%	28.78%	27.39%	25.45%	27.91%		26.84%	19.12%	24.98%	21.30%
XVI	19.60%	29.74%	28.44%	23.67%	29.09%	29.06%	22.90%	20.58%	29.42%	28.21%	24.03%	28.28%	26.84%		20.20%	25.94%	20.68%
XVIII	31.44%	18.60%	19.87%	27.70%	19.29%	19.09%	34.82%	42.16%	19.74%	20.41%	17.57%	19.90%	19.12%	20.20%		20.58%	23.59%
XIX	20.63%	25.78%	27.19%	22.38%	26.23%	25.71%	22.39%	21.45%	26.78%	25.83%	22.48%	25.80%	24.98%	25.94%	20.58%		19.97%
XXII	25.85%	19.83%	20.97%	28.14%	19.86%	19.82%	28.68%	24.35%	20.22%	20.08%	18.60%	20.63%	21.30%	20.68%	23.59%	19.97%	

Figure S13 Comparison analysis of the 5 kb non-coding flanking sequences of *SFBB*s in *Pyrus* S_5-locus. (a) A snapshot showing the alignment of the 5kb upstream sequences of *SFBB*s. (b) Pairwise identity of the 5kb upstream sequences of *SFBB*s. (c) A snapshot showing the alignment of the 5kb downstream sequences of *SFBB*s. (d) Pairwise identity of the 5kb downstream sequences of *SFBB*s.

(a)

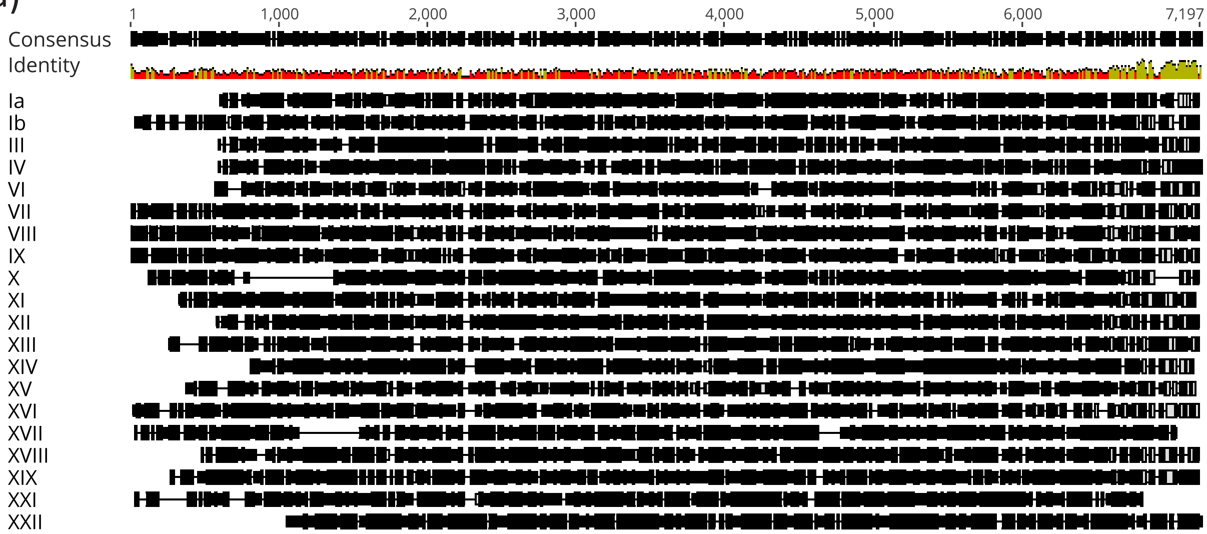

(b)

	Ia	Ib	III	IV	VI	VII	VIII	IX	X	XI	XII	XIII	XIV	XV	XVI	XVII	XVIII	XIX	XXI	XXII
Ia		30.79%	29.93%	19.64%	18.83%	20.57%	18.86%	29.50%	19.26%	29.51%	20.45%	26.25%	19.83%	19.20%	19.78%	29.79%	19.52%	27.77%	26.48%	24.01%
Ib	30.79%		33.86%	19.08%	19.58%	20.19%	20.64%	33.59%	19.88%	29.58%	20.22%	26.62%	19.99%	19.00%	20.20%	30.86%	19.56%	27.01%	27.32%	25.20%
III	29.93%	33.86%		20.46%	20.51%	21.32%	21.05%	41.23%	21.43%	31.50%	20.92%	26.91%	21.21%	19.83%	20.26%	32.52%	20.42%	29.35%	27.14%	26.67%
IV	19.64%	19.08%	20.46%		32.78%	28.36%	34.87%	20.23%	34.72%	21.14%	28.44%	19.32%	31.15%	24.45%	30.17%	20.14%	27.37%	21.58%	20.25%	20.02%
VI	18.83%	19.58%	20.51%	32.78%		28.80%	32.32%	19.82%	32.32%	21.06%	27.79%	19.66%	30.72%	25.05%	29.77%	20.53%	26.39%	21.62%	20.08%	19.18%
VII	20.57%	20.19%	21.32%	28.36%	28.80%		28.41%	21.79%	29.78%	22.28%	28.41%	18.70%	29.25%	27.62%	29.77%	21.49%	26.64%	21.62%	21.36%	19.37%
VIII	18.86%	20.64%	21.05%	34.87%	32.32%	28.41%		21.32%	40.98%	21.54%	27.84%	20.58%	31.51%	24.38%	30.02%	20.90%	27.22%	22.37%	20.65%	19.82%
IX	29.50%	33.59%	41.23%	20.23%	19.82%	21.79%	21.32%		20.83%	29.18%	21.24%	26.45%	20.15%	18.59%	20.08%	31.15%	20.06%	28.52%	26.59%	25.75%
X	19.26%	19.88%	21.43%	34.72%	32.32%	29.78%	40.98%	20.83%		21.17%	27.83%	20.08%	32.49%	24.52%	29.09%	20.02%	26.14%	21.15%	19.40%	19.76%
XI	29.51%	29.58%	31.50%	21.14%	21.06%	22.28%	21.54%	29.18%	21.17%		21.32%	25.57%	21.38%	20.63%	21.19%	31.17%	21.66%	31.11%	27.61%	24.52%
XII	20.45%	20.22%	20.92%	28.44%	27.79%	28.41%	27.84%	21.24%	27.83%	21.32%		19.36%	28.89%	26.89%	28.39%	20.20%	26.42%	21.14%	19.96%	20.20%
XIII	26.25%	26.62%	26.91%	19.32%	19.66%	18.70%	20.58%	26.45%	20.08%	25.57%	19.36%		19.03%	18.63%	18.93%	25.97%	19.45%	26.24%	27.78%	25.93%
XIV	19.83%	19.99%	21.21%	31.15%	30.72%	29.25%	31.51%	20.15%	32.49%	21.38%	28.89%	19.03%		25.69%	30.29%	21.33%	27.39%	21.24%	19.76%	20.09%
XV	19.20%	19.00%	19.83%	24.45%	25.05%	27.62%	24.38%	18.59%	24.52%	20.63%	26.89%	18.63%	25.69%		26.14%	20.53%	25.86%	21.89%	20.01%	18.80%
XVI	19.78%	20.20%	20.26%	30.17%	29.77%	29.77%	30.02%	20.08%	29.09%	21.19%	28.39%	18.93%	30.29%	26.14%		20.67%	26.85%	21.35%	19.61%	20.07%
XVII	29.79%	30.86%	32.52%	20.14%	20.53%	21.49%	20.90%	31.15%	20.02%	31.17%	20.20%	25.97%	21.33%	20.53%	20.67%		20.78%	28.37%	26.00%	25.12%
XVIII	19.52%	19.56%	20.42%	27.37%	26.39%	26.64%	27.22%	20.06%	26.14%	21.66%	26.42%	19.45%	27.39%	25.86%	26.85%	20.78%		22.33%	20.63%	19.23%
XIX	27.77%	27.01%	29.35%	21.58%	21.62%	21.62%	22.37%	28.52%	21.15%	31.11%	21.14%	26.24%	21.24%	21.89%	21.35%	28.37%	22.33%		28.52%	26.66%
XXI	26.48%	27.32%	27.14%	20.25%	20.08%	21.36%	20.65%	26.59%	19.40%	27.61%	19.96%	27.78%	19.76%	20.01%	19.61%	26.00%	20.63%	28.52%		25.89%
XXII	24.01%	25.20%	26.67%	20.02%	19.18%	19.37%	19.82%	25.75%	19.76%	24.52%	20.20%	25.93%	20.09%	18.80%	20.07%	25.12%	19.23%	26.66%	25.89%	

(c)

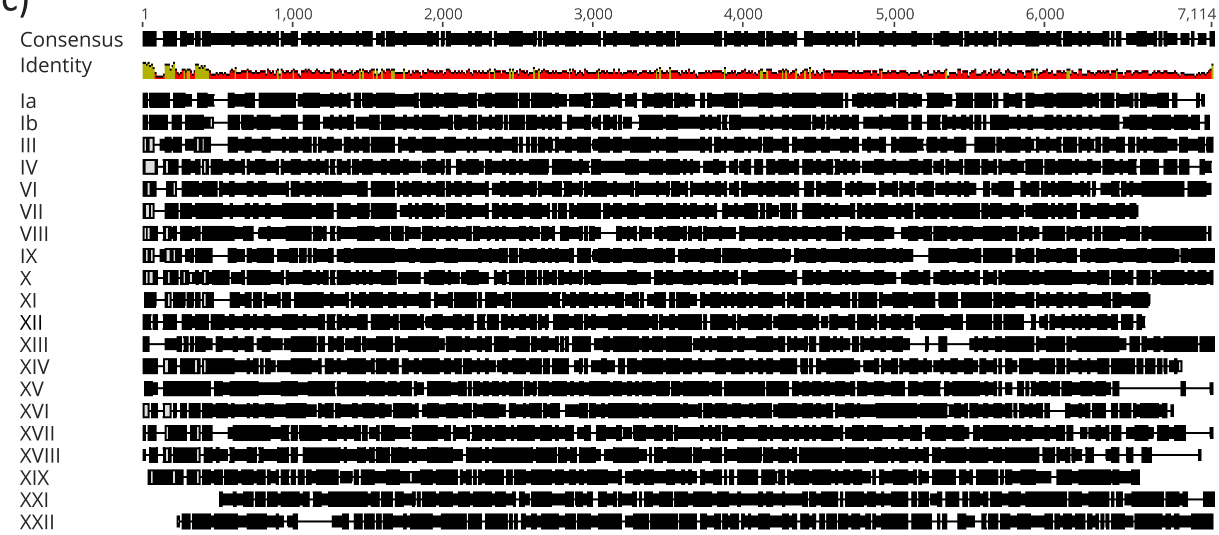

(d)

	Ia	Ib	III	IV	VI	VII	VIII	IX	X	XI	XII	XIII	XIV	XV	XVI	XVII	XVIII	XIX	XXI	XXII
Ia		30.79%	29.93%	19.64%	18.83%	20.57%	18.86%	29.50%	19.26%	29.51%	20.45%	26.25%	19.83%	19.20%	19.78%	29.79%	19.52%	27.77%	26.48%	24.01%
Ib	30.79%		33.86%	19.08%	19.58%	20.19%	20.64%	33.59%	19.88%	29.58%	20.22%	26.62%	19.99%	19.00%	20.20%	30.86%	19.56%	27.01%	27.32%	25.20%
III	29.93%	33.86%		20.46%	20.51%	21.32%	21.05%	41.23%	21.43%	31.50%	20.92%	26.91%	21.21%	19.83%	20.26%	32.52%	20.42%	29.35%	27.14%	26.67%
IV	19.64%	19.08%	20.46%		32.78%	28.36%	34.87%	20.23%	34.72%	21.14%	28.44%	19.32%	31.15%	24.45%	30.17%	20.14%	27.37%	21.58%	20.25%	20.02%
VI	18.83%	19.58%	20.51%	32.78%		28.80%	32.32%	19.82%	32.32%	21.06%	27.79%	19.66%	30.72%	25.05%	29.77%	20.53%	26.39%	21.62%	20.08%	19.18%
VII	20.57%	20.19%	21.32%	28.36%	28.80%		28.41%	21.79%	29.78%	22.28%	28.41%	18.70%	29.25%	27.62%	29.77%	21.49%	26.64%	21.62%	21.36%	19.37%
VIII	18.86%	20.64%	21.05%	34.87%	32.32%	28.41%		21.32%	40.98%	21.54%	27.84%	20.58%	31.51%	24.38%	30.02%	20.90%	27.22%	22.37%	20.65%	19.82%
IX	29.50%	33.59%	41.23%	20.23%	19.82%	21.79%	21.32%		20.83%	29.18%	21.24%	26.45%	20.15%	18.59%	20.08%	31.15%	20.06%	28.52%	26.59%	25.75%
X	19.26%	19.88%	21.43%	34.72%	32.32%	29.78%	40.98%	20.83%		21.17%	27.83%	20.08%	32.49%	24.52%	29.09%	20.02%	26.14%	21.15%	19.40%	19.76%
XI	29.51%	29.58%	31.50%	21.14%	21.06%	22.28%	21.54%	29.18%	21.17%		21.32%	25.57%	21.38%	20.63%	21.19%	31.17%	21.66%	31.11%	27.61%	24.52%
XII	20.45%	20.22%	20.92%	28.44%	27.79%	28.41%	27.84%	21.24%	27.83%	21.32%		19.36%	28.89%	26.89%	28.39%	20.20%	26.42%	21.14%	19.96%	20.20%
XIII	26.25%	26.62%	26.91%	19.32%	19.66%	18.70%	20.58%	26.45%	20.08%	25.57%	19.36%		19.03%	18.63%	18.93%	25.97%	19.45%	26.24%	27.78%	25.93%
XIV	19.83%	19.99%	21.21%	31.15%	30.72%	29.25%	31.51%	20.15%	32.49%	21.38%	28.89%	19.03%		25.69%	30.29%	21.33%	27.39%	21.24%	19.76%	20.09%
XV	19.20%	19.00%	19.83%	24.45%	25.05%	27.62%	24.38%	18.59%	24.52%	20.63%	26.89%	18.63%	25.69%		26.14%	20.53%	25.86%	21.89%	20.01%	18.80%
XVI	19.78%	20.20%	20.26%	30.17%	29.77%	29.77%	30.02%	20.08%	29.09%	21.19%	28.39%	18.93%	30.29%	26.14%		20.67%	26.85%	21.35%	19.61%	20.07%
XVII	29.79%	30.86%	32.52%	20.14%	20.53%	21.49%	20.90%	31.15%	20.02%	31.17%	20.20%	25.97%	21.33%	20.53%	20.67%		20.78%	28.37%	26.00%	25.12%
XVIII	19.52%	19.56%	20.42%	27.37%	26.39%	26.64%	27.22%	20.06%	26.14%	21.66%	26.42%	19.45%	27.39%	25.86%	26.85%	20.78%		22.33%	20.63%	19.23%
XIX	27.77%	27.01%	29.35%	21.58%	21.62%	21.62%	22.37%	28.52%	21.15%	31.11%	21.14%	26.24%	21.24%	21.89%	21.35%	28.37%	22.33%		28.52%	26.66%
XXI	26.48%	27.32%	27.14%	20.25%	20.08%	21.36%	20.65%	26.59%	19.40%	27.61%	19.96%	27.78%	19.76%	20.01%	19.61%	26.00%	20.63%	28.52%		25.89%
XXII	24.01%	25.20%	26.67%	20.02%	19.18%	19.37%	19.82%	25.75%	19.76%	24.52%	20.20%	25.93%	20.09%	18.80%	20.07%	25.12%	19.23%	26.66%	25.89%	

Figure S14 Comparison analysis of the 5 kb non-coding flanking sequences of *SFBB*s in *Pyrus* S_{67}-locus. (a) A snapshot showing the alignment of the 5kb upstream sequences of *SFBB*s. (b) Pairwise identity of the 5kb upstream sequences of *SFBB*s. (c) A snapshot showing the alignment of the 5kb downstream sequences of *SFBB*s. (d) Pairwise identity of the 5kb downstream sequences of *SFBB*s.

(a)

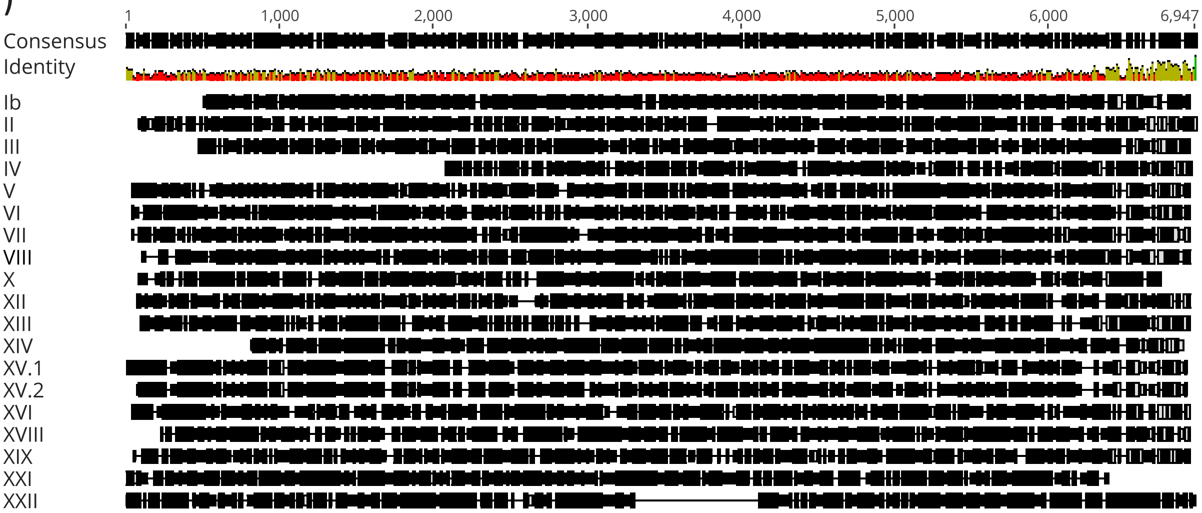

(b)

	Ib	II	III	IV	V	VI	VII	VIII	X	XII	XIII	XIV	XV.1	XV.2	XVI	XVIII	XIX	XXI	XXII
Ib		23.54%	41.61%	24.90%	27.18%	26.29%	27.13%	27.39%	23.54%	23.87%	22.91%	30.08%	27.01%	27.24%	26.56%	26.33%	26.65%	26.07%	22.96%
II	23.54%		24.56%	34.33%	22.94%	23.07%	24.09%	24.67%	31.23%	40.72%	36.22%	24.11%	22.60%	22.49%	23.58%	24.12%	23.43%	24.82%	22.96%
III	41.61%	24.56%		24.73%	27.68%	26.99%	27.43%	28.19%	22.40%	24.89%	22.98%	27.88%	27.13%	26.77%	26.71%	27.42%	27.10%	25.89%	21.83%
IV	24.90%	34.33%	24.73%		24.02%	25.02%	24.83%	26.00%	31.02%	34.33%	33.07%	26.21%	22.95%	22.73%	25.08%	25.63%	25.41%	25.01%	21.92%
V	27.18%	22.94%	27.68%	24.02%		32.97%	33.01%	31.43%	22.33%	23.46%	23.23%	29.37%	26.09%	26.66%	32.13%	30.73%	33.07%	24.80%	22.20%
VI	26.29%	23.07%	26.99%	25.02%	32.97%		37.36%	31.62%	23.17%	24.27%	24.53%	29.89%	26.47%	26.19%	43.05%	30.06%	33.26%	25.22%	23.07%
VII	27.13%	24.09%	27.43%	24.83%	33.01%	37.36%		32.24%	23.51%	24.10%	23.71%	29.74%	26.63%	27.70%	37.54%	30.36%	33.82%	25.62%	23.07%
VIII	27.39%	24.67%	28.19%	26.00%	31.43%	31.62%	32.24%		23.90%	23.82%	24.80%	28.58%	24.85%	25.21%	31.41%	31.24%	30.11%	26.33%	21.20%
X	23.54%	31.23%	22.40%	31.02%	22.33%	23.17%	23.51%	23.90%		32.02%	32.17%	23.99%	21.58%	21.80%	22.83%	24.45%	23.21%	26.53%	21.88%
XII	23.87%	40.72%	24.89%	34.33%	23.46%	24.27%	24.10%	23.82%	32.02%		37.29%	24.99%	23.50%	23.19%	23.41%	24.92%	22.88%	24.81%	21.68%
XIII	22.91%	36.22%	22.98%	33.07%	23.23%	24.53%	23.71%	24.80%	32.17%	37.29%		24.52%	23.08%	23.22%	23.33%	24.41%	24.04%	25.50%	21.90%
XIV	30.08%	24.11%	27.88%	26.21%	29.37%	29.89%	29.74%	28.58%	23.99%	24.99%	24.52%		48.59%	48.21%	29.73%	28.59%	29.22%	26.58%	24.47%
XV.1	27.01%	22.60%	27.13%	22.95%	26.09%	26.47%	26.63%	24.85%	21.58%	23.50%	23.08%	48.59%		68.88%	26.49%	26.23%	25.52%	25.36%	23.10%
XV.2	27.24%	22.49%	26.77%	22.73%	26.66%	26.19%	27.70%	25.21%	21.80%	23.19%	23.22%	48.21%	68.88%		26.27%	25.72%	26.43%	25.26%	22.93%
XVI	26.56%	23.58%	26.71%	25.08%	32.13%	43.05%	37.54%	31.41%	22.83%	23.41%	23.33%	29.73%	26.49%	26.27%		29.64%	33.27%	24.77%	23.33%
XVIII	26.33%	24.12%	27.42%	25.63%	30.73%	30.06%	30.36%	31.24%	24.45%	24.92%	24.41%	28.59%	26.23%	25.72%	29.64%		29.86%	25.78%	22.55%
XIX	26.65%	23.43%	27.10%	25.41%	33.07%	33.26%	33.82%	30.11%	23.21%	22.88%	24.04%	29.22%	25.52%	26.43%	33.27%	29.86%		24.78%	22.64%
XXI	26.07%	24.82%	25.89%	25.01%	24.80%	25.22%	25.62%	26.33%	26.53%	24.81%	25.50%	26.58%	25.36%	25.26%	24.77%	25.78%	24.78%		23.03%
XXII	22.96%	22.96%	21.83%	21.92%	22.20%	23.07%	23.07%	21.20%	21.88%	21.68%	21.90%	24.47%	23.10%	22.93%	23.33%	22.55%	22.64%	23.03%	

(c)

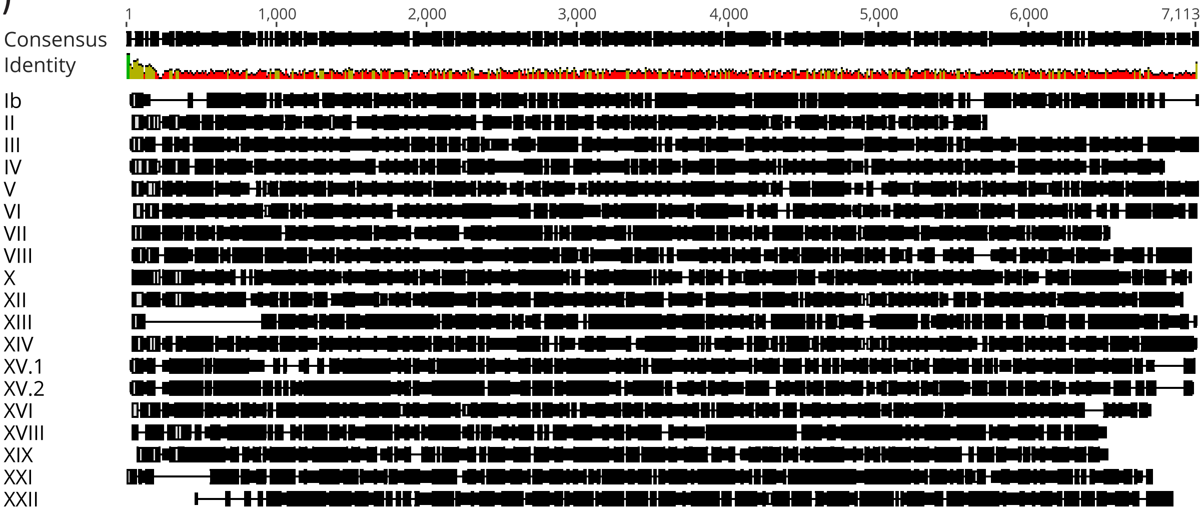

(d)

	Ib	II	III	IV	V	VI	VII	VIII	X	XII	XIII	XIV	XV.1	XV.2	XVI	XVIII	XIX	XXI	XXII
Ib		23.84%	24.52%	25.43%	24.17%	23.65%	28.02%	24.89%	24.49%	25.85%	25.32%	24.62%	25.64%	25.13%	26.33%	25.55%	24.80%	28.40%	25.72%
II	23.84%		24.28%	25.85%	34.45%	31.44%	26.75%	25.15%	28.55%	31.86%	22.58%	36.33%	23.38%	24.11%	27.29%	25.37%	25.19%	24.10%	22.58%
III	24.52%	24.28%		42.93%	24.68%	25.19%	28.29%	34.75%	24.61%	25.76%	23.26%	25.89%	25.08%	24.46%	27.58%	26.05%	25.45%	23.98%	24.46%
IV	25.43%	25.85%	42.93%		27.67%	26.38%	29.25%	35.99%	27.96%	27.85%	23.35%	28.18%	24.85%	25.30%	27.68%	27.01%	28.25%	24.75%	23.39%
V	24.17%	34.45%	24.68%	27.67%		33.19%	27.39%	25.89%	30.12%	32.50%	23.37%	43.99%	24.42%	24.04%	28.51%	25.16%	27.61%	23.37%	24.81%
VI	23.65%	31.44%	25.19%	26.38%	33.19%		27.52%	25.31%	28.52%	31.23%	23.38%	32.30%	24.40%	23.73%	28.38%	25.15%	26.36%	24.41%	23.94%
VII	28.02%	26.75%	28.29%	29.25%	27.39%	27.52%		28.04%	28.16%	29.05%	25.02%	28.14%	25.69%	27.27%	28.74%	26.30%	27.60%	25.85%	25.07%
VIII	24.89%	25.15%	34.75%	35.99%	25.89%	25.31%	28.04%		26.43%	27.07%	21.76%	26.41%	23.49%	23.09%	27.20%	26.27%	26.59%	24.30%	23.22%
X	24.49%	28.55%	24.61%	27.96%	30.12%	28.52%	28.16%	26.43%		30.54%	22.90%	30.43%	23.86%	23.41%	27.37%	26.84%	28.03%	23.86%	23.57%
XII	25.85%	31.86%	25.76%	27.85%	32.50%	31.23%	29.05%	27.07%	30.54%		23.83%	32.34%	24.57%	23.31%	28.19%	27.13%	27.52%	26.13%	24.48%
XIII	25.32%	22.58%	23.26%	23.35%	23.37%	23.38%	25.02%	21.76%	22.90%	23.83%		23.85%	22.66%	22.09%	24.83%	23.77%	23.65%	25.84%	26.95%
XIV	24.62%	36.33%	25.89%	28.18%	43.99%	32.30%	28.14%	26.41%	30.43%	32.34%	23.85%		23.88%	24.14%	27.81%	25.01%	26.83%	24.68%	23.89%
XV.1	25.64%	23.38%	25.08%	24.85%	24.42%	24.40%	25.69%	23.49%	23.86%	24.57%	22.66%	23.88%		57.41%	25.01%	25.62%	26.09%	25.26%	23.76%
XV.2	25.13%	24.11%	24.46%	25.30%	24.04%	23.73%	27.27%	23.09%	23.41%	23.31%	22.09%	24.14%	57.41%		25.65%	25.66%	26.86%	25.35%	23.27%
XVI	26.33%	27.29%	27.58%	27.68%	28.51%	28.38%	28.74%	27.20%	27.37%	28.19%	24.83%	27.81%	25.01%	25.65%		27.60%	27.66%	25.82%	24.59%
XVIII	25.55%	25.37%	26.05%	27.01%	25.16%	25.15%	26.30%	26.27%	26.84%	27.13%	23.77%	25.01%	25.62%	25.66%	27.60%		27.16%	25.93%	25.81%
XIX	24.80%	25.19%	25.45%	28.25%	27.61%	26.36%	27.60%	26.59%	28.03%	27.52%	23.65%	26.83%	26.09%	26.86%	27.66%	27.16%		25.93%	25.71%
XXI	28.40%	24.10%	23.98%	24.75%	23.37%	24.41%	25.85%	24.30%	23.86%	26.13%	25.84%	24.68%	25.26%	25.35%	25.82%	25.93%	25.93%		25.53%
XXII	25.72%	22.58%	24.46%	23.39%	24.81%	23.94%	25.07%	23.22%	23.57%	24.48%	26.95%	23.89%	23.76%	23.27%	24.59%	25.81%	25.71%	25.53%	

Figure S15 Comparison analysis of the 5 kb non-coding flanking sequences of *SFBB*s in *Pyrus* S_{101}-locus. (a) A snapshot showing the alignment of the 5kb upstream sequences of *SFBB*s. (b) Pairwise identity of the 5kb upstream sequences of *SFBB*s. (c) A snapshot showing the alignment of the 5kb downstream sequences of *SFBB*s. (d) Pairwise identity of the 5kb downstream sequences of *SFBB*s.
