# Supplementary material for: Long-read genome sequencing reveals the sequence characteristics of pear self-incompatibility locus
Source: Mol Hortic. 2025 Mar 1;5:13. doi: 10.1186/s43897-024-00132-0 (PMC11871771; doi:10.1186/s43897-024-00132-0)
Supplement: Supplementary file 1 — Supplementary Material 1: Table S1 Comparison of ‘Yali’ genome with previously published assemblies of Pyrus and Malus species. Table S2 Annotation of the repeats in ‘Yali’ genome. Table S3 Annotation of the non-coding RNAs in ‘Dananguo’ and 'Yali' genomes. Table S4 Identification of the F-box genes in Pyrus, Malus and Prunus S-loci. Table S5 Function annotation of the predicted genes in S-loci. Table S6 Sequence similarity (%) among Pyrus and Malus SFBB genes. Table S7 Sequence similarity (%) among Prunus SFB and SLF genes. Table S8 Sequence similarity among Prunus SFB and SLF genes. Table S9 Sequence similarity (%) among Pyrus and Malus S-RNase genes. Table S10 Prediction of gene duplication events of Pyrus and Malus SFBB genes. Table S11 Sequence similarity of the non-coding flanking sequences of SFBBs in Pyrus and Malus S-loci. Table S12 Analysis of number and length of LTR retrotransposon in different S-loci. Table S13 Identification of the LTR retrotransposon in different S-loci. Table S14 RPKM values of the genes commonly existed in the tested S-loci. Table S15 Sequence similarity (%) among the reported Pyrus S-RNase genes. Table S16 The accession numbers of S-RNase and S-locus F-box genes in Pyrus, Malus, and Prunus.Table S17 Primers used in this study. Figure S1 Isolation of the conserved F-box motif in the reported S-locus F-box proteins in Pyrus and Malus. The accession numbers of these F-box proteins were listed in Table S13. Figure S2 Phylogenetic classifications of S-locus F-box genes in Prunus. The S-locus F-box (SLF/SFB) proteins in Prunus comprised by 12 groups, SLF1→SLF11 and SFB. Each group were highlighted with different colors. Figure S3 Phylogenetic analysis of the F-box genes identified from this and previous studies. Cycles with black color present the F-box genes identified from previous study (Huang et al., 2023). The rates (%) of different types of gene duplication events (dispersed, proximal, tandem and transposed) of the S-locus F-box ge [file 43897_2024_132_MOESM1_ESM.zip › Supplementary Figures S16 to S19.pdf]

(a)

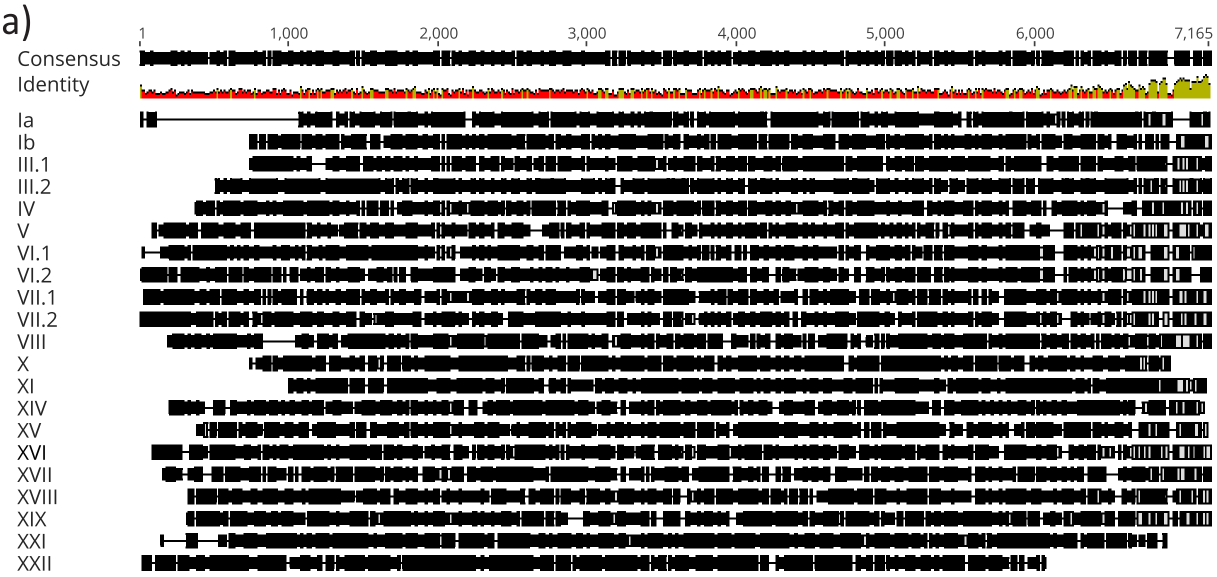

(b)

	Ia	Ib	III.1	III.2	IV	V	VI.1	VI.2	VII.1	VII.2	VIII	X	XI	XIV	XV	XVI	XVII	XVIII	XIX	XXI	XXII
Ia		27.39%	25.93%	26.12%	23.60%	23.28%	23.61%	22.18%	23.77%	22.37%	23.99%	27.70%	28.23%	23.67%	25.88%	23.11%	25.37%	23.08%	24.45%	25.47%	24.32%
Ib	27.39%		28.95%	30.22%	28.39%	28.98%	27.70%	26.80%	29.61%	28.03%	24.88%	28.50%	28.88%	24.68%	29.99%	28.88%	25.51%	28.81%	28.84%	26.08%	26.95%
III.1	25.93%	28.95%		30.97%	29.07%	28.74%	28.35%	25.94%	29.47%	28.49%	25.05%	26.61%	30.29%	26.61%	29.56%	27.99%	26.80%	29.09%	29.43%	26.02%	25.55%
III.2	26.12%	30.22%	30.97%		29.17%	29.16%	28.75%	27.61%	29.65%	28.83%	24.78%	26.77%	28.79%	26.13%	30.91%	29.00%	26.58%	28.65%	29.33%	25.73%	25.06%
IV	23.60%	28.39%	29.07%	29.17%		30.79%	29.57%	28.47%	30.85%	29.86%	26.29%	26.73%	28.92%	25.35%	29.72%	30.63%	27.16%	30.71%	31.04%	24.80%	25.62%
V	23.28%	28.98%	28.74%	29.16%	30.79%		32.67%	30.93%	38.55%	36.27%	24.51%	26.20%	27.27%	24.08%	29.14%	35.78%	25.02%	29.61%	31.87%	23.32%	23.47%
VI.1	23.61%	27.70%	28.35%	28.75%	29.57%	32.67%		48.91%	32.91%	30.45%	24.99%	26.62%	28.18%	25.29%	29.27%	32.62%	26.12%	30.02%	31.52%	24.68%	24.54%
VI.2	22.18%	26.80%	25.94%	27.61%	28.47%	30.93%	48.91%		32.67%	31.36%	23.55%	25.10%	26.68%	23.85%	28.05%	31.91%	25.24%	28.80%	29.34%	23.31%	24.61%
VII.1	23.77%	29.61%	29.47%	29.65%	30.85%	38.55%	32.91%	32.67%		46.36%	26.34%	27.59%	28.75%	26.41%	30.39%	41.04%	27.82%	30.68%	32.64%	24.78%	24.03%
VII.2	22.37%	28.03%	28.49%	28.83%	29.86%	36.27%	30.45%	31.36%	46.36%		25.20%	26.60%	27.36%	25.08%	29.15%	39.24%	26.52%	30.33%	30.42%	23.61%	24.00%
VIII	23.99%	24.88%	25.05%	24.78%	26.29%	24.51%	24.99%	23.55%	26.34%	25.20%		25.21%	28.02%	36.34%	25.76%	25.53%	35.48%	25.17%	24.68%	24.54%	23.47%
X	27.70%	28.50%	26.61%	26.77%	26.73%	26.20%	26.62%	25.10%	27.59%	26.60%	25.21%		27.77%	26.56%	27.81%	27.19%	26.42%	27.60%	26.24%	25.29%	26.77%
XI	28.23%	28.88%	30.29%	28.79%	28.92%	27.27%	28.18%	26.68%	28.75%	27.36%	28.02%	27.77%		27.96%	28.71%	28.01%	28.09%	26.82%	27.00%	28.59%	26.90%
XIV	23.67%	24.68%	26.61%	26.13%	25.35%	24.08%	25.29%	23.85%	26.41%	25.08%	36.34%	26.56%	27.96%		25.77%	24.92%	52.39%	25.18%	24.56%	25.47%	25.02%
XV	25.88%	29.99%	29.56%	30.91%	29.72%	29.14%	29.27%	28.05%	30.39%	29.15%	25.76%	27.81%	28.71%	25.77%		30.38%	25.74%	29.43%	30.30%	25.92%	26.82%
XVI	23.11%	28.88%	27.99%	29.00%	30.63%	35.78%	32.62%	31.91%	41.04%	39.24%	25.53%	27.19%	28.01%	24.92%	30.38%		27.22%	30.35%	32.25%	23.74%	24.02%
XVII	25.37%	25.51%	26.80%	26.58%	27.16%	25.02%	26.12%	25.24%	27.82%	26.52%	35.48%	26.42%	28.09%	52.39%	25.74%	27.22%		25.23%	25.55%	24.64%	25.55%
XVIII	23.08%	28.81%	29.09%	28.65%	30.71%	29.61%	30.02%	28.80%	30.68%	30.33%	25.17%	27.60%	26.82%	25.18%	29.43%	30.35%	25.23%		31.06%	24.13%	24.46%
XIX	24.45%	28.84%	29.43%	29.33%	31.04%	31.87%	31.52%	29.34%	32.64%	30.42%	24.68%	26.24%	27.00%	24.56%	30.30%	32.25%	25.55%	31.06%		25.61%	24.78%
XXI	25.47%	26.08%	26.02%	25.73%	24.80%	23.32%	24.68%	23.31%	24.78%	23.61%	24.54%	25.29%	28.59%	25.47%	25.92%	23.74%	24.64%	24.13%	25.61%		26.32%
XXII	24.32%	26.95%	25.55%	25.06%	25.62%	23.47%	24.54%	24.61%	24.03%	24.00%	23.47%	26.77%	26.90%	25.02%	26.82%	24.02%	25.55%	24.46%	24.78%	26.32%	

(c)

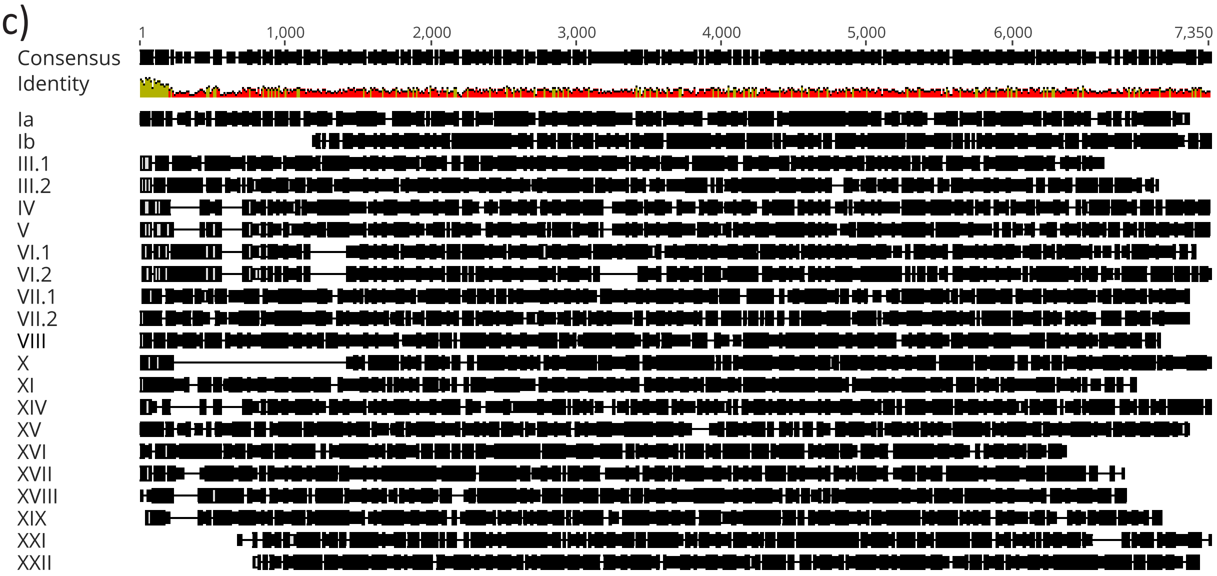

(d)

	Ia	Ib	III.1	III.2	IV	V	VI.1	VI.2	VII.1	VII.2	VIII	X	XI	XIV	XV	XVI	XVII	XVIII	XIX	XXI	XXII
Ia		23.58%	22.30%	21.86%	21.36%	21.28%	22.68%	21.57%	33.90%	35.24%	22.64%	19.71%	23.96%	20.50%	33.33%	23.44%	21.66%	21.91%	21.28%	23.41%	22.62%
Ib	23.58%		25.68%	24.34%	23.85%	24.34%	24.61%	24.21%	22.94%	23.17%	24.58%	23.03%	24.72%	24.92%	23.51%	26.06%	24.75%	25.35%	23.88%	25.15%	25.66%
III.1	22.30%	25.68%		30.16%	26.55%	26.67%	27.01%	26.85%	21.98%	23.01%	30.16%	22.26%	28.50%	25.71%	22.11%	28.30%	29.02%	27.87%	28.14%	24.71%	27.06%
III.2	21.86%	24.34%	30.16%		24.65%	25.54%	26.58%	26.27%	22.23%	22.53%	39.17%	21.41%	27.59%	25.54%	22.57%	27.45%	27.00%	27.12%	26.55%	24.37%	25.77%
IV	21.36%	23.85%	26.55%	24.65%		42.58%	29.07%	29.73%	21.55%	21.76%	24.85%	21.46%	27.22%	35.85%	21.86%	25.51%	26.67%	25.72%	28.93%	23.07%	25.37%
V	21.28%	24.34%	26.67%	25.54%	42.58%		29.88%	30.50%	21.67%	21.58%	25.05%	22.78%	26.29%	37.42%	21.47%	25.58%	27.63%	26.89%	29.24%	23.65%	25.96%
VI.1	22.68%	24.61%	27.01%	26.58%	29.07%	29.88%		66.93%	21.35%	22.86%	26.33%	22.45%	26.58%	29.49%	21.65%	26.08%	25.86%	25.89%	28.42%	23.98%	25.29%
VI.2	21.57%	24.21%	26.85%	26.27%	29.73%	30.50%	66.93%		21.35%	22.09%	26.37%	23.01%	25.76%	29.51%	21.36%	25.99%	25.99%	25.68%	28.58%	23.18%	25.66%
VII.1	33.90%	22.94%	21.98%	22.23%	21.55%	21.67%	21.35%	21.35%		46.26%	23.14%	19.67%	23.37%	21.36%	31.88%	23.55%	21.70%	21.50%	21.14%	22.22%	23.06%
VII.2	35.24%	23.17%	23.01%	22.53%	21.76%	21.58%	22.86%	22.09%	46.26%		23.24%	19.39%	23.72%	21.88%	31.24%	23.90%	22.16%	21.65%	21.30%	22.63%	22.15%
VIII	22.64%	24.58%	30.16%	39.17%	24.85%	25.05%	26.33%	26.37%	23.14%	23.24%		20.64%	28.61%	25.53%	22.56%	27.58%	26.93%	26.65%	26.24%	24.41%	25.40%
X	19.71%	23.03%	22.26%	21.41%	21.46%	22.78%	22.45%	23.01%	19.67%	19.39%	20.64%		22.45%	21.55%	19.59%	21.35%	22.11%	21.56%	21.23%	21.48%	21.77%
XI	23.96%	24.72%	28.50%	27.59%	27.22%	26.29%	26.58%	25.76%	23.37%	23.72%	28.61%	22.45%		26.02%	23.11%	27.92%	27.86%	27.95%	27.28%	24.37%	26.07%
XIV	20.50%	24.92%	25.71%	25.54%	35.85%	37.42%	29.49%	29.51%	21.36%	21.88%	25.53%	21.55%	26.02%		21.82%	25.22%	25.61%	25.20%	28.67%	23.92%	25.13%
XV	33.33%	23.51%	22.11%	22.57%	21.86%	21.47%	21.65%	21.36%	31.88%	31.24%	22.56%	19.59%	23.11%	21.82%		22.39%	22.41%	21.28%	21.22%	23.26%	22.80%
XVI	23.44%	26.06%	28.30%	27.45%	25.51%	25.58%	26.08%	25.99%	23.55%	23.90%	27.58%	21.35%	27.92%	25.22%	22.39%		27.39%	26.52%	26.82%	25.42%	28.60%
XVII	21.66%	24.75%	29.02%	27.00%	26.67%	27.63%	25.86%	25.99%	21.70%	22.16%	26.93%	22.11%	27.86%	25.61%	22.41%	27.39%		28.72%	27.39%	24.52%	25.77%
XVIII	21.91%	25.35%	27.87%	27.12%	25.72%	26.89%	25.89%	25.68%	21.50%	21.65%	26.65%	21.56%	27.95%	25.20%	21.28%	26.52%	28.72%		26.16%	24.75%	26.57%
XIX	21.28%	23.88%	28.14%	26.55%	28.93%	29.24%	28.42%	28.58%	21.14%	21.30%	26.24%	21.23%	27.28%	28.67%	21.22%	26.82%	27.39%	26.16%		24.22%	25.29%
XXI	23.41%	25.15%	24.71%	24.37%	23.07%	23.65%	23.98%	23.18%	22.22%	22.63%	24.41%	21.48%	24.37%	23.92%	23.26%	25.42%	24.52%	24.75%	24.22%		25.08%
XXII	22.62%	25.66%	27.06%	25.77%	25.37%	25.96%	25.29%	25.66%	23.06%	22.15%	25.40%	21.77%	26.07%	25.13%	22.80%	28.60%	25.77%	26.57%	25.29%	25.08%	

Figure S16 Comparison analysis of the 5 kb non-coding flanking sequences of *SFBB*s in *Malus* S_2-locus. (a) A snapshot showing the alignment of the 5kb upstream sequences of *SFBB*s. (b) Pairwise identity of the 5kb upstream sequences of *SFBB*s. (c) A snapshot showing the alignment of the 5kb downstream sequences of *SFBB*s. (d) Pairwise identity of the 5kb downstream sequences of *SFBB*s.

(a)

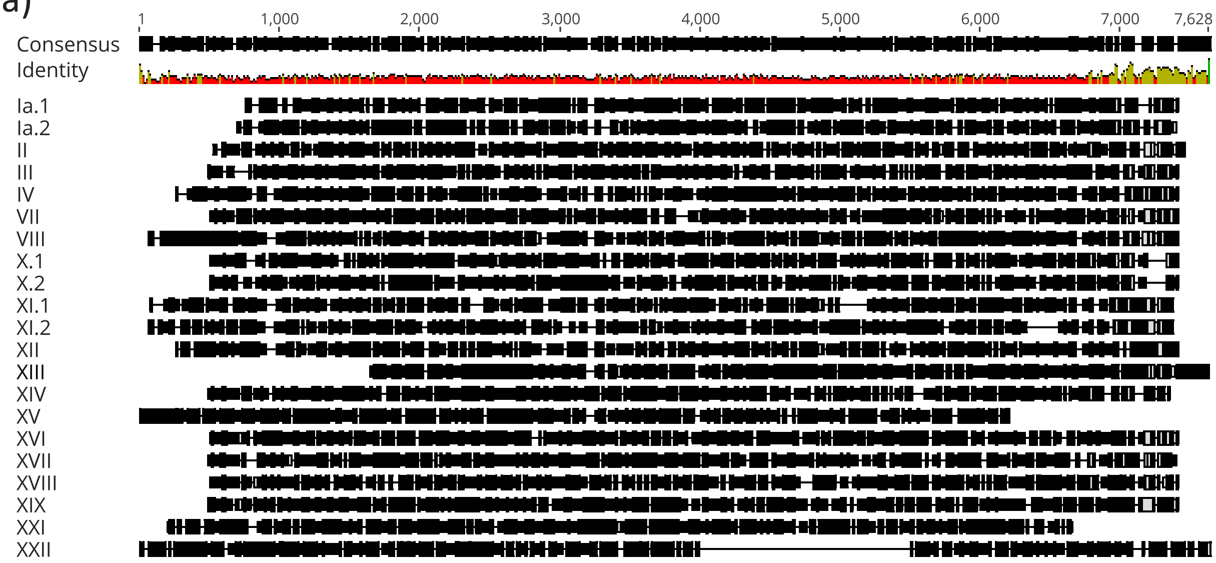

(b)

	Ia.1	Ia.2	II	III	IV	VII	VIII	X.1	X.2	XI.1	XI.2	XII	XIII	XIV	XV	XVI	XVII	XVIII	XIX	XXI	XXII
Ia.1		41.13%	21.27%	21.21%	25.09%	21.77%	24.56%	21.06%	21.18%	23.73%	24.08%	23.88%	28.89%	21.60%	25.04%	21.86%	21.38%	20.49%	21.61%	26.62%	19.46%
Ia.2	41.13%		22.65%	21.91%	25.98%	22.24%	25.96%	21.41%	21.52%	26.03%	25.46%	24.29%	28.58%	21.57%	26.26%	22.99%	22.73%	21.45%	22.54%	26.49%	19.61%
II	21.27%	22.65%		27.17%	23.06%	28.10%	22.95%	25.29%	24.09%	20.68%	20.74%	21.50%	24.34%	35.99%	20.51%	28.75%	36.43%	24.74%	28.45%	21.12%	20.21%
III	21.21%	21.91%	27.17%		21.21%	33.11%	22.01%	25.42%	25.65%	20.47%	20.31%	20.63%	23.67%	27.24%	19.86%	32.76%	26.57%	25.90%	32.49%	21.03%	19.98%
IV	25.09%	25.98%	23.06%	21.21%		21.73%	40.64%	20.98%	20.27%	29.13%	29.76%	34.38%	30.59%	21.62%	25.15%	23.01%	22.12%	21.33%	23.17%	24.53%	19.11%
VII	21.77%	22.24%	28.10%	33.11%	21.73%		23.03%	24.59%	25.43%	20.85%	21.93%	21.68%	23.23%	28.15%	21.37%	37.25%	27.86%	26.34%	40.90%	21.10%	20.44%
VIII	24.56%	25.96%	22.95%	22.01%	40.64%	23.03%		21.52%	20.21%	29.98%	30.71%	35.86%	31.99%	22.45%	27.22%	22.79%	21.92%	22.31%	23.35%	26.71%	21.29%
X.1	21.06%	21.41%	25.29%	25.42%	20.98%	24.59%	21.52%		37.01%	19.93%	20.06%	19.67%	22.14%	26.16%	21.52%	25.58%	25.09%	35.26%	24.94%	20.72%	20.07%
X.2	21.18%	21.52%	24.09%	25.65%	20.27%	25.43%	20.21%	37.01%		18.63%	18.95%	19.86%	21.82%	24.70%	20.57%	25.64%	25.11%	46.11%	25.70%	20.07%	18.79%
XI.1	23.73%	26.03%	20.68%	20.47%	29.13%	20.85%	29.98%	19.93%	18.63%		44.08%	29.41%	28.82%	21.43%	22.96%	20.79%	21.50%	20.30%	21.77%	24.44%	20.19%
XI.2	24.08%	25.46%	20.74%	20.31%	29.76%	21.93%	30.71%	20.06%	18.95%	44.08%		28.86%	29.13%	21.47%	25.67%	22.14%	21.32%	20.13%	21.73%	24.33%	18.75%
XII	23.88%	24.29%	21.50%	20.63%	34.38%	21.68%	35.86%	19.67%	19.86%	29.41%	28.86%		29.24%	21.82%	23.90%	21.56%	21.69%	21.13%	22.13%	24.27%	19.49%
XIII	28.89%	28.58%	24.34%	23.67%	30.59%	23.23%	31.99%	22.14%	21.82%	28.82%	29.13%	29.24%		24.54%	53.92%	24.28%	24.83%	22.40%	24.70%	29.19%	21.07%
XIV	21.60%	21.57%	35.99%	27.24%	21.62%	28.15%	22.45%	26.16%	24.70%	21.43%	21.47%	21.82%	24.54%		21.12%	28.43%	51.02%	27.30%	28.08%	23.38%	21.17%
XV	25.04%	26.26%	20.51%	19.86%	25.15%	21.37%	27.22%	21.52%	20.57%	22.96%	25.67%	23.90%	53.92%	21.12%		21.46%	21.22%	20.03%	21.52%	28.33%	21.77%
XVI	21.86%	22.99%	28.75%	32.76%	23.01%	37.25%	22.79%	25.58%	25.64%	20.79%	22.14%	21.56%	24.28%	28.43%	21.46%		27.72%	26.27%	37.16%	21.40%	20.60%
XVII	21.38%	22.73%	36.43%	26.57%	22.12%	27.86%	21.92%	25.09%	25.11%	21.50%	21.32%	21.69%	24.83%	51.02%	21.22%	27.72%		26.70%	27.14%	21.63%	20.16%
XVIII	20.49%	21.45%	24.74%	25.90%	21.33%	26.34%	22.31%	35.26%	46.11%	20.30%	20.13%	21.13%	22.40%	27.30%	20.03%	26.27%	26.70%		26.82%	21.13%	19.40%
XIX	21.61%	22.54%	28.45%	32.49%	23.17%	40.90%	23.35%	24.94%	25.70%	21.77%	21.73%	22.13%	24.70%	28.08%	21.52%	37.16%	27.14%	26.82%		20.16%	20.97%
XXI	26.62%	26.49%	21.12%	21.03%	24.53%	21.10%	26.71%	20.72%	20.07%	24.44%	24.33%	24.27%	29.19%	23.38%	28.33%	21.40%	21.63%	21.13%	20.16%		20.85%
XXII	19.46%	19.61%	20.21%	19.98%	19.11%	20.44%	21.29%	20.07%	18.79%	20.19%	18.75%	19.49%	21.07%	21.17%	21.77%	20.60%	20.16%	19.40%	20.97%	20.85%	

(c)

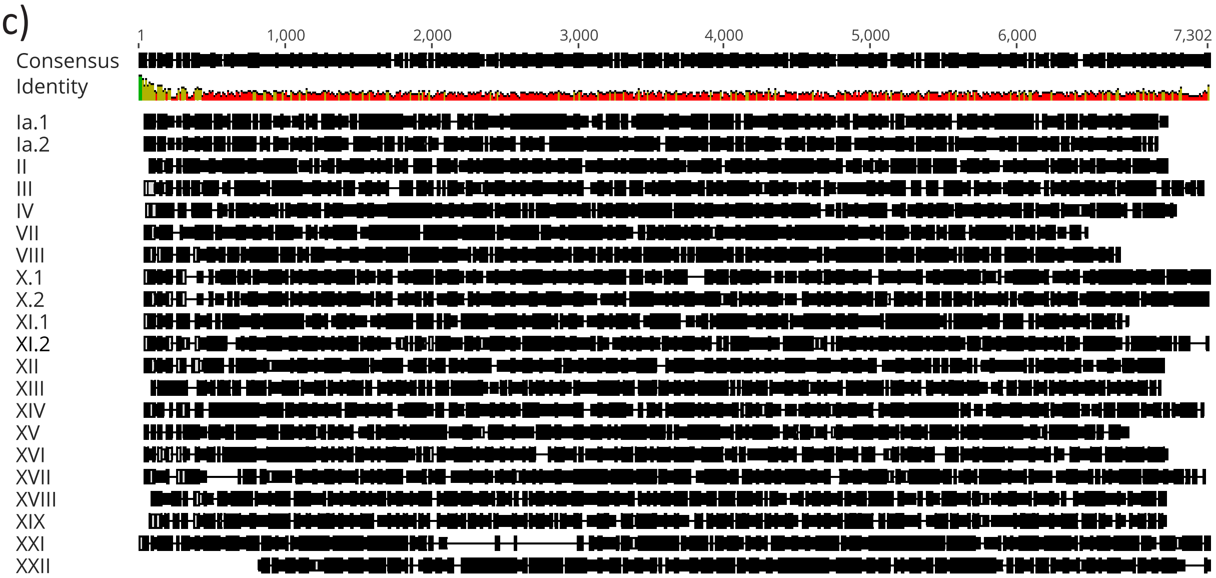

(d)

	Ia.1	Ia.2	II	III	IV	VII	VIII	X.1	X.2	XI.1	XI.2	XII	XIII	XIV	XV	XVI	XVII	XVIII	XIX	XXI	XXII
Ia.1		42.07%	25.80%	23.69%	23.07%	25.49%	26.04%	24.11%	22.84%	24.90%	24.53%	24.48%	21.55%	23.72%	26.13%	23.80%	23.35%	21.18%	21.24%	22.63%	25.70%
Ia.2	42.07%		24.86%	23.91%	23.57%	25.46%	25.57%	22.93%	23.19%	24.95%	23.90%	24.81%	22.10%	24.77%	26.69%	25.12%	24.11%	21.71%	21.72%	23.47%	25.90%
II	25.80%	24.86%		29.27%	24.38%	26.18%	28.70%	28.92%	29.32%	30.47%	30.31%	25.09%	22.90%	29.58%	27.75%	30.20%	24.50%	23.39%	23.94%	22.52%	26.85%
III	23.69%	23.91%	29.27%		24.84%	26.02%	28.93%	31.24%	30.22%	28.57%	31.33%	25.25%	22.33%	30.88%	27.59%	30.56%	23.61%	23.61%	23.36%	22.70%	24.95%
IV	23.07%	23.57%	24.38%	24.84%		26.07%	24.99%	23.72%	22.57%	24.18%	25.18%	36.44%	21.85%	24.24%	24.06%	23.87%	33.87%	21.88%	22.25%	21.63%	24.98%
VII	25.49%	25.46%	26.18%	26.02%	26.07%		26.63%	25.57%	24.53%	26.25%	25.65%	26.16%	22.90%	24.52%	26.42%	25.60%	24.71%	22.88%	22.93%	22.95%	26.88%
VIII	26.04%	25.57%	28.70%	28.93%	24.99%	26.63%		27.50%	27.54%	29.15%	29.35%	26.29%	23.71%	28.03%	29.10%	29.38%	26.02%	24.66%	24.18%	24.03%	27.62%
X.1	24.11%	22.93%	28.92%	31.24%	23.72%	25.57%	27.50%		45.09%	27.06%	31.20%	25.09%	24.03%	37.07%	25.89%	30.48%	23.51%	23.61%	23.54%	21.82%	24.14%
X.2	22.84%	23.19%	29.32%	30.22%	22.57%	24.53%	27.54%	45.09%		27.97%	31.15%	24.19%	22.62%	34.60%	26.49%	29.43%	22.92%	22.52%	22.33%	22.02%	24.78%
XI.1	24.90%	24.95%	30.47%	28.57%	24.18%	26.25%	29.15%	27.06%	27.97%		28.90%	25.18%	23.80%	28.03%	29.38%	31.16%	24.07%	23.31%	23.57%	23.59%	27.17%
XI.2	24.53%	23.90%	30.31%	31.33%	25.18%	25.65%	29.35%	31.20%	31.15%	28.90%		25.96%	21.76%	33.15%	27.28%	30.17%	24.33%	22.84%	23.57%	22.45%	25.22%
XII	24.48%	24.81%	25.09%	25.25%	36.44%	26.16%	26.29%	25.09%	24.19%	25.18%	25.96%		21.19%	25.35%	24.97%	25.21%	55.78%	23.00%	23.26%	22.84%	24.43%
XIII	21.55%	22.10%	22.90%	22.33%	21.85%	22.90%	23.71%	24.03%	22.62%	23.80%	21.76%	21.19%		22.51%	23.07%	23.06%	20.31%	33.20%	33.72%	19.80%	21.57%
XIV	23.72%	24.77%	29.58%	30.88%	24.24%	24.52%	28.03%	37.07%	34.60%	28.03%	33.15%	25.35%	22.51%		27.68%	29.36%	23.16%	22.74%	22.99%	23.99%	25.30%
XV	26.13%	26.69%	27.75%	27.59%	24.06%	26.42%	29.10%	25.89%	26.49%	29.38%	27.28%	24.97%	23.07%	27.68%		28.14%	24.28%	23.50%	22.79%	23.35%	27.22%
XVI	23.80%	25.12%	30.20%	30.56%	23.87%	25.60%	29.38%	30.48%	29.43%	31.16%	30.17%	25.21%	23.06%	29.36%	28.14%		23.62%	22.95%	23.57%	22.88%	25.39%
XVII	23.35%	24.11%	24.50%	23.61%	33.87%	24.71%	26.02%	23.51%	22.92%	24.07%	24.33%	55.78%	20.31%	23.16%	24.28%	23.62%		21.87%	21.46%	22.33%	25.26%
XVIII	21.18%	21.71%	23.39%	23.61%	21.88%	22.88%	24.66%	23.61%	22.52%	23.31%	22.84%	23.00%	33.20%	22.74%	23.50%	22.95%	21.87%		40.16%	20.07%	23.15%
XIX	21.24%	21.72%	23.94%	23.36%	22.25%	22.93%	24.18%	23.54%	22.33%	23.57%	23.57%	23.26%	33.72%	22.99%	22.79%	23.57%	21.46%	40.16%		20.28%	22.03%
XXI	22.63%	23.47%	22.52%	22.70%	21.63%	22.95%	24.03%	21.82%	22.02%	23.59%	22.45%	22.84%	19.80%	23.99%	23.35%	22.88%	22.33%	20.07%	20.28%		22.28%
XXII	25.70%	25.90%	26.85%	24.95%	24.98%	26.88%	27.62%	24.14%	24.78%	27.17%	25.22%	24.43%	21.57%	25.30%	27.22%	25.39%	25.26%	23.15%	22.03%	22.28%	

Figure S17 Comparison analysis of the 5 kb non-coding flanking sequences of *SFBB*s in *Malus* S_3-locus. (a) A snapshot showing the alignment of the 5kb upstream sequences of *SFBB*s. (b) Pairwise identity of the 5kb upstream sequences of *SFBB*s. (c) A snapshot showing the alignment of the 5kb downstream sequences of *SFBB*s. (d) Pairwise identity of the 5kb downstream sequences of *SFBB*s.

(a)

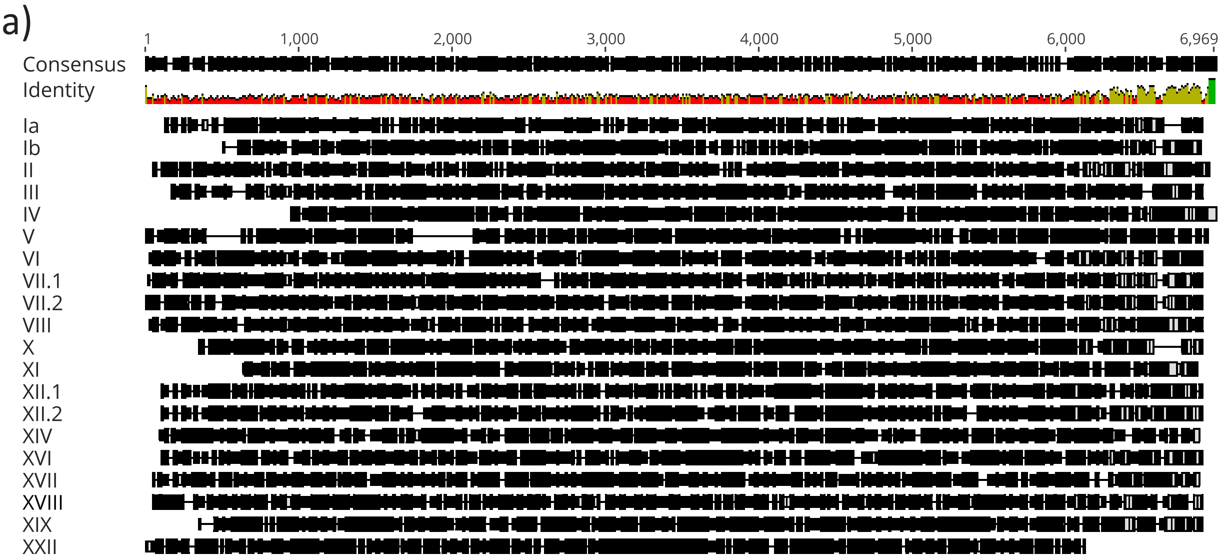

(b)

	Ia	Ib	II	III	IV	V	VI	VII.1	VII.2	VIII	X	XI	XII.1	XII.2	XIV	XVI	XVII	XVIII	XIX	XXII
Ia		24.19%	23.87%	23.51%	26.32%	22.35%	23.61%	24.42%	24.78%	23.83%	25.72%	25.37%	33.07%	32.59%	22.80%	31.63%	23.87%	24.12%	24.81%	24.69%
Ib	24.19%		28.63%	29.11%	29.96%	25.40%	28.23%	29.11%	29.76%	28.67%	28.16%	28.77%	23.39%	24.17%	27.84%	25.24%	27.55%	28.94%	29.64%	26.55%
II	23.87%	28.63%		30.14%	29.54%	23.12%	33.72%	33.37%	33.35%	32.62%	29.32%	30.33%	26.21%	26.30%	28.36%	26.75%	29.51%	33.14%	30.78%	25.70%
III	23.51%	29.11%	30.14%		28.57%	22.66%	28.63%	30.08%	30.59%	31.02%	27.54%	28.03%	24.26%	24.29%	27.53%	24.33%	28.10%	29.66%	29.30%	25.09%
IV	26.32%	29.96%	29.54%	28.57%		25.01%	29.43%	28.70%	30.13%	29.30%	28.38%	29.34%	27.49%	27.78%	28.80%	27.48%	28.82%	29.87%	30.12%	27.38%
V	22.35%	25.40%	23.12%	22.66%	25.01%		23.13%	23.43%	25.20%	23.22%	23.01%	24.92%	22.48%	22.62%	23.19%	22.98%	24.23%	24.72%	23.77%	23.54%
VI	23.61%	28.23%	33.72%	28.63%	29.43%	23.13%		37.68%	38.79%	32.56%	29.77%	29.82%	25.17%	25.25%	28.38%	27.43%	28.36%	35.33%	30.40%	25.10%
VII.1	24.42%	29.11%	33.37%	30.08%	28.70%	23.43%	37.68%		45.39%	33.44%	28.54%	28.86%	25.70%	25.31%	29.72%	26.84%	29.93%	35.62%	30.79%	24.59%
VII.2	24.78%	29.76%	33.35%	30.59%	30.13%	25.20%	38.79%	45.39%		32.87%	30.14%	29.35%	26.17%	25.75%	30.30%	27.76%	29.75%	36.41%	31.78%	25.34%
VIII	23.83%	28.67%	32.62%	31.02%	29.30%	23.22%	32.56%	33.44%	32.87%		27.82%	28.70%	24.77%	25.03%	28.21%	25.62%	28.97%	30.81%	31.03%	23.77%
X	25.72%	28.16%	29.32%	27.54%	28.38%	23.01%	29.77%	28.54%	30.14%	27.82%		28.38%	25.17%	25.50%	28.45%	26.00%	28.45%	30.37%	29.50%	27.00%
XI	25.37%	28.77%	30.33%	28.03%	29.34%	24.92%	29.82%	28.86%	29.35%	28.70%	28.38%		26.26%	26.03%	28.77%	26.44%	29.08%	28.04%	29.04%	27.34%
XII.1	33.07%	23.39%	26.21%	24.26%	27.49%	22.48%	25.17%	25.70%	26.17%	24.77%	25.17%	26.26%		44.03%	24.27%	36.14%	25.53%	26.91%	25.84%	23.98%
XII.2	32.59%	24.17%	26.30%	24.29%	27.78%	22.62%	25.25%	25.31%	25.75%	25.03%	25.50%	26.03%	44.03%		23.62%	35.33%	25.38%	26.08%	24.81%	23.44%
XIV	22.80%	27.84%	28.36%	27.53%	28.80%	23.19%	28.38%	29.72%	30.30%	28.21%	28.45%	28.77%	24.27%	23.62%		24.40%	51.62%	29.32%	31.63%	25.65%
XVI	31.63%	25.24%	26.75%	24.33%	27.48%	22.98%	27.43%	26.84%	27.76%	25.62%	26.00%	26.44%	36.14%	35.33%	24.40%		25.05%	26.56%	25.81%	23.59%
XVII	23.87%	27.55%	29.51%	28.10%	28.82%	24.23%	28.36%	29.93%	29.75%	28.97%	28.45%	29.08%	25.53%	25.38%	51.62%	25.05%		28.87%	30.63%	25.40%
XVIII	24.12%	28.94%	33.14%	29.66%	29.87%	24.72%	35.33%	35.62%	36.41%	30.81%	30.37%	28.04%	26.91%	26.08%	29.32%	26.56%	28.87%		30.65%	25.59%
XIX	24.81%	29.64%	30.78%	29.30%	30.12%	23.77%	30.40%	30.79%	31.78%	31.03%	29.50%	29.04%	25.84%	24.81%	31.63%	25.81%	30.63%	30.65%		25.73%
XXII	24.69%	26.55%	25.70%	25.09%	27.38%	23.54%	25.10%	24.59%	25.34%	23.77%	27.00%	27.34%	23.98%	23.44%	25.65%	23.59%	25.40%	25.59%	25.73%	

(c)

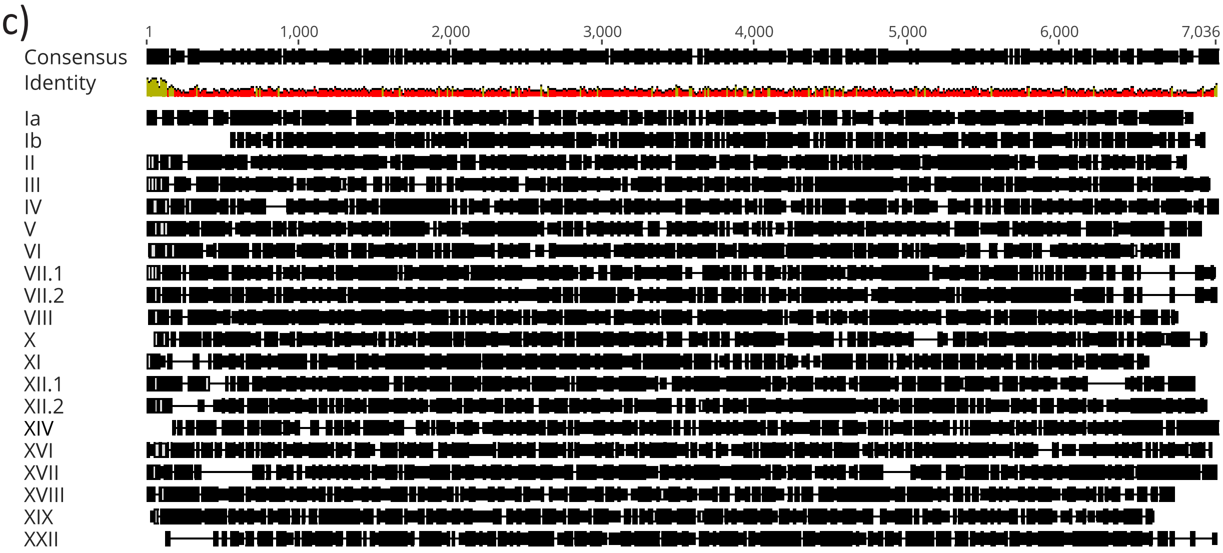

(d)

	Ia	Ib	II	III	IV	V	VI	VII.1	VII.2	VIII	X	XI	XII.1	XII.2	XIV	XVI	XVII	XVIII	XIX	XXII
Ia		20.55%	20.50%	20.65%	19.85%	21.00%	20.82%	21.80%	20.85%	20.57%	21.75%	20.03%	19.99%	28.61%	28.86%	29.53%	19.13%	22.11%	31.19%	27.80%
Ib	20.55%		25.11%	26.18%	24.82%	25.50%	24.25%	25.90%	25.15%	25.65%	25.38%	27.83%	25.32%	21.44%	20.26%	21.18%	24.86%	26.32%	21.11%	22.17%
II	20.50%	25.11%		31.71%	23.43%	29.86%	23.27%	24.31%	23.66%	24.95%	30.15%	24.76%	23.49%	20.01%	19.34%	21.32%	22.11%	32.03%	21.38%	20.36%
III	20.65%	26.18%	31.71%		23.51%	33.13%	23.76%	24.41%	23.78%	23.32%	29.71%	25.22%	22.88%	20.60%	20.14%	21.47%	23.31%	47.50%	20.41%	20.89%
IV	19.85%	24.82%	23.43%	23.51%		22.70%	30.86%	24.47%	24.18%	30.97%	23.97%	24.12%	34.98%	19.74%	19.64%	20.34%	32.75%	23.38%	20.95%	18.75%
V	21.00%	25.50%	29.86%	33.13%	22.70%		23.41%	23.80%	22.74%	22.64%	29.63%	23.35%	22.70%	20.21%	19.39%	21.31%	22.25%	34.72%	21.43%	19.77%
VI	20.82%	24.25%	23.27%	23.76%	30.86%	23.41%		22.98%	22.74%	29.38%	23.36%	24.33%	30.54%	20.82%	19.76%	21.42%	28.03%	24.60%	21.15%	20.37%
VII.1	21.80%	25.90%	24.31%	24.41%	24.47%	23.80%	22.98%		44.31%	25.16%	23.51%	24.25%	24.21%	19.57%	18.43%	19.42%	21.27%	25.76%	20.99%	19.79%
VII.2	20.85%	25.15%	23.66%	23.78%	24.18%	22.74%	22.74%	44.31%		24.94%	23.16%	25.47%	24.03%	20.12%	19.58%	19.45%	22.07%	25.32%	20.83%	19.76%
VIII	20.57%	25.65%	24.95%	23.32%	30.97%	22.64%	29.38%	25.16%	24.94%		23.84%	23.79%	32.43%	21.13%	20.83%	20.19%	30.56%	24.84%	21.64%	19.75%
X	21.75%	25.38%	30.15%	29.71%	23.97%	29.63%	23.36%	23.51%	23.16%	23.84%		23.78%	23.24%	19.38%	19.61%	19.88%	23.14%	30.72%	21.62%	19.45%
XI	20.03%	27.83%	24.76%	25.22%	24.12%	23.35%	24.33%	24.25%	25.47%	23.79%	23.78%		25.24%	20.93%	19.23%	19.55%	24.59%	24.79%	20.24%	21.47%
XII.1	19.99%	25.32%	23.49%	22.88%	34.98%	22.70%	30.54%	24.21%	24.03%	32.43%	23.24%	25.24%		21.45%	18.67%	20.36%	54.00%	24.10%	21.90%	18.14%
XII.2	28.61%	21.44%	20.01%	20.60%	19.74%	20.21%	20.82%	19.57%	20.12%	21.13%	19.38%	20.93%	21.45%		28.69%	29.90%	20.17%	20.48%	30.91%	27.35%
XIV	28.86%	20.26%	19.34%	20.14%	19.64%	19.39%	19.76%	18.43%	19.58%	20.83%	19.61%	19.23%	18.67%	28.69%		31.22%	18.97%	20.00%	44.69%	25.88%
XVI	29.53%	21.18%	21.32%	21.47%	20.34%	21.31%	21.42%	19.42%	19.45%	20.19%	19.88%	19.55%	20.36%	29.90%	31.22%		19.44%	21.34%	36.68%	25.88%
XVII	19.13%	24.86%	22.11%	23.31%	32.75%	22.25%	28.03%	21.27%	22.07%	30.56%	23.14%	24.59%	54.00%	20.17%	18.97%	19.44%		23.12%	19.28%	18.69%
XVIII	22.11%	26.32%	32.03%	47.50%	23.38%	34.72%	24.60%	25.76%	25.32%	24.84%	30.72%	24.79%	24.10%	20.48%	20.00%	21.34%	23.12%		21.93%	21.19%
XIX	31.19%	21.11%	21.38%	20.41%	20.95%	21.43%	21.15%	20.99%	20.83%	21.64%	21.62%	20.24%	21.90%	30.91%	44.69%	36.68%	19.28%	21.93%		27.71%
XXII	27.80%	22.17%	20.36%	20.89%	18.75%	19.77%	20.37%	19.79%	19.76%	19.75%	19.45%	21.47%	18.14%	27.35%	25.88%	25.88%	18.69%	21.19%	27.71%	

Figure S18 Comparison analysis of the 5 kb non-coding flanking sequences of *SFBB*s in *Malus* S_5-locus. (a) A snapshot showing the alignment of the 5kb upstream sequences of *SFBB*s. (b) Pairwise identity of the 5kb upstream sequences of *SFBB*s. (c) A snapshot showing the alignment of the 5kb downstream sequences of *SFBB*s. (d) Pairwise identity of the 5kb downstream sequences of *SFBB*s.

(a)

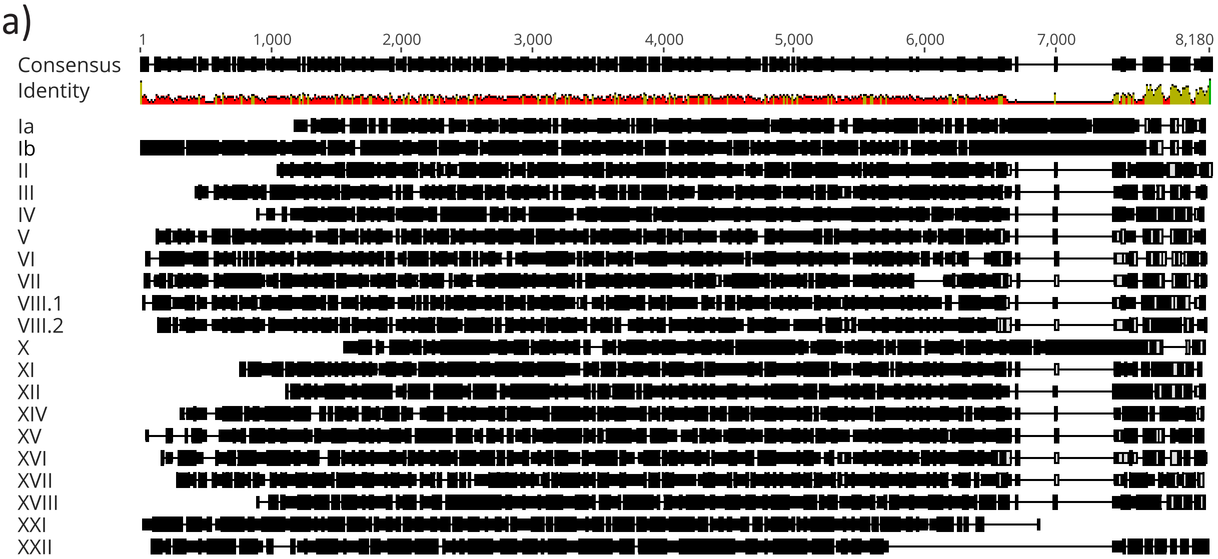

(b)

	Ia	Ib	II	III	IV	V	VI	VII	VIII.1	VIII.2	X	XI	XII	XIV	XV	XVI	XVII	XVIII	XXI	XXII
Ia		41.13%	22.96%	20.89%	22.75%	20.68%	20.43%	21.49%	21.37%	21.88%	27.57%	22.32%	22.78%	20.76%	20.92%	21.92%	21.41%	21.63%	23.81%	20.06%
Ib	41.13%		24.78%	23.63%	24.31%	22.70%	21.76%	24.00%	23.87%	23.23%	30.29%	24.69%	24.79%	23.97%	22.39%	23.45%	23.23%	23.57%	27.76%	22.84%
II	22.96%	24.78%		30.24%	28.99%	28.73%	28.11%	29.70%	30.24%	30.47%	23.93%	30.24%	32.04%	29.56%	29.09%	29.57%	31.38%	30.67%	25.05%	23.52%
III	20.89%	23.63%	30.24%		27.20%	28.97%	28.48%	29.84%	29.37%	30.47%	21.29%	30.08%	29.34%	27.32%	29.63%	29.43%	27.45%	28.65%	24.93%	20.94%
IV	22.75%	24.31%	28.99%	27.20%		27.08%	26.59%	27.67%	27.84%	28.91%	22.21%	29.17%	29.89%	26.28%	28.01%	26.83%	27.13%	29.74%	25.33%	23.03%
V	20.68%	22.70%	28.73%	28.97%	27.08%		27.95%	29.45%	37.04%	36.22%	20.87%	29.62%	27.84%	24.33%	26.67%	28.99%	25.28%	26.40%	20.55%	18.93%
VI	20.43%	21.76%	28.11%	28.48%	26.59%	27.95%		41.85%	27.41%	28.43%	19.57%	28.89%	27.55%	24.16%	31.30%	34.70%	27.24%	26.60%	21.83%	20.31%
VII	21.49%	24.00%	29.70%	29.84%	27.67%	29.45%	41.85%		30.72%	30.39%	20.89%	30.13%	28.21%	25.02%	31.96%	37.58%	26.95%	26.95%	22.47%	21.65%
VIII.1	21.37%	23.87%	30.24%	29.37%	27.84%	37.04%	27.41%	30.72%		46.69%	21.28%	30.54%	28.78%	25.97%	27.35%	29.99%	26.24%	27.44%	22.64%	20.13%
VIII.2	21.88%	23.23%	30.47%	30.47%	28.91%	36.22%	28.43%	30.39%	46.69%		21.19%	31.43%	29.80%	26.87%	27.88%	29.79%	27.78%	27.92%	22.99%	20.46%
X	27.57%	30.29%	23.93%	21.29%	22.21%	20.87%	19.57%	20.89%	21.28%	21.19%		23.22%	23.74%	20.71%	21.87%	21.36%	20.36%	23.30%	25.12%	19.49%
XI	22.32%	24.69%	30.24%	30.08%	29.17%	29.62%	28.89%	30.13%	30.54%	31.43%	23.22%		30.67%	27.18%	30.51%	30.45%	28.26%	28.24%	25.62%	21.22%
XII	22.78%	24.79%	32.04%	29.34%	29.89%	27.84%	27.55%	28.21%	28.78%	29.80%	23.74%	30.67%		28.27%	28.94%	29.78%	30.22%	28.80%	24.50%	23.05%
XIV	20.76%	23.97%	29.56%	27.32%	26.28%	24.33%	24.16%	25.02%	25.97%	26.87%	20.71%	27.18%	28.27%		25.71%	24.83%	52.01%	26.50%	24.87%	21.67%
XV	20.92%	22.39%	29.09%	29.63%	28.01%	26.67%	31.30%	31.96%	27.35%	27.88%	21.87%	30.51%	28.94%	25.71%		33.62%	26.07%	28.49%	22.18%	20.84%
XVI	21.92%	23.45%	29.57%	29.43%	26.83%	28.99%	34.70%	37.58%	29.99%	29.79%	21.36%	30.45%	29.78%	24.83%	33.62%		26.66%	26.55%	22.85%	20.01%
XVII	21.41%	23.23%	31.38%	27.45%	27.13%	25.28%	27.24%	26.95%	26.24%	27.78%	20.36%	28.26%	30.22%	52.01%	26.07%	26.66%		27.40%	24.77%	22.58%
XVIII	21.63%	23.57%	30.67%	28.65%	29.74%	26.40%	26.60%	26.95%	27.44%	27.92%	23.30%	28.24%	28.80%	26.50%	28.49%	26.55%	27.40%		24.26%	21.42%
XXI	23.81%	27.76%	25.05%	24.93%	25.33%	20.55%	21.83%	22.47%	22.64%	22.99%	25.12%	25.62%	24.50%	24.87%	22.18%	22.85%	24.77%	24.26%		23.79%
XXII	20.06%	22.84%	23.52%	20.94%	23.03%	18.93%	20.31%	21.65%	20.13%	20.46%	19.49%	21.22%	23.05%	21.67%	20.84%	20.01%	22.58%	21.42%	23.79%	

(c)

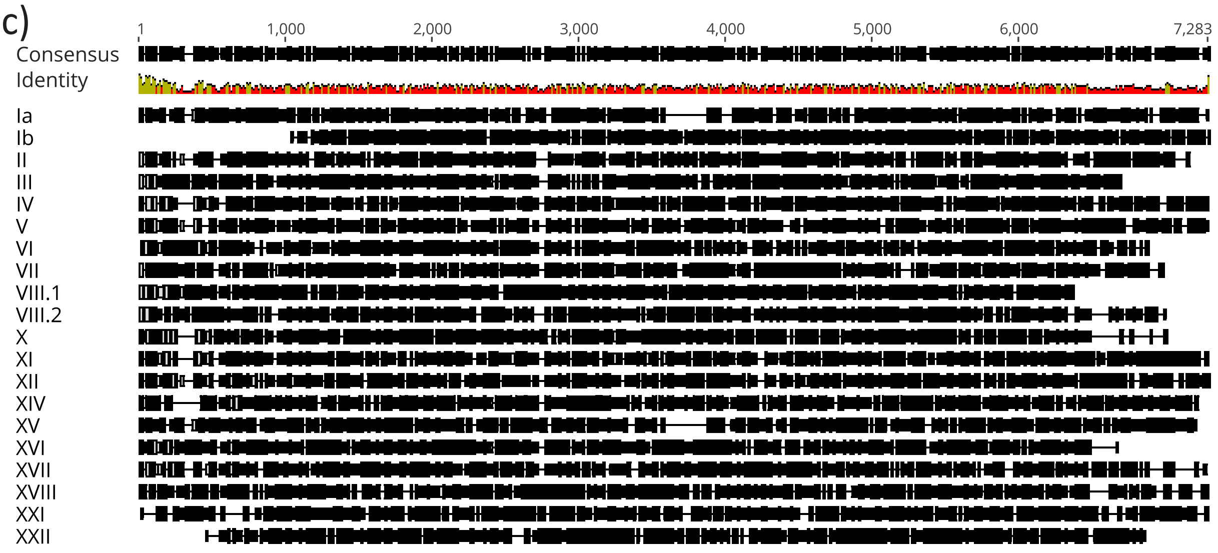

(d)

	Ia	Ib	II	III	IV	V	VI	VII	VIII.1	VIII.2	X	XI	XII	XIV	XV	XVI	XVII	XVIII	XXI	XXII
Ia		23.20%	21.80%	22.65%	22.26%	21.29%	21.80%	21.79%	22.18%	21.82%	21.82%	22.23%	21.23%	22.29%	39.38%	21.75%	22.13%	20.81%	20.58%	23.76%
Ib	23.20%		24.17%	25.89%	25.14%	24.28%	25.82%	25.57%	26.37%	24.67%	24.15%	25.66%	24.66%	23.90%	23.59%	26.27%	24.12%	23.13%	24.27%	26.43%
II	21.80%	24.17%		28.31%	31.17%	31.30%	29.15%	28.88%	25.95%	26.31%	24.88%	31.24%	30.41%	30.33%	23.74%	28.55%	29.80%	24.32%	21.08%	24.87%
III	22.65%	25.89%	28.31%		28.82%	29.01%	30.21%	30.77%	27.22%	26.69%	26.62%	29.30%	29.52%	28.12%	23.61%	29.95%	29.38%	25.31%	21.96%	25.84%
IV	22.26%	25.14%	31.17%	28.82%		36.23%	30.37%	28.24%	27.25%	26.34%	26.83%	41.60%	35.30%	34.65%	23.74%	29.18%	29.01%	23.57%	21.25%	25.80%
V	21.29%	24.28%	31.30%	29.01%	36.23%		29.39%	28.00%	26.58%	24.68%	25.63%	35.71%	33.45%	31.53%	23.09%	29.16%	28.57%	23.38%	21.73%	25.09%
VI	21.80%	25.82%	29.15%	30.21%	30.37%	29.39%		28.88%	27.71%	25.22%	26.29%	28.98%	29.27%	29.38%	22.50%	29.63%	30.01%	23.85%	23.17%	25.68%
VII	21.79%	25.57%	28.88%	30.77%	28.24%	28.00%	28.88%		25.68%	26.41%	24.79%	27.67%	28.32%	27.96%	23.50%	28.67%	27.73%	23.10%	21.36%	24.06%
VIII.1	22.18%	26.37%	25.95%	27.22%	27.25%	26.58%	27.71%	25.68%		26.18%	26.12%	26.42%	26.79%	26.34%	23.67%	28.22%	26.38%	24.88%	42.26%	25.64%
VIII.2	21.82%	24.67%	26.31%	26.69%	26.34%	24.68%	25.22%	26.41%	26.18%		27.51%	24.42%	25.04%	24.44%	22.24%	27.50%	25.85%	41.43%	20.70%	25.86%
X	21.82%	24.15%	24.88%	26.62%	26.83%	25.63%	26.29%	24.79%	26.12%	27.51%		25.73%	26.21%	26.52%	21.64%	28.46%	26.01%	24.77%	20.00%	24.17%
XI	22.23%	25.66%	31.24%	29.30%	41.60%	35.71%	28.98%	27.67%	26.42%	24.42%	25.73%		34.87%	32.82%	23.38%	28.73%	28.90%	22.06%	21.34%	25.26%
XII	21.23%	24.66%	30.41%	29.52%	35.30%	33.45%	29.27%	28.32%	26.79%	25.04%	26.21%	34.87%		31.46%	22.57%	28.66%	29.50%	23.02%	20.56%	24.39%
XIV	22.29%	23.90%	30.33%	28.12%	34.65%	31.53%	29.38%	27.96%	26.34%	24.44%	26.52%	32.82%	31.46%		22.63%	28.40%	29.48%	23.30%	20.83%	25.55%
XV	39.38%	23.59%	23.74%	23.61%	23.74%	23.09%	22.50%	23.50%	23.67%	22.24%	21.64%	23.38%	22.57%	22.63%		23.08%	22.94%	21.28%	20.77%	23.33%
XVI	21.75%	26.27%	28.55%	29.95%	29.18%	29.16%	29.63%	28.67%	28.22%	27.50%	28.46%	28.73%	28.66%	28.40%	23.08%		28.77%	24.54%	22.84%	25.86%
XVII	22.13%	24.12%	29.80%	29.38%	29.01%	28.57%	30.01%	27.73%	26.38%	25.85%	26.01%	28.90%	29.50%	29.48%	22.94%	28.77%		24.16%	20.72%	25.94%
XVIII	20.81%	23.13%	24.32%	25.31%	23.57%	23.38%	23.85%	23.10%	24.88%	41.43%	24.77%	22.06%	23.02%	23.30%	21.28%	24.54%	24.16%		21.02%	24.72%
XXI	20.58%	24.27%	21.08%	21.96%	21.25%	21.73%	23.17%	21.36%	42.26%	20.70%	20.00%	21.34%	20.56%	20.83%	20.77%	22.84%	20.72%	21.02%		23.90%
XXII	23.76%	26.43%	24.87%	25.84%	25.80%	25.09%	25.68%	24.06%	25.64%	25.86%	24.17%	25.26%	24.39%	25.55%	23.33%	25.86%	25.94%	24.72%	23.90%	

Figure S19 Comparison analysis of the 5 kb non-coding flanking sequences of *SFBB*s in *Malus* S_9-locus. (a) A snapshot showing the alignment of the 5kb upstream sequences of *SFBB*s. (b) Pairwise identity of the 5kb upstream sequences of *SFBB*s. (c) A snapshot showing the alignment of the 5kb downstream sequences of *SFBB*s. (d) Pairwise identity of the 5kb downstream sequences of *SFBB*s.
