# Supplementary material for: Long-read genome sequencing reveals the sequence characteristics of pear self-incompatibility locus
Source: Mol Hortic. 2025 Mar 1;5:13. doi: 10.1186/s43897-024-00132-0 (PMC11871771; doi:10.1186/s43897-024-00132-0)
Supplement: Supplementary file 1 — Supplementary Material 1: Table S1 Comparison of ‘Yali’ genome with previously published assemblies of Pyrus and Malus species. Table S2 Annotation of the repeats in ‘Yali’ genome. Table S3 Annotation of the non-coding RNAs in ‘Dananguo’ and 'Yali' genomes. Table S4 Identification of the F-box genes in Pyrus, Malus and Prunus S-loci. Table S5 Function annotation of the predicted genes in S-loci. Table S6 Sequence similarity (%) among Pyrus and Malus SFBB genes. Table S7 Sequence similarity (%) among Prunus SFB and SLF genes. Table S8 Sequence similarity among Prunus SFB and SLF genes. Table S9 Sequence similarity (%) among Pyrus and Malus S-RNase genes. Table S10 Prediction of gene duplication events of Pyrus and Malus SFBB genes. Table S11 Sequence similarity of the non-coding flanking sequences of SFBBs in Pyrus and Malus S-loci. Table S12 Analysis of number and length of LTR retrotransposon in different S-loci. Table S13 Identification of the LTR retrotransposon in different S-loci. Table S14 RPKM values of the genes commonly existed in the tested S-loci. Table S15 Sequence similarity (%) among the reported Pyrus S-RNase genes. Table S16 The accession numbers of S-RNase and S-locus F-box genes in Pyrus, Malus, and Prunus.Table S17 Primers used in this study. Figure S1 Isolation of the conserved F-box motif in the reported S-locus F-box proteins in Pyrus and Malus. The accession numbers of these F-box proteins were listed in Table S13. Figure S2 Phylogenetic classifications of S-locus F-box genes in Prunus. The S-locus F-box (SLF/SFB) proteins in Prunus comprised by 12 groups, SLF1→SLF11 and SFB. Each group were highlighted with different colors. Figure S3 Phylogenetic analysis of the F-box genes identified from this and previous studies. Cycles with black color present the F-box genes identified from previous study (Huang et al., 2023). The rates (%) of different types of gene duplication events (dispersed, proximal, tandem and transposed) of the S-locus F-box ge [file 43897_2024_132_MOESM1_ESM.zip › Supplementary Figures S20 to S30.pdf]

(a)

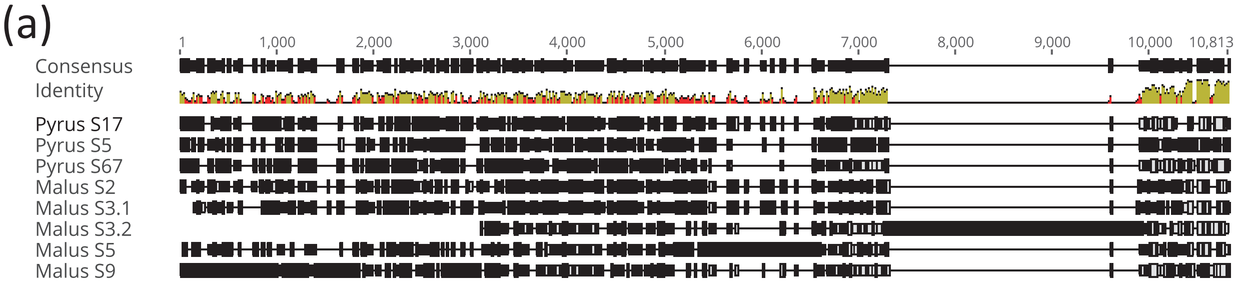

(b)

	Pyrus S17	Pyrus S5	Pyrus S67	Malus S2	Malus S3.1	Malus S3.2	Malus S5	Malus S9
Pyrus S17		32.49%	35.64%	35.85%	36.43%	24.47%	31.96%	34.32%
Pyrus S5	32.49%		34.88%	33.24%	33.49%	22.32%	31.42%	31.84%
Pyrus S67	35.64%	34.88%		34.67%	34.96%	28.88%	34.48%	38.00%
Malus S2	35.85%	33.24%	34.67%		99.96%	20.95%	32.43%	30.95%
Malus S3.1	36.43%	33.49%	34.96%	99.96%		20.95%	32.60%	31.31%
Malus S3.2	24.47%	22.32%	28.88%	20.95%	20.95%		28.68%	43.17%
Malus S5	31.96%	31.42%	34.48%	32.43%	32.60%	28.68%		40.42%
Malus S9	34.32%	31.84%	38.00%	30.95%	31.31%	43.17%	40.42%	

(c)

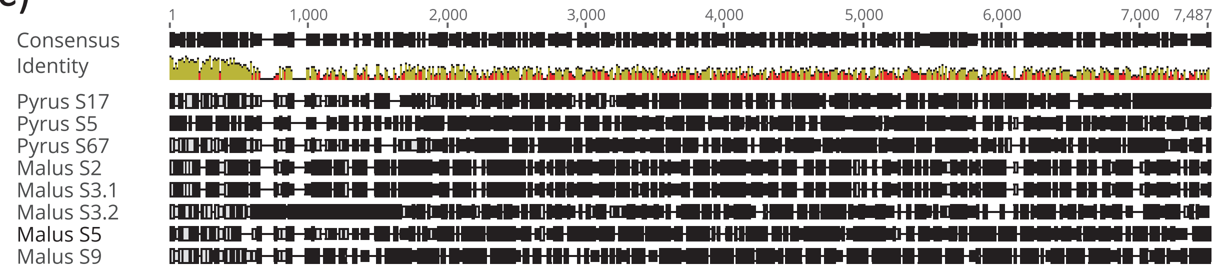

(d)

	Pyrus S17	Pyrus S5	Pyrus S67	Malus S2	Malus S3.1	Malus S3.2	Malus S5	Malus S9
Pyrus S17		33.06%	40.37%	35.18%	35.18%	42.65%	38.51%	35.39%
Pyrus S5	33.06%		30.60%	28.94%	28.94%	28.10%	34.76%	28.89%
Pyrus S67	40.37%	30.60%		32.81%	32.81%	34.75%	35.26%	33.13%
Malus S2	35.18%	28.94%	32.81%		100%	32.22%	34.04%	32.39%
Malus S3.1	35.18%	28.94%	32.81%	100%		32.22%	34.04%	32.39%
Malus S3.2	42.65%	28.10%	34.75%	32.22%	32.22%		32.04%	30.87%
Malus S5	38.51%	34.76%	35.26%	34.04%	34.04%	32.04%		33.72%
Malus S9	35.39%	28.89%	33.13%	32.39%	32.39%	30.87%	33.72%	

Figure S20 Comparison analysis of the 5 kb non-coding flanking sequences of *SFBB*s in group Ia. (a) A snapshot showing the alignment of the 5kb upstream sequences of *SFBB*s. (b) Pairwise identity of the 5kb upstream sequences of *SFBB*s. (c) A snapshot showing the alignment of the 5kb downstream sequences of *SFBB*s. (d) Pairwise identity of the 5kb downstream sequences of *SFBB*s. Malus S3.1 and S3.2 represent the *Malus SFBB.Ia.1-*S_3 and *SFBB.Ia.2-*S_3, respectively.

(a)

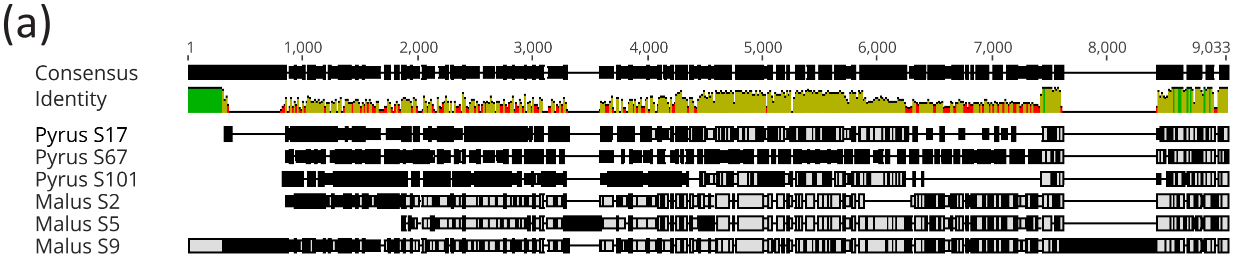

(b)

	Pyrus S17	Pyrus S67	Pyrus S101	Malus S2	Malus S5	Malus S9
Pyrus S17		34.43%	52.09%	52.04%	56.99%	47.15%
Pyrus S67	34.43%		30.01%	44.81%	44.04%	39.57%
Pyrus S101	52.09%	30.01%		45.14%	49.66%	43.62%
Malus S2	52.04%	44.81%	45.14%		85.14%	76.68%
Malus S5	56.99%	44.04%	49.66%	85.14%		78.54%
Malus S9	47.15%	39.57%	43.62%	76.68%	78.54%	

(c)

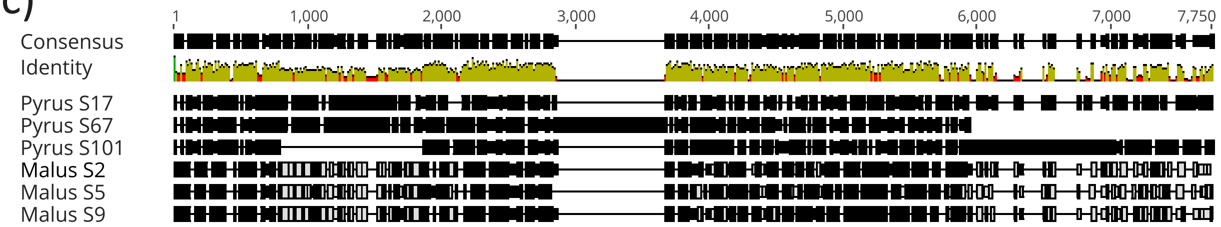

(d)

	Pyrus S17	Pyrus S67	Pyrus S101	Malus S2	Malus S5	Malus S9
Pyrus S17		63.95%	47.53%	37.23%	37.17%	37.39%
Pyrus S67	63.95%		57.74%	33.10%	33.23%	33.33%
Pyrus S101	47.53%	57.74%		29.34%	29.17%	29.34%
Malus S2	37.23%	33.10%	29.34%		96.65%	96.88%
Malus S5	37.17%	33.23%	29.17%	96.65%		96.82%
Malus S9	37.39%	33.33%	29.34%	96.88%	96.82%	

Figure S21 Comparison analysis of the 5 kb non-coding flanking sequences of *SFBB*s in group Ib. (a) A snapshot showing the alignment of the 5kb upstream sequences of *SFBB*s. (b) Pairwise identity of the 5kb upstream sequences of *SFBB*s. (c) A snapshot showing the alignment of the 5kb downstream sequences of *SFBB*s. (d) Pairwise identity of the 5kb downstream sequences of *SFBB*s.

(a)

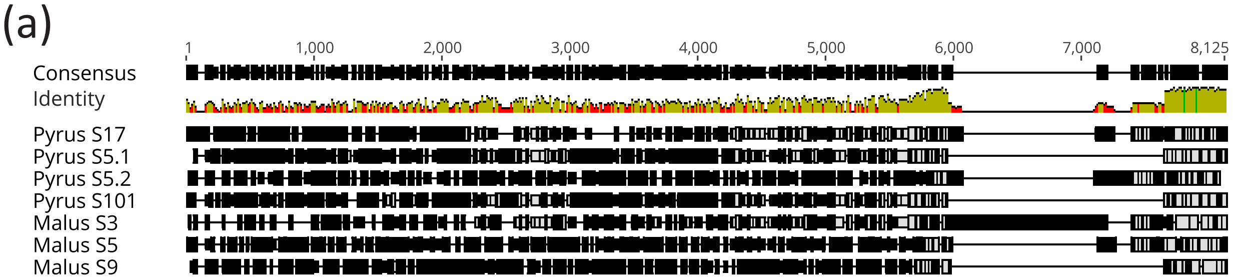

(b)

	Pyrus S17	Pyrus S5.1	Pyrus S5.2	Pyrus S101	Malus S3	Malus S5	Malus S9
Pyrus S17		45.17%	37.11%	45.69%	46.74%	38.52%	32.57%
Pyrus S5.1	45.17%		33.45%	94.98%	37.92%	36.51%	40.09%
Pyrus S5.2	37.11%	33.45%		33.32%	33.08%	39.03%	33.08%
Pyrus S101	45.69%	94.98%	33.32%		38.20%	36.67%	40.32%
Malus S3	46.74%	37.92%	33.08%	38.20%		31.50%	28.28%
Malus S5	38.52%	36.51%	39.03%	36.67%	31.50%		35.10%
Malus S9	32.57%	40.09%	33.08%	40.32%	28.28%	35.10%	

(c)

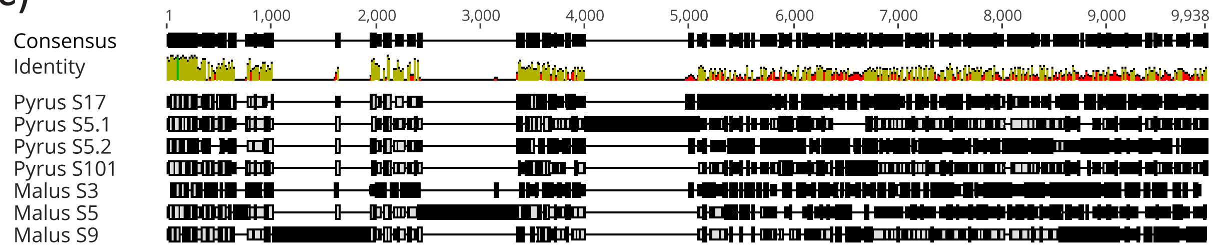

(d)

	Pyrus S17	Pyrus S5.1	Pyrus S5.2	Pyrus S101	Malus S3	Malus S5	Malus S9
Pyrus S17		34.51%	37.28%	41.97%	32.40%	32.47%	33.21%
Pyrus S5.1	34.51%		33.19%	64.42%	27.25%	37.18%	43.29%
Pyrus S5.2	37.28%	33.19%		40.78%	28.68%	32.63%	34.64%
Pyrus S101	41.97%	64.42%	40.78%		34.68%	44.70%	56.41%
Malus S3	32.40%	27.25%	28.68%	34.68%		26.75%	28.84%
Malus S5	32.47%	37.18%	32.63%	44.70%	26.75%		34.36%
Malus S9	33.21%	43.29%	34.64%	56.41%	28.84%	34.36%	

Figure S22 Comparison analysis of the 5 kb non-coding flanking sequences of *SFBB*s in group II. (a) A snapshot showing the alignment of the 5kb upstream sequences of *SFBB*s. (b) Pairwise identity of the 5kb upstream sequences of *SFBB*s. (c) A snapshot showing the alignment of the 5kb downstream sequences of *SFBB*s. (d) Pairwise identity of the 5kb downstream sequences of *SFBB*s. Pyrus S5.1 and S5.2 represent the *Pyrus SFBB.II.1-S_5* and *SFBB.II.2-S_5*, respectively.

(a)

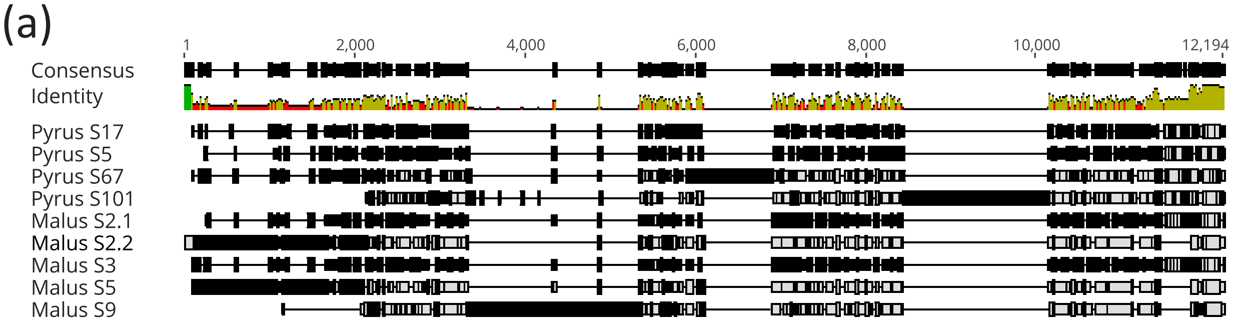

(b)

	Pyrus S17	Pyrus S5	Pyrus S67	Pyrus S101	Malus S2.1	Malus S2.2	Malus S3	Malus S5	Malus S9
Pyrus S17		32.27%	32.26%	28.93%	37.12%	29.94%	36.78%	29.94%	23.81%
Pyrus S5	32.27%		33.40%	29.08%	38.78%	29.08%	38.74%	29.08%	23.05%
Pyrus S67	32.26%	33.40%		45.73%	35.67%	51.12%	35.45%	51.12%	39.52%
Pyrus S101	28.93%	29.08%	45.73%		30.17%	51.11%	30.25%	51.11%	37.96%
Malus S2.1	37.12%	38.78%	35.67%	30.17%		31.84%	99.64%	31.84%	24.65%
Malus S2.2	29.94%	29.08%	51.12%	51.11%	31.84%		31.83%	100%	50.63%
Malus S3	36.78%	38.74%	35.45%	30.25%	99.64%	31.83%		31.83%	24.69%
Malus S5	29.94%	29.08%	51.12%	51.11%	31.84%	100%	31.83%		50.63%
Malus S9	23.81%	23.05%	39.52%	37.96%	24.65%	50.63%	24.69%	50.63%	

(c)

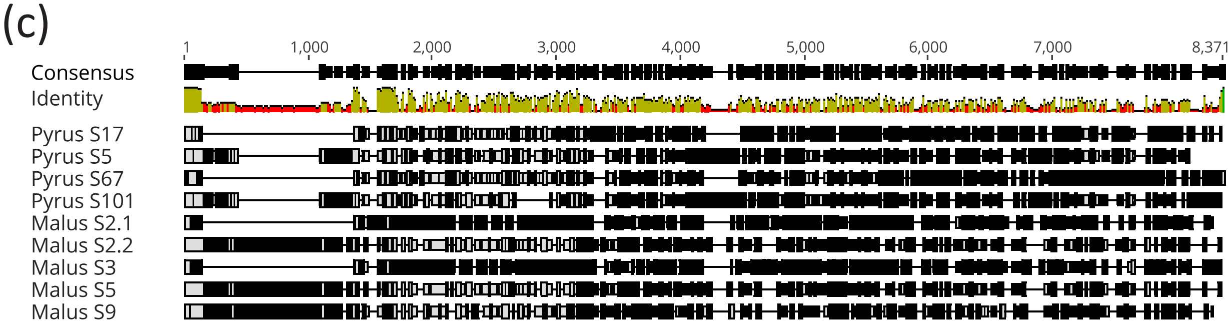

(d)

	Pyrus S17	Pyrus S5	Pyrus S67	Pyrus S101	Malus S2.1	Malus S2.2	Malus S3	Malus S5	Malus S9
Pyrus S17		41.30%	46.30%	37.28%	32.79%	33.80%	32.91%	33.80%	33.47%
Pyrus S5	41.30%		55.62%	90.23%	33.53%	46.40%	33.97%	46.40%	45.94%
Pyrus S67	46.30%	55.62%		51.95%	35.45%	36.85%	35.91%	36.85%	36.73%
Pyrus S101	37.28%	90.23%	51.95%		31.45%	42.01%	31.86%	42.01%	42.02%
Malus S2.1	32.79%	33.53%	35.45%	31.45%		27.84%	98.37%	27.84%	27.90%
Malus S2.2	33.80%	46.40%	36.85%	42.01%	27.84%		28.00%	100%	96.65%
Malus S3	32.91%	33.97%	35.91%	31.86%	98.37%	28.00%		28.00%	28.25%
Malus S5	33.80%	46.40%	36.85%	42.01%	27.84%	100%	28.00%		96.65%
Malus S9	33.47%	45.94%	36.73%	42.02%	27.90%	96.65%	28.25%	96.65%	

Figure S23 Comparison analysis of the 5 kb non-coding flanking sequences of *SFBB*s in group III. (a) A snapshot showing the alignment of the 5kb upstream sequences of *SFBB*s. (b) Pairwise identity of the 5kb upstream sequences of *SFBB*s. (c) A snapshot showing the alignment of the 5kb downstream sequences of *SFBB*s. (d) Pairwise identity of the 5kb downstream sequences of *SFBB*s. Malus S2.1 and S2.2 represent the *Malus SFBB.III.1-S_2* and *SFBB.III.2-S_2*, respectively.

(a)

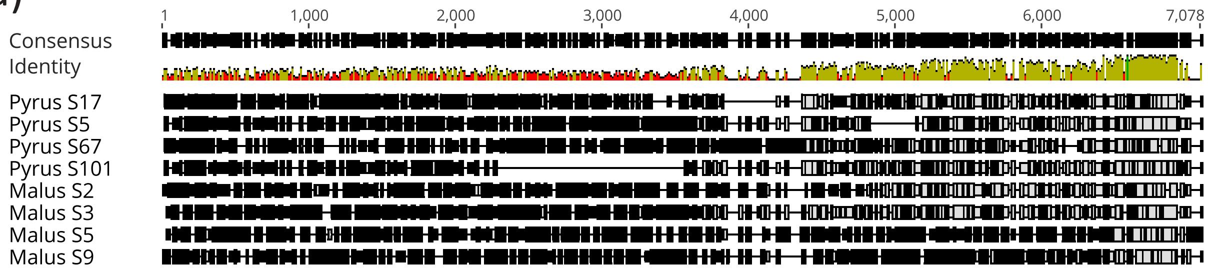

(b)

	Pyrus S17	Pyrus S5	Pyrus S67	Pyrus S101	Malus S2	Malus S3	Malus S5	Malus S9
Pyrus S17		49.38%	45.63%	50.85%	50.36%	73.77%	36.91%	40.74%
Pyrus S5	49.38%		46.22%	62.83%	46.69%	52.25%	34.29%	40.45%
Pyrus S67	45.63%	46.22%		45.47%	45.14%	46.66%	31.44%	37.36%
Pyrus S101	50.85%	62.83%	45.47%		46.67%	51.74%	32.42%	39.38%
Malus S2	50.36%	46.69%	45.14%	46.67%		51.53%	36.45%	40.11%
Malus S3	73.77%	52.25%	46.66%	51.74%	51.53%		37.63%	42.51%
Malus S5	36.91%	34.29%	31.44%	32.42%	36.45%	37.63%		30.61%
Malus S9	40.74%	40.45%	37.36%	39.38%	40.11%	42.51%	30.61%	

(c)

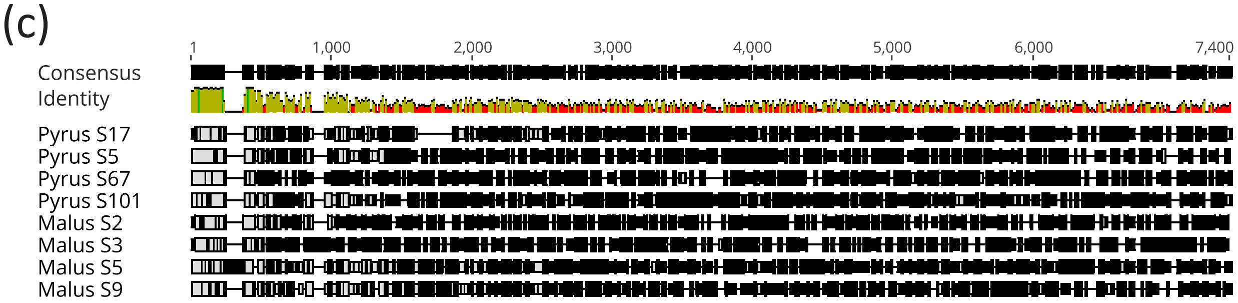

(d)

	Pyrus S17	Pyrus S5	Pyrus S67	Pyrus S101	Malus S2	Malus S3	Malus S5	Malus S9
Pyrus S17		36.73%	33.86%	40.02%	36.29%	30.99%	44.32%	51.38%
Pyrus S5	36.73%		32.68%	39.69%	32.61%	26.80%	37.12%	39.27%
Pyrus S67	33.86%	32.68%		34.95%	30.05%	27.12%	35.15%	37.85%
Pyrus S101	40.02%	39.69%	34.95%		35.55%	31.32%	42.88%	48.55%
Malus S2	36.29%	32.61%	30.05%	35.55%		29.54%	34.90%	36.35%
Malus S3	30.99%	26.80%	27.12%	31.32%	29.54%		31.13%	30.75%
Malus S5	44.32%	37.12%	35.15%	42.88%	34.90%	31.13%		53.14%
Malus S9	51.38%	39.27%	37.85%	48.55%	36.35%	30.75%	53.14%	

Figure S24 Comparison analysis of the 5 kb non-coding flanking sequences of *SFBB*s in group IV. (a) A snapshot showing the alignment of the 5kb upstream sequences of *SFBB*s. (b) Pairwise identity of the 5kb upstream sequences of *SFBB*s. (c) A snapshot showing the alignment of the 5kb downstream sequences of *SFBB*s. (d) Pairwise identity of the 5kb downstream sequences of *SFBB*s.

(a)

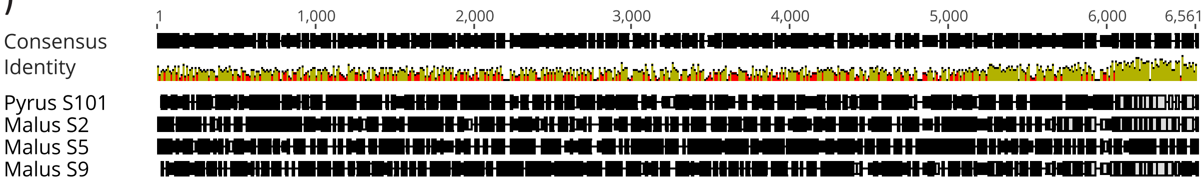

(b)

	Pyrus S101	Malus S2	Malus S5	Malus S9
Pyrus S101		40.03%	31.08%	38.91%
Malus S2	40.03%		34.36%	45.70%
Malus S5	31.08%	34.36%		32.96%
Malus S9	38.91%	45.70%	32.96%	

(c)

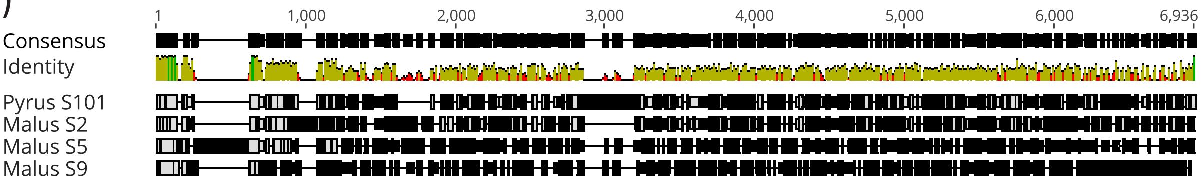

(d)

	Pyrus S101	Malus S2	Malus S5	Malus S9
Pyrus S101		75.39%	40.64%	37.97%
Malus S2	75.39%		41.87%	37.85%
Malus S5	40.64%	41.87%		33.55%
Malus S9	37.97%	37.85%	33.55%	

Figure S25 Comparison analysis of the 5 kb non-coding flanking sequences of *SFBB*s in group V. (a) A snapshot showing the alignment of the 5kb upstream sequences of *SFBB*s. (b) Pairwise identity of the 5kb upstream sequences of *SFBB*s. (c) A snapshot showing the alignment of the 5kb downstream sequences of *SFBB*s. (d) Pairwise identity of the 5kb downstream sequences of *SFBB*s.

(a)

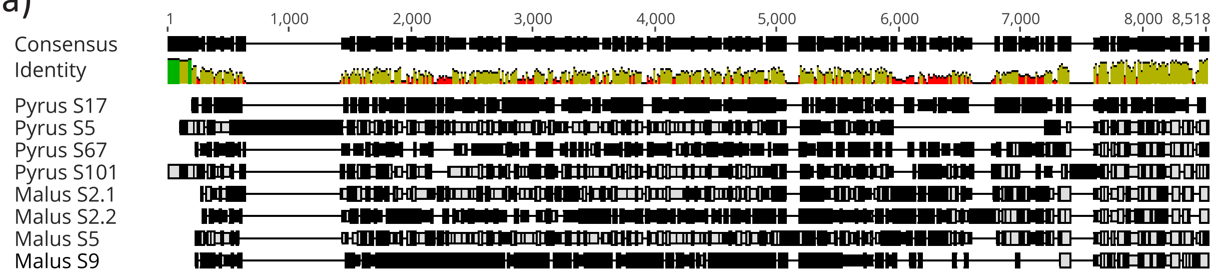

(b)

	Pyrus S17	Pyrus S5	Pyrus S67	Pyrus S101	Malus S2.1	Malus S2.2	Malus S5	Malus S9
Pyrus S17		26.91%	27.83%	32.31%	36.04%	28.47%	34.85%	26.17%
Pyrus S5	26.91%		37.41%	63.29%	63.38%	31.31%	60.23%	34.91%
Pyrus S67	27.83%	37.41%		46.30%	49.52%	37.40%	48.09%	30.40%
Pyrus S101	32.31%	63.29%	46.30%		78.26%	42.04%	74.28%	36.66%
Malus S2.1	36.04%	63.38%	49.52%	78.26%		48.98%	88.39%	38.19%
Malus S2.2	28.47%	31.31%	37.40%	42.04%	48.98%		47.98%	41.16%
Malus S5	34.85%	60.23%	48.09%	74.28%	88.39%	47.98%		37.32%
Malus S9	26.17%	34.91%	30.40%	36.66%	38.19%	41.16%	37.32%	

(c)

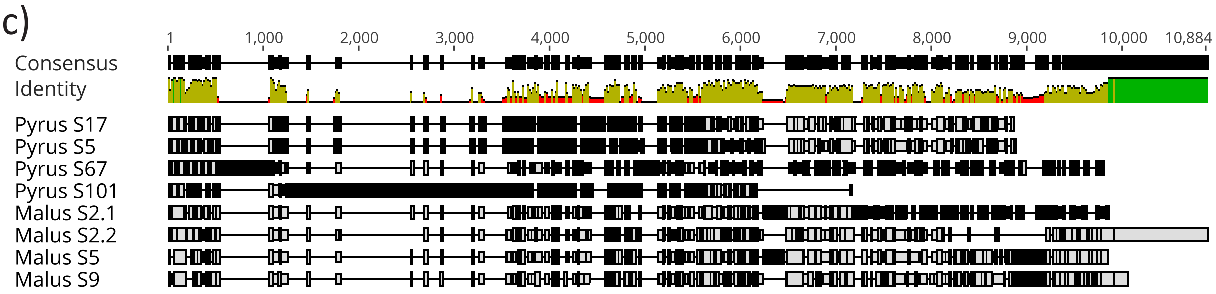

(d)

	Pyrus S17	Pyrus S5	Pyrus S67	Pyrus S101	Malus S2.1	Malus S2.2	Malus S5	Malus S9
Pyrus S17		93.73%	34.07%	45.77%	48.20%	55.24%	61.82%	64.39%
Pyrus S5	93.73%		33.82%	45.85%	47.70%	54.64%	61.14%	63.64%
Pyrus S67	34.07%	33.82%		20.51%	40.03%	42.30%	42.61%	44.13%
Pyrus S101	45.77%	45.85%	20.51%		26.29%	27.44%	25.90%	26.84%
Malus S2.1	48.20%	47.70%	40.03%	26.29%		58.65%	63.77%	62.46%
Malus S2.2	55.24%	54.64%	42.30%	27.44%	58.65%		75.08%	78.96%
Malus S5	61.82%	61.14%	42.61%	25.90%	63.77%	75.08%		94.52%
Malus S9	64.39%	63.64%	44.13%	26.84%	62.46%	78.96%	94.52%	

Figure S26 Comparison analysis of the 5 kb non-coding flanking sequences of *SFBB*s in group VI. (a) A snapshot showing the alignment of the 5kb upstream sequences of *SFBB*s. (b) Pairwise identity of the 5kb upstream sequences of *SFBB*s. (c) A snapshot showing the alignment of the 5kb downstream sequences of *SFBB*s. (d) Pairwise identity of the 5kb downstream sequences of *SFBB*s. Malus S2.1 and S2.2 represent the *Malus SFBB.VI.1-S*$_2$ and *SFBB.VI.2-S*$_2$, respectively.

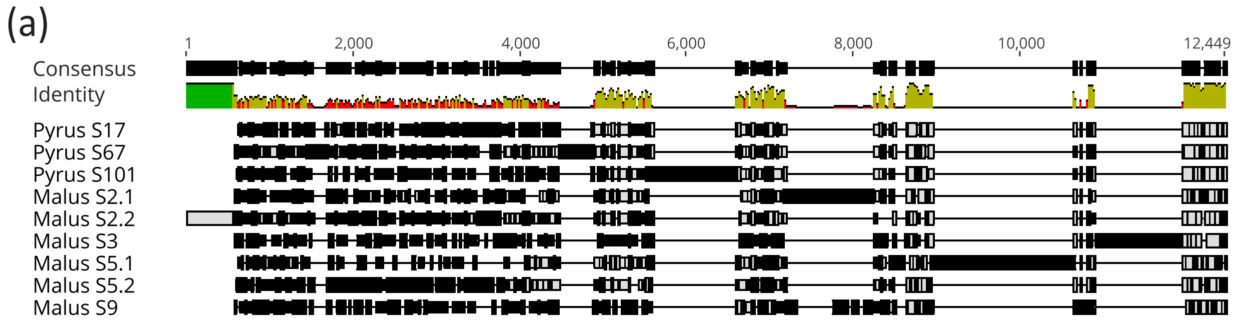

(b)

	Pyrus S17	Pyrus S67	Pyrus S101	Malus S2.1	Malus S2.2	Malus S3	Malus S5.1	Malus S5.2	Malus S9
Pyrus S17		52.19%	42.55%	41.94%	52.93%	31.13%	33.30%	50.98%	37.13%
Pyrus S67	52.19%		43.07%	42.70%	74.14%	32.00%	37.14%	50.53%	38.04%
Pyrus S101	42.55%	43.07%		36.08%	42.28%	27.77%	31.11%	40.44%	33.92%
Malus S2.1	41.94%	42.70%	36.08%		44.73%	29.46%	30.50%	44.28%	39.83%
Malus S2.2	52.93%	74.14%	42.28%	44.73%		35.11%	38.31%	54.72%	38.86%
Malus S3	31.13%	32.00%	27.77%	29.46%	35.11%		23.72%	32.83%	26.86%
Malus S5.1	33.30%	37.14%	31.11%	30.50%	38.31%	23.72%		34.34%	29.07%
Malus S5.2	50.98%	50.53%	40.44%	44.28%	54.72%	32.83%	34.34%		39.94%
Malus S9	37.13%	38.04%	33.92%	39.83%	38.86%	26.86%	29.07%	39.94%	

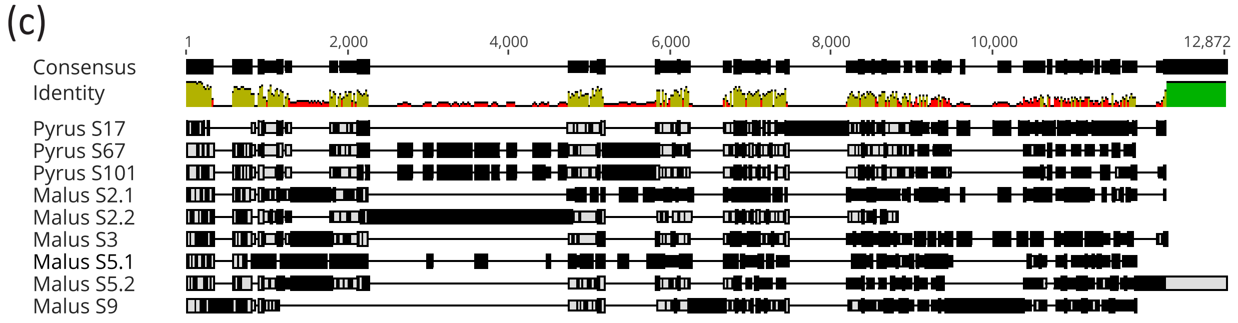

(d)

	Pyrus S17	Pyrus S67	Pyrus S101	Malus S2.1	Malus S2.2	Malus S3	Malus S5.1	Malus S5.2	Malus S9
Pyrus S17		35.58%	34.61%	28.80%	34.88%	39.61%	24.88%	34.50%	35.63%
Pyrus S67	35.58%		95.33%	32.38%	48.47%	37.04%	30.87%	38.13%	33.14%
Pyrus S101	34.61%	95.33%		31.32%	47.29%	35.98%	30.49%	35.25%	33.52%
Malus S2.1	28.80%	32.38%	31.32%		27.82%	44.74%	29.44%	42.15%	28.49%
Malus S2.2	34.88%	48.47%	47.29%	27.82%		37.59%	25.16%	33.69%	32.92%
Malus S3	39.61%	37.04%	35.98%	44.74%	37.59%		32.17%	51.81%	38.76%
Malus S5.1	24.88%	30.87%	30.49%	29.44%	25.16%	32.17%		35.11%	25.13%
Malus S5.2	34.50%	38.13%	35.25%	42.15%	33.69%	51.81%	35.11%		41.29%
Malus S9	35.63%	33.14%	33.52%	28.49%	32.92%	38.76%	25.13%	41.29%	

Figure S27 Comparison analysis of the 5 kb non-coding flanking sequences of *SFBB*s in group VII. (a) A snapshot showing the alignment of the 5kb upstream sequences of *SFBB*s. (b) Pairwise identity of the 5kb upstream sequences of *SFBB*s. (c) A snapshot showing the alignment of the 5kb downstream sequences of *SFBB*s. (d) Pairwise identity of the 5kb downstream sequences of *SFBB*s. Malus S2.1, S2.2, S5.1, and S5.2 represent the *Malus SFBB.VII.1-*S_2, *SFBB.VII.2-*S_2, *SFBB.VII.1-*S_5, and *SFBB.VII.2-*S_5, respectively.

(a)

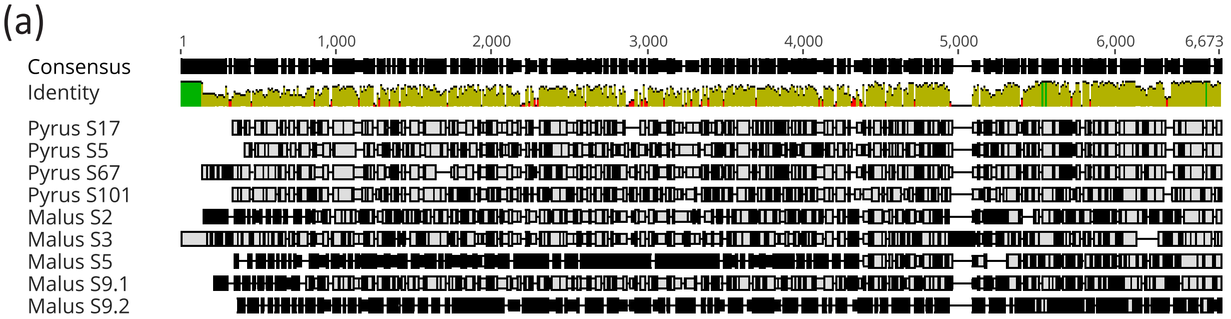

(b)

	Pyrus S17	Pyrus S5	Pyrus S67	Pyrus S101	Malus S2	Malus S3	Malus S5	Malus S9.1	Malus S9.2
Pyrus S17		96.91%	95.87%	95.93%	83.69%	85.93%	53.27%	85.49%	46.30%
Pyrus S5	96.91%		95.96%	96.09%	84.37%	85.75%	53.58%	86.10%	46.37%
Pyrus S67	95.87%	95.96%		95.41%	81.10%	85.60%	53.05%	83.71%	46.43%
Pyrus S101	95.93%	96.09%	95.41%		84.56%	86.98%	53.48%	86.34%	46.70%
Malus S2	83.69%	84.37%	81.10%	84.56%		81.23%	55.75%	93.63%	46.79%
Malus S3	85.93%	85.75%	85.60%	86.98%	81.23%		51.21%	83.51%	44.67%
Malus S5	53.27%	53.58%	53.05%	53.48%	55.75%	51.21%		55.69%	38.46%
Malus S9.1	85.49%	86.10%	83.71%	86.34%	93.63%	83.51%	55.69%		47.07%
Malus S9.2	46.30%	46.37%	46.43%	46.70%	46.79%	44.67%	38.46%	47.07%	

(c)

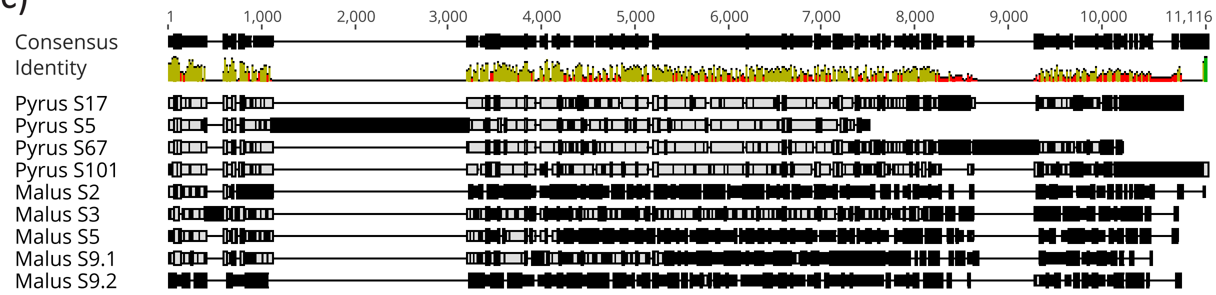

(d)

	Pyrus S17	Pyrus S5	Pyrus S67	Pyrus S101	Malus S2	Malus S3	Malus S5	Malus S9.1	Malus S9.2
Pyrus S17		57.51%	83.17%	90.86%	37.06%	64.61%	45.37%	50.41%	34.48%
Pyrus S5	57.51%		57.44%	56.82%	28.21%	48.70%	34.24%	40.17%	25.38%
Pyrus S67	83.17%	57.44%		77.55%	34.96%	61.04%	41.92%	48.14%	32.40%
Pyrus S101	90.86%	56.82%	77.55%		36.71%	63.27%	45.32%	51.06%	35.37%
Malus S2	37.06%	28.21%	34.96%	36.71%		33.88%	31.94%	30.36%	30.81%
Malus S3	64.61%	48.70%	61.04%	63.27%	33.88%		42.81%	46.53%	31.37%
Malus S5	45.37%	34.24%	41.92%	45.32%	31.94%	42.81%		37.80%	29.16%
Malus S9.1	50.41%	40.17%	48.14%	51.06%	30.36%	46.53%	37.80%		27.32%
Malus S9.2	34.48%	25.38%	32.40%	35.37%	30.81%	31.37%	29.16%	27.32%	

Figure S28 Comparison analysis of the 5 kb non-coding flanking sequences of *SFBB*s in group VIII. (a) A snapshot showing the alignment of the 5kb upstream sequences of *SFBB*s. (b) Pairwise identity of the 5kb upstream sequences of *SFBB*s. (c) A snapshot showing the alignment of the 5kb downstream sequences of *SFBB*s. (d) Pairwise identity of the 5kb downstream sequences of *SFBB*s. Malus S9.1 and S9.2 represent the *Malus SFBB.VIII.1-*S_9 and *SFBB.VIII.2-*S_9, respectively.

(a)

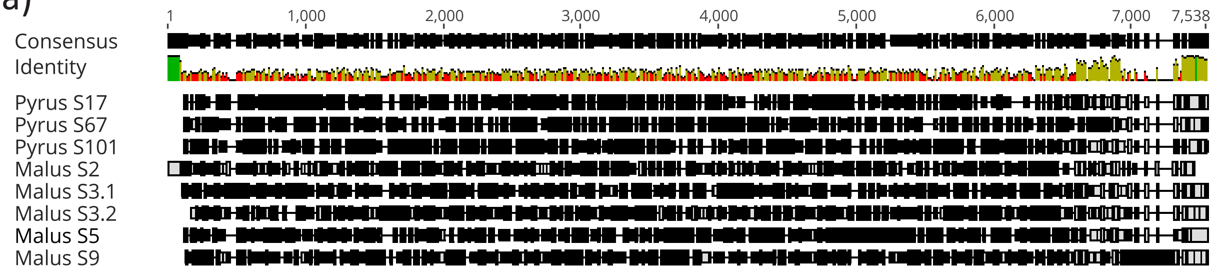

(b)

	Pyrus S17	Pyrus S67	Pyrus S101	Malus S2	Malus S3.1	Malus S3.2	Malus S5	Malus S9
Pyrus S17		27.77%	30.16%	32.81%	31.35%	33.82%	33.47%	33.52%
Pyrus S67	27.77%		27.97%	34.78%	31.16%	35.73%	29.31%	29.67%
Pyrus S101	30.16%	27.97%		35.66%	31.82%	36.59%	31.45%	31.43%
Malus S2	32.81%	34.78%	35.66%		38.61%	100%	34.67%	43.08%
Malus S3.1	31.35%	31.16%	31.82%	38.61%		39.70%	34.92%	36.68%
Malus S3.2	33.82%	35.73%	36.59%	100%	39.70%		35.83%	43.93%
Malus S5	33.47%	29.31%	31.45%	34.67%	34.92%	35.83%		34.17%
Malus S9	33.52%	29.67%	31.43%	43.08%	36.68%	43.93%	34.17%	

(c)

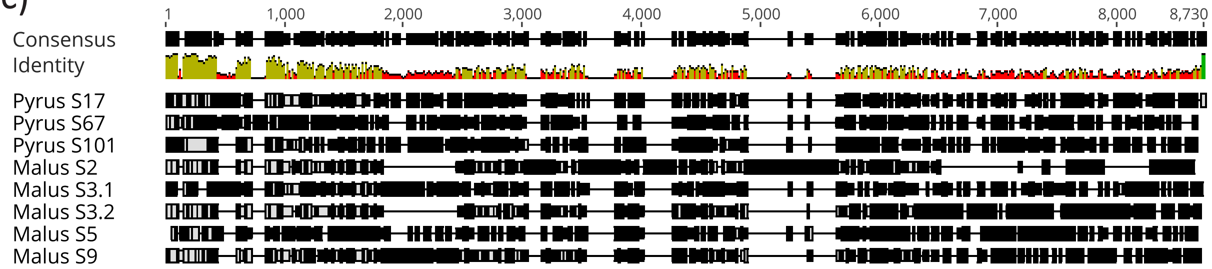

(d)

	Pyrus S17	Pyrus S67	Pyrus S101	Malus S2	Malus S3.1	Malus S3.2	Malus S5	Malus S9
Pyrus S17		33.09%	34.30%	26.84%	39.89%	34.71%	30.87%	39.88%
Pyrus S67	33.09%		31.40%	22.22%	34.49%	29.86%	31.66%	32.91%
Pyrus S101	34.30%	31.40%		26.08%	35.83%	34.22%	32.42%	37.78%
Malus S2	26.84%	22.22%	26.08%		27.17%	44.15%	22.21%	39.70%
Malus S3.1	39.89%	34.49%	35.83%	27.17%		34.65%	31.15%	44.74%
Malus S3.2	34.71%	29.86%	34.22%	44.15%	34.65%		30.84%	52.21%
Malus S5	30.87%	31.66%	32.42%	22.21%	31.15%	30.84%		32.49%
Malus S9	39.88%	32.91%	37.78%	39.70%	44.74%	52.21%	32.49%	

Figure S29 Comparison analysis of the 5 kb non-coding flanking sequences of *SFBB*s in group X. (a) A snapshot showing the alignment of the 5kb upstream sequences of *SFBB*s. (b) Pairwise identity of the 5kb upstream sequences of *SFBB*s. (c) A snapshot showing the alignment of the 5kb downstream sequences of *SFBB*s. (d) Pairwise identity of the 5kb downstream sequences of *SFBB*s. Malus S3.1 and S3.2 represent the *Malus SFBB.X.1-*S_3 and *SFBB.X.2-*S_3, respectively.

(a)

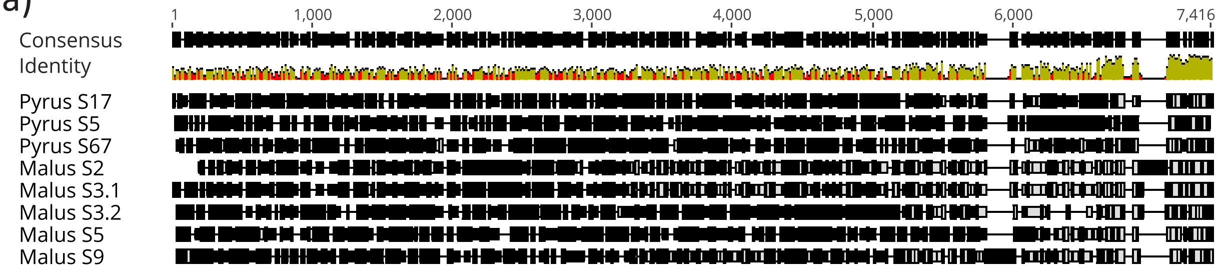

(b)

	Pyrus S17	Pyrus S5	Pyrus S67	Malus S2	Malus S3.1	Malus S3.2	Malus S5	Malus S9
Pyrus S17		29.39%	32.88%	39.32%	40.28%	32.25%	33.16%	36.78%
Pyrus S5	29.39%		32.48%	36.08%	36.94%	31.17%	32.67%	33.10%
Pyrus S67	32.88%	32.48%		41.21%	42.07%	50.23%	35.68%	39.36%
Malus S2	39.32%	36.08%	41.21%		96.55%	42.62%	39.43%	55.46%
Malus S3.1	40.28%	36.94%	42.07%	96.55%		43.44%	40.27%	56.91%
Malus S3.2	32.25%	31.17%	50.23%	42.62%	43.44%		34.05%	39.15%
Malus S5	33.16%	32.67%	35.68%	39.43%	40.27%	34.05%		38.06%
Malus S9	36.78%	33.10%	39.36%	55.46%	56.91%	39.15%	38.06%	

(c)

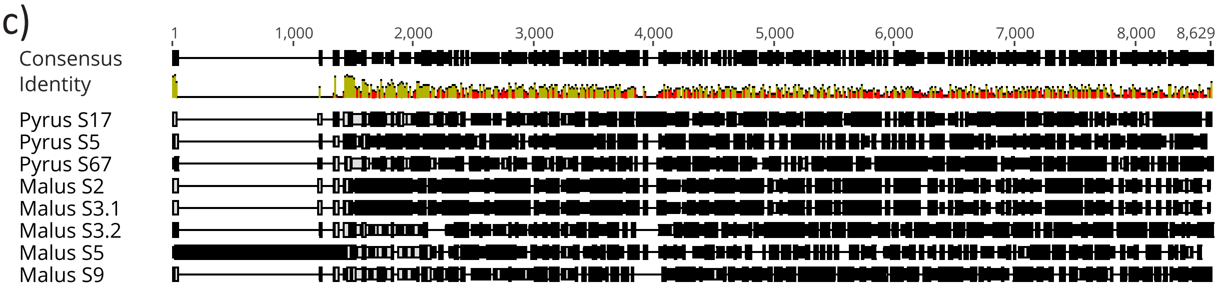

(d)

	Pyrus S17	Pyrus S5	Pyrus S67	Malus S2	Malus S3.1	Malus S3.2	Malus S5	Malus S9
Pyrus S17		31.58%	33.28%	30.69%	30.69%	41.38%	26.98%	45.15%
Pyrus S5	31.58%		29.59%	27.04%	27.04%	30.74%	23.57%	32.38%
Pyrus S67	33.28%	29.59%		28.49%	28.49%	34.79%	23.90%	36.13%
Malus S2	30.69%	27.04%	28.49%		100%	29.33%	21.66%	32.31%
Malus S3.1	30.69%	27.04%	28.49%	100%		29.33%	21.66%	32.31%
Malus S3.2	41.38%	30.74%	34.79%	29.33%	29.33%		25.55%	45.21%
Malus S5	26.98%	23.57%	23.90%	21.66%	21.66%	25.55%		27.70%
Malus S9	45.15%	32.38%	36.13%	32.31%	32.31%	45.21%	27.70%	

Figure S30 Comparison analysis of the 5 kb non-coding flanking sequences of *SFBB*s in group XI. (a) A snapshot showing the alignment of the 5kb upstream sequences of *SFBB*s. (b) Pairwise identity of the 5kb upstream sequences of *SFBB*s. (c) A snapshot showing the alignment of the 5kb downstream sequences of *SFBB*s. (d) Pairwise identity of the 5kb downstream sequences of *SFBB*s. Malus S3.1 and S3.2 represent the *Malus SFBB.XI.1-*S_3 and *SFBB.XI.2-*S_3, respectively.
