# Supplementary material for: Long-read genome sequencing reveals the sequence characteristics of pear self-incompatibility locus
Source: Mol Hortic. 2025 Mar 1;5:13. doi: 10.1186/s43897-024-00132-0 (PMC11871771; doi:10.1186/s43897-024-00132-0)
Supplement: Supplementary file 1 — Supplementary Material 1: Table S1 Comparison of ‘Yali’ genome with previously published assemblies of Pyrus and Malus species. Table S2 Annotation of the repeats in ‘Yali’ genome. Table S3 Annotation of the non-coding RNAs in ‘Dananguo’ and 'Yali' genomes. Table S4 Identification of the F-box genes in Pyrus, Malus and Prunus S-loci. Table S5 Function annotation of the predicted genes in S-loci. Table S6 Sequence similarity (%) among Pyrus and Malus SFBB genes. Table S7 Sequence similarity (%) among Prunus SFB and SLF genes. Table S8 Sequence similarity among Prunus SFB and SLF genes. Table S9 Sequence similarity (%) among Pyrus and Malus S-RNase genes. Table S10 Prediction of gene duplication events of Pyrus and Malus SFBB genes. Table S11 Sequence similarity of the non-coding flanking sequences of SFBBs in Pyrus and Malus S-loci. Table S12 Analysis of number and length of LTR retrotransposon in different S-loci. Table S13 Identification of the LTR retrotransposon in different S-loci. Table S14 RPKM values of the genes commonly existed in the tested S-loci. Table S15 Sequence similarity (%) among the reported Pyrus S-RNase genes. Table S16 The accession numbers of S-RNase and S-locus F-box genes in Pyrus, Malus, and Prunus.Table S17 Primers used in this study. Figure S1 Isolation of the conserved F-box motif in the reported S-locus F-box proteins in Pyrus and Malus. The accession numbers of these F-box proteins were listed in Table S13. Figure S2 Phylogenetic classifications of S-locus F-box genes in Prunus. The S-locus F-box (SLF/SFB) proteins in Prunus comprised by 12 groups, SLF1→SLF11 and SFB. Each group were highlighted with different colors. Figure S3 Phylogenetic analysis of the F-box genes identified from this and previous studies. Cycles with black color present the F-box genes identified from previous study (Huang et al., 2023). The rates (%) of different types of gene duplication events (dispersed, proximal, tandem and transposed) of the S-locus F-box ge [file 43897_2024_132_MOESM1_ESM.zip › Supplementary Figures S31 to S40.pdf]

(a)

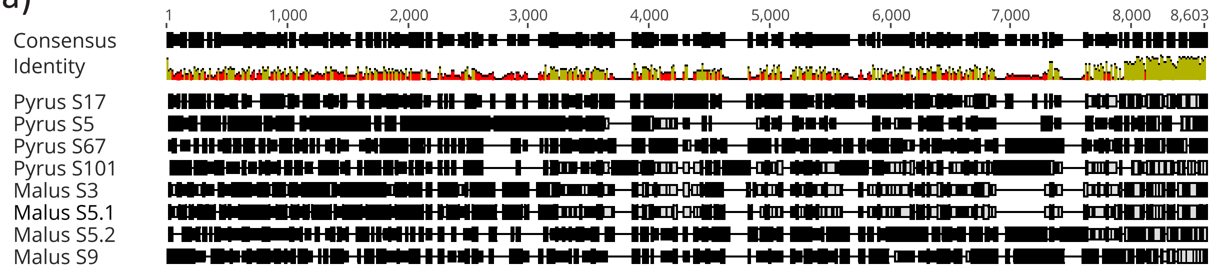

(b)

	Pyrus S17	Pyrus S5	Pyrus S67	Pyrus S101	Malus S3	Malus S5.1	Malus S5.2	Malus S9
Pyrus S17		26.56%	30.12%	34.58%	42.09%	42.06%	32.88%	31.21%
Pyrus S5	26.56%		23.66%	27.01%	38.42%	38.45%	24.00%	27.41%
Pyrus S67	30.12%	23.66%		30.82%	34.64%	34.63%	30.37%	30.44%
Pyrus S101	34.58%	27.01%	30.82%		48.20%	48.19%	33.65%	33.07%
Malus S3	42.09%	38.42%	34.64%	48.20%		99.96%	34.97%	38.67%
Malus S5.1	42.06%	38.45%	34.63%	48.19%	99.96%		34.95%	38.67%
Malus S5.2	32.88%	24.00%	30.37%	33.65%	34.97%	34.95%		30.95%
Malus S9	31.21%	27.41%	30.44%	33.07%	38.67%	38.67%	30.95%	

(c)

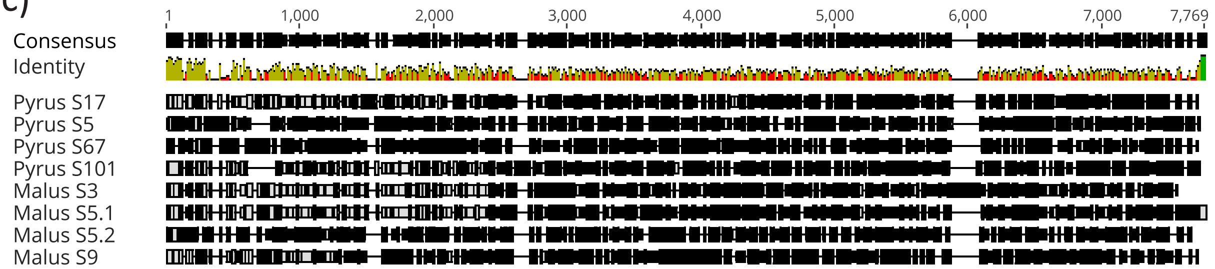

(d)

	Pyrus S17	Pyrus S5	Pyrus S67	Pyrus S101	Malus S3	Malus S5.1	Malus S5.2	Malus S9
Pyrus S17		34.46%	30.57%	43.84%	46.18%	46.75%	31.16%	38.25%
Pyrus S5	34.46%		28.38%	34.89%	34.32%	35.23%	27.65%	31.97%
Pyrus S67	30.57%	28.38%		29.85%	32.18%	32.86%	27.72%	31.50%
Pyrus S101	43.84%	34.89%	29.85%		42.76%	43.68%	31.40%	34.67%
Malus S3	46.18%	34.32%	32.18%	42.76%		95.73%	30.27%	36.68%
Malus S5.1	46.75%	35.23%	32.86%	43.68%	95.73%		31.13%	37.44%
Malus S5.2	31.16%	27.65%	27.72%	31.40%	30.27%	31.13%		28.29%
Malus S9	38.25%	31.97%	31.50%	34.67%	36.68%	37.44%	28.29%	

Figure S31 Comparison analysis of the 5 kb non-coding flanking sequences of *SFBB*s in group XII. (a) A snapshot showing the alignment of the 5kb upstream sequences of *SFBB*s. (b) Pairwise identity of the 5kb upstream sequences of *SFBB*s. (c) A snapshot showing the alignment of the 5kb downstream sequences of *SFBB*s. (d) Pairwise identity of the 5kb downstream sequences of *SFBB*s. Malus S5.1 and S5.2 represent the *Malus SFBB.XII.1-*S_5 and *SFBB.XII.2-*S_5, respectively.

(a)

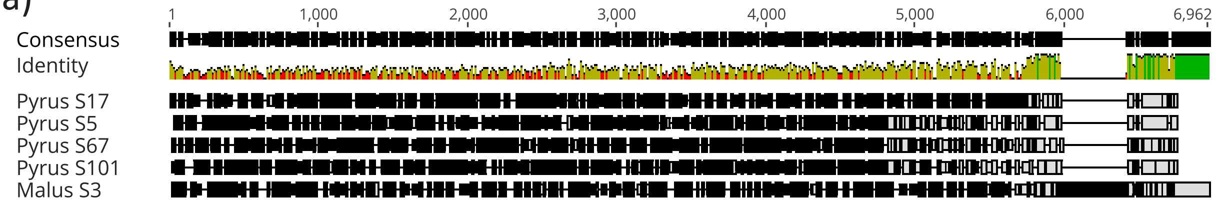

(b)

	Pyrus S17	Pyrus S5	Pyrus S67	Pyrus S101	Malus S3
Pyrus S17		38.99%	37.66%	38.27%	32.49%
Pyrus S5	38.99%		48.14%	68.30%	36.44%
Pyrus S67	37.66%	48.14%		47.47%	33.48%
Pyrus S101	38.27%	68.30%	47.47%		35.47%
Malus S3	32.49%	36.44%	33.48%	35.47%	

(c)

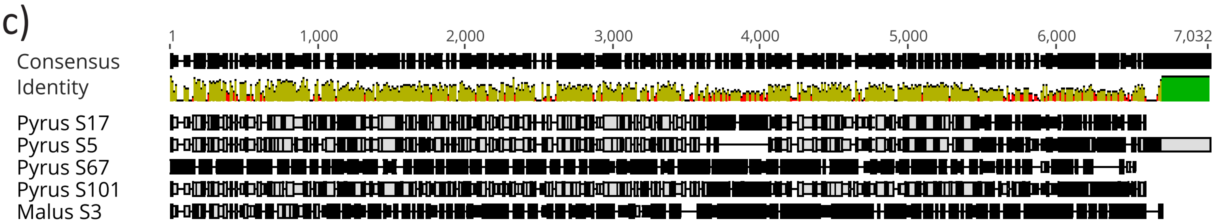

(d)

	Pyrus S17	Pyrus S5	Pyrus S67	Pyrus S101	Malus S3
Pyrus S17		77.57%	33.15%	84.62%	44.79%
Pyrus S5	77.57%		31.91%	87.09%	42.73%
Pyrus S67	33.15%	31.91%		33.25%	36.18%
Pyrus S101	84.62%	87.09%	33.25%		45.74%
Malus S3	44.79%	42.73%	36.18%	45.74%	

Figure S32 Comparison analysis of the 5 kb non-coding flanking sequences of *SFBB*s in group XIII. (a) A snapshot showing the alignment of the 5kb upstream sequences of *SFBB*s. (b) Pairwise identity of the 5kb upstream sequences of *SFBB*s. (c) A snapshot showing the alignment of the 5kb downstream sequences of *SFBB*s. (d) Pairwise identity of the 5kb downstream sequences of *SFBB*s.

(a)

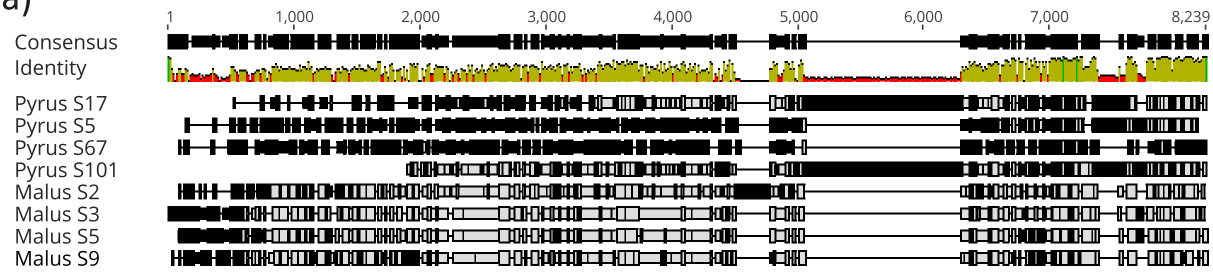

(b)

	Pyrus S17	Pyrus S5	Pyrus S67	Pyrus S101	Malus S2	Malus S3	Malus S5	Malus S9
Pyrus S17		33.99%	27.50%	79.98%	43.56%	45.47%	45.55%	45.32%
Pyrus S5	33.99%		32.14%	44.39%	45.68%	47.12%	47.12%	45.21%
Pyrus S67	27.50%	32.14%		35.17%	44.56%	44.69%	44.76%	43.81%
Pyrus S101	79.98%	44.39%	35.17%		61.39%	65.88%	65.94%	64.47%
Malus S2	43.56%	45.68%	44.56%	61.39%		85.00%	85.01%	83.20%
Malus S3	45.47%	47.12%	44.69%	65.88%	85.00%		100%	91.74%
Malus S5	45.55%	47.12%	44.76%	65.94%	85.01%	100%		92.56%
Malus S9	45.32%	45.21%	43.81%	64.47%	83.20%	91.74%	92.56%	

(c)

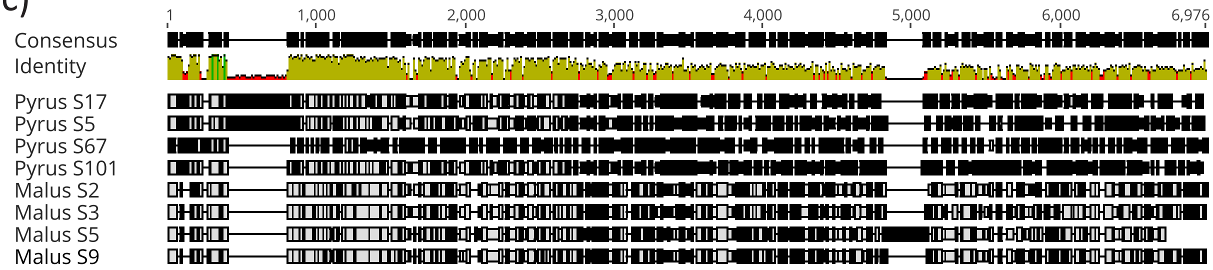

(d)

	Pyrus S17	Pyrus S5	Pyrus S67	Pyrus S101	Malus S2	Malus S3	Malus S5	Malus S9
Pyrus S17		63.22%	34.59%	58.55%	48.49%	48.34%	47.42%	48.38%
Pyrus S5	63.22%		36.67%	65.58%	51.03%	51.09%	49.77%	51.06%
Pyrus S67	34.59%	36.67%		39.91%	38.57%	38.48%	37.22%	38.57%
Pyrus S101	58.55%	65.58%	39.91%		54.39%	54.43%	52.92%	54.24%
Malus S2	48.49%	51.03%	38.57%	54.39%		97.27%	92.11%	97.28%
Malus S3	48.34%	51.09%	38.48%	54.43%	97.27%		94.75%	98.61%
Malus S5	47.42%	49.77%	37.22%	52.92%	92.11%	94.75%		93.46%
Malus S9	48.38%	51.06%	38.57%	54.24%	97.28%	98.61%	93.46%	

Figure S33 Comparison analysis of the 5 kb non-coding flanking sequences of *SFBB*s in group XIV. (a) A snapshot showing the alignment of the 5kb upstream sequences of *SFBB*s. (b) Pairwise identity of the 5kb upstream sequences of *SFBB*s. (c) A snapshot showing the alignment of the 5kb downstream sequences of *SFBB*s. (d) Pairwise identity of the 5kb downstream sequences of *SFBB*s.

(a)

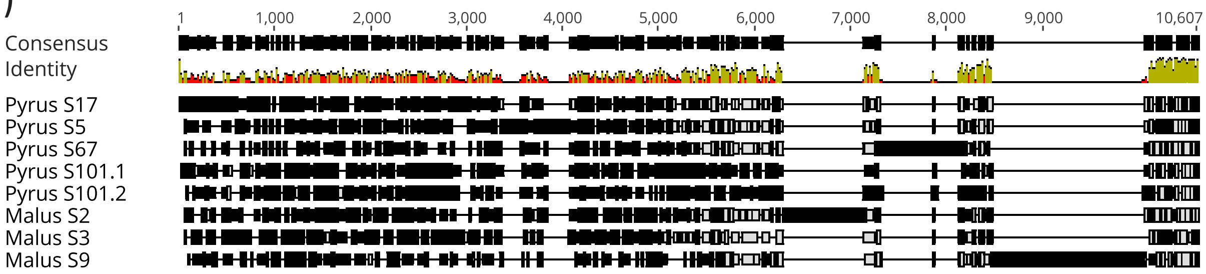

(b)

	Pyrus S17	Pyrus S5	Pyrus S67	Pyrus S101.1	Pyrus S101.2	Malus S2	Malus S3	Malus S9
Pyrus S17		59.85%	33.90%	34.24%	34.04%	36.02%	46.96%	29.12%
Pyrus S5	59.85%		35.51%	33.57%	32.98%	38.46%	43.49%	29.66%
Pyrus S67	33.90%	35.51%		28.45%	28.71%	32.75%	33.74%	27.84%
Pyrus S101.1	34.24%	33.57%	28.45%		61.18%	29.87%	33.21%	23.59%
Pyrus S101.2	34.04%	32.98%	28.71%	61.18%		29.69%	32.13%	24.29%
Malus S2	36.02%	38.46%	32.75%	29.87%	29.69%		35.16%	27.15%
Malus S3	46.96%	43.49%	33.74%	33.21%	32.13%	35.16%		28.62%
Malus S9	29.12%	29.66%	27.84%	23.59%	24.29%	27.15%	28.62%	

(c)

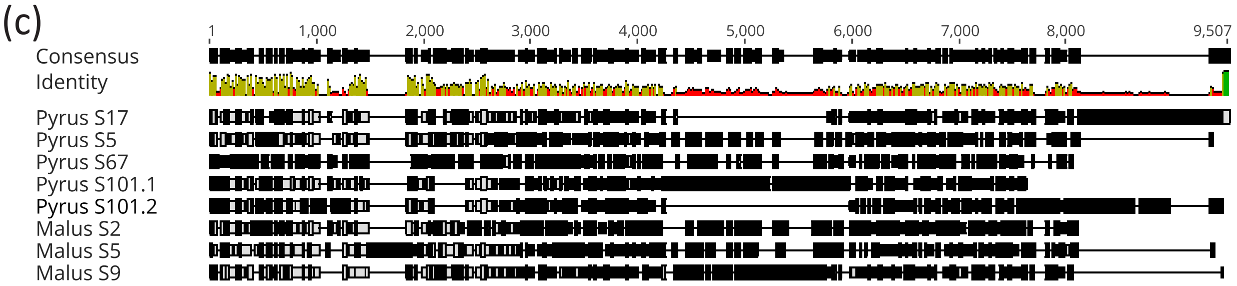

(d)

	Pyrus S17	Pyrus S5	Pyrus S67	Pyrus S101.1	Pyrus S101.2	Malus S2	Malus S5	Malus S9
Pyrus S17		33.41%	28.60%	32.21%	35.07%	37.44%	35.62%	50.72%
Pyrus S5	33.41%		30.89%	35.27%	28.46%	41.06%	41.71%	42.99%
Pyrus S67	28.60%	30.89%		26.42%	24.80%	30.43%	28.58%	29.29%
Pyrus S101.1	32.21%	35.27%	26.42%		59.24%	35.72%	33.51%	41.01%
Pyrus S101.2	35.07%	28.46%	24.80%	59.24%		30.39%	27.73%	27.36%
Malus S2	37.44%	41.06%	30.43%	35.72%	30.39%		37.95%	41.81%
Malus S5	35.62%	41.71%	28.58%	33.51%	27.73%	37.95%		45.10%
Malus S9	50.72%	42.99%	29.29%	41.01%	27.36%	41.81%	45.10%	

Figure S34 Comparison analysis of the 5 kb non-coding flanking sequences of *SFBB*s in group XV. (a) A snapshot showing the alignment of the 5kb upstream sequences of *SFBB*s. (b) Pairwise identity of the 5kb upstream sequences of *SFBB*s. (c) A snapshot showing the alignment of the 5kb downstream sequences of *SFBB*s. (d) Pairwise identity of the 5kb downstream sequences of *SFBB*s. Pyrus S101.1 and S101.2 represent the *Pyrus SFBB.XV.1-S_{101}* and *SFBB.XV.2-S_{101}*, respectively.

(a)

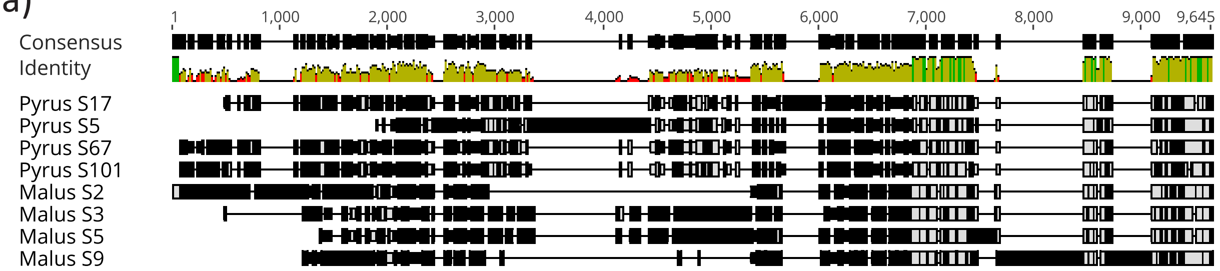

(b)

	Pyrus S17	Pyrus S5	Pyrus S67	Pyrus S101	Malus S2	Malus S3	Malus S5	Malus S9
Pyrus S17		64.78%	84.36%	87.20%	40.88%	45.03%	45.25%	37.05%
Pyrus S5	64.78%		68.93%	70.13%	37.89%	42.97%	41.79%	31.67%
Pyrus S67	84.36%	68.93%		90.37%	42.38%	47.09%	47.90%	39.00%
Pyrus S101	87.20%	70.13%	90.37%		42.38%	47.92%	48.25%	39.34%
Malus S2	40.88%	37.89%	42.38%	42.38%		54.10%	59.76%	72.75%
Malus S3	45.03%	42.97%	47.09%	47.92%	54.10%		96.29%	49.30%
Malus S5	45.25%	41.79%	47.90%	48.25%	59.76%	96.29%		48.14%
Malus S9	37.05%	31.67%	39.00%	39.34%	72.75%	49.30%	48.14%	

(c)

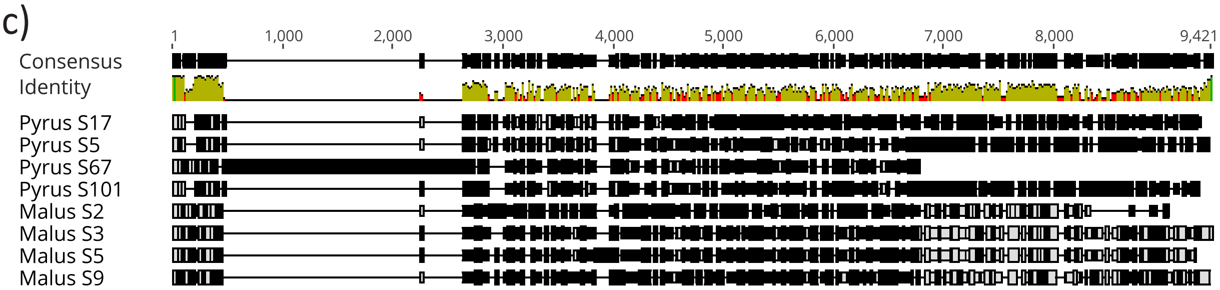

(d)

	Pyrus S17	Pyrus S5	Pyrus S67	Pyrus S101	Malus S2	Malus S3	Malus S5	Malus S9
Pyrus S17		44.87%	32.94%	45.02%	30.77%	38.03%	37.19%	37.70%
Pyrus S5	44.87%		49.59%	80.90%	31.58%	38.44%	37.62%	38.11%
Pyrus S67	32.94%	49.59%		49.94%	23.57%	25.94%	25.32%	25.67%
Pyrus S101	45.02%	80.90%	49.94%		31.69%	38.39%	37.49%	38.04%
Malus S2	30.77%	31.58%	23.57%	31.69%		51.00%	49.81%	50.72%
Malus S3	38.03%	38.44%	25.94%	38.39%	51.00%		97.31%	97.87%
Malus S5	37.19%	37.62%	25.32%	37.49%	49.81%	97.31%		95.28%
Malus S9	37.70%	38.11%	25.67%	38.04%	50.72%	97.87%	95.28%	

Figure S35 Comparison analysis of the 5 kb non-coding flanking sequences of *SFBB*s in group XVI. (a) A snapshot showing the alignment of the 5kb upstream sequences of *SFBB*s. (b) Pairwise identity of the 5kb upstream sequences of *SFBB*s. (c) A snapshot showing the alignment of the 5kb downstream sequences of *SFBB*s. (d) Pairwise identity of the 5kb downstream sequences of *SFBB*s.

(a)

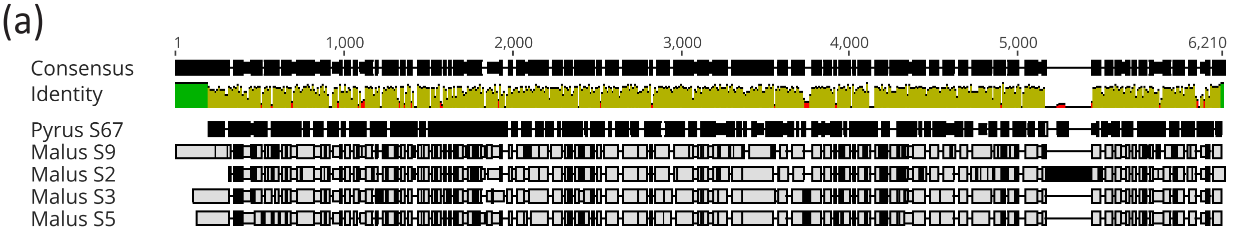

(b)

	Pyrus S67	Malus S9	Malus S2	Malus S3	Malus S5
Pyrus S67		42.92%	42.19%	42.85%	42.85%
Malus S9	42.92%		92.67%	97.62%	97.61%
Malus S2	42.19%	92.67%		92.57%	92.57%
Malus S3	42.85%	97.62%	92.57%		100%
Malus S5	42.85%	97.61%	92.57%	100%	

(c)

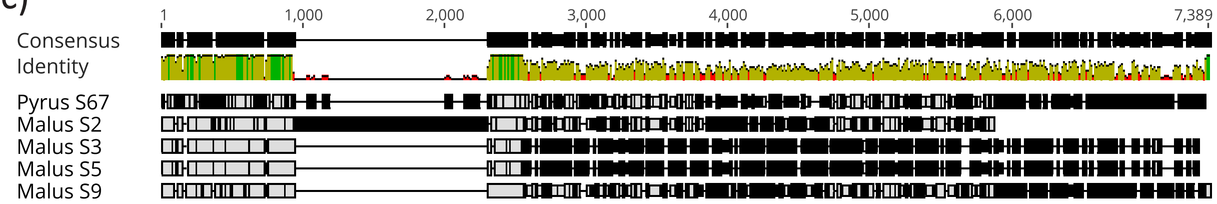

(d)

	Pyrus S67	Malus S2	Malus S3	Malus S5	Malus S9
Pyrus S67		57.55%	43.70%	43.70%	71.07%
Malus S2	57.55%		41.28%	41.28%	70.88%
Malus S3	43.70%	41.28%		100%	48.89%
Malus S5	43.70%	41.28%	100%		48.89%
Malus S9	71.07%	70.88%	48.89%	48.89%	

Figure S36 Comparison analysis of the 5 kb non-coding flanking sequences of *SFBB*s in group XVII. (a) A snapshot showing the alignment of the 5kb upstream sequences of *SFBB*s. (b) Pairwise identity of the 5kb upstream sequences of *SFBB*s. (c) A snapshot showing the alignment of the 5kb downstream sequences of *SFBB*s. (d) Pairwise identity of the 5kb downstream sequences of *SFBB*s.

(a)

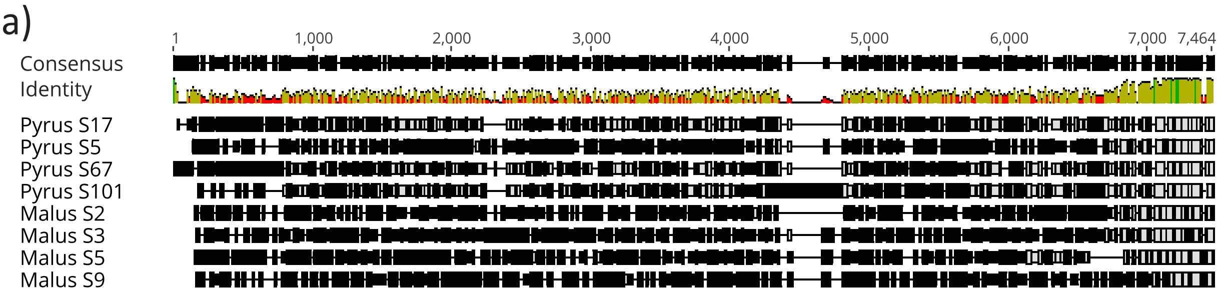

(b)

	Pyrus S17	Pyrus S5	Pyrus S67	Pyrus S101	Malus S2	Malus S3	Malus S5	Malus S9
Pyrus S17		42.04%	95.86%	77.33%	40.10%	42.37%	41.42%	32.17%
Pyrus S5	42.04%		41.53%	38.95%	35.55%	37.69%	36.42%	31.05%
Pyrus S67	95.86%	41.53%		76.28%	39.71%	42.28%	41.16%	32.04%
Pyrus S101	77.33%	38.95%	76.28%		36.13%	40.21%	38.91%	31.19%
Malus S2	40.10%	35.55%	39.71%	36.13%		33.96%	34.13%	30.66%
Malus S3	42.37%	37.69%	42.28%	40.21%	33.96%		35.07%	31.70%
Malus S5	41.42%	36.42%	41.16%	38.91%	34.13%	35.07%		30.28%
Malus S9	32.17%	31.05%	32.04%	31.19%	30.66%	31.70%	30.28%	

(c)

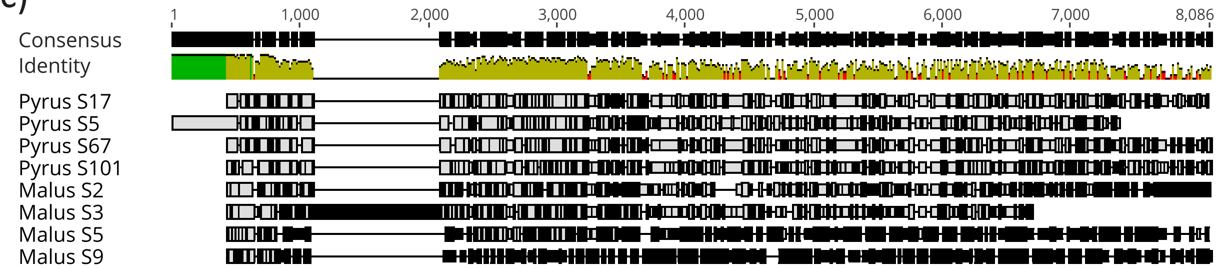

(d)

	Pyrus S17	Pyrus S5	Pyrus S67	Pyrus S101	Malus S2	Malus S3	Malus S5	Malus S9
Pyrus S17		97.16%	97.39%	92.59%	76.18%	71.04%	50.51%	42.59%
Pyrus S5	97.16%		96.99%	92.60%	82.09%	71.23%	52.35%	43.02%
Pyrus S67	97.39%	96.99%		92.55%	76.06%	71.07%	50.17%	42.80%
Pyrus S101	92.59%	92.60%	92.55%		76.76%	71.56%	50.29%	43.31%
Malus S2	76.18%	82.09%	76.06%	76.76%		70.52%	47.98%	37.22%
Malus S3	71.04%	71.23%	71.07%	71.56%	70.52%		44.63%	34.95%
Malus S5	50.51%	52.35%	50.17%	50.29%	47.98%	44.63%		30.03%
Malus S9	42.59%	43.02%	42.80%	43.31%	37.22%	34.95%	30.03%	

Figure S37 Comparison analysis of the 5 kb non-coding flanking sequences of *SFBB*s in group XVIII. (a) A snapshot showing the alignment of the 5kb upstream sequences of *SFBB*s. (b) Pairwise identity of the 5kb upstream sequences of *SFBB*s. (c) A snapshot showing the alignment of the 5kb downstream sequences of *SFBB*s. (d) Pairwise identity of the 5kb downstream sequences of *SFBB*s.

(a)

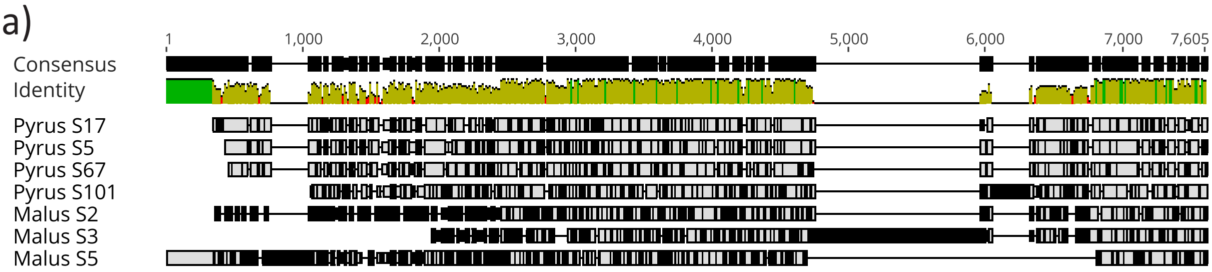

(b)

	Pyrus S17	Pyrus S5	Pyrus S67	Pyrus S101	Malus S2	Malus S3	Malus S5
Pyrus S17		96.06%	95.78%	84.70%	70.00%	59.64%	71.71%
Pyrus S5	96.06%		96.69%	85.44%	71.18%	60.36%	71.56%
Pyrus S67	95.78%	96.69%		85.50%	70.85%	59.84%	70.94%
Pyrus S101	84.70%	85.44%	85.50%		70.04%	57.77%	72.48%
Malus S2	70.00%	71.18%	70.85%	70.04%		69.28%	59.87%
Malus S3	59.64%	60.36%	59.84%	57.77%	69.28%		53.60%
Malus S5	71.71%	71.56%	70.94%	72.48%	59.87%	53.60%	

(c)

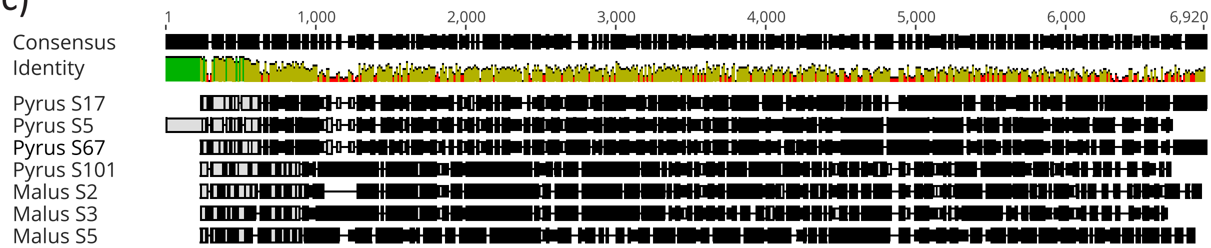

(d)

	Pyrus S17	Pyrus S5	Pyrus S67	Pyrus S101	Malus S2	Malus S3	Malus S5
Pyrus S17		64.47%	63.06%	36.23%	37.42%	36.44%	36.70%
Pyrus S5	64.47%		97.59%	36.86%	36.94%	36.32%	37.03%
Pyrus S67	63.06%	97.59%		36.60%	36.90%	36.41%	36.47%
Pyrus S101	36.23%	36.86%	36.60%		74.65%	76.94%	37.49%
Malus S2	37.42%	36.94%	36.90%	74.65%		84.22%	38.08%
Malus S3	36.44%	36.32%	36.41%	76.94%	84.22%		38.13%
Malus S5	36.70%	37.03%	36.47%	37.49%	38.08%	38.13%	

Figure S38 Comparison analysis of the 5 kb non-coding flanking sequences of *SFBB*s in group XIX. (a) A snapshot showing the alignment of the 5kb upstream sequences of *SFBB*s. (b) Pairwise identity of the 5kb upstream sequences of *SFBB*s. (c) A snapshot showing the alignment of the 5kb downstream sequences of *SFBB*s. (d) Pairwise identity of the 5kb downstream sequences of *SFBB*s.

(a)

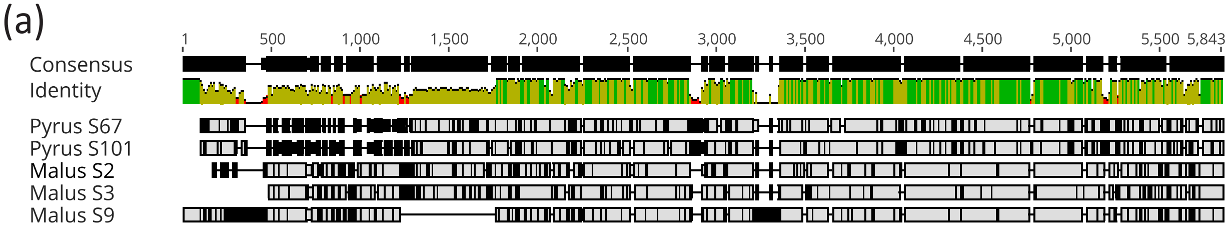

(b)

	Pyrus S67	Pyrus S101	Malus S2	Malus S3	Malus S9
Pyrus S67		94.68%	78.67%	82.00%	70.50%
Pyrus S101	94.68%		79.39%	82.53%	71.03%
Malus S2	78.67%	79.39%		94.56%	80.99%
Malus S3	82.00%	82.53%	94.56%		83.52%
Malus S9	70.50%	71.03%	80.99%	83.52%	

(c)

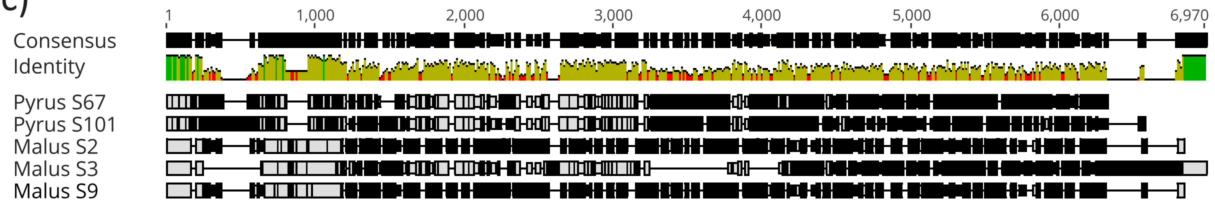

(d)

	Pyrus S67	Pyrus S101	Malus S2	Malus S3	Malus S9
Pyrus S67		68.81%	40.35%	71.06%	40.33%
Pyrus S101	68.81%		38.58%	49.64%	38.57%
Malus S2	40.35%	38.58%		37.28%	98.43%
Malus S3	71.06%	49.64%	37.28%		37.30%
Malus S9	40.33%	38.57%	98.43%	37.30%	

Figure S39 Comparison analysis of the 5 kb non-coding flanking sequences of *SFBB*s in group XXI. (a) A snapshot showing the alignment of the 5kb upstream sequences of *SFBB*s. (b) Pairwise identity of the 5kb upstream sequences of *SFBB*s. (c) A snapshot showing the alignment of the 5kb downstream sequences of *SFBB*s. (d) Pairwise identity of the 5kb downstream sequences of *SFBB*s.

(a)

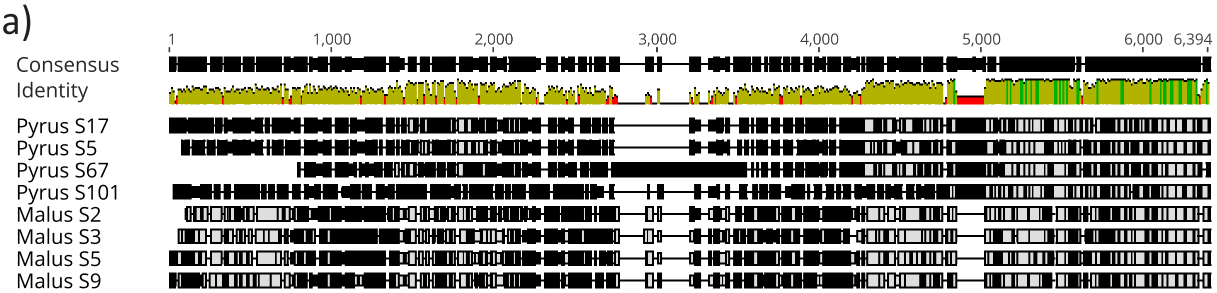

(b)

	Pyrus S17	Pyrus S5	Pyrus S67	Pyrus S101	Malus S2	Malus S3	Malus S5	Malus S9
Pyrus S17		93.88%	80.46%	53.98%	60.41%	59.67%	59.95%	59.81%
Pyrus S5	93.88%		81.50%	53.63%	60.22%	59.52%	60.08%	60.19%
Pyrus S67	80.46%	81.50%		49.76%	58.44%	57.67%	58.48%	58.30%
Pyrus S101	53.98%	53.63%	49.76%		48.46%	47.79%	48.38%	48.12%
Malus S2	60.41%	60.22%	58.44%	48.46%		95.16%	98.92%	96.35%
Malus S3	59.67%	59.52%	57.67%	47.79%	95.16%		95.24%	95.20%
Malus S5	59.95%	60.08%	58.48%	48.38%	98.92%	95.24%		95.42%
Malus S9	59.81%	60.19%	58.30%	48.12%	96.35%	95.20%	95.42%	

(c)

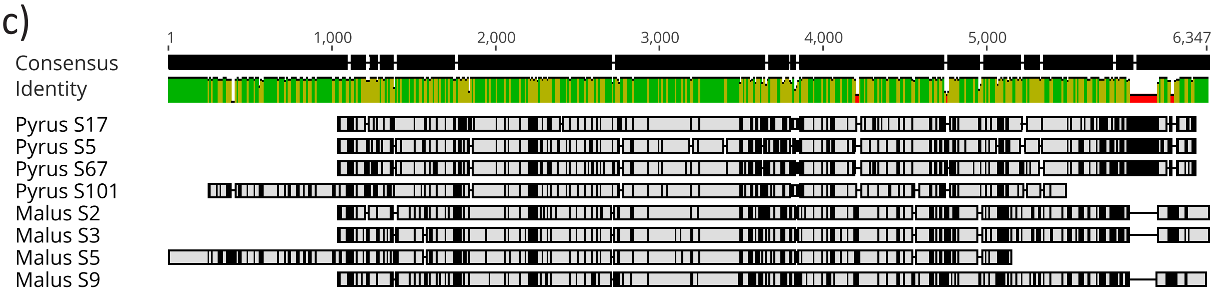

(d)

	Pyrus S17	Pyrus S5	Pyrus S67	Pyrus S101	Malus S2	Malus S3	Malus S5	Malus S9
Pyrus S17		98.28%	98.32%	98.46%	90.23%	90.21%	93.75%	90.10%
Pyrus S5	98.28%		98.90%	98.34%	90.21%	90.18%	93.81%	90.04%
Pyrus S67	98.32%	98.90%		98.09%	90.11%	90.09%	93.64%	89.98%
Pyrus S101	98.46%	98.34%	98.09%		93.71%	93.69%	93.53%	93.52%
Malus S2	90.23%	90.21%	90.11%	93.71%		99.14%	99.14%	98.82%
Malus S3	90.21%	90.18%	90.09%	93.69%	99.14%		99.98%	98.67%
Malus S5	93.75%	93.81%	93.64%	93.53%	99.14%	99.98%		98.56%
Malus S9	90.10%	90.04%	89.98%	93.52%	98.82%	98.67%	98.56%	

Figure S40 Comparison analysis of the 5 kb non-coding flanking sequences of *SFBB*s in group XXII. (a) A snapshot showing the alignment of the 5kb upstream sequences of *SFBB*s. (b) Pairwise identity of the 5kb upstream sequences of *SFBB*s. (c) A snapshot showing the alignment of the 5kb downstream sequences of *SFBB*s. (d) Pairwise identity of the 5kb downstream sequences of *SFBB*s.
