# Supplementary material for: Long-read genome sequencing reveals the sequence characteristics of pear self-incompatibility locus
Source: Mol Hortic. 2025 Mar 1;5:13. doi: 10.1186/s43897-024-00132-0 (PMC11871771; doi:10.1186/s43897-024-00132-0)
Supplement: Supplementary file 1 — Supplementary Material 1: Table S1 Comparison of ‘Yali’ genome with previously published assemblies of Pyrus and Malus species. Table S2 Annotation of the repeats in ‘Yali’ genome. Table S3 Annotation of the non-coding RNAs in ‘Dananguo’ and 'Yali' genomes. Table S4 Identification of the F-box genes in Pyrus, Malus and Prunus S-loci. Table S5 Function annotation of the predicted genes in S-loci. Table S6 Sequence similarity (%) among Pyrus and Malus SFBB genes. Table S7 Sequence similarity (%) among Prunus SFB and SLF genes. Table S8 Sequence similarity among Prunus SFB and SLF genes. Table S9 Sequence similarity (%) among Pyrus and Malus S-RNase genes. Table S10 Prediction of gene duplication events of Pyrus and Malus SFBB genes. Table S11 Sequence similarity of the non-coding flanking sequences of SFBBs in Pyrus and Malus S-loci. Table S12 Analysis of number and length of LTR retrotransposon in different S-loci. Table S13 Identification of the LTR retrotransposon in different S-loci. Table S14 RPKM values of the genes commonly existed in the tested S-loci. Table S15 Sequence similarity (%) among the reported Pyrus S-RNase genes. Table S16 The accession numbers of S-RNase and S-locus F-box genes in Pyrus, Malus, and Prunus.Table S17 Primers used in this study. Figure S1 Isolation of the conserved F-box motif in the reported S-locus F-box proteins in Pyrus and Malus. The accession numbers of these F-box proteins were listed in Table S13. Figure S2 Phylogenetic classifications of S-locus F-box genes in Prunus. The S-locus F-box (SLF/SFB) proteins in Prunus comprised by 12 groups, SLF1→SLF11 and SFB. Each group were highlighted with different colors. Figure S3 Phylogenetic analysis of the F-box genes identified from this and previous studies. Cycles with black color present the F-box genes identified from previous study (Huang et al., 2023). The rates (%) of different types of gene duplication events (dispersed, proximal, tandem and transposed) of the S-locus F-box ge [file 43897_2024_132_MOESM1_ESM.zip › Supplementary Figures S41 to S50.docx]

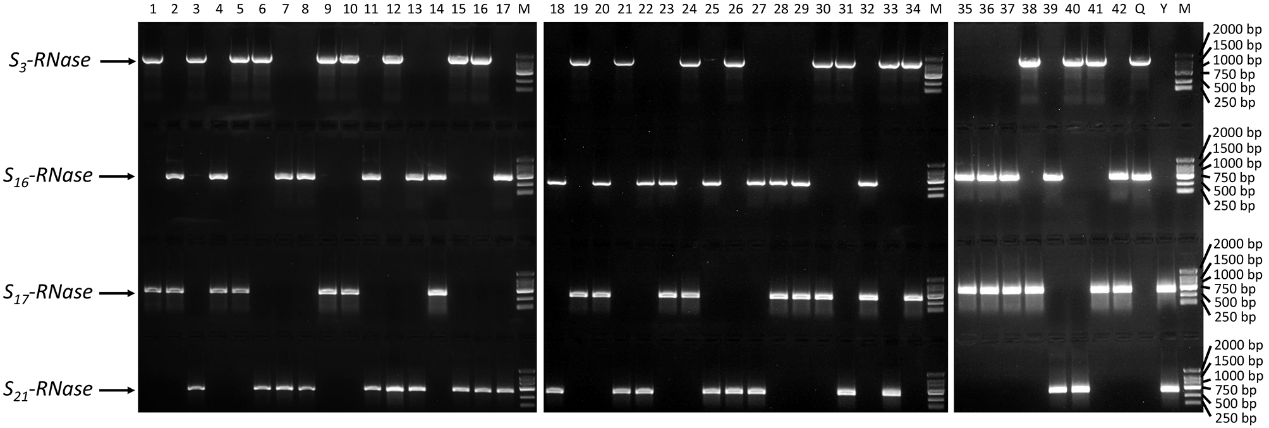


**Figure S41** Identification of *S*-genotypes of the individuals in the cross-pollinated progeny of ‘Yali’ × ‘Xueqing’. The lanes 1-42 represent the individuals. Q and Y represent the pear cultivars ‘Xueqing’ and ‘Yali’, respectively. M indicates the DNA ladder.


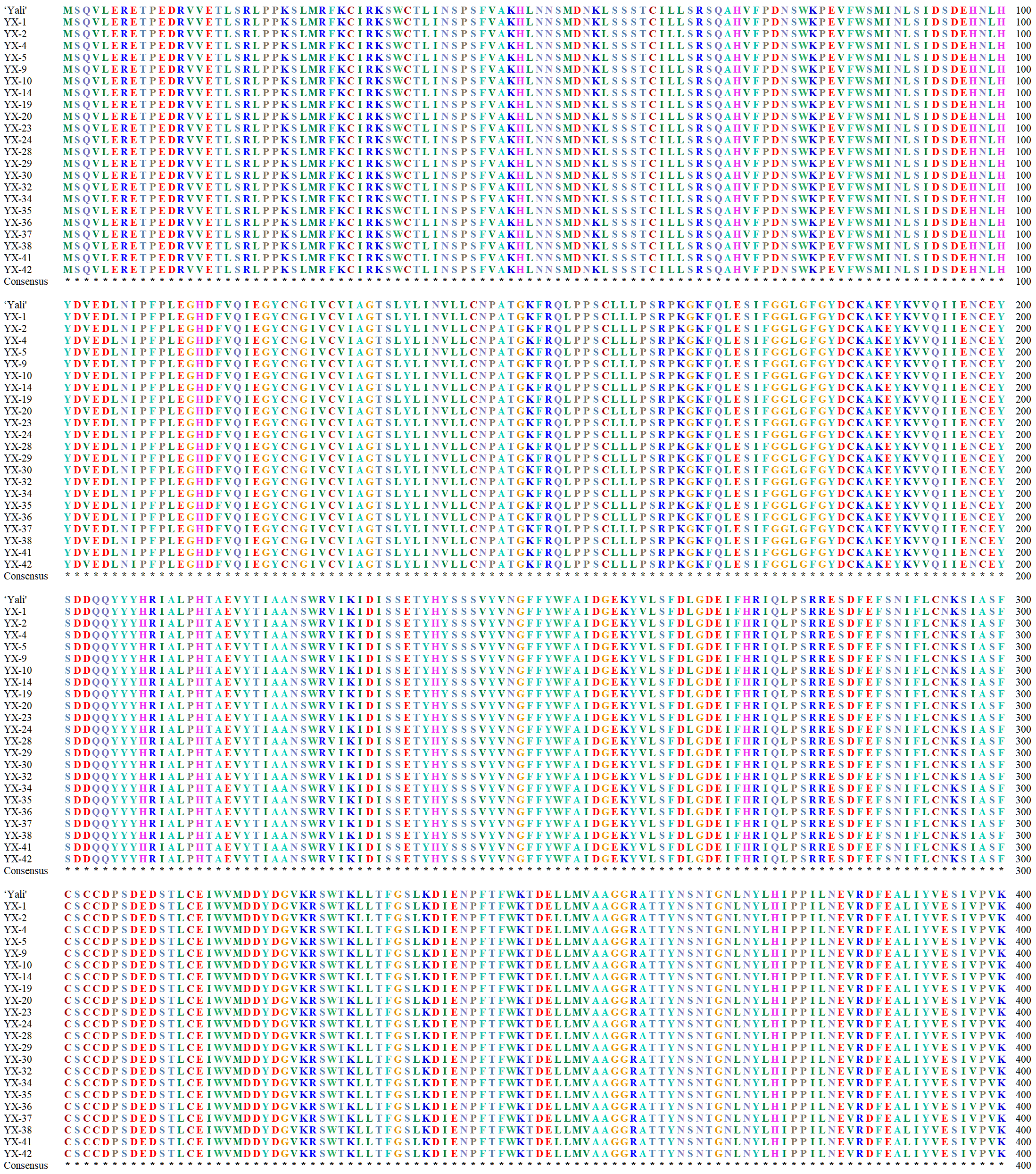


**Figure S42** The amino acid sequences of *PbrSFBB.Ia-S_17_* in any individual including *S_17_-RNase* were identical to that in ‘Yali’. YX-1, 2, 4, 5, 9, 10, 14, 19, 20, 23, 24, 28, 29, 30, 32, 34, 35, 36, 37, 38, 41, and 42 are the individuals of the cross-pollinated progeny of ‘Yali’ × ‘Xueqing’.


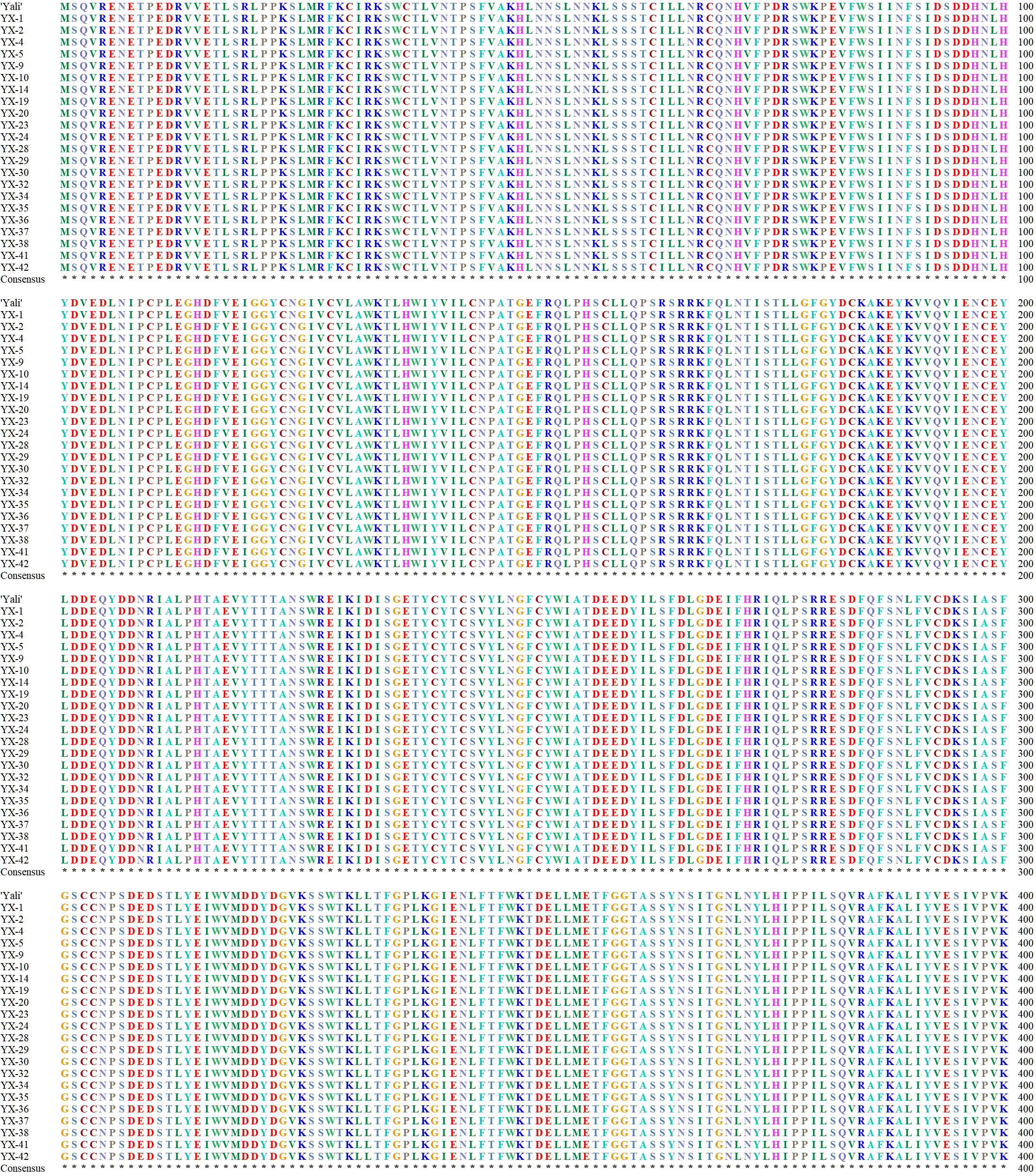


**Figure S43** The amino acid sequences of *PbrSFBB.Ib-S_17_* in any individual including *S_17_-RNase* were identical to that in ‘Yali’. YX-1, 2, 4, 5, 9, 10, 14, 19, 20, 23, 24, 28, 29, 30, 32, 34, 35, 36, 37, 38, 41, and 42 are the individuals of the cross-pollinated progeny of ‘Yali’ × ‘Xueqing’.


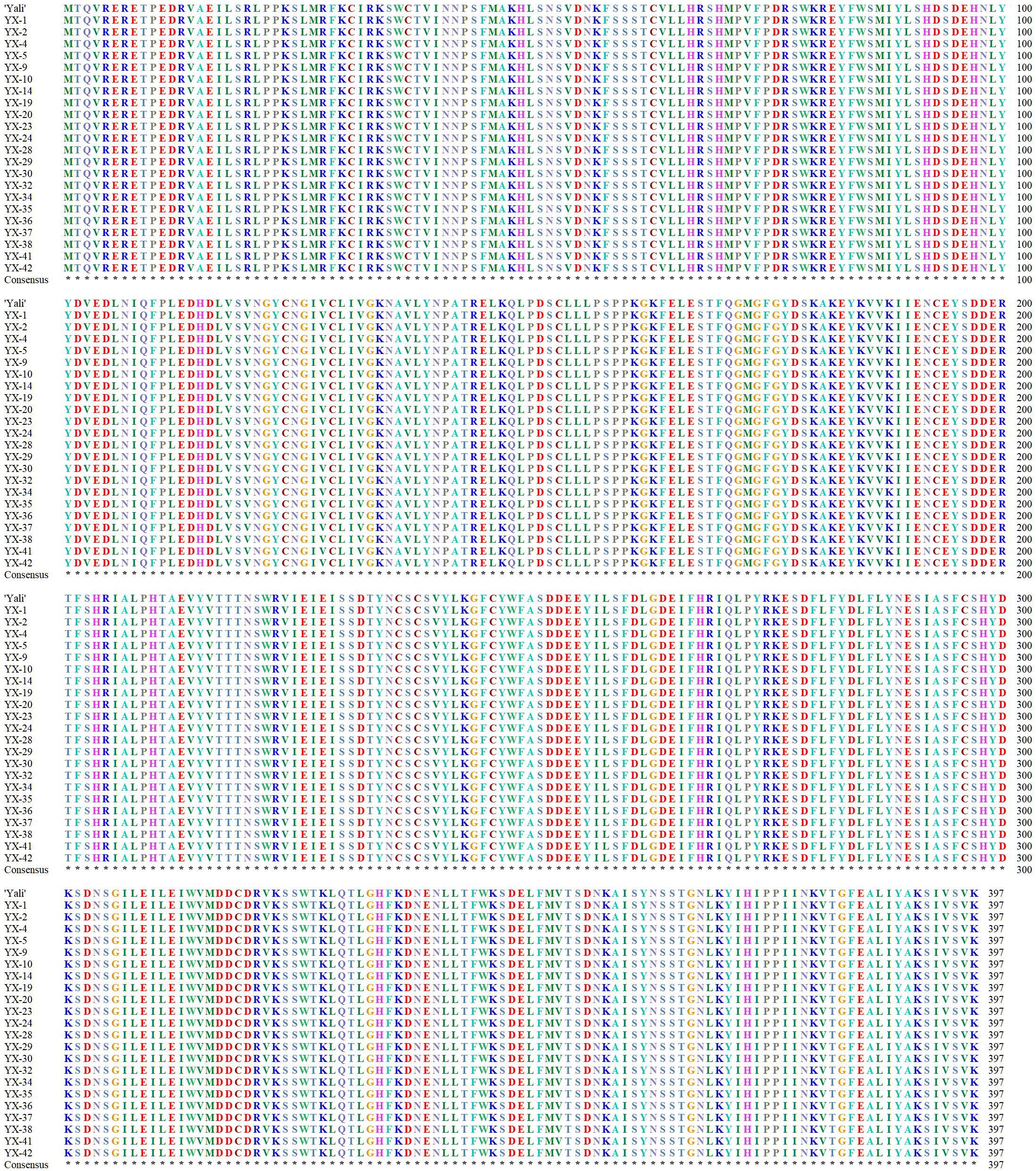


**Figure S44** The amino acid sequences of *PbrSFBB.II-S_17_* in any individual including *S_17_-RNase* were identical to that in ‘Yali’. YX-1, 2, 4, 5, 9, 10, 14, 19, 20, 23, 24, 28, 29, 30, 32, 34, 35, 36, 37, 38, 41, and 42 are the individuals of the cross-pollinated progeny of ‘Yali’ × ‘Xueqing’.


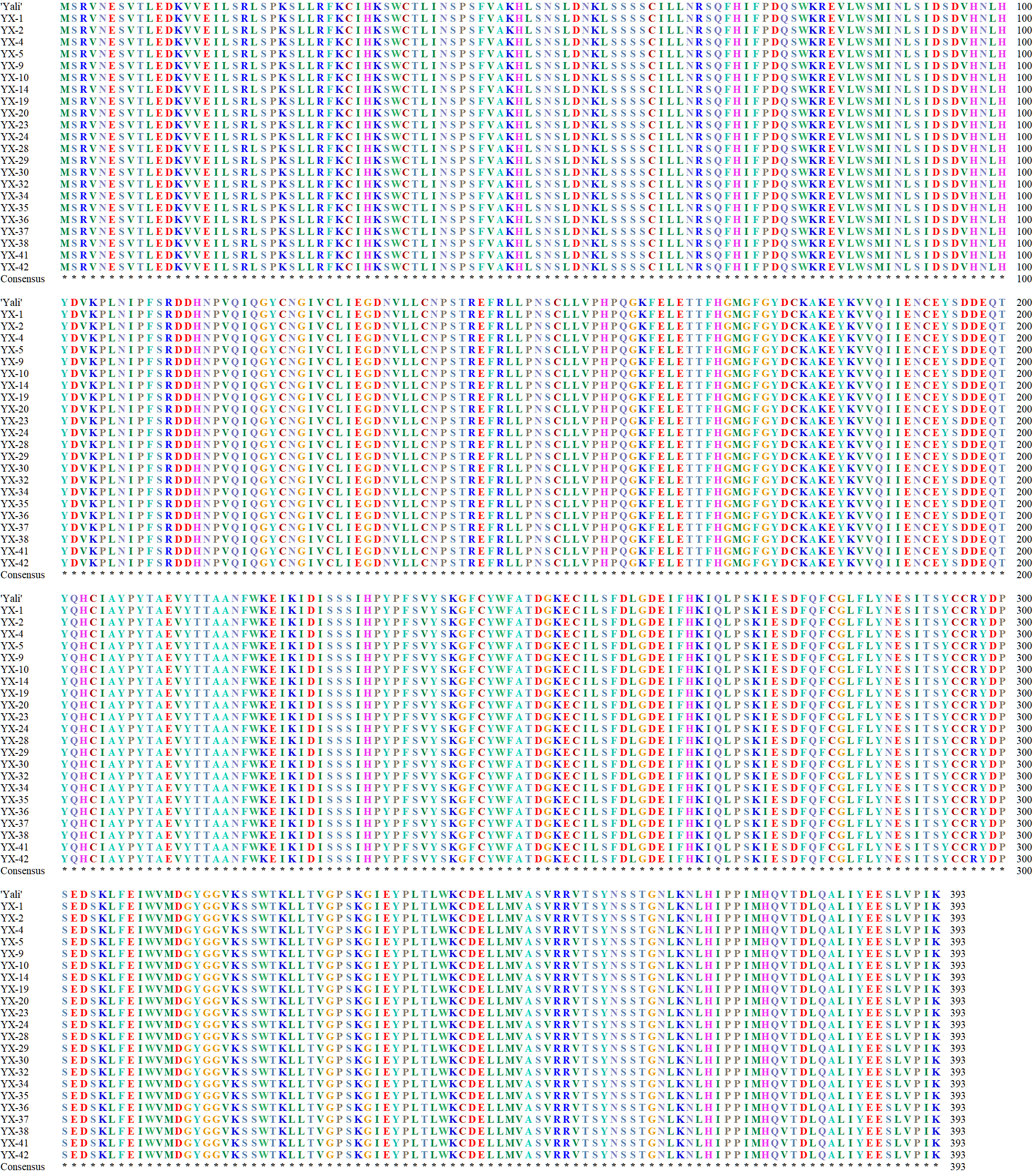


**Figure S45** The amino acid sequences of *PbrSFBB.III-S_17_* in any individual including *S_17_-RNase* were identical to that in ‘Yali’. YX-1, 2, 4, 5, 9, 10, 14, 19, 20, 23, 24, 28, 29, 30, 32, 34, 35, 36, 37, 38, 41, and 42 are the individuals of the cross-pollinated progeny of ‘Yali’ × ‘Xueqing’.


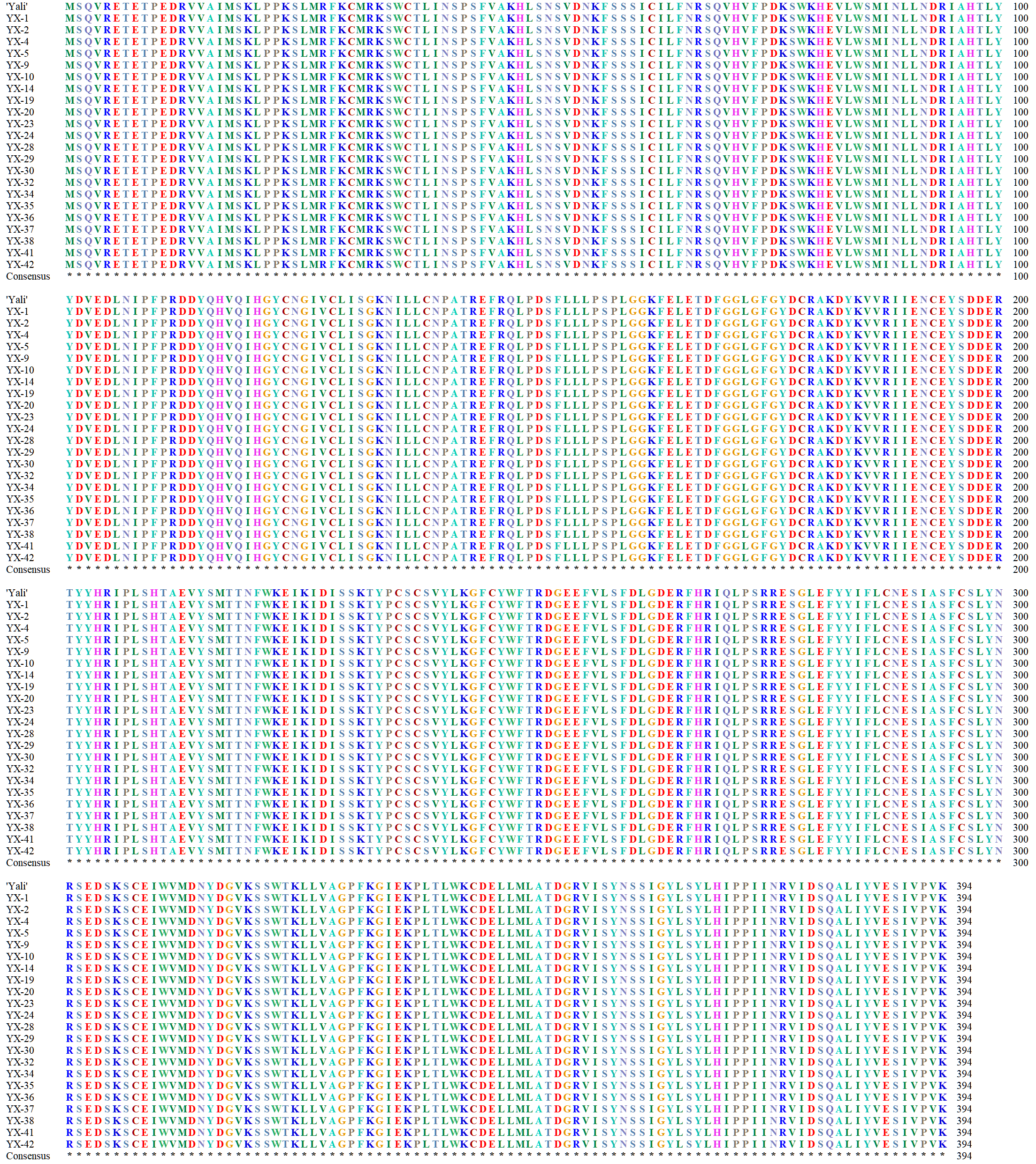


**Figure S46** The amino acid sequences of *PbrSFBB.IV-S_17_* in any individual including *S_17_-RNase* were identical to that in ‘Yali’. YX-1, 2, 4, 5, 9, 10, 14, 19, 20, 23, 24, 28, 29, 30, 32, 34, 35, 36, 37, 38, 41, and 42 are the individuals of the cross-pollinated progeny of ‘Yali’ × ‘Xueqing’.


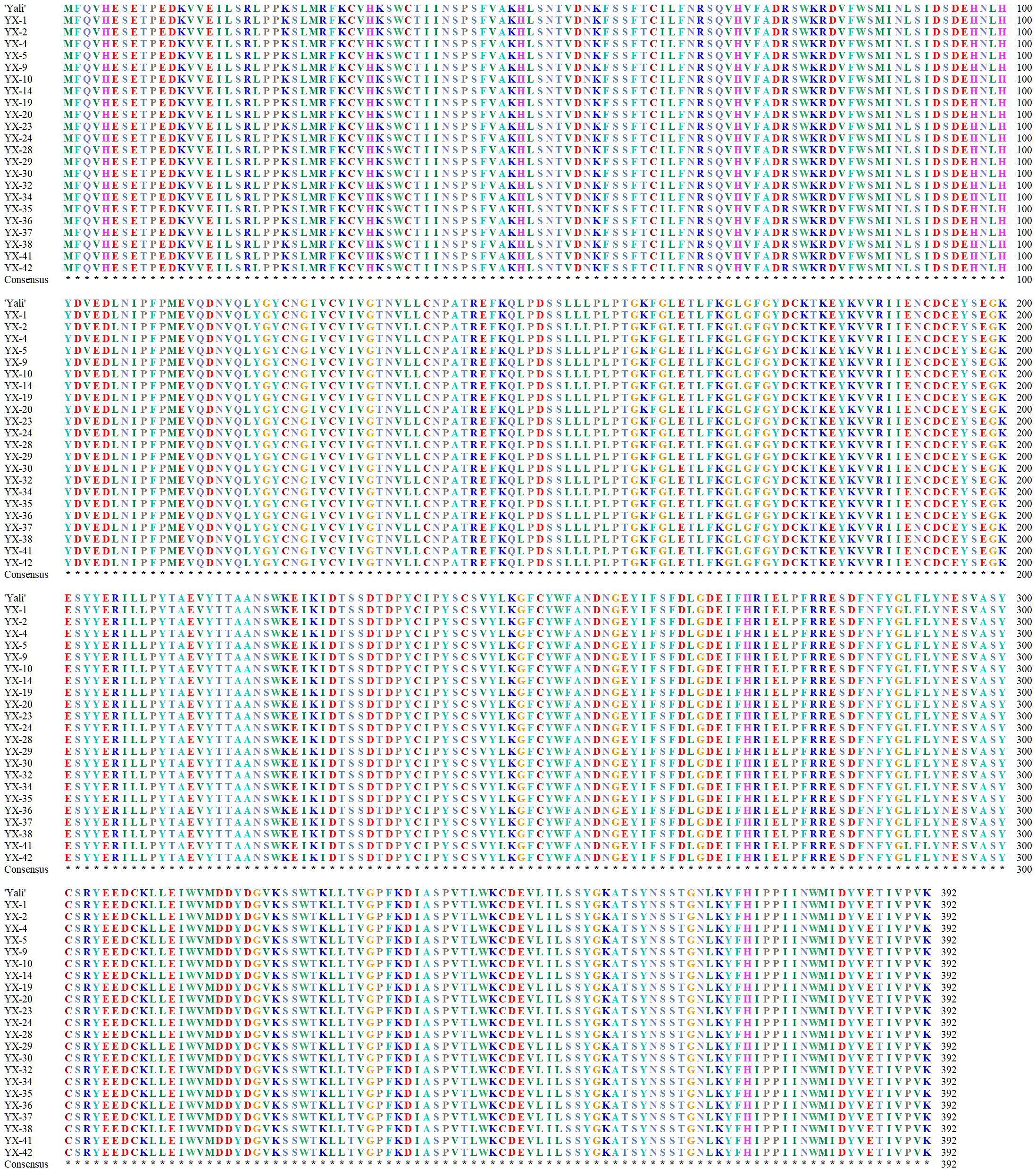


**Figure S47** The amino acid sequences of *PbrSFBB.VI-S_17_* in any individual including *S_17_-RNase* were identical to that in ‘Yali’. YX-1, 2, 4, 5, 9, 10, 14, 19, 20, 23, 24, 28, 29, 30, 32, 34, 35, 36, 37, 38, 41, and 42 are the individuals of the cross-pollinated progeny of ‘Yali’ × ‘Xueqing’.


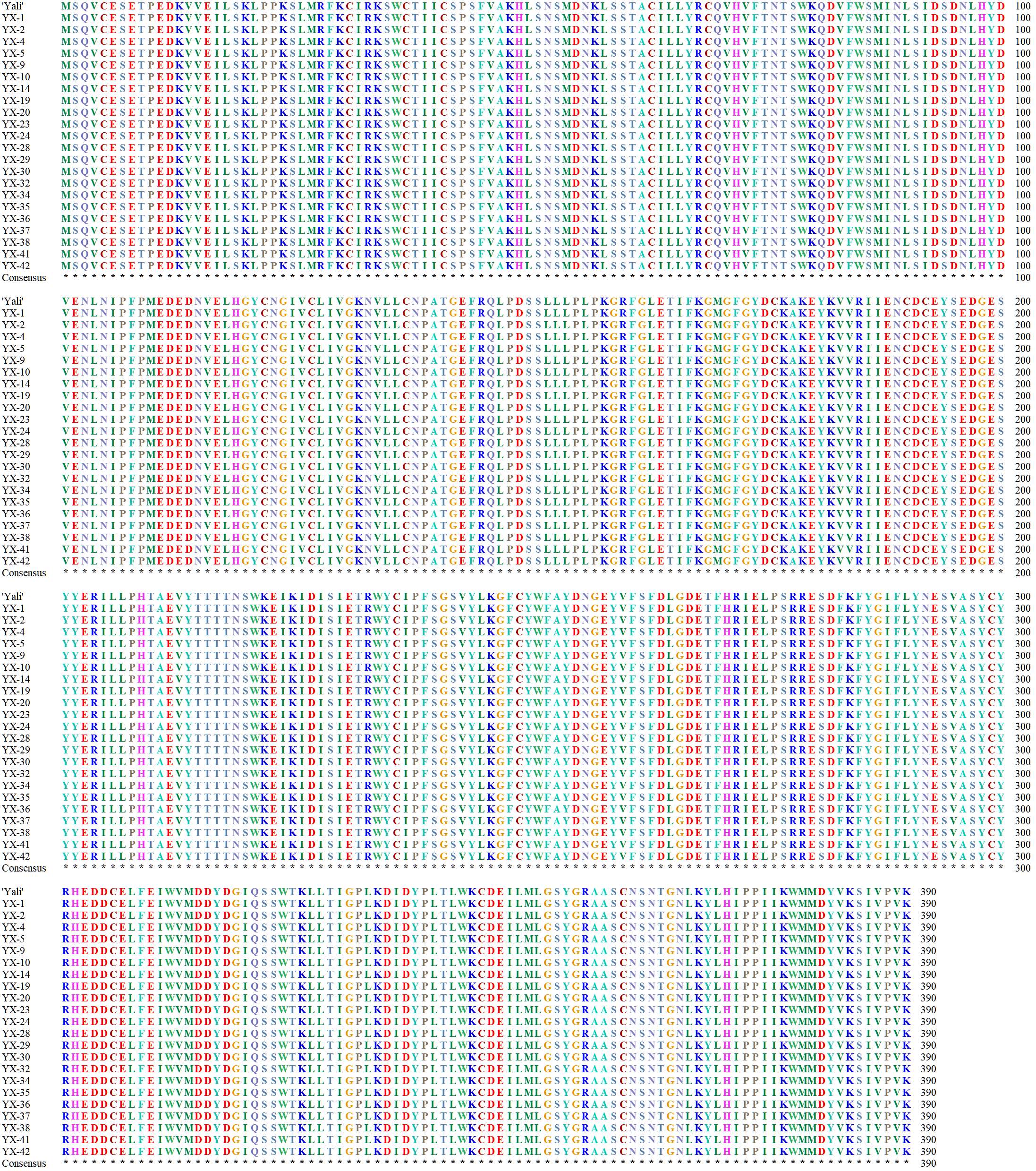


**Figure S48** The amino acid sequences of *PbrSFBB.VII-S_17_* in any individual including *S_17_-RNase* were identical to that in ‘Yali’. YX-1, 2, 4, 5, 9, 10, 14, 19, 20, 23, 24, 28, 29, 30, 32, 34, 35, 36, 37, 38, 41, and 42 are the individuals of the cross-pollinated progeny of ‘Yali’ × ‘Xueqing’.


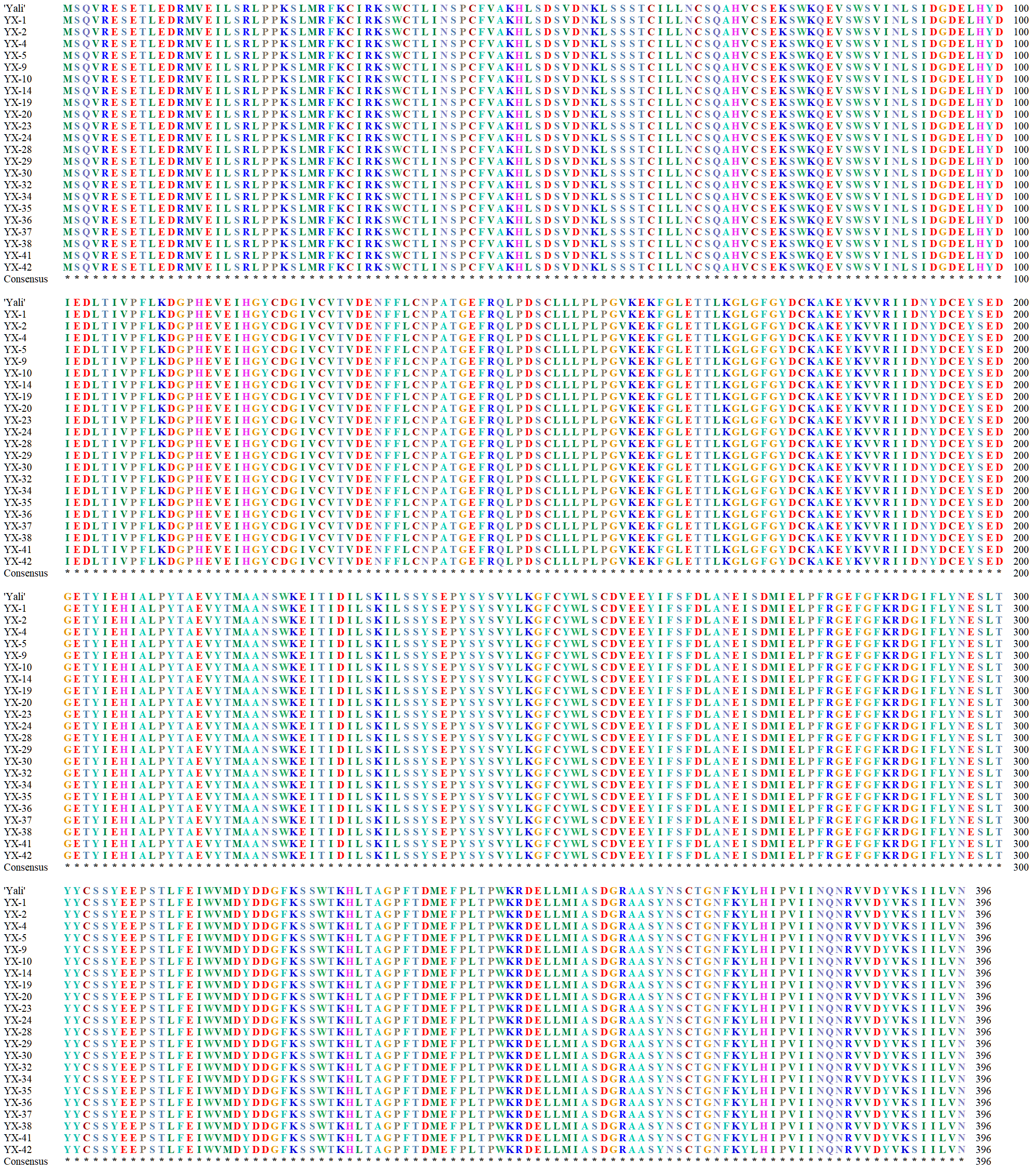


**Figure S49** The amino acid sequences of *PbrSFBB.VIII-S_17_* in any individual including *S_17_-RNase* were identical to that in ‘Yali’. YX-1, 2, 4, 5, 9, 10, 14, 19, 20, 23, 24, 28, 29, 30, 32, 34, 35, 36, 37, 38, 41, and 42 are the individuals of the cross-pollinated progeny of ‘Yali’ × ‘Xueqing’.


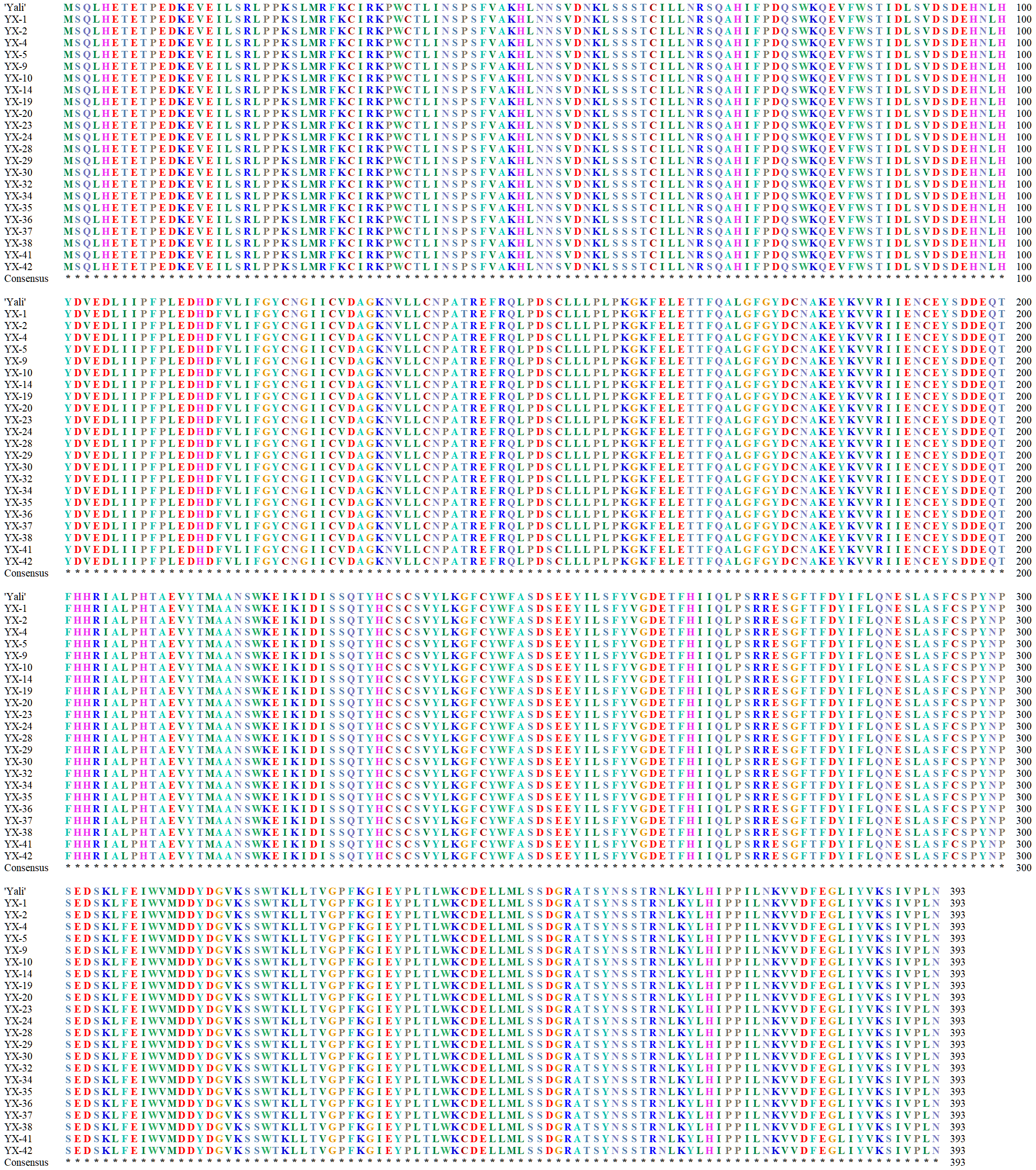


**Figure S50** The amino acid sequences of *PbrSFBB.X-S_17_* in any individual including *S_17_-RNase* were identical to that in ‘Yali’. YX-1, 2, 4, 5, 9, 10, 14, 19, 20, 23, 24, 28, 29, 30, 32, 34, 35, 36, 37, 38, 41, and 42 are the individuals of the cross-pollinated progeny of ‘Yali’ × ‘Xueqing’.
