# Supplementary material for: Long-read genome sequencing reveals the sequence characteristics of pear self-incompatibility locus
Source: Mol Hortic. 2025 Mar 1;5:13. doi: 10.1186/s43897-024-00132-0 (PMC11871771; doi:10.1186/s43897-024-00132-0)
Supplement: Supplementary file 1 — Supplementary Material 1: Table S1 Comparison of ‘Yali’ genome with previously published assemblies of Pyrus and Malus species. Table S2 Annotation of the repeats in ‘Yali’ genome. Table S3 Annotation of the non-coding RNAs in ‘Dananguo’ and 'Yali' genomes. Table S4 Identification of the F-box genes in Pyrus, Malus and Prunus S-loci. Table S5 Function annotation of the predicted genes in S-loci. Table S6 Sequence similarity (%) among Pyrus and Malus SFBB genes. Table S7 Sequence similarity (%) among Prunus SFB and SLF genes. Table S8 Sequence similarity among Prunus SFB and SLF genes. Table S9 Sequence similarity (%) among Pyrus and Malus S-RNase genes. Table S10 Prediction of gene duplication events of Pyrus and Malus SFBB genes. Table S11 Sequence similarity of the non-coding flanking sequences of SFBBs in Pyrus and Malus S-loci. Table S12 Analysis of number and length of LTR retrotransposon in different S-loci. Table S13 Identification of the LTR retrotransposon in different S-loci. Table S14 RPKM values of the genes commonly existed in the tested S-loci. Table S15 Sequence similarity (%) among the reported Pyrus S-RNase genes. Table S16 The accession numbers of S-RNase and S-locus F-box genes in Pyrus, Malus, and Prunus.Table S17 Primers used in this study. Figure S1 Isolation of the conserved F-box motif in the reported S-locus F-box proteins in Pyrus and Malus. The accession numbers of these F-box proteins were listed in Table S13. Figure S2 Phylogenetic classifications of S-locus F-box genes in Prunus. The S-locus F-box (SLF/SFB) proteins in Prunus comprised by 12 groups, SLF1→SLF11 and SFB. Each group were highlighted with different colors. Figure S3 Phylogenetic analysis of the F-box genes identified from this and previous studies. Cycles with black color present the F-box genes identified from previous study (Huang et al., 2023). The rates (%) of different types of gene duplication events (dispersed, proximal, tandem and transposed) of the S-locus F-box ge [file 43897_2024_132_MOESM1_ESM.zip › Supplementary Figures S51 to S62.docx]

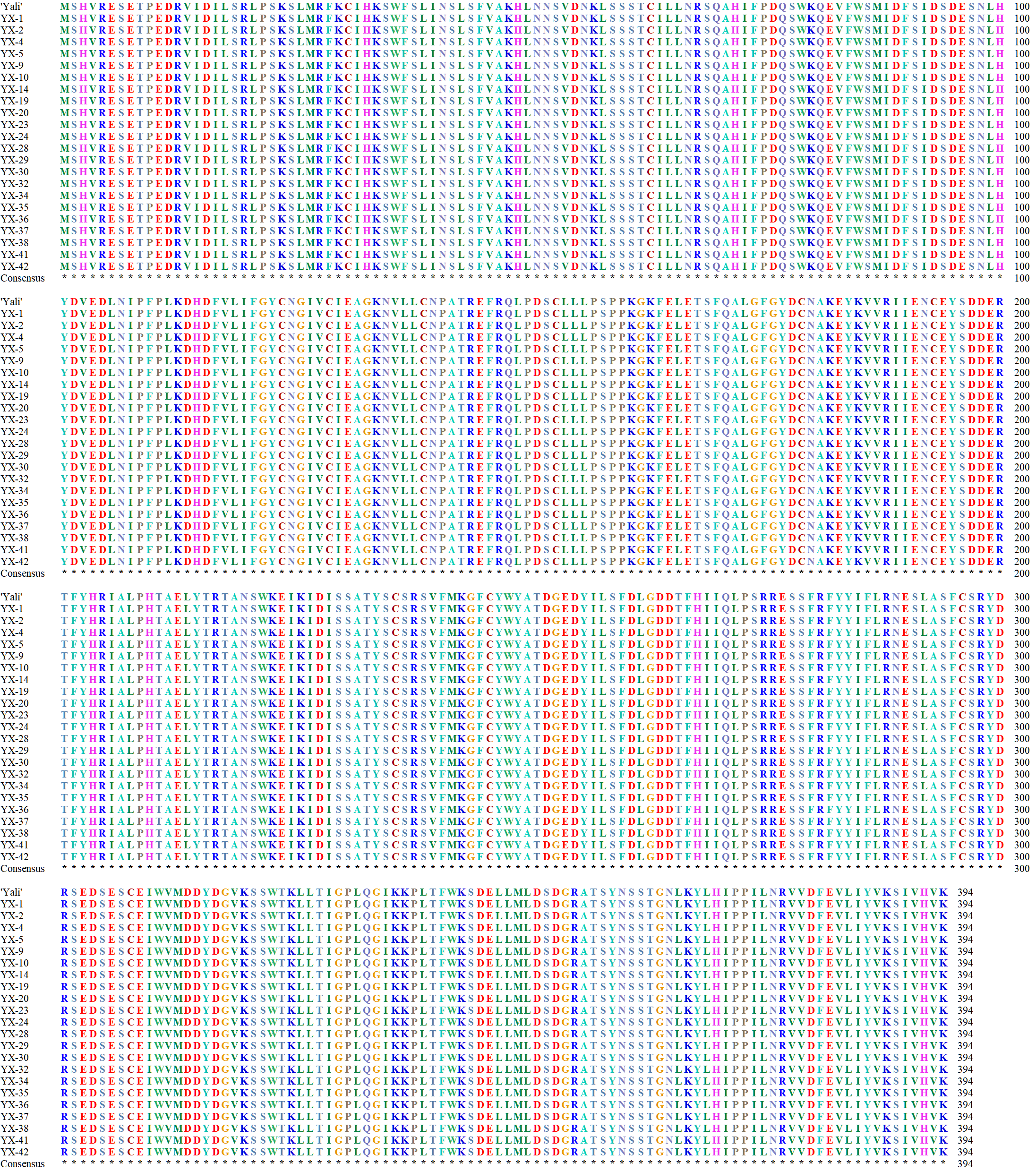


**Figure S51** The amino acid sequences of *PbrSFBB.XI-S_17_* in any individual including *S_17_-RNase* were identical to that in ‘Yali’. YX-1, 2, 4, 5, 9, 10, 14, 19, 20, 23, 24, 28, 29, 30, 32, 34, 35, 36, 37, 38, 41, and 42 are the individuals of the cross-pollinated progeny of ‘Yali’ × ‘Xueqing’.


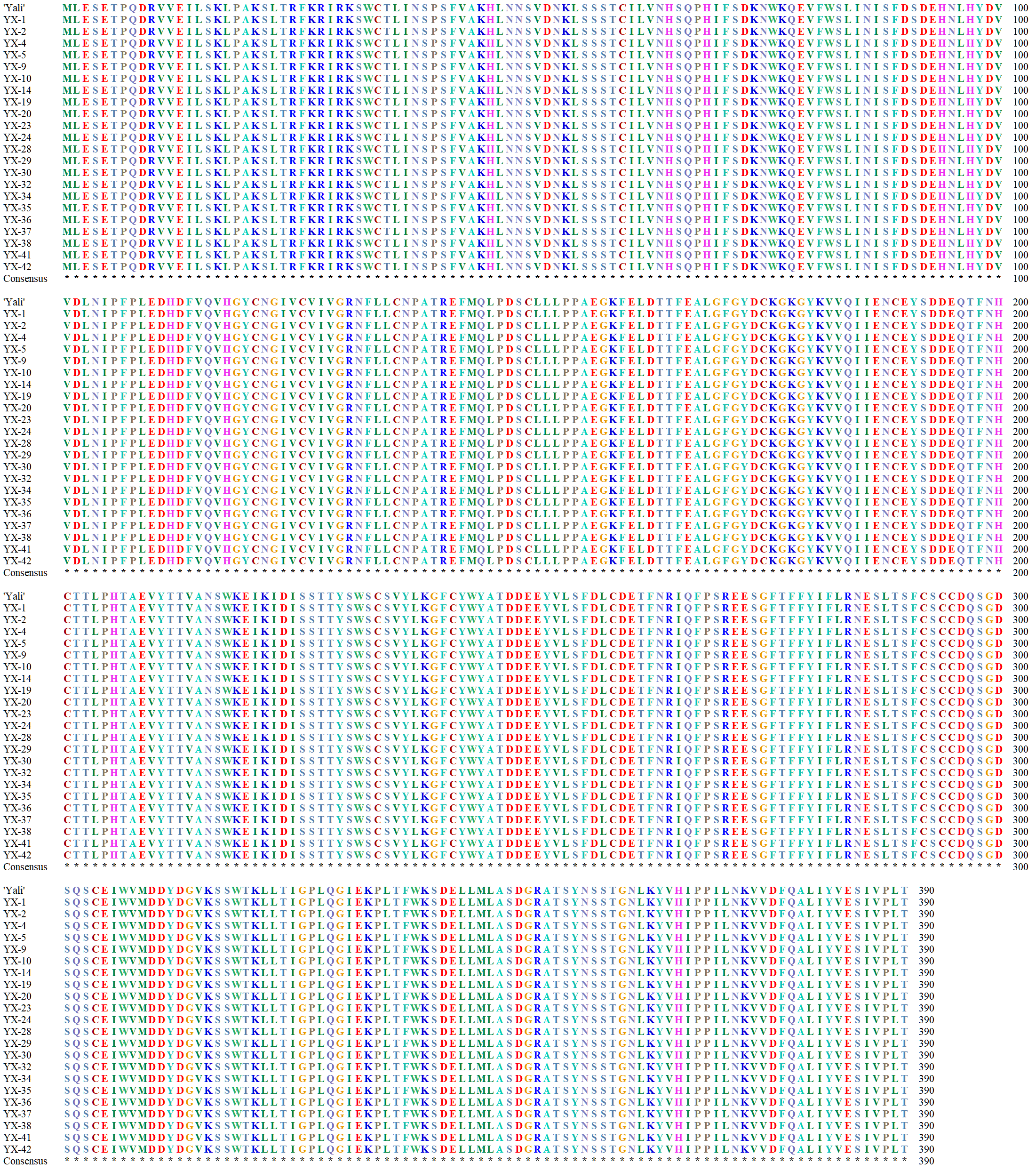


**Figure S52** The amino acid sequences of *PbrSFBB.XII-S_17_* in any individual including *S_17_-RNase* were identical to that in ‘Yali’. YX-1, 2, 4, 5, 9, 10, 14, 19, 20, 23, 24, 28, 29, 30, 32, 34, 35, 36, 37, 38, 41, and 42 are the individuals of the cross-pollinated progeny of ‘Yali’ × ‘Xueqing’.


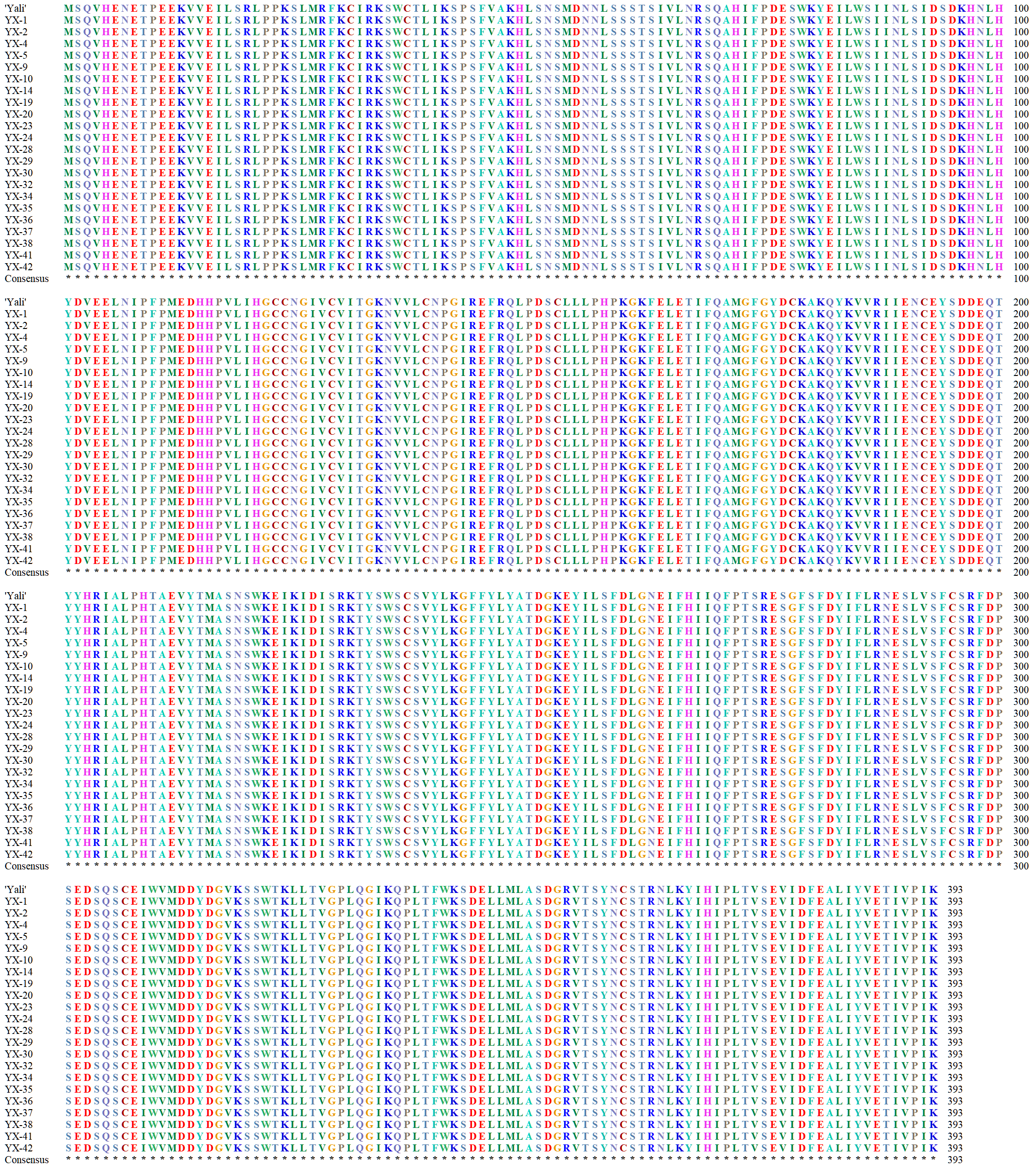


**Figure S53** The amino acid sequences of *PbrSFBB.XIII-S_17_* in any individual including *S_17_-RNase* were identical to that in ‘Yali’. YX-1, 2, 4, 5, 9, 10, 14, 19, 20, 23, 24, 28, 29, 30, 32, 34, 35, 36, 37, 38, 41, and 42 are the individuals of the cross-pollinated progeny of ‘Yali’ × ‘Xueqing’.


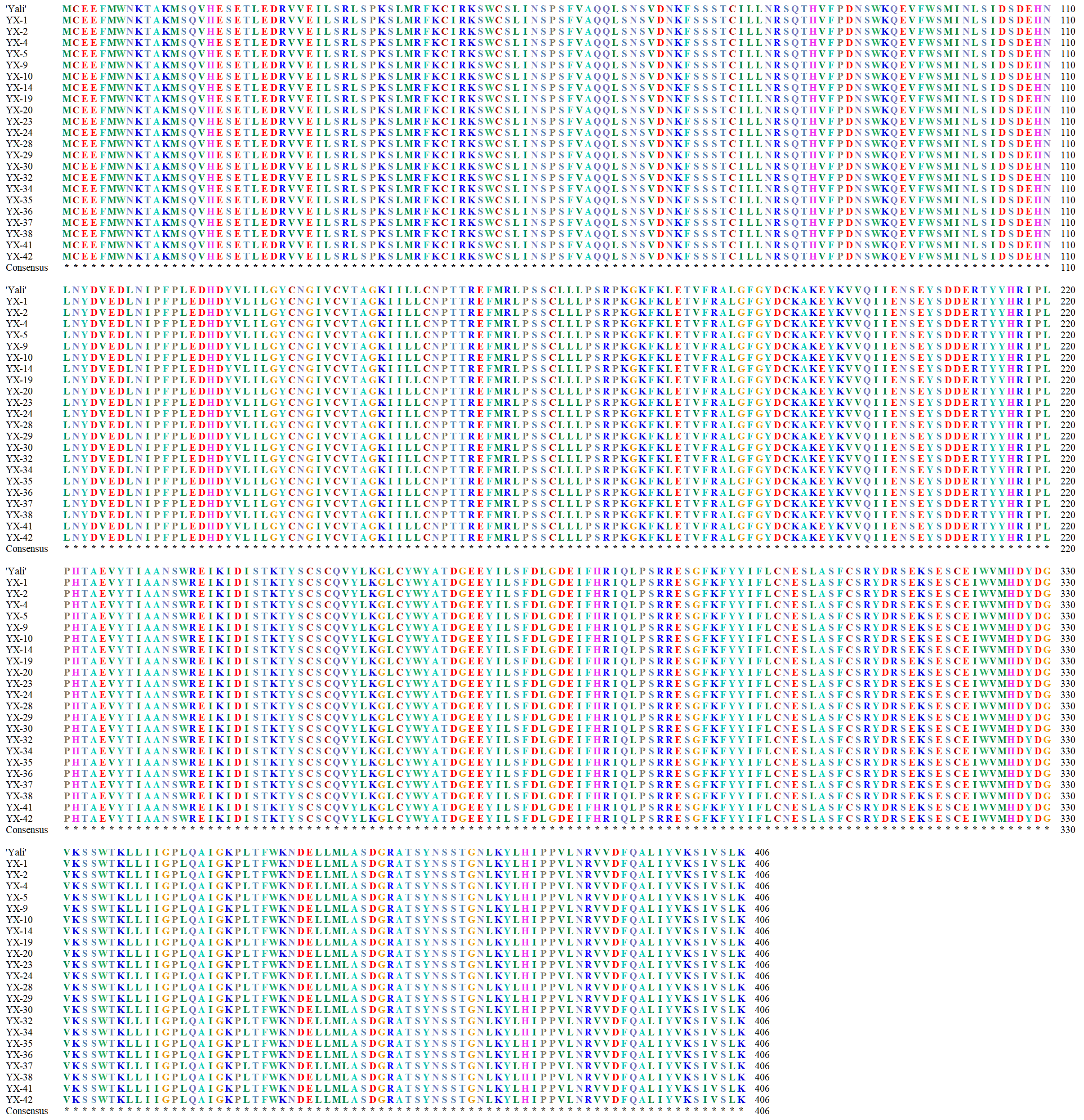


**Figure S54** The amino acid sequences of *PbrSFBB.XIV-S_17_* in any individual including *S_17_-RNase* were identical to that in ‘Yali’. YX-1, 2, 4, 5, 9, 10, 14, 19, 20, 23, 24, 28, 29, 30, 32, 34, 35, 36, 37, 38, 41, and 42 are the individuals of the cross-pollinated progeny of ‘Yali’ × ‘Xueqing’.


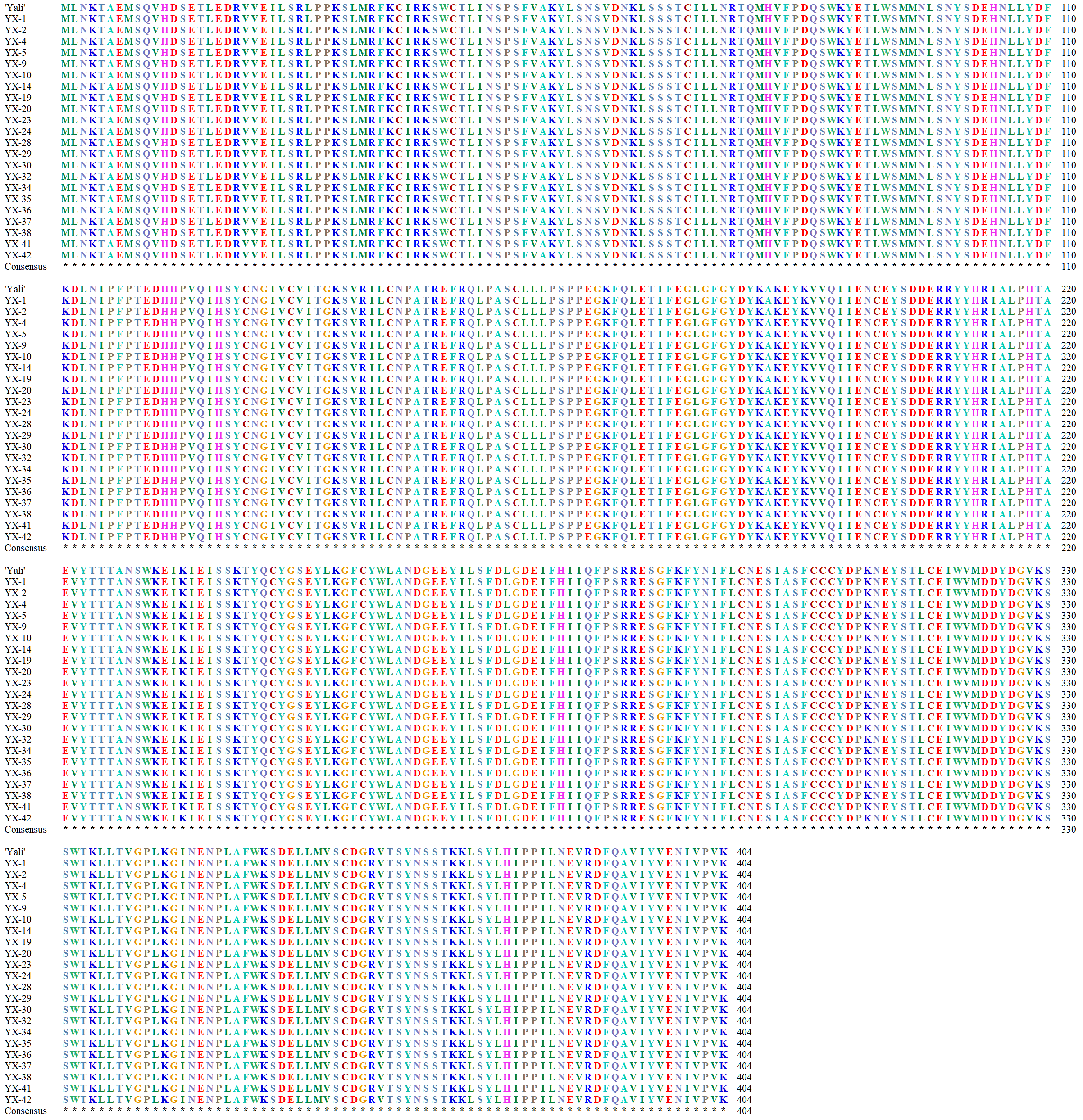


**Figure S55** The amino acid sequences of *PbrSFBB.XV-S_17_* in any individual including *S_17_-RNase* were identical to that in ‘Yali’. YX-1, 2, 4, 5, 9, 10, 14, 19, 20, 23, 24, 28, 29, 30, 32, 34, 35, 36, 37, 38, 41, and 42 are the individuals of the cross-pollinated progeny of ‘Yali’ × ‘Xueqing’.


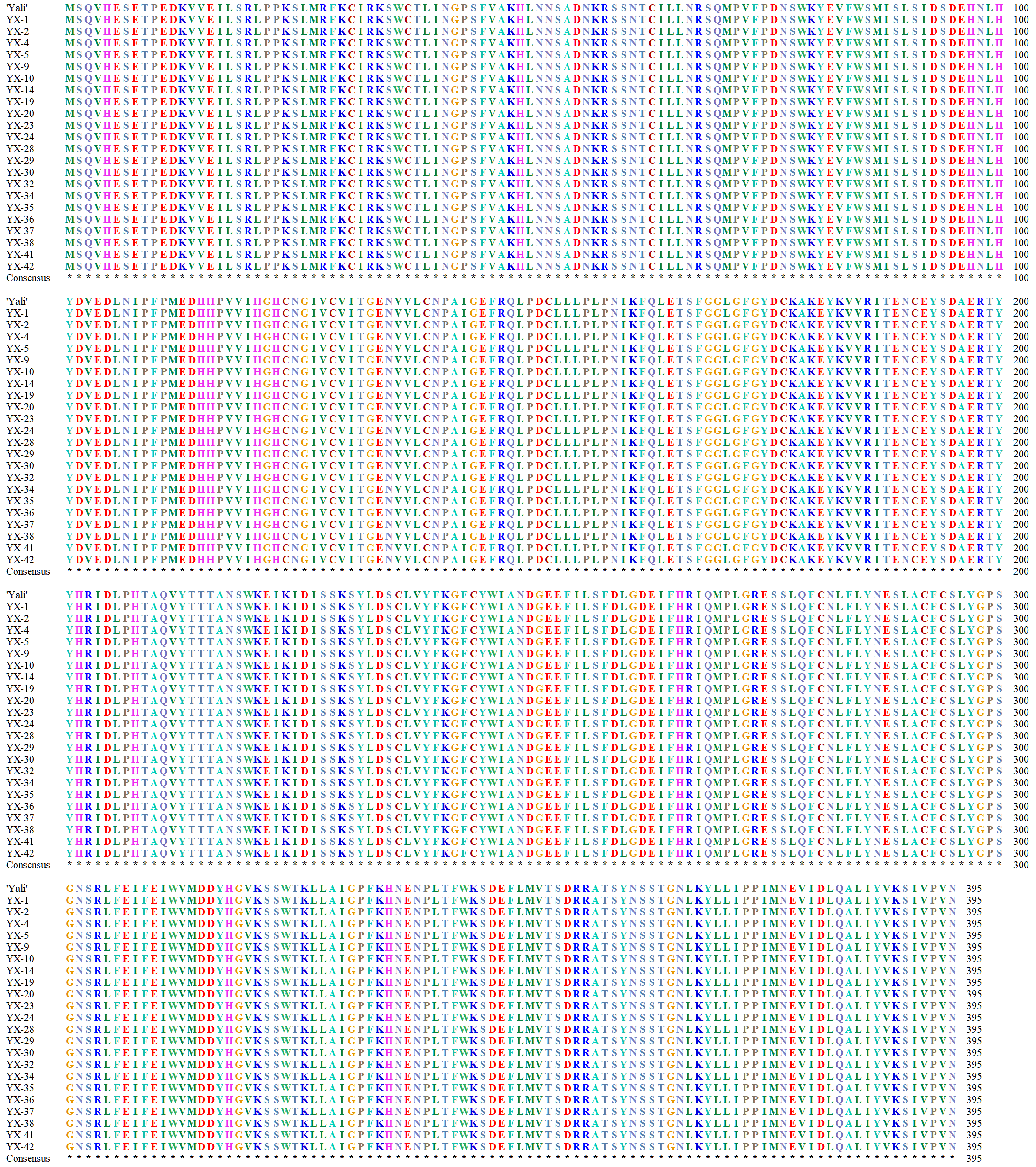


**Figure S56** The amino acid sequences of *PbrSFBB.XVI-S_17_* in any individual including *S_17_-RNase* were identical to that in ‘Yali’. YX-1, 2, 4, 5, 9, 10, 14, 19, 20, 23, 24, 28, 29, 30, 32, 34, 35, 36, 37, 38, 41, and 42 are the individuals of the cross-pollinated progeny of ‘Yali’ × ‘Xueqing’.


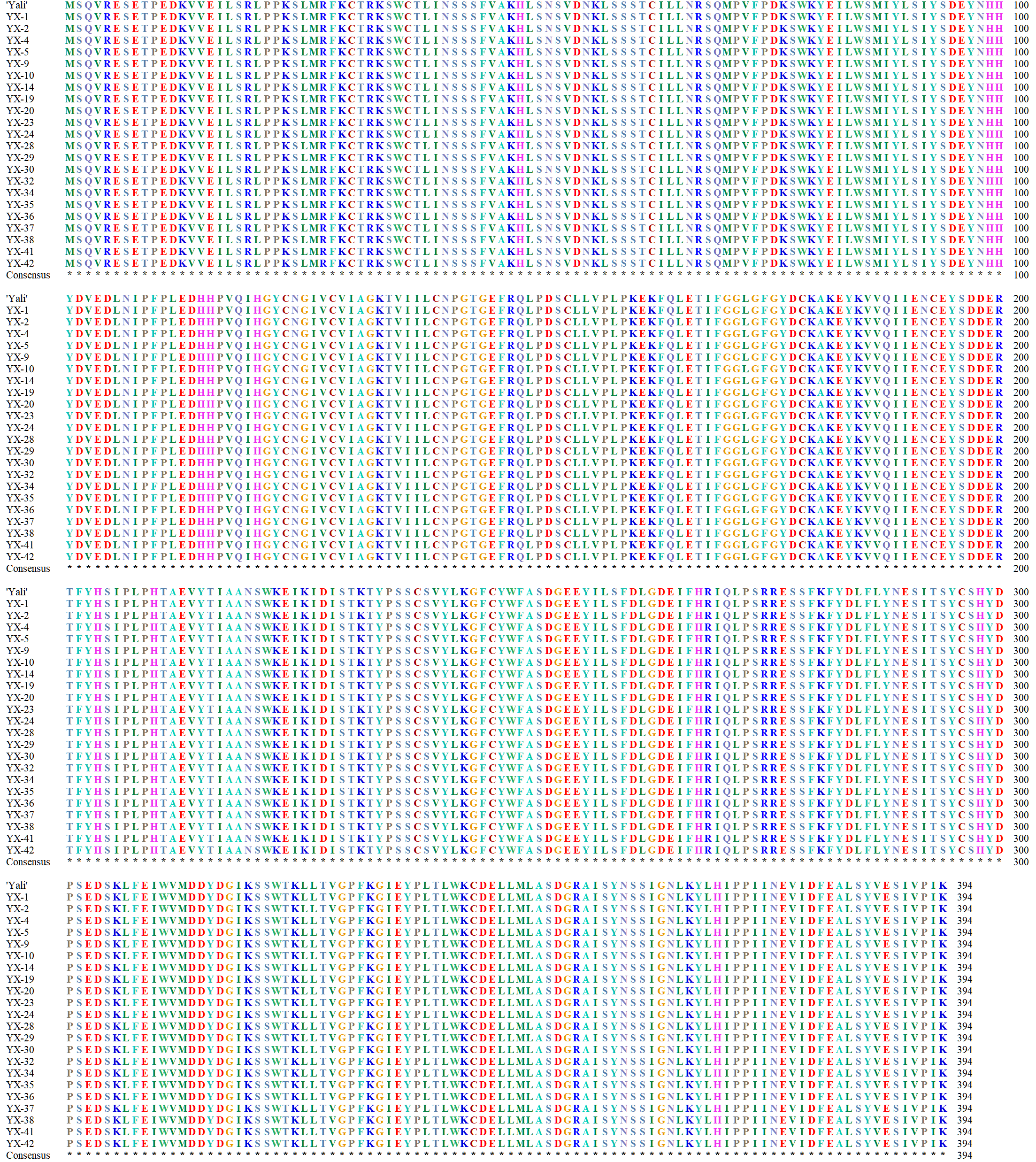


**Figure S57** The amino acid sequences of *PbrSFBB.XVIII-S_17_* in any individual including *S_17_-RNase* were identical to that in ‘Yali’. YX-1, 2, 4, 5, 9, 10, 14, 19, 20, 23, 24, 28, 29, 30, 32, 34, 35, 36, 37, 38, 41, and 42 are the individuals of the cross-pollinated progeny of ‘Yali’ × ‘Xueqing’.


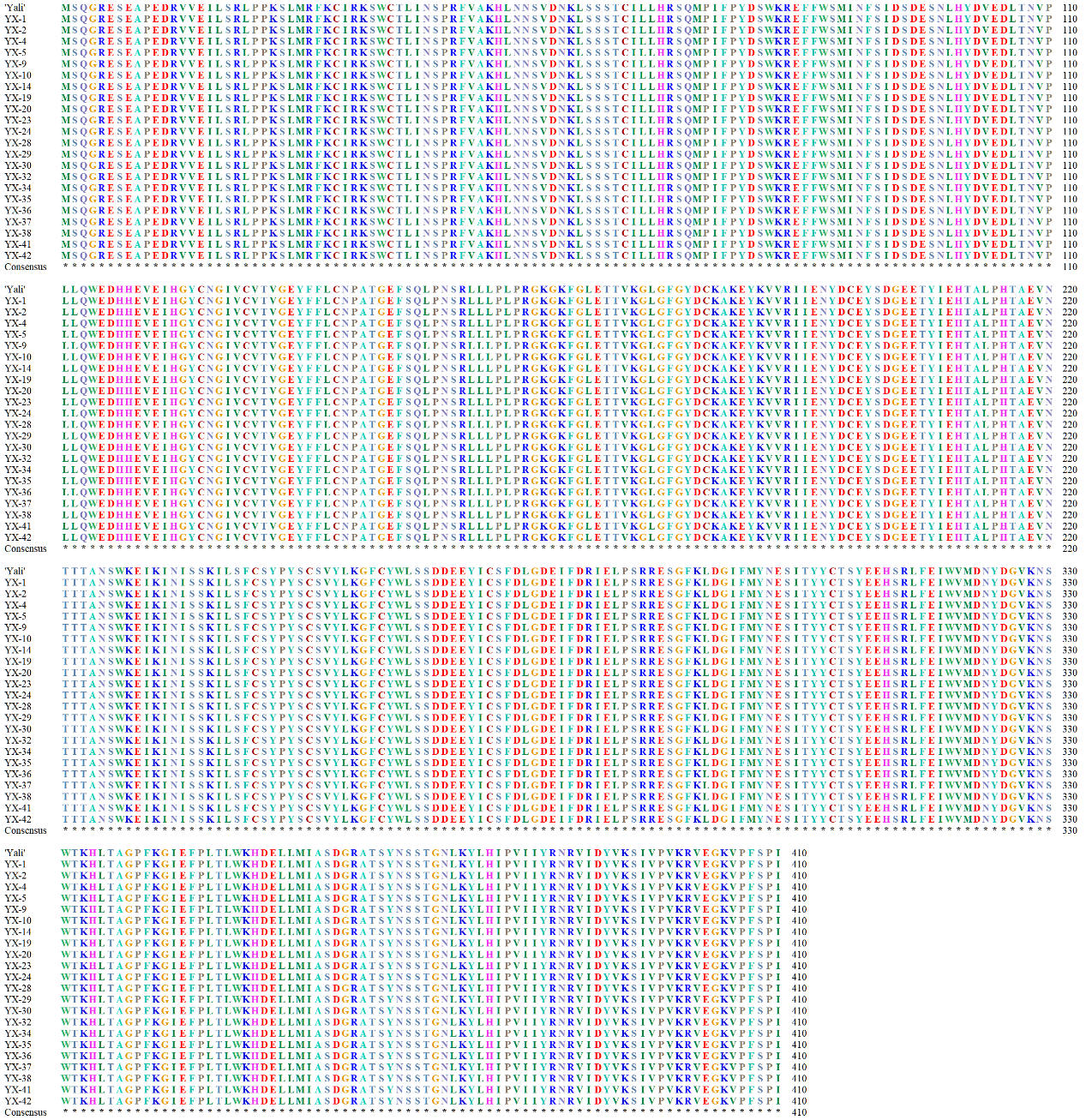


**Figure S58** The amino acid sequences of *PbrSFBB.XIX-S_17_* in any individual including *S_17_-RNase* were identical to that in ‘Yali’. YX-1, 2, 4, 5, 9, 10, 14, 19, 20, 23, 24, 28, 29, 30, 32, 34, 35, 36, 37, 38, 41, and 42 are the individuals of the cross-pollinated progeny of ‘Yali’ × ‘Xueqing’.


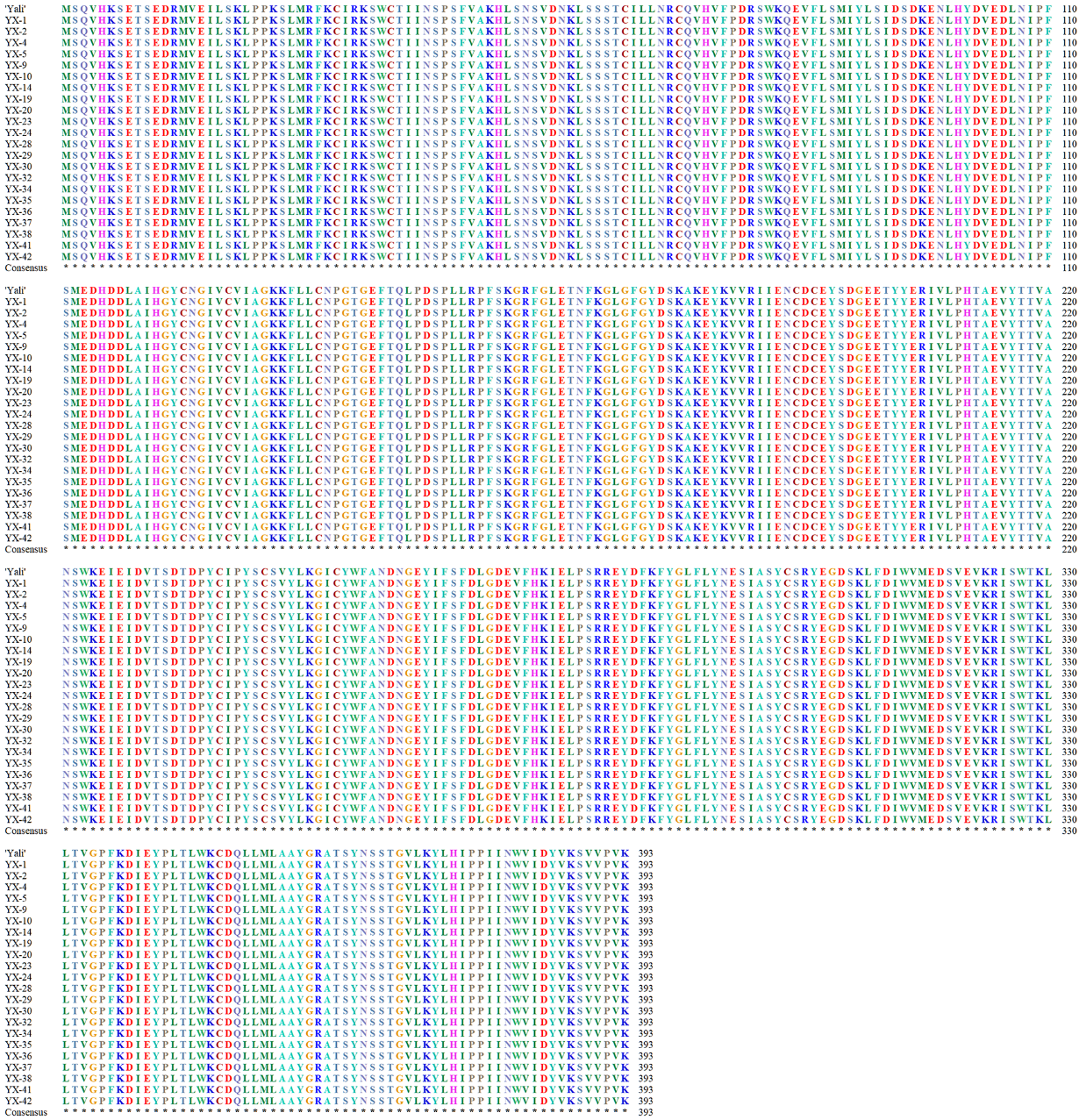


**Figure S59** The amino acid sequences of *PbrSFBB.XX-S_17_* in any individual including *S_17_-RNase* were identical to that in ‘Yali’. YX-1, 2, 4, 5, 9, 10, 14, 19, 20, 23, 24, 28, 29, 30, 32, 34, 35, 36, 37, 38, 41, and 42 are the individuals of the cross-pollinated progeny of ‘Yali’ × ‘Xueqing’.


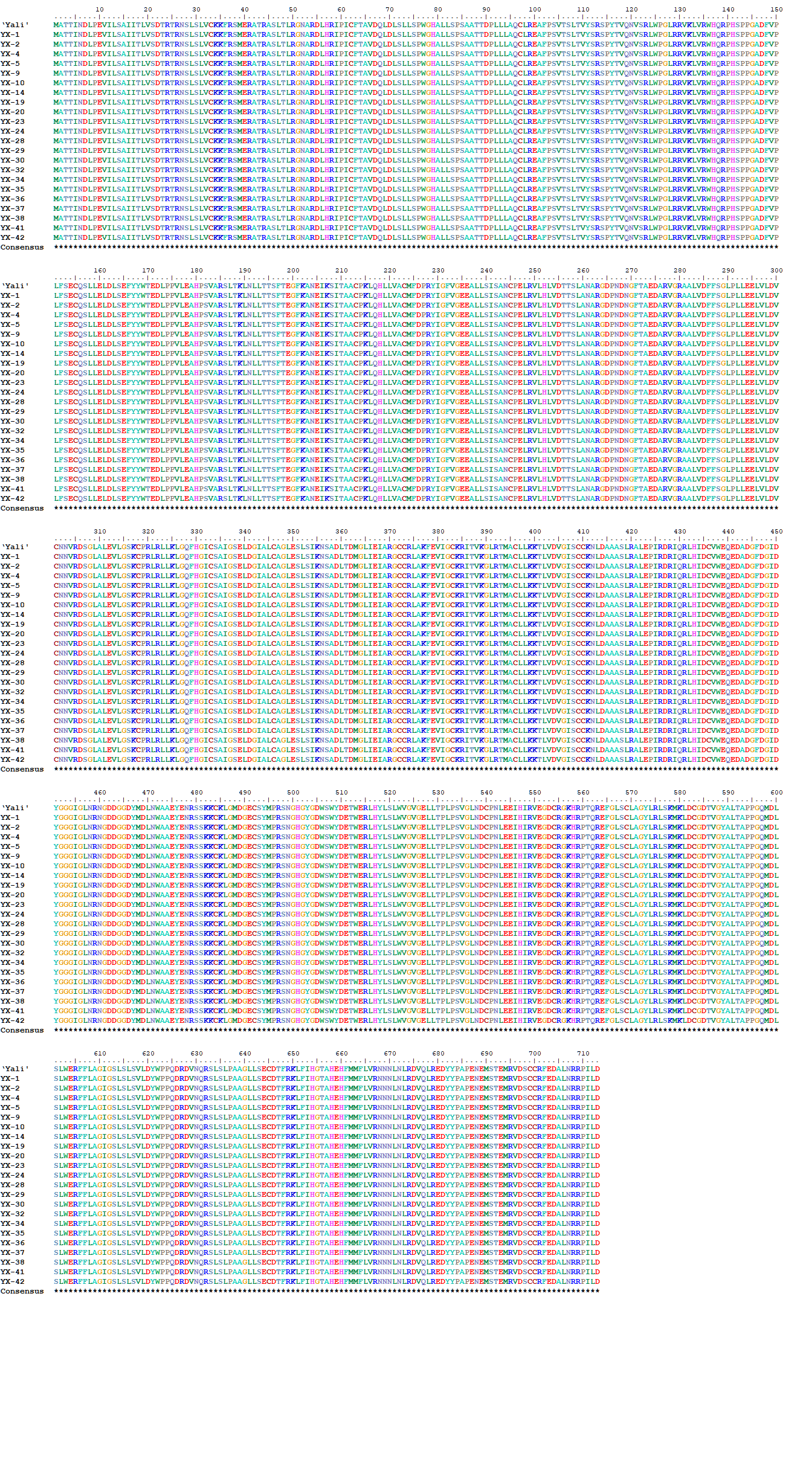


**Figure S60** The amino acid sequences of *PbrSFBB.XXII-S_17_* in any individual including *S_17_-RNase* were identical to that in ‘Yali’. YX-1, 2, 4, 5, 9, 10, 14, 19, 20, 23, 24, 28, 29, 30, 32, 34, 35, 36, 37, 38, 41, and 42 are the individuals of the cross-pollinated progeny of ‘Yali’ × ‘Xueqing’.


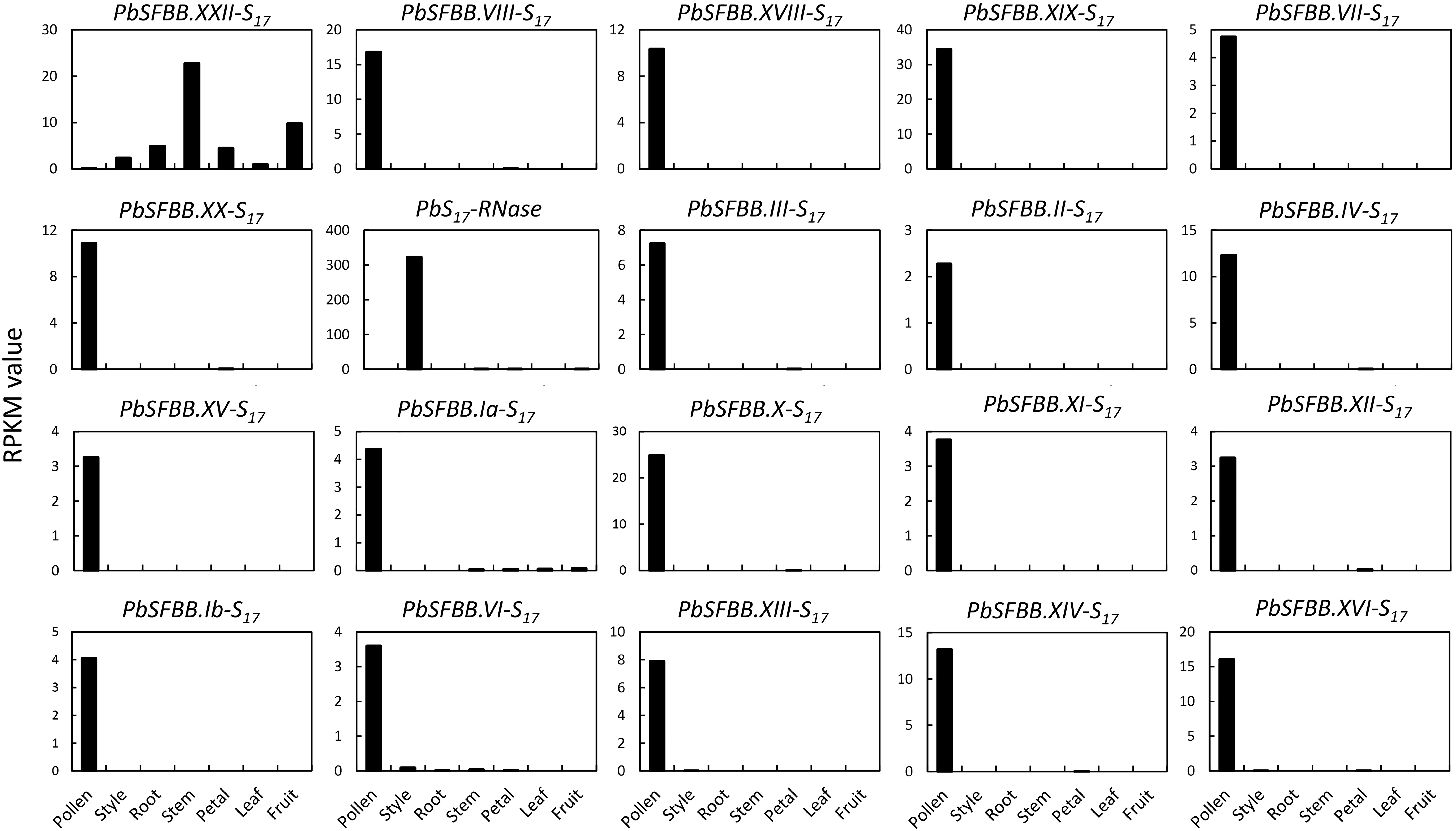


**Figure S61** Expression analysis of *S-RNase* and *SFBB* genes in *Pyrus S_17_*-locus in different tissues.


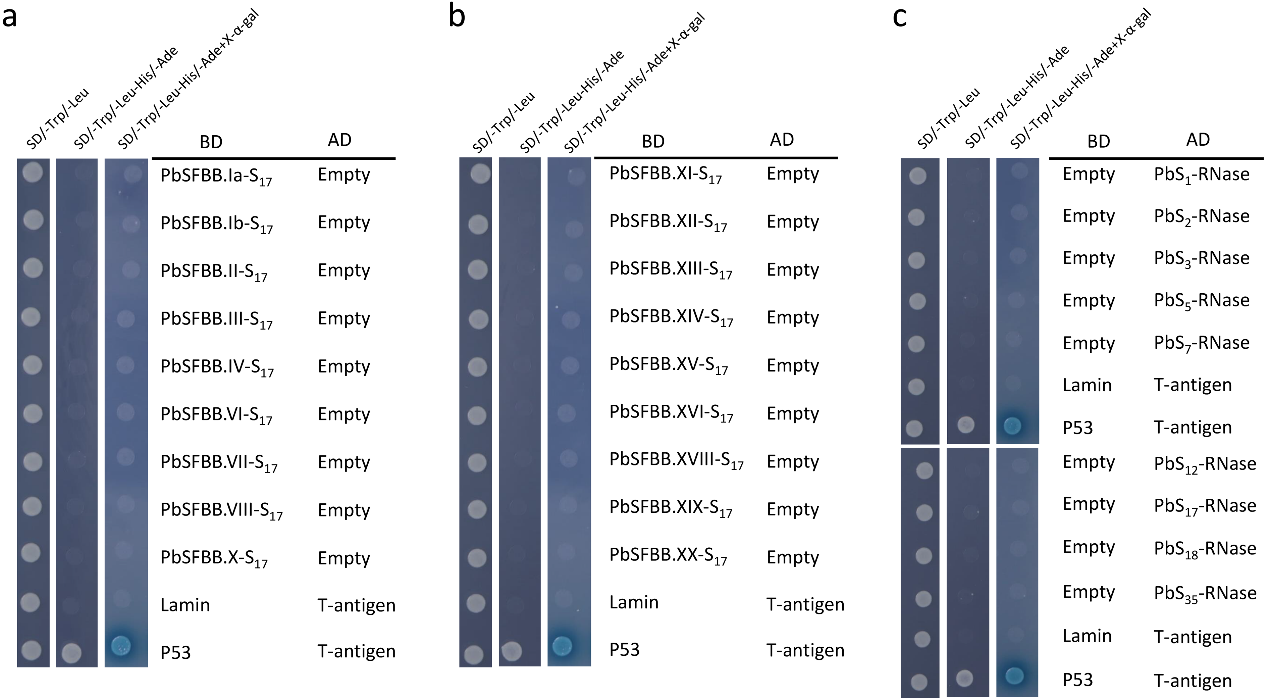


**Figure S62** Self-activation of S-RNase and SFBB proteins in *Pyrus S_17_*-locus in yeast cells. AD and BD present the pGADT7 and pGBKT7 vectors respectively. Positive and negative controls were assigned as P53/T-antigen and Lamin/T-antigen, respectively. SD/-Trp/-Leu indicates the SD medium lacking Trp and Leu; SD/-Trp/-Leu/-His/-Ade indicates the SD medium lacking Trp, Leu, His, and Ade; SD/-Trp/-Leu-His/-Ade + X-α-gal indicates that x-a-gal was mixed into the SD medium lacking Trp, Leu, His, and Ade.
